# Ferroptosis-associated myeloid cell heterogeneity and inflammatory amplification following spinal cord injury

**DOI:** 10.3389/fimmu.2026.1831161

**Published:** 2026-04-22

**Authors:** Jian Zhang, Song Wang, Minghang Zhang, Han Qin, Weiliang Wu, Guoliang Chen, Genlong Jiao

**Affiliations:** 1 Dongguan Key Laboratory of Central Nervous System Injury and Repair/Dongguan Institute of Spine and Spinal Cord Injury, The Sixth Affiliated Hospital of Jinan University (Dongguan), Dongguan, China; 2 Department of Neurosurgery, The Affiliated Guangdong Second Provincial General Hospital of Jinan University, Guangzhou, China; 3 Department of Orthopedic Surgery, The First Affiliated Hospital of Jinan University, Guangzhou, China

**Keywords:** bioinformatics, ferroptosis, HMOX1, inflammatory microenvironment, macrophages/microglia, spinal cord injury

## Abstract

**Background:**

Spinal cord injury (SCI) causes severe and persistent neurological dysfunction. Ferroptosis has been implicated in multiple neurological disorders, but its contribution to SCI and its relationship to myeloid-cell responses, inflammatory amplification and disturbed iron homeostasis remain unclear.

**Methods:**

We integrated public bulk RNA-sequencing and single-cell RNA-sequencing datasets with experiments in a rat SCI model to define ferroptosis-associated changes across the molecular, cellular and tissue levels. Differential expression, pathway enrichment, co-expression and protein–protein interaction analyses, pseudotime inference and cell–cell communication modelling were used to identify candidate molecules and relevant myeloid subpopulations, followed by qPCR, western blotting and immunofluorescence validation.

**Results:**

Ferroptosis-associated molecular alterations in SCI showed marked temporal dynamics and remained embedded within pathological networks linked to inflammation, oxidative stress and hypoxic responses. Single-cell analysis indicated that these signals were concentrated primarily in myeloid cells, particularly the HMOX1-high M1a and M1b subclusters. Pseudotime and cell–cell communication analyses further suggested that these subpopulations progress along a continuous trajectory towards inflammation-amplifying states and may influence the local microenvironment through MIF, TGFβ, PTN and CD99 signalling. Animal experiments further showed that sustained inflammatory activation occurs in parallel with dysregulation of ferroptosis-associated molecules, accompanied by local myeloid-cell activation and enhanced HMOX1-associated stress responses.

**Conclusions:**

In SCI, ferroptosis-associated signals appear to be concentrated within HMOX1-associated myeloid subpopulations and may be sustained through cell-state reprogramming and intercellular signaling networks. HMOX1 emerges as a candidate hub linking disturbed iron handling, ferroptosis and myeloid inflammatory remodeling.

## 1 Introduction

The disabling consequences of spinal cord injury (SCI) are determined not only by the initial mechanical insult, but also by the secondary injury cascade that unfolds thereafter. Uncontrolled inflammation, oxidative stress and cell death jointly drive pathological progression during this phase, yet the interplay among these processes remains incompletely understood (1). Ferroptosis is a form of regulated cell death driven by iron-dependent lipid peroxidation and closely associated with iron accumulation, membrane lipid peroxidation and disruption of the GPX4/SLC7A11 axis (2). Previous studies have shown that activation of the Nrf2/GPX4 axis can attenuate SCI and improve functional recovery, supporting a role for ferroptosis in SCI pathogenesis (3). Transcriptomic analyses have likewise revealed marked ferroptosis-associated molecular abnormalities at different time points after SCI, closely linked to hypoxic and inflammatory signaling (4). However, current knowledge of ferroptosis in SCI remains largely confined to whole-tissue observations or individual molecules. Existing studies have therefore established primarily that ferroptosis occurs, while offering much less insight into how it is sustained and amplified within the injured microenvironment (3, 4).

During secondary injury after SCI, macrophages and microglia act as key effector cells in the inflammatory response. They contribute to lesion progression not only through regulation of local cytokine networks, phagocytic clearance and antigen presentation, but also through sustained interactions with neurons, astrocytes and oligodendrocytes, thereby reshaping the lesion microenvironment and influencing tissue repair (5–7). Importantly, the post-injury myeloid response does not conform to a simple M1/M2 binary; rather, it comprises multiple subpopulations that differ substantially in metabolic state, inflammatory intensity and communication capacity (8–10). Moreover, immune cells within the post-SCI microenvironment are highly heterogeneous, and microglial function is not fixed, but is continually reshaped by dynamic state reprogramming and ongoing crosstalk with surrounding cells.

We therefore hypothesized that ferroptosis-associated molecular abnormalities in SCI are not uniformly distributed, but preferentially concentrated in specific myeloid subpopulations that may be associated with the amplification of disturbed iron metabolism and oxidative stress into tissue-level inflammatory responses. To date, however, the relationships among ferroptosis, myeloid-cell heterogeneity, state transitions and intercellular communication have not been systematically defined. We therefore focused on a putative pathological framework linking ferroptosis, macrophage/microglial subpopulations and inflammatory amplification after SCI. By integrating bulk RNA-sequencing and single-cell RNA-sequencing analyses, we sought to delineate ferroptosis-associated molecular alterations across different stages of SCI, define myeloid-cell heterogeneity and clarify their interrelationships. Through this approach, we aimed to investigate how ferroptosis-associated molecular changes may relate to secondary injury through specific inflammatory myeloid subpopulations and to provide a framework for more precise therapeutic targeting.

## 2 Materials and methods

### 2.1 Establishment of the spinal cord injury model

A total of 60 adult female Wistar rats (8 weeks old; 230 ± 10 g) were purchased from Zhejiang Vital River Laboratory Animal Technology Co., Ltd. (Laboratory Animal Production License No. SCXK (Zhe) 2020-0002). The animals were randomly assigned to four groups: a sham-operated group (Sham, *n* = 15), an SCI 1-day group (SCI_1 d, *n* = 15), an SCI 3-day group (SCI_3 d, *n* = 15) and an SCI 7-day group (SCI_7 d, *n* = 15). All animal experiments were approved by the Institutional Animal Ethics Committee of Jinan University (Approval No. 20231124-03) and were conducted in accordance with institutional guidelines for the care and use of laboratory animals. ats were fasted before surgery but allowed free access to water. Anesthesia was induced with 2% sodium pentobarbital (50 mg/kg, intraperitoneally), and animals were placed prone on a thermostatically controlled heating pad to maintain body temperature. After routine shaving and disinfection, a midline dorsal incision was made at the T10 level, and the paraspinal muscles were separated to expose the vertebral lamina. The T10 spinous process and lamina were removed to expose the spinal cord. In the SCI groups, a contusive injury was delivered to the dorsal aspect of the T10 spinal cord using the Louisville Injury System Apparatus (LISA; Louisville, USA) with the following parameters: velocity, 1.0 m/s; displacement, 1.2 mm; dwell time, 1.0 s. In the Sham group, the spinal cord was exposed without impact. Successful model establishment was preliminarily confirmed by obvious local congestion or hemorrhage at the injury site immediately after impact, transient twitching of the hind limbs and tail, and marked postoperative impairment of bilateral hind limb motor function (11, 12).

After surgery, gentamicin was administered subcutaneously at 10 mg/kg for 7 consecutive days. Manual bladder expression was performed twice daily until spontaneous voiding returned. All animals were housed under controlled environmental conditions (22–24 °C; 12-h light/dark cycle) with free access to food and water. At 1, 3 and 7 days after surgery, rats were anaesthetized with 2% sodium pentobarbital by intraperitoneal injection, followed by transcardial perfusion. Spinal cord tissue from the injured segment was then collected for subsequent analyses.

### 2.2 Data sources and processing

This study included the GSE45006, GSE151371 and GSE213240 datasets from the Gene Expression Omnibus (GEO) database (13). From GSE45006, 4 control samples and 12 spinal cord injury samples collected at 1, 3 and 7 days after injury (4 samples per time point) were selected for transcriptome-wide differential expression analysis. From GSE151371, samples from 10 healthy controls and 38 patients with SCI were included for external validation of candidate genes (14). From GSE213240, single-cell RNA-sequencing data from 2 Sham, 2 Moderate and 2 Severe samples were selected for analyses of cellular heterogeneity and cellular localization of candidate genes (15).

Single-cell transcriptomic analyses were performed using the Seurat package (16). Genes expressed in at least 5 cells were retained, and only cells with at least 300 detected genes were included for subsequent analyses. Quality control was then performed by excluding cells with fewer than 500 or more than 6, 000 detected genes, as well as cells with a mitochondrial transcript proportion greater than 10%. After quality filtering, the data were normalised, and the top 4, 000 highly variable genes were selected for downstream dimensionality reduction. Batch effects were corrected using the Harmony package, and cell clustering was performed in Seurat with a clustering resolution of 0.4 (17). Cell type annotation was conducted using the SingleR package with the MouseRNAseqData database as the reference (18, 19). Marker genes for each cell cluster were identified using the FindAllMarkers function, with an adjusted P value < 0.05 and |log2FC| ≥ 1 as the selection criteria.

### 2.3 Identification of ferroptosis-related genes

Differential expression analysis was performed using the limma package, with an adjusted *P* value < 0.05 and |log2FC| ≥ 1 set as the screening criteria. Ferroptosis-related genes were obtained from the FerrDb database and intersected with the differentially expressed genes identified at each time point to define ferroptosis-related differentially expressed genes (FDEGs) after SCI (20). Temporal clustering analysis was then performed on the overall FDEG set to identify major dynamic expression patterns after SCI. The resulting clusters were visualized as heatmaps based on per-gene Z-score-normalized expression values arranged in chronological order. Row clustering was applied to group genes with similar temporal expression profiles, whereas column clustering was disabled to preserve the temporal order of the experimental groups.

### 2.4 Functional enrichment analysis

GO and KEGG enrichment analyses were performed separately for the FDEGs identified at days 1, 3 and 7 after SCI (21–23). In parallel, gene set enrichment analysis (GSEA) was conducted on ranked whole-transcriptome expression profiles to evaluate the dynamic enrichment of pathways across different time points. An adjusted *P* < 0.05 was considered statistically significant. GSEA results were presented as normalized enrichment scores (NES) and visualized using ridge plots (21, 24).

### 2.5 Weighted gene co-expression network analysis and identification of key genes

Weighted gene co-expression network analysis (WGCNA) was used to construct gene co-expression networks (25, 26). Briefly, a gene expression correlation matrix was first generated, and an appropriate soft-thresholding power (β) was selected to construct an approximately scale-free network. This was then transformed into a topological overlap matrix (TOM), and co-expression modules were identified using dynamic tree cutting. Module eigengenes were subsequently correlated with sample groups, and modules significantly associated with the SCI process (*P* < 0.05, |cor| > 0.6) were retained for further analysis. Candidate key modules were then determined by integrating ferroptosis-related genes, and genes with module membership (MM) > 0.8 and gene significance (GS) > 0.6 were defined as hub genes.

### 2.6 Protein–protein interaction network analysis

FDEGs from each time point were uploaded to the STRING database to construct protein–protein interaction (PPI) networks, with the minimum interaction score threshold set at 0.4 (27). The resulting networks were imported into Cytoscape for visualization, and the top three highest-scoring modules were selected for further investigation (28, 29). Hub genes were then identified using the cytoHubba plugin based on the maximal clique centrality (MCC) algorithm, and the top 10 ranked genes were retained for subsequent analyses (30).

### 2.7 Construction of the upstream regulatory network

Transcription factors associated with the core genes were predicted using the miRNet platform, and a TF–mRNA regulatory network was constructed (31). Potential miRNAs targeting the core genes were predicted using the miRWalk 2.0 database and further cross-validated with miRDB and miRNet. The identified TF–mRNA and miRNA–mRNA interactions were imported into Cytoscape for network visualization, and key regulatory factors were preliminarily screened on the basis of network topological features.

### 2.8 Construction of the ceRNA regulatory network

After obtaining core gene-associated miRNAs, key miRNAs targeting two or more core genes were selected. These candidate miRNAs were then submitted to miRBase for sequence annotation and conservation analysis, and their potential target lncRNAs were predicted using the starBase database (32, 33). On the basis of the resulting lncRNA–miRNA and miRNA–mRNA interaction pairs, an lncRNA–miRNA–mRNA competing endogenous RNA (ceRNA) regulatory network was constructed to identify potentially important non-coding RNA regulators (34).

### 2.9 Immune cell infiltration analysis

The GSVA package was used to perform single-sample gene set enrichment analysis (ssGSEA) to evaluate immune cell infiltration across samples (35). Immune-related gene signatures were obtained from published literature and comprised 28 immune cell subsets or immune functional states (36). ssGSEA generated an enrichment score matrix for each sample across these 28 immune cell or immune functional categories. Between-group comparisons were performed using the Wilcoxon rank-sum test, and multiple testing correction was conducted using the Benjamini–Hochberg method. An FDR < 0.05 was considered statistically significant.

### 2.10 Pseudotime trajectory analysis of microglial/macrophage subpopulations

Pseudotime analysis was performed using Monocle. Seurat objects were converted into CellDataSet objects, and ordering genes were selected for trajectory construction. Dimensionality reduction was then used to reconstruct cell-state trajectories, and a pseudotime value was assigned to each cell (37). Based on the distribution of different subpopulations along the trajectory and the expression dynamics of key genes over pseudotime, the potential evolutionary relationships and functional state transitions of these subpopulations after spinal cord injury were evaluated.

### 2.11 Cell–cell communication analysis

Cell–cell communication analysis was performed on the single-cell transcriptomic data using the CellChat package (38). Based on the subpopulation annotations, microglia/macrophages were reclassified into the M0, M1a, M1b and M2–M5 subgroups, and CellChatDB.mouse was used as the ligand–receptor interaction database to infer potential communication networks among these subpopulations. The number, frequency and overall strength of intercellular interactions were then assessed to identify major signaling pathways as well as potential signal senders and receivers. Circular plots and bubble plots were used to visualize the intercellular communication network and key signaling pathways.

### 2.12 Quantitative real-time polymerase chain reaction

Spinal cord tissue from the T10 segment was collected at 1, 3 and 7 days after SCI, as well as after sham surgery, and rapidly stored at −80 °C. Total RNA was extracted using TRIzol reagent (Invitrogen, Thermo Fisher Scientific, USA). Genomic DNA removal and reverse transcription were performed according to the manufacturer’s instructions for the PrimeScript™ RT reagent Kit with gDNA Eraser (Perfect Real Time) (Takara, Japan; Code No. RR047A) to generate cDNA. Quantitative real-time PCR was subsequently carried out using TB Green^®^ Premix Ex Taq™ II (Tli RNaseH Plus) (Takara, Japan; Code No. RR820A) on a CFX96 Real-Time PCR Detection System (Bio-Rad, USA). The amplification protocol consisted of an initial denaturation at 95 °C for 30 s, followed by 40 cycles of 95 °C for 5 s and 60 °C for 20 s, and a melting-curve analysis was performed at the end of amplification. GAPDH was used as the internal reference gene, and relative expression levels of *IL1α, IL1β, IL10, TNFα, IL18, CD86, CD206, TLR4, HMOX1, GPX4, FTH1* and *FTL* were calculated using the 2^−ΔΔCt method. Primer sequences are listed in **Supplementary Table 1**.

### 2.13 Western blotting

Spinal cord tissue (~0.5 cm) centered on the lesion site was collected and lysed in RIPA buffer containing protease inhibitors (P0013C, Beyotime), followed by incubation on ice for 30 min. Lysates were then centrifuged at 12, 000 × g for 20 min at 4 °C, and the supernatants were collected. Total protein concentration was determined using a BCA Protein Assay Kit (P0012, Beyotime). Equal amounts of protein were mixed with 5× protein loading buffer (Fd002, Fdbio science) and denatured at 100 °C for 10 min. Samples were then separated by SDS–PAGE using precast Gels Plus Bis-Tris gels (SLE019, Smart-lifesciences) at 90 V until the protein marker (26616-1, Thermo Fisher) reached the bottom of the gel. Proteins were subsequently transferred onto PVDF membranes (BS-PVDF-45, Merck Millipore) using a wet-transfer system at a constant current of 300 mA for 1.5 h.

After transfer, membranes were blocked with 5% bovine serum albumin (V900933, Sigma-Aldrich) for 2 h at room temperature and then incubated overnight at 4 °C with the following primary antibodies: iNOS (18985-1-AP, Proteintech, 1:1, 000), Arg-1 (16001-1-AP, Proteintech, 1:5, 000), TLR4 (ab22048, Abcam, 1:800), CD206 (ab64693, Abcam, 1:1, 000), p53 (ab26, Abcam, 1:1, 000), GPX4 (ab125066, Abcam, 1:1, 000), NRF2 (16396-1-AP, Proteintech, 1:2, 000), HMOX1 (10701-1-AP, Proteintech, 1:10, 000), SLC7A11 (26864-1-AP, Proteintech, 1:1, 000), FTH1 (ab183781, Abcam, 1:1, 000), FTL (10727-1-AP, Proteintech, 1:2, 000), β-actin (MA5-11869, Invitrogen, 1:1, 000) and Vinculin (700062, Invitrogen, 1:1, 000). Membranes were washed three times with TBST the following day and incubated with the appropriate HRP-conjugated secondary antibodies (goat anti-rabbit IgG or goat anti-mouse IgG, R&D Systems, USA, 1:1, 000). After additional washing, protein bands were visualized using an enhanced chemiluminescence substrate (36208ES60, Yeasen), and images were acquired using a chemiluminescence imaging system. Band intensities were quantified using ImageJ software, and target protein expression was normalized to the corresponding internal control, β-actin or Vinculin.

### 2.14 Immunofluorescence staining

Spinal cord tissue from the lesion center was fixed overnight in 4% paraformaldehyde, dehydrated sequentially in 15% and 30% sucrose solutions, embedded in OCT compound after sinking, and sectioned into 20-μm-thick frozen sections. After washing in PBS, sections were permeabilized with 0.1% Triton X-100 at room temperature and blocked for 2 h at room temperature in blocking solution containing 10% donkey serum and 3% bovine serum albumin. Sections were then incubated overnight at 4 °C with the following primary antibodies: anti-Iba1 (ab178846, Abcam, 1:500), anti-iNOS (18985-1-AP, Proteintech, 1:250) and anti-HMOX1 (10701-1-AP, Proteintech, 1:400). On the following day, sections were washed with PBS and incubated for 2 h at room temperature in the dark with Alexa Fluor 488-conjugated donkey anti-rabbit secondary antibody (A32790TR, Invitrogen, 1: 1, 000). Nuclei were then counterstained with DAPI (D1306, Thermo Fisher Scientific, 1: 1, 000) for 10 min at room temperature in the dark. Finally, sections were mounted with an anti-fade mounting medium, images were acquired using a fluorescence microscope (Axio Imager Z2, Zeiss), and image analysis was performed using ImageJ software.

### 2.15 Statistical analysis

Experimental data were analyzed using GraphPad Prism 9.0 and are presented as mean ± SEM. Comparisons between two groups were performed using the unpaired Student’s t-test. Comparisons among multiple groups were conducted using one-way analysis of variance (ANOVA), followed by Tukey’s *post hoc* test for pairwise comparisons. When the assumptions of normality or homogeneity of variance were not met, non-parametric tests were applied. A *P* value < 0.05 was considered statistically significant.

## 3 Results

### 3.1 Identification and dynamic expression patterns of ferroptosis-related differentially expressed genes after SCI

**Figure 1:**
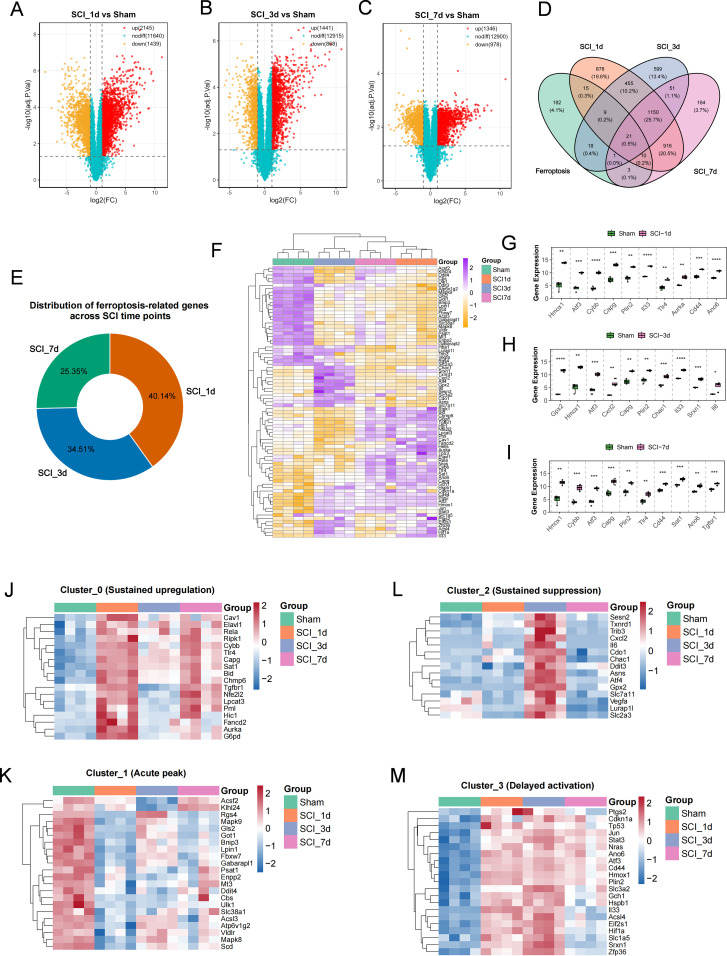
Identification and dynamic expression patterns of ferroptosis-related differentially expressed genes after spinal cord injury. **(A–C)** Volcano plots showing the distribution of differentially expressed genes (DEGs) between the Sham group and SCI at different time points, including SCI_1d **(A)**, SCI_3d **(B)**, and SCI_7d **(C)**. The x-axis represents log_2_(Fold Change), and the y-axis represents –log_10_(P value). Red dots indicate significantly upregulated genes, green dots indicate significantly downregulated genes, and black dots indicate non-significant genes (FDR < 0.05 and |log_2_FC| > 1). **(D)** Venn diagram showing the overlap between DEGs at different time points after SCI and ferroptosis-related genes obtained from FerrDb. The overlapping genes were defined as ferroptosis-related differentially expressed genes (FDEGs). **(E)** Pie chart showing the proportions of FDEGs identified at 1, 3, and 7 days after SCI, indicating that ferroptosis-related responses were more active at the early stage after injury. **(F)** Heatmap showing the overall expression patterns of FDEGs across the Sham, SCI_1d, SCI_3d, and SCI_7d groups. Colors represent normalized expression levels. Both genes and samples were hierarchically clustered, revealing distinct group-specific expression patterns across different time points. **(G–I)** Expression distributions of representative FDEGs at 1 day **(G)**, 3 days **(H)**, and 7 days **(I)** after SCI. Several genes, including Hmox1, Atf3, Capg, Plin2, and Tlr4, were repeatedly dysregulated across multiple time points, suggesting persistent regulatory roles in ferroptosis after SCI. **(J–M)** Temporal clustering analysis of FDEGs. Panels **(J, K, L)**, and M correspond to Cluster_0, Cluster_1, Cluster_2, and Cluster_3, respectively. The overall FDEG set was grouped into four temporal expression patterns, including sustained upregulation, early peaking, sustained suppression, and delayed activation. Expression values were normalized on a per-gene basis using Z-scores and displayed in chronological order. Rows represent genes and columns represent samples. Row clustering was applied to display genes with similar temporal expression profiles, whereas column clustering was disabled to preserve the temporal sequence of the experimental groups.

Differential expression analysis showed that, compared with the Sham group, 3, 584, 2, 309 and 2, 324 differentially expressed genes (DEGs) were identified at 1, 3 and 7 days after SCI, respectively. The largest number of DEGs was observed at day 1, indicating that the most extensive transcriptional remodeling occurred during the early phase of injury (**Figures 1A–C**). Intersecting the DEGs from each time point with the FerrDb ferroptosis-related gene set yielded 57, 49 and 36 ferroptosis-related differentially expressed genes (FDEGs), respectively (**Figures 1D, E**; **Supplementary Table 2**). The overall heat map clearly distinguished the Sham samples from those collected at different post-SCI time points, indicating a systematic shift in the ferroptosis-related transcriptional landscape after injury (**Figure 1F**). Among these genes, Hmox1, Atf3, Cybb, Capg, Plin2, Cd44 and Tlr4 showed relatively high expression across multiple time points, whereas Gpx2, Cxcl2, Chac1, Srxn1 and Il6 exhibited stage-dependent fluctuations (**Figures 1G–I**). Further temporal clustering analysis classified the FDEGs into four expression patterns: persistently upregulated, early-peaking, persistently suppressed and delayed-activation profiles. These temporal patterns were visualized as heatmaps using per-gene Z-score-normalized expression values arranged in chronological order. Among them, Hmox1, Tp53, Ptgs2, Stat3 and Tgfbr1 mainly exhibited delayed activation, whereas Cybb, Capg and Rela showed sustained upregulation, suggesting that the ferroptosis-related molecular network undergoes marked temporal remodeling after SCI. (**Figures 1J–M**; **Supplementary Table 3**).

### 3.2 FDEGs after SCI are enriched in coupled oxidative stress–inflammation–ferroptosis pathways

**Figure 2:**
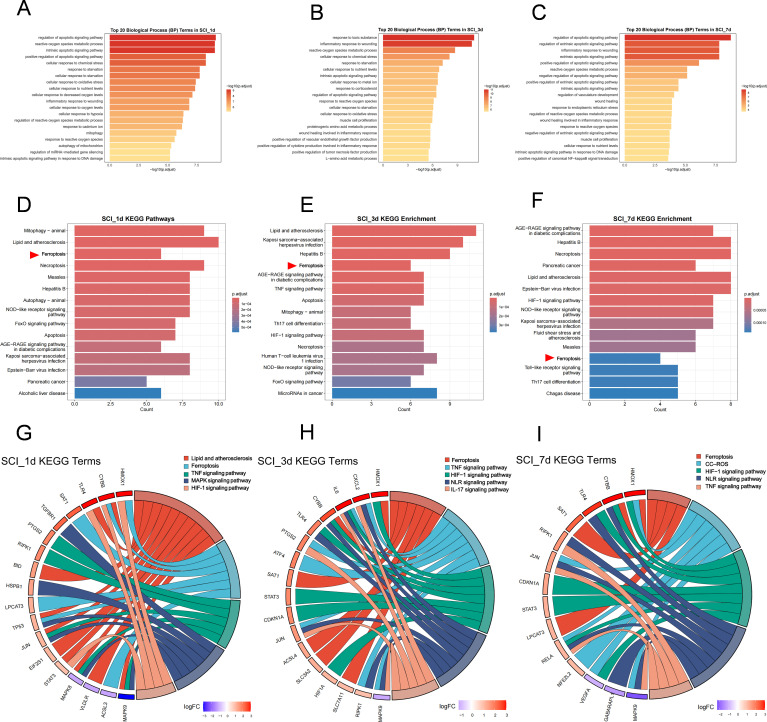
GO and KEGG enrichment analyses of FDEGs at different time points after SCI. **(A–C)** Top 20 enriched GO biological process (BP) terms of FDEGs at 1 day **(A)**, 3 days **(B)**, and 7 days **(C)** after SCI. The x-axis represents −log10(adjusted P value), and color intensity indicates enrichment significance. **(D–F)** Top 15 enriched KEGG pathways of FDEGs at 1 day **(D)**, 3 days **(E)**, and 7 days **(F)** after SCI. The x-axis indicates gene count, and color represents adjusted P value. Red arrows indicate the ferroptosis pathway. **(G–I)** Chord diagrams showing the relationships between representative enriched KEGG pathways and their associated genes at 1 day **(G)**, 3 days **(H)**, and 7 days **(I)** after SCI. Different colors represent different pathways, and the colors of outer gene labels indicate logFC values.

GO analysis showed that FDEGs after SCI were consistently enriched at different time points in biological processes related to reactive oxygen species metabolism, oxidative and chemical stress responses, regulation of apoptotic signaling and injury-associated inflammatory responses. At specific stages, they were also involved in hypoxia responses, mitophagy and endoplasmic reticulum stress (**Figures 2A–C**; **Supplementary Table 4**). Consistent with this, KEGG analysis showed persistent enrichment of the ferroptosis pathway at all post-SCI time points, accompanied by dynamic activation of inflammation- and stress-related pathways, including Toll-like receptor, NOD-like receptor, TNF, HIF-1 and IL-17 signaling (**Figures 2D–F**). Chord plots further revealed that genes such as Hmox1, Cybb, Tlr4, Tp53, Rela and Ripk1 repeatedly appeared across multiple enriched pathways (**Figures 2G–I**; **Supplementary Table 5**), indicating that ferroptosis-related changes after SCI are closely associated with sustained oxidative stress– and inflammation-related signaling pathways. Notably, these enrichment patterns were also consistent with the temporal clustering results, which showed that ferroptosis-related genes followed distinct trajectories rather than a uniform monotonic trend. Whereas some genes remained continuously dysregulated, others showed an early increase followed by later re-elevation, indicating that oxidative stress-, inflammatory-, hypoxia-, and ferroptosis-related programs were represented at multiple stages of SCI progression rather than being confined to a single acute phase.

### 3.3 WGCNA identifies stage-associated modules and core ferroptosis hub genes across the course of SCI

**Figure 3:**
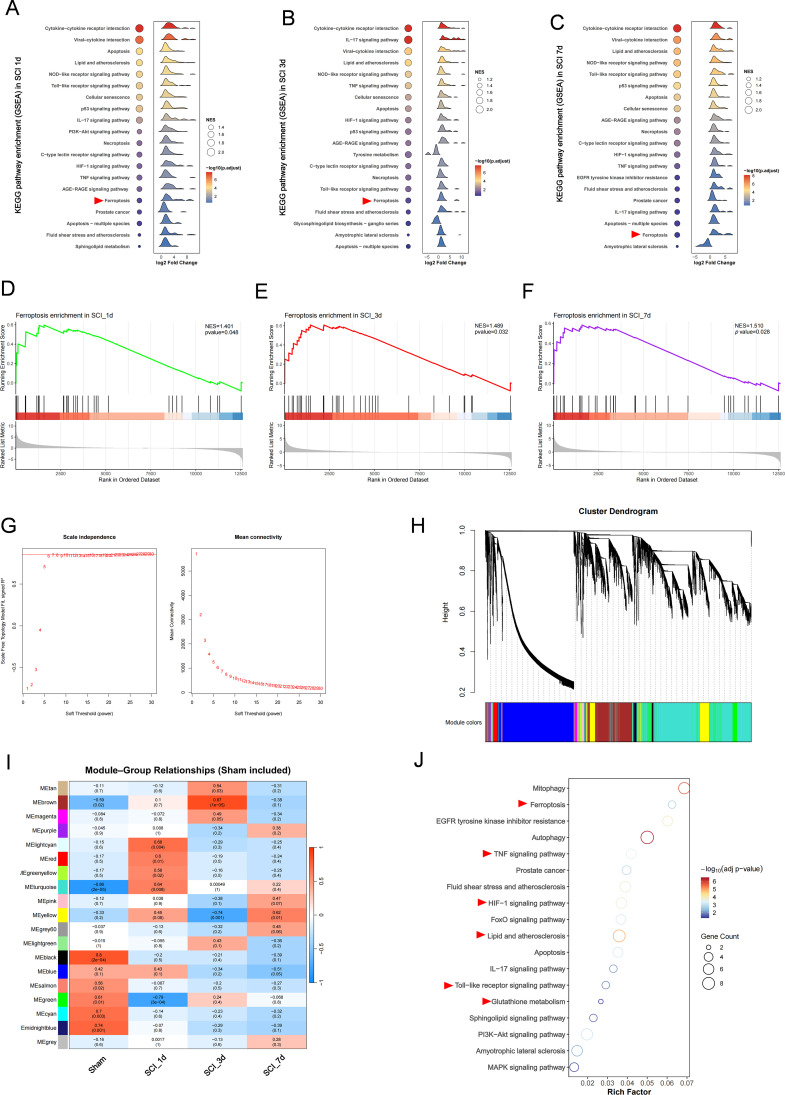
WGCNA identifies stage-associated modules and core ferroptosis hub genes after SCI. **(A–F)** GSEA ridge plots showing significantly enriched KEGG pathways at different time points after SCI. Panels A and B represent upregulated and downregulated pathways in SCI_1d vs Sham, panels C and D represent upregulated and downregulated pathways in SCI_3d vs Sham, and panels E and F represent upregulated and downregulated pathways in SCI_7d vs Sham. Different colors indicate different pathways, and the x-axis represents normalized enrichment scores (NES). **(G)** Soft-thresholding analysis for WGCNA. The left panel shows the scale-free topology fit index across different soft-thresholding powers, and the right panel shows the corresponding mean connectivity, based on which the soft-thresholding power was selected for network construction. **(H)** Gene dendrogram and module assignment generated by WGCNA. Different colors indicate distinct co-expression modules. **(I)** Heatmap of module–trait relationships showing the correlations between module eigengenes and experimental groups, including Sham, SCI_1d, SCI_3d, and SCI_7d. Values in each cell indicate correlation coefficients and corresponding P values. **(J)** KEGG enrichment bubble plot of stage-specific ferroptosis hub genes. The x-axis indicates GeneRatio, bubble size represents gene count, and color indicates adjusted P value.

GSEA based on ranked whole-transcriptome expression profiles showed pronounced time-dependent remodeling of KEGG pathway activity after SCI and was broadly consistent with the over-representation analyses of FDEGs. Inflammation- and stress-related pathways, including cytokine–cytokine receptor interaction, Toll-like receptor, NOD-like receptor, TNF and HIF-1 signaling, remained enriched across different time points, while the ferroptosis pathway showed consistent positive enrichment at days 1, 3 and 7 (**Figures 3A–F**). Similar pathway-level changes were therefore observed across ranked whole-transcriptome profiles as well as within the FDEG subset. Following construction of the co-expression network based on the selected soft-thresholding power, stage-specific co-expression modules associated with different phases of injury were identified (**Figures 3G, H**; **Supplementary Tables 6** and **S7**). Specifically, MElightcyan, MEturquoise and MEred were positively correlated with SCI_1d, whereas MEgreen was negatively correlated with SCI_1d. MEbrown showed the strongest positive correlation with SCI_3d, while MEyellow was negatively associated with SCI_3d but became positively correlated at SCI_7d (**Figure 3I**), indicating marked stage-dependent restructuring of the co-expression network after SCI. Further intersection of stage-associated modules with the FerrDb gene set identified phase-specific ferroptosis hub-gene programmes (**Supplementary Table 8**): SCI_1d was centered on MAPK8/PIK3CA/SQSTM1; SCI_3d was characterized by the ATF4–CHAC1–HIF1A/KEAP1 axis; and SCI_7d was represented by NFE2L2, MAP1LC3A, VEGFA and SLC2A3. These hub genes were mainly enriched in pathways related to mitophagy, ferroptosis, autophagy, TNF and HIF-1 signaling (**Figure 3J**), indicating that ferroptosis-associated hub-gene programmers are not static after SCI, but instead show stage-dependent transitions as injury progresses.

### 3.4 PPI network analysis reveals stage-specific reorganisation of ferroptosis-associated hub-gene networks after SCI

**Figure 4:**
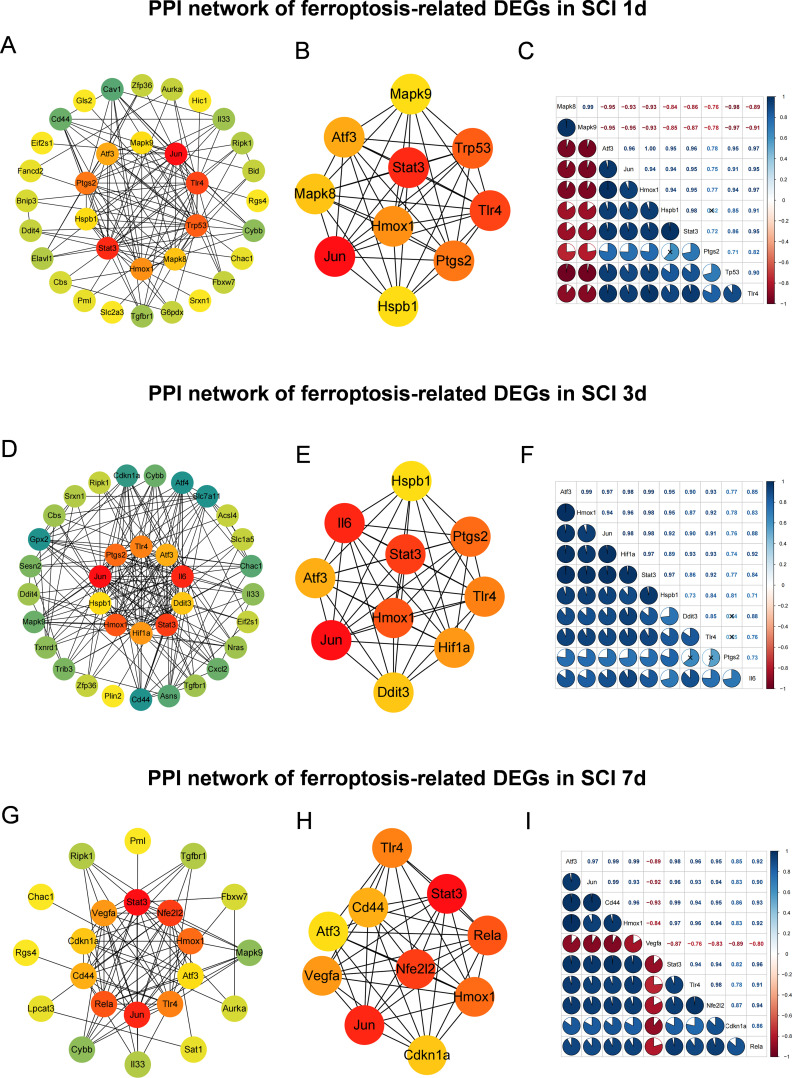
PPI network analysis reveals stage-dependent rewiring of ferroptosis hub-gene networks after SCI. **(A, D, G)** Protein–protein interaction (PPI) networks generated from ferroptosis-related differentially expressed genes (FDEGs) at 1, 3, and 7 days after SCI, respectively. Node color intensity indicates the maximal clique centrality (MCC) score. **(B, E, H)** Top 10 hub-gene subnetworks extracted from each stage-specific PPI network based on MCC ranking. **(C, F, I)** Pairwise correlation heatmaps of the top 10 hub genes at the corresponding time points. Blue denotes positive correlation and red denotes negative correlation, with color intensity reflecting correlation strength.

PPI network analysis showed that the ferroptosis-associated network underwent marked stage-specific reorganization as SCI progressed (**Supplementary Table 9**). At SCI_1d, the network was characterized by the Mapk8/Mapk9–Atf3/Jun–Hmox1/Tlr4 axis, consistent with prominent stress initiation and inflammatory activation during the acute phase (**Figures 4A, B**). At SCI_3d, the network shifted towards an Atf3–Hmox1–Hif1a/Il6–Stat3 axis, consistent with closer coordination between hypoxic adaptation and inflammatory–stress programmes (**Figures 4D, E**). By SCI_7d, the network had evolved into a more complex configuration centered on Atf3, Cd44, Hmox1, Stat3, Tlr4, Nfe2l2 and Rela, together with branched Vegfa/Cdkn1a features, consistent with increasing association with persistent inflammation, antioxidant adaptation and tissue remodeling at the later stage (**Figures 4G, H**). Pairwise correlation heatmaps further refined the stage-dependent rewiring of ferroptosis-associated hub-gene networks (**Figures 4C, F, I**; **Supplementary Table 10**). At SCI_1d, the correlation structure showed a clear modular split: Mapk8 and Mapk9 formed a tightly correlated pair, but were broadly negatively correlated with most of the remaining hub genes. In contrast, Atf3, Jun, Hmox1, Hspb1, Stat3, Ptgs2, Tp53 and Tlr4 were positively correlated with one another overall, indicating an early separation between a stress-kinase branch and a coordinated inflammatory/stress-response module. At SCI_3d, this early separation was largely lost, and the network became much more integrated, with broadly positive correlations across most hub genes. Particularly strong positive coupling was observed among Atf3, Hmox1, Jun, Hif1a and Stat3, whereas Ddit3, Tlr4 and Il6 were also incorporated into the same positively coordinated structure; Ptgs2 remained positively associated with this network, although with relatively weaker coupling than the central nodes. By SCI_7d, a broad late-stage positively correlated module had emerged among Atf3, Jun, Cd44, Hmox1, Stat3, Tlr4, Nfe2l2 and Rela, whereas Vegfa showed broad negative correlations with nearly all of these core genes, indicating that it became relatively distinct from the main late-stage core module. Cdkn1a remained positively linked to the network, but with comparatively weaker correlations than the main core module. Together, these correlation patterns indicate that ferroptosis-associated hub-gene networks after SCI do not remain in a single stable configuration, but instead shift from early module separation, through subacute network integration, to a more heterogeneous late-stage correlation structure.

### 3.5 Construction of upstream regulatory networks and external validation of key hub genes

**Figure 5:**
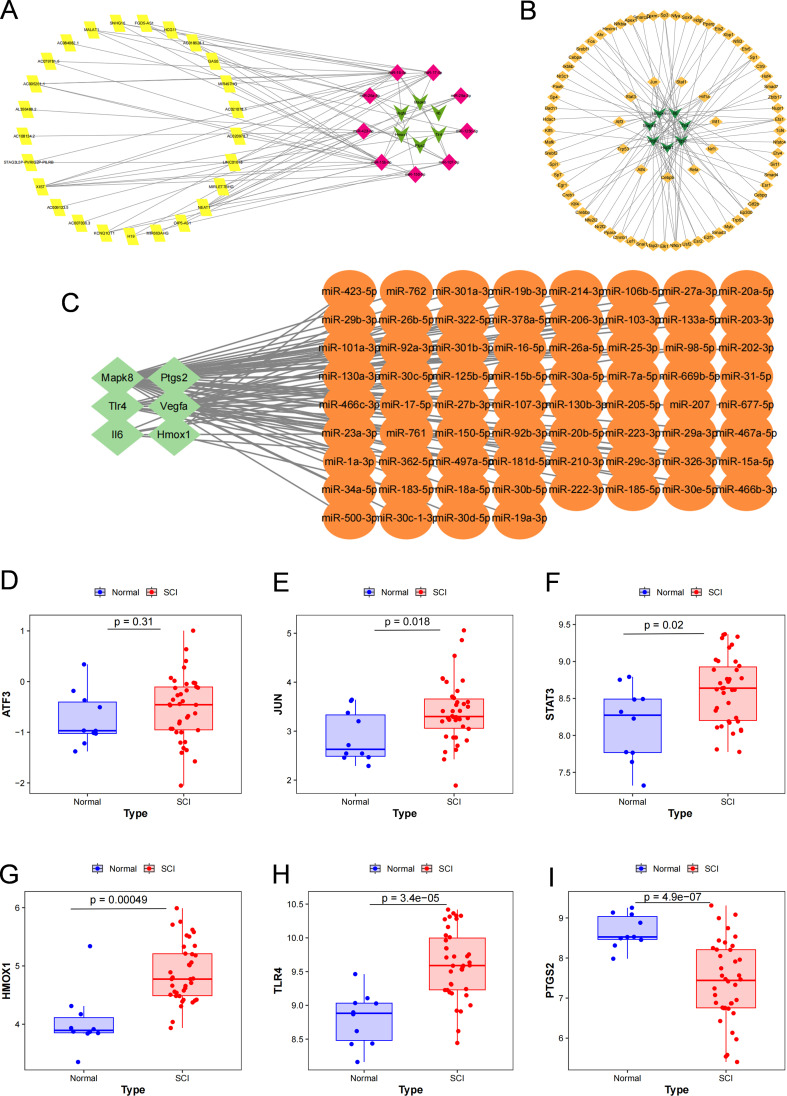
Upstream regulatory networks and external validation of representative hub genes. **(A)** lncRNA–miRNA–mRNA ceRNA network constructed for key hub genes. Yellow squares represent lncRNAs, magenta diamonds represent miRNAs, and green triangles represent mRNAs. **(B)** Transcription factor (TF)–mRNA regulatory network of key hub genes. Orange circles represent TFs and green triangles represent target mRNAs. **(C)** miRNA–mRNA regulatory network of key hub genes. Orange circles represent miRNAs and green diamonds represent target mRNAs. **(D–I)** External validation of representative hub genes in the GSE151371 dataset. Boxplots show the expression levels of ATF3 **(D)**, JUN **(E)**, STAT3 **(F)**, HMOX1 **(G)**, TLR4 **(H)**, and PTGS2 **(I)** in healthy controls and SCI samples. P values are indicated in each panel.

The ceRNA network comprised 25 lncRNAs, 9 miRNAs and 6 mRNAs, forming a regulatory framework centered on Mapk8, Vegfa, Tlr4, Ptgs2, Il6 and Hmox1, with miR-16-5p and miR-15b-5p showing the highest connectivity (**Figure 5A**; **Supplementary Table 11**). The TF–mRNA network identified 75 transcription factors regulating 7 hub genes. Among these, Ptgs2, Vegfa, Il6 and Hmox1 exhibited relatively high regulatory connectivity, whereas Nfkb1, Ets1, Jun and Hif1a constituted the principal upstream transcriptional regulatory nodes (**Figure 5B**; **Supplementary Table 12**). Among the highly connected target genes, Hmox1 attracted particular attention because it was connected to multiple major upstream transcription factor nodes in the TF–mRNA network. In particular, Jun and Hif1a emerged as notable regulators associated with Hmox1, suggesting that Hmox1 was positioned at the intersection of oxidative stress-related and hypoxia-related transcriptional regulation. The miRNA–mRNA network further showed that 68 miRNAs targeted 6 hub genes, with Mapk8 displaying the highest miRNA connectivity, followed by Vegfa and Tlr4 (**Figure 5C**; **Supplementary Table 13**). Validation in an independent cohort showed that, compared with healthy controls, JUN, STAT3, HMOX1 and TLR4 were significantly upregulated in SCI samples, whereas PTGS2 was significantly downregulated; ATF3 did not show a statistically significant difference (**Figures 5D–I**; **Supplementary Table 14**). These findings suggest that ferroptosis-associated hub genes after SCI are not aberrantly expressed in isolation, but are embedded within multilayered regulatory networks, among which JUN, STAT3, HMOX1 and TLR4 show relatively stable cross-cohort consistency.

### 3.6 Immune remodeling after SCI and its association with HMOX1/TLR4-related myeloid inflammatory signatures

**Figure 6:**
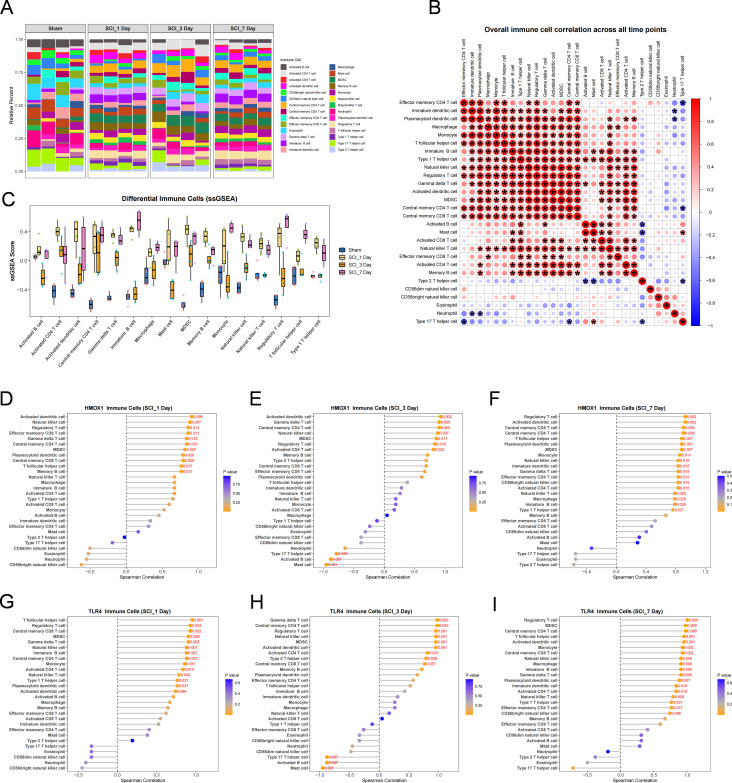
Immune infiltration remodeling after SCI and its association with the HMOX1/TLR4-centered myeloid inflammatory axis. **(A)** Heatmap showing ssGSEA scores of 28 immune cell populations across the Sham, SCI_1d, SCI_3d, and SCI_7d groups. **(B)** Correlation heatmap of immune cell infiltration across all samples. Colors indicate pairwise correlation coefficients among immune cell populations. **(C)** Boxplots showing the relative infiltration scores of representative immune cell populations across the four groups. **(D–F)** Correlation heatmaps showing the associations between HMOX1 expression and immune cell infiltration at 1 day **(D)**, 3 days **(E)**, and 7 days **(F)** after SCI, respectively. **(G–I)** Correlation heatmaps showing the associations between TLR4 expression and immune cell infiltration at 1 day **(G)**, 3 days **(H)**, and 7 days **(I)** after SCI, respectively. Colors represent correlation strength and direction.

ssGSEA revealed pronounced time-dependent remodeling of the immune microenvironment after SCI. The enrichment of inflammation-related immune cell signatures was already markedly increased during the early phase after injury, reached a more coordinated state at day 3, and showed partial reorganization and stratification by day 7 (**Figures 6A–C**; **Supplementary Table 14**). Specifically, activated dendritic cells, myeloid-derived suppressor cells (MDSCs), natural killer cells, γδ T cells and multiple T-cell subsets remained elevated after injury, whereas macrophage-related signatures showed an early increase, partial maintenance at day 3 and a decline by day 7 (**Figures 6A, C**). Immune-cell correlation analysis further showed that the immune network in the Sham group was relatively dispersed, whereas after SCI—particularly at day 3—antigen-presenting cells, MDSCs and NK/T-cell subsets formed a more tightly coordinated network (**Figure 6B**). Notably, HMOX1 and TLR4 showed stable but stage-dependent correlations with this immune remodeling process (**Figures 6D–I**; **Supplementary Table 15**). Both genes were positively correlated with multiple inflammation-related immune signatures as early as day 1, with these associations becoming stronger at day 3; by day 7, they remained strongly positively correlated with activated dendritic cells, MDSCs, regulatory T cells, central memory CD4 T cells and monocytes. Among these relationships, the correlations of both genes with macrophage-related signatures were most prominent at day 7, particularly the strong positive association between TLR4 and macrophage-related signatures (**Figures 6H, I**). Collectively, these results suggest that immune remodeling after SCI is closely associated with HMOX1- and TLR4-related myeloid inflammatory signatures, particularly within macrophage- and dendritic-cell-associated modules.

### 3.7 Single-cell transcriptomics shows enrichment of ferroptosis-associated signals in inflammatory myeloid subpopulations after SCI

**Figure 7:**
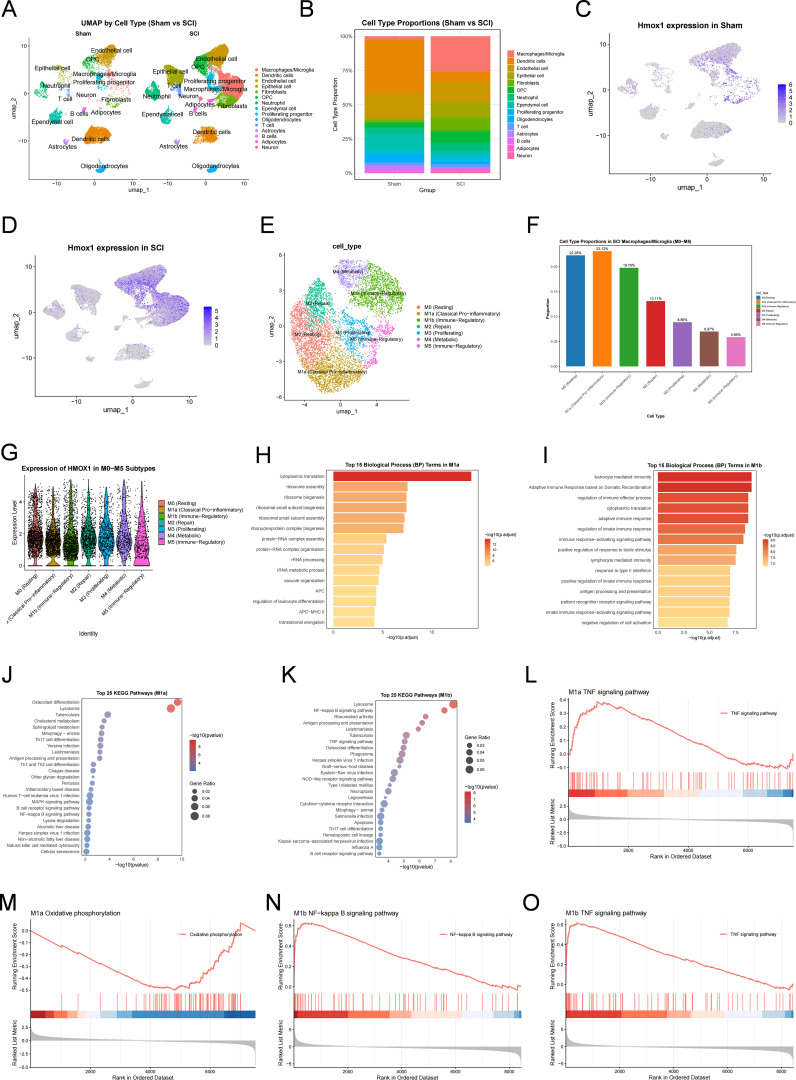
Single-cell transcriptomics identifies HMOX1-associated inflammatory myeloid subpopulations after SCI. **(A)** UMAP plot showing the major cell populations identified in the spinal cord single-cell RNA-sequencing dataset. **(B)** UMAP plot showing the distribution of cells across Sham, Moderate, and Severe SCI groups. **(C)** Feature plots showing Hmox1 expression across all cell populations. **(D)** Dot plot showing the relative expression and proportion of Hmox1-expressing cells across major cell types. **(E)** UMAP plot of reclustered myeloid cells, identifying seven subpopulations (M0, M1a, M1b, M2, M3, M4, and M5). **(F)** Bar plot showing the relative proportions of the seven myeloid subpopulations. **(G)** Violin plot showing Hmox1 expression across myeloid subpopulations. **(H, I)** GO enrichment analysis of marker genes from M1a **(H)** and M1b **(I)**. **(J, K)** KEGG enrichment analysis of marker genes from M1a **(J)** and M1b **(K)**. **(L, M)** GSEA plots showing significantly enriched KEGG pathways in M1a relative to M0. **(N, O)** GSEA plots showing significantly enriched KEGG pathways in M1b relative to M0.

Single-cell transcriptomic analysis showed marked remodeling of spinal cord cellular composition after SCI, with a pronounced expansion of macrophages/microglia populations, which represented one of the principal cellular sources of Hmox1 expression (**Figures 7A–D**). Compared with the Sham group, both the distribution and intensity of Hmox1 expression were markedly increased in the SCI group, indicating that ferroptosis-associated signals after injury were not uniformly distributed across all cell types, but were enriched predominantly within the myeloid compartment (**Figures 7C, D**). Further reclustering of myeloid cells from SCI samples identified seven subpopulations—M0, M1a, M1b, M2, M3, M4 and M5—among which M1a and M1b were the dominant injury-associated subgroups (**Figures 7E, F**; **Supplementary Table 16**). Notably, HMOX1 remained highly expressed in both dominant subpopulations (**Figure 7G**), suggesting the ferroptosis-associated myeloid signals SCI were distributed across more than one inflammatory myeloid state rather than being confined to a single subpopulation.

Among these, M1a was characterized by genes such as Apoc1, Siglec1, Apoe and Htr2b, consistent with a macrophage-like state biased towards inflammatory activation. Its functional profile was enriched mainly in lysosome-, mitophagy- and ribosome-related processes (**Figures 7H, J**; **Supplementary Table 17**), indicating substantial organelle remodeling and metabolic adaptation alongside pro-inflammatory activation. By contrast, M1b retained microglial markers such as P2ry12, Tmem119 and Gpr34, but was strongly enriched in TNF signaling, NF-kappa B signaling, NOD-like receptor signaling, and antigen processing and presentation pathways (**Figures 7I, K**; **Supplementary Table 18**), indicating a more activated state that preserves microglial features while showing a stronger association with inflammatory signal integration. GSEA further showed that, relative to M0, M1a was enriched in the TNF signaling pathway and accompanied by downregulation of oxidative phosphorylation, whereas M1b displayed even stronger enrichment of TNF and NF-kappa B signaling (**Figures 7L–O**; **Supplementary Table 18**). Together, these findings indicate that ferroptosis-associated signals after SCI are concentrated primarily within two HMOX1-high inflammatory myeloid subpopulations that show distinct inflammatory and metabolic features.

### 3.8 Pseudotime and cell–cell communication analyses suggest dynamic progression and communication remodeling in HMOX1-associated myeloid subpopulations

**Figure 8:**
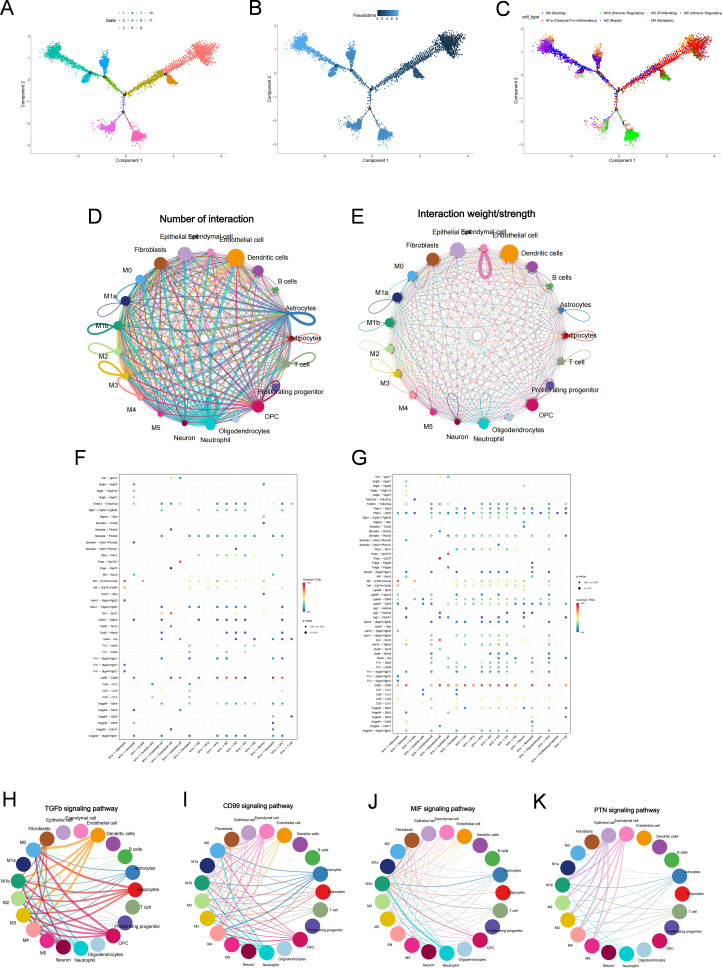
Pseudotime and cell–cell communication analyses reveal progressive activation of HMOX1-associated myeloid subpopulations after SCI. **(A)** Pseudotime trajectory of myeloid cells reconstructed by Monocle. **(B)** Distribution of myeloid subpopulations along the pseudotime trajectory. **(C)** Pseudotime trajectory colored by myeloid subpopulation identity, showing the transition from M0 toward the injury-associated M1a and M1b branches. **(D)** Global cell–cell communication network inferred by CellChat across major cell populations in the SCI spinal cord microenvironment. **(E)** Quantification of the number and strength of inferred interactions among major cell populations. **(F, G)** Outgoing communication patterns of the two dominant injury-associated myeloid subpopulations, M1a **(F)** and M1b **(G)**. **(H, I)** Bubble plots showing representative ligand–receptor signaling pathways enriched in M1a **(H)** and M1b **(I)**, respectively. **(J, K)** Differential signaling profiles of injury-associated myeloid subpopulations, highlighting the prominent involvement of MIF/TGFβ signaling in M1a and MIF/CD99/PTN signaling in M1b.

Pseudotime analysis suggested that myeloid cells underwent continuous state transitions after SCI, with M0 cells localized mainly to the initial region of the trajectory, whereas the two injury-associated dominant subpopulations, M1a and M1b, occupied distinct terminal branches. This pattern was consistent with a transition from a relatively homeostatic-like state towards more differentiated injury-associated inflammatory states(**Figures 8A–C**). Together with the preceding findings, persistent HMOX1-high expression along this trajectory suggests that ferroptosis-associated signals may be related to macrophage/microglial state transitions CellChat analysis further showed that this trajectory was accompanied by marked remodeling of intercellular communication networks (**Figures 8D, E**). Both M1a and M1b exhibited a high number of interactions and strong communication intensity, indicating that they were prominent communication-active subpopulations within the post-injury myeloid compartment. Pathway analysis indicated that M1a was more prominently associated with the TGFβ and MIF signaling axes, whereas M1b showed greater involvement in CD99 and PTN signaling (**Figures 8F–K**), suggesting differences in their communication preferences and potential functional orientation. Notably, MIF signaling occupied a prominent position in both injury-associated myeloid subpopulations (**Figure 8J**), indicating that HMOX1-associated macrophage/microglial states may be linked to inflammatory communication within the injured microenvironment.

### 3.9 Experimental validation supports parallel inflammatory activation and ferroptosis-related molecular changes after SCI

**Figure 9:**
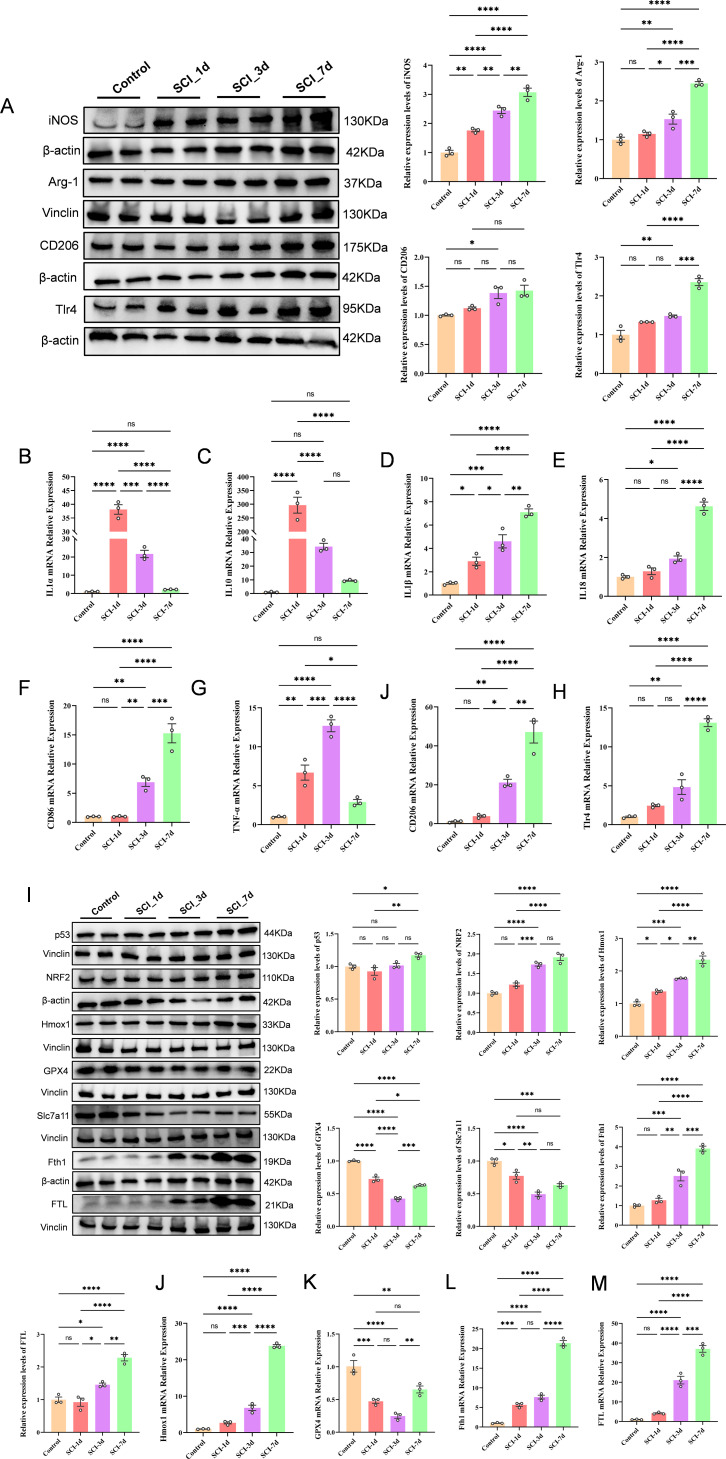
Experimental validation confirms coordinated inflammatory activation and ferroptosis-related remodeling after SCI. **(A)** Representative Western blot images and densitometric quantification of inflammation-related proteins, including iNOS, Arg-1, TLR4, and CD206, in spinal cord tissues from the Sham, SCI_1d, SCI_3d, and SCI_7d groups. **(B–H)** qPCR analysis of inflammation-related genes, including Il1a **(B)**, Il1b **(C)**, Il10 **(D)**, Tnfa **(E)**, Il18 **(F)**, Cd86 **(G)**, and Cd206 **(H)**, across the four groups. **(I)** Representative Western blot images and densitometric quantification of ferroptosis-related proteins, including p53, GPX4, NRF2, HMOX1, SLC7A11, FTH1, and FTL, in spinal cord tissues from the Sham, SCI_1d, SCI_3d, and SCI_7d groups. **(J–M)** qPCR analysis of ferroptosis-related genes, including Tlr4 **(J)**, Hmox1 **(K)**, Gpx4 **(L)**, and Fth1/Ftl **(M)**, across the four groups. Data are presented as mean ± SEM. Statistical significance was determined by one-way ANOVA followed by Tukey’s multiple-comparison test. *P < 0.05, **P < 0.01, ***P < 0.001, ****P < 0.0001, ns > 0.05.

qPCR and western blot analyses consistently showed that SCI induced sustained inflammatory activation in spinal cord tissue, accompanied by concurrent changes inferroptosis-related molecules (**Figures 9A–M**). Within the inflammatory module, protein levels of iNOS and TLR4 were markedly increased, while Arg-1 also showed an overall upward trend. Consistently, qPCR demonstrated significant upregulation of Il1a, Il1b, Il18, Tnfa, Cd86, Cd206 and Tlr4. Among these, Il1a was strongly induced as early as SCI_1d (approximately 38-fold), whereas Tlr4 showed a more pronounced increase at SCI_7d (approximately 13-fold), consistent with sustained local inflammatory activation and altered macrophage-associated activation signatures (**Figures 9A–H, J**). Within the ferroptosis module, p53, NRF2, HMOX1, FTH1 and FTL were generally increased, whereas GPX4 and SLC7A11 were markedly reduced (**Figures 9I–M**). Notably, Hmox1 was elevated by approximately 24-fold at SCI_7d, whereas Gpx4 declined to its lowest level at SCI_3d (approximately 0.25-fold) and recovered only partially thereafter. These findings are consistent with altered iron-handling–related molecular profiles occurring in parallel with suppression of the GPX4/SLC7A11 antioxidant defense axis after SCI.

### 3.10 Immunofluorescence validation shows progressive myeloid activation and sustained HMOX1-associated stress signals after SCI

**Figure 10:**
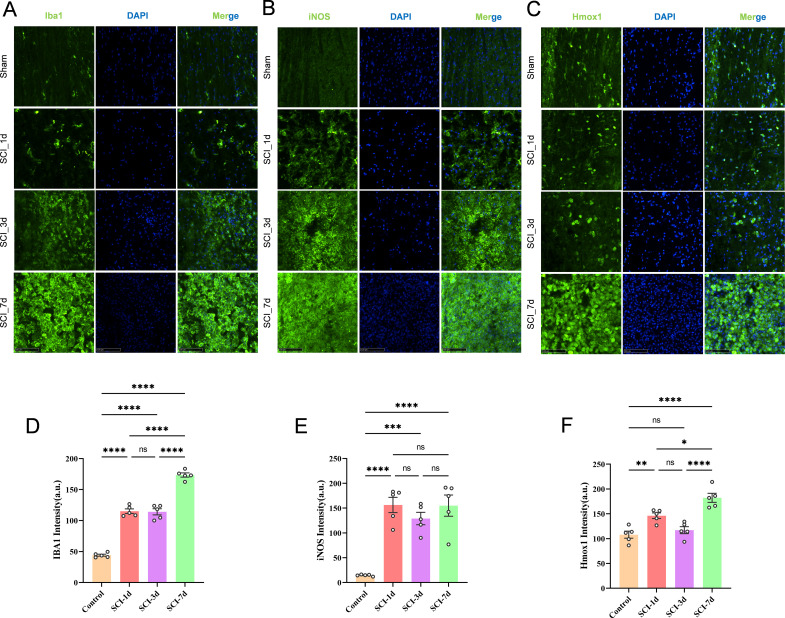
Immunofluorescence validation reveals persistent myeloid activation and progressive HMOX1-associated stress after SCI. **(A–C)** Representative immunofluorescence images showing IBA1 **(A)**, iNOS **(B)**, and Hmox1 **(C)** staining in spinal cord tissues from the Sham, SCI_1d, SCI_3d, and SCI_7d groups. **(D–F)** Quantification of fluorescence intensity for IBA1 **(D)**, iNOS **(E)**, and Hmox1 **(F)** across the four groups. IBA1-positive cells increased markedly after SCI and exhibited a transition from ramified morphology in the Sham group to hypertrophic and clustered activated morphologies after injury. iNOS was rapidly induced after SCI and remained elevated throughout the observation period, whereas Hmox1 showed a more progressive increase and reached the highest level at SCI_7d. Data are presented as mean ± SEM. Statistical significance was determined by one-way ANOVA followed by Tukey’s multiple-comparison test. *P < 0.05, **P < 0.01, ***P < 0.001, ****P < 0.0001, ns > 0.05. Scale bars, as indicated in the images.

Immunofluorescence staining showed a marked increase in IBA1 signal within the injured region after SCI, accompanied by a morphological shift from a ramified appearance to hypertrophic and cluster-like activated forms (**Figures 10A, D**). Quantitative analysis revealed that IBA1 fluorescence intensity was already significantly increased at SCI_1d, remained elevated at SCI_3d, and was further enhanced at SCI_7d, consistent with progressive local myeloid-cell activation after injury. At the same time, iNOS increased rapidly after SCI and remained highly expressed from SCI_1d onward (**Figures 10B, E**), indicating that the pro-inflammatory activation was initiated early after injury and persisted thereafter. By contrast, Hmox1 displayed a more prominent late-stage enhancement pattern: although it was already increased at SCI_1d compared with the control group, it reached its highest level at SCI_7d, with further expansion of the staining area and the density of positive cells (**Figures 10C, F**). Together, these findings indicate temporally distinct but overlapping changes in myeloid activation, pro-inflammatory signaling and HMOX1-associated stress markers within the injured region after SCI, and are consistent with parallel involvement of inflammatory activation and ferroptosis-related molecular stress during secondary injury progression.

## 4 Discussion

### 4.1 Ferroptosis and inflammatory amplification occur in parallel after SCI

Ferroptosis after SCI is not simply reflected by dysregulation of a few local genes or a single pathway; rather, it arises within a broader pathological reprogramming characterized by inflammation, oxidative stress and cell death (2, 3). Differential expression analysis showed that ferroptosis-associated genes were most profoundly perturbed as early as day 1 after injury and remained imbalanced at days 3 and 7, suggesting that ferroptosis-related molecular stress is rapidly engaged and remains detectable throughout secondary injury progression. Functional enrichment analysis likewise demonstrated sustained enrichment of the ferroptosis pathway, together with canonical inflammatory and stress-associated pathways, including TNF, Toll-like receptor, NOD-like receptor and HIF-1 signaling. Together, these findings suggest that ferroptosis-associated changes in SCI are better viewed within a broader pathological context involving inflammation, oxidative stress and metabolic imbalance, rather than as an isolated cell-death-related phenomenon (39, 40). Viewed together, the enrichment results and the temporal clustering patterns suggest that the ferroptosis-associated response after SCI is not a single-wave event. The persistent enrichment of oxidative stress-, inflammation-, hypoxia- and ferroptosis-related pathways across multiple stages is consistent with the observation that not all ferroptosis-related genes follow a simple monotonic trajectory. Instead, some genes appear to be induced early after injury and then become re-elevated at later stages, which may reflect an initial acute response triggered by primary trauma and hemorrhage-associated oxidative stress, followed by a later reinforcement phase driven by persistent innate immune activation, disturbed iron handling, hypoxic adaptation and tissue remodeling during secondary injury.

The sustained-upregulation cluster (Cluster_0), which includes Cybb, Rela, Tlr4, Ripk1, Capg and Tgfbr1, is most consistent with a persistent inflammatory redox module rather than a transient injury signal. In particular, CYBB/NOX2 is a major source of phagocyte-derived reactive oxygen species after SCI, and TLR4-dependent innate immune activation is closely linked to hemorrhage-associated microglia/macrophage responses in the injured cord; together, these genes suggest a prolonged ROS–inflammation circuit that may amplify secondary damage after SCI (41, 42). The early-peaking cluster (Cluster_1), enriched in Bnip3, Ulk1, Gabarapl1, Gabarapl2, Ddit4, Acsl3 and Scd, is more compatible with an acute adaptive response involving autophagy/mitophagy and membrane-lipid remodeling. Such a pattern may reflect an early attempt to buffer mitochondrial stress and reshape lipid composition immediately after injury, because BNIP3/NIX-mediated mitophagy can reduce mitochondrial ROS and protect against ferroptosis, whereas ACSL3-dependent MUFA activation promotes a ferroptosis-resistant state (43, 44). By contrast, the sustained-suppression cluster (Cluster_2) includes several canonical stress-response or antioxidant-related genes, such as Atf4, Ddit3, Chac1, Slc7a11, Txnrd1 and Gpx2. Because many of these genes are normally associated with ferroptosis-linked amino-acid stress and redox adaptation, their sustained suppression may indicate progressive failure or exhaustion of compensatory defense mechanisms, thereby lowering resistance to lipid peroxidation as injury evolves (45). Finally, the delayed-activation cluster (Cluster_3), containing Acsl4, Atf3, Hif1a, Hmox1, Ptgs2, Stat3, Tp53, Gch1, Hspb1 and Plin2, appears to define a later remodeling program in which heme/iron handling, ferroptotic susceptibility and counter-regulatory survival responses coexist. This interpretation is particularly plausible because ACSL4 is a determinant of ferroptosis sensitivity, ATF3 can promote ferroptosis by repressing SLC7A11, GCH1-BH4 and HSPB1 can oppose ferroptotic lipid damage, and HMOX1 has been identified as a ferroptosis hub gene in SCI [ (46–49). Taken together, these cluster-resolved patterns suggest that ferroptosis-associated transcription after SCI is temporally ordered, progressing from early adaptive buffering to persistent inflammatory amplification and later heme/iron-driven remodeling, rather than unfolding as a single uniform program.

Consistent with this interpretation, qPCR and western blot analyses showed persistent upregulation of iron metabolism- and heme-processing-related molecules, including HMOX1, FTH1 and FTL, together with suppression of the GPX4/SLC7A11 antioxidant defense axis after SCI. This pattern is consistent with enhanced iron handling together with weakened protection against lipid peroxidation. In particular, the sustained increase in HMOX1 alongside the marked reduction in GPX4 suggests that the post-injury microenvironment is not simply mounting a generalized oxidative-stress response, but may be associated with conditions more favorable to ferroptosis-related molecular stress. Although NRF2 also trended upwards, implying some degree of compensatory antioxidant activity, this endogenous response did not appear sufficient to fully counterbalance the increased lipid peroxidation-related stress associated with suppression of the GPX4/SLC7A11 axis. The injured spinal cord therefore appears to enter a state in which compensatory defenses are engaged, yet redox homeostasis remains incompletely restored.

### 4.2 Myeloid-cell heterogeneity provides a cellular context for interpreting ferroptosis-associated changes after SCI

Secondary injury after SCI has long been linked to activated macrophages and microglia, yet the traditional binary M1/M2 framework is insufficient to capture the complexity of post-injury myeloid states in terms of metabolic programming, inflammatory capacity and intercellular communication (50–52). The myeloid response after SCI is therefore not a simple switch in polarization, but a composite response involving multiple subpopulations with distinct transcriptional and functional features (8–10). Moreover, ferroptosis-associated responses are not uniformly distributed across the myeloid compartment, but were enriched to specific injury-associated subpopulations. In the present study, M1a and M1b emerged as the two dominant post-injury states, and both showed high HMOX1 expression. This pattern suggests that ferroptosis-associated signals in SCI do not represent a diffuse, but instead concentrate within defined inflammatory myeloid states.

These two subpopulations are nonetheless appeared to differ in their dominant biological characteristics. M1a appears to be more closely associated with phagocytic–lysosomal remodeling, lipid metabolic adaptation and organelle-level reprogramming, whereas M1b is more strongly linked to inflammatory signal integration and immune amplification, with enrichment of TNF, NF-κB, NOD-like receptor and antigen-presentation-related programmes (53, 54). Myeloid reprogramming after SCI is therefore not a unidirectional increase in pro-inflammatory activity, but diverges into at least two dominant pathological states with distinct molecular profiles: one biased towards more closely related to, and the other towards sustained integration and propagation of inflammatory signaling. Persistent HMOX1 expression in both subpopulations further suggests that ferroptosis-associated responses are not restricted to a single myeloid subset, but may instead represent a common molecular feature shared by multiple injury-associated inflammatory myeloid states.

### 4.3 HMOX1 is associated with ferroptosis-related stress and myeloid inflammatory remodeling after SCI

As a canonical stress-response molecule, HMOX1 participates in antioxidant and cytoprotective processes through the heme oxygenase pathway (55, 56). Under conditions of disturbed iron handling after SCI, however, its role is likely to be context-dependent. On the one hand, HMOX1 upregulation may reflect a compensatory response to oxidative and inflammatory stress. On the other hand, the free iron released during heme degradation may further promote lipid peroxidation, thereby increasing susceptibility to ferroptosis (57, 58). In light of the sustained upregulation of FTH1 and FTL, together with suppression of the GPX4/SLC7A11 axis observed here, HMOX1 appears to be closely associated with a pathological setting characterized by disturbed heme metabolism, altered iron handling and persistent oxidative stress. In addition, HMOX1 was not uniformly distributed across cell types, but was enriched in injury-associated myeloid subpopulations. Immune infiltration analysis further showed significant associations between HMOX1 and myeloid as well as antigen-presenting cell related signatures, supporting its close association with inflammatory myeloid responses. Taken together, these findings suggest that HMOX1 may mark not only enhanced local oxidative stress, but also a subset of injury-associated myeloid states linked to persistent inflammatory activation after SCI. From the perspective of transcriptional control, our network suggests that JUN and HIF1A are the transcription factors most prominently linked to HMOX1 in SCI. Importantly, this finding should not be interpreted as implying that HMOX1 is driven by a single upstream pathway. Rather, previous mechanistic studies indicate that HMOX1 is governed by a layered stress-responsive regulatory architecture. At the core of this architecture is the NRF2–BACH1 switch: NRF2 acts as a potent transcriptional activator of HMOX1, whereas BACH1 functions as a dominant repressor at Maf/ARE-like enhancer elements and must be displaced or inactivated before robust HMOX1 induction can occur (59–61). Superimposed on this redox-sensitive module is a hypoxia-responsive input mediated by HIF-1, which directly activates HMOX1 transcription under low-oxygen conditions (62). In parallel, AP-1 family regulation introduces an additional layer of context dependence: Jun proteins associate with the HMOX1 regulatory region, but their effects are not uniformly activating, as JunB promotes whereas JunD restrains HMOX1 expression, indicating that AP-1 composition may shape both the amplitude and direction of HMOX1 induction (63). In this context, the prominence of JUN and HIF1A in our SCI network likely does not mean that they replace the canonical NRF2–BACH1 module; rather, it suggests that, within the injured spinal cord, hypoxic and inflammatory stress signals may represent the context-dominant inputs through which the broader HMOX1 regulatory program is engaged. This interpretation is also consistent with our stage-resolved analyses, in which HIF1A-related signals were more evident during the subacute phase, whereas NFE2L2 entered the late-stage ferroptosis-associated hub program, supporting a temporally layered model of HMOX1 regulation after SCI. Taken together, these findings position HMOX1 as a candidate hub linking ferroptosis and myeloid inflammatory reprogramming after SCI, rather than as a simple marker of tissue injury.

### 4.4 Pseudotime progression and cell–cell communication analyses suggest dynamic remodeling of HMOX1-associated myeloid states after SCI

Pseudotime analysis suggested that, after SCI, myeloid cells transition from a relatively homeostatic M0-like state along a continuous trajectory towards the two principal injury-associated subpopulations, M1a and M1b. This pattern was consistent with progressive transcriptional reorganization of myeloid states within the injured microenvironment, rather than simple persistence of a pre-existing inflammatory population (53, 64). Along this trajectory, HMOX1 showed a sustained upward trend. Together with its coordinated variation with ferroptosis-associated molecules and inflammatory phenotypes, this pattern suggests that ferroptosis-associated signals may be related to the progression of injury-associated myeloid state transitions, rather than being restricted to a single terminal stage (40, 54).

CellChat analysis further suggested that this state progression was accompanied by substantial remodeling of intercellular communication patterns. Both the M1a and M1b subpopulations displayed high communication activity, indicating that they were communication-active components of the post-injury myeloid compartment. More specifically, M1a was more strongly associated with MIF- and TGFβ-related signaling axes, whereas M1b was more prominently enriched for CD99, PTN and selected inflammatory ligand–receptor networks, suggesting differences in their communication preferences and potential functional orientation Of particular note, MIF signaling occupied a prominent position in injury-associated myeloid subpopulations, indicating that it may represent a candidate pathway associated with the coordination of oxidative stress- and inflammation-related signaling in the injured microenvironment. Persistent pathology after SCI may therefore depend not only on whether ferroptosis-related stress is present, but also on how injury-associated myeloid states are embedded within local communication networks (10, 40, 54, 65). From this perspective, the pathological relevance of ferroptosis in SCI may involve not only cell-intrinsic stress responses, but also its association with broader inflammatory microenvironment remodeling through specific myeloid subpopulations.

### 4.5 Limitations and future perspectives

Several limitations of this study should be acknowledged. First, although multiple public datasets were integrated and complemented by animal-based validation, the overall evidence remains largely correlative. In particular, direct gain-of-function or loss-of-function experiments for key nodes such as HMOX1, TLR4 and MIF were not performed in the present study. Therefore, our findings are better interpreted as identifying candidate molecules, candidate cell states and plausible pathway-level associations, rather than establishing definitive causal mechanisms. Second, the single-cell RNA-sequencing data and the bulk RNA time-series data were not fully derived from the same biological source. Accordingly, the present findings are better suited to revealing cell-state changes and pathway-level trends than to replacing strictly time-resolved tissue transcriptomic analyses. Third, owing to the limited resolution of the available datasets and the complexity of marker-gene expression, the boundary between infiltrating peripheral macrophages and resident microglia could not be delineated with absolute clarity.

Despite these limitations, our data support the view that ferroptosis-associated changes in SCI are not uniformly distributed, but are enriched in injury-associated myeloid subpopulations. In particular, HMOX1-associated myeloid states may represent candidate pathological correlates of disturbed iron handling, inflammatory remodeling and altered microenvironmental communication after SCI. However, whether HMOX1 directly drives these processes will require future validation using cell-type-specific overexpression, knockdown or knockout approaches.

In addition, future therapeutic strategies for SCI may therefore need to move beyond generic antioxidant or broad anti-inflammatory approaches towards more structured intervention frameworks centered on the ferroptosis–myeloid reprogramming axis. In particular, our findings raise the possibility that HMOX1-oriented intervention strategies may warrant consideration, although such approaches are unlikely to be biologically unidirectional. Trehalose, for example, has been reported to inhibit ferroptosis and promote functional recovery after SCI through activation of the NRF2/HO-1 pathway (66). In addition, stabilization of HMOX1 itself has also been reported to suppress ferroptosis and ameliorate SCI (67). Metformin has likewise been shown to alleviate SCI by inhibiting neuronal ferroptosis in a manner partly dependent on HMOX1 upregulation (68). On the other hand, the role of HMOX1/HO-1 in ferroptosis is likely to be context-dependent, because HO-1-mediated heme degradation can also increase labile intracellular iron availability and thereby, under some conditions, facilitate ferroptotic injury (69). From this perspective, a more realistic translational strategy may not be indiscriminate HMOX1 activation, but rather stage-specific and cell-type-specific modulation of HO-1/HMOX1, potentially combined with therapies that directly constrain iron-dependent lipid peroxidation.

## 5 Conclusions

Ferroptosis-associated signals are not uniformly distributed across cell types, but are enriched in HMOX1-high, injury-associated myeloid cells, particularly the M1a and M1b subpopulations. These dynamically reprogrammed states may be associated with inflammatory communication and microenvironmental remodeling through MIF, TGFβ, PTN and CD99 signaling. Collectively, our findings identify HMOX1-associated myeloid responses as candidate pathological correlates of disturbed iron handling and secondary injury after SCI.

## Data Availability

The datasets presented in this study can be found in online repositories. The names of the repository/repositories and accession number(s) can be found in the article/**Supplementary Material**.
