# Supplementary material for: Ferroptosis-associated myeloid cell heterogeneity and inflammatory amplification following spinal cord injury
Source: Front Immunol. 2026 Apr 22;17:1831161. doi: 10.3389/fimmu.2026.1831161 (PMC13143767; doi:10.3389/fimmu.2026.1831161)
Supplement: Supplementary file 1 [file DataSheet1.zip › Supplementary Table S1.docx]

**Supplementary Table 1**

*Primer sequences used for RT-qPCR*

| **Gene** | **Forward primer (5′→3′)** | **Reverse primer (5′→3′)** |
| --- | --- | --- |
| Hmox1 | CAAGCACAGGGTGACAGAAGAGG | TCTGTGAGGGACTCTGGTCTTTGTG |
| Tlr4 | CCCTGCCACCATTTACAGTTCG | GAGTCCCAGCCAGATGCAAGAG |
| IL-1α | GGGATGATGACGACCTGCTAG | ACCACTTGTTGGCTTATGTTCTG |
| IL-10 | CACTCCCTGCATTACAATC | CAATGATGGTATTATAGGATCCC |
| IL-1β | AGTCACTCGCATGGCATGTG | ATATGTCGGGCTGGTTCCAC |
| TNF-α | AGCATGATCCGAGATGTGGAA | TAGACAGAAGAGCGTGGTGGC |
| CD86 | ACAGCAAAAGACACCCACGG | CTTGTTTCATTCTGAGCCTCCTC |
| CD206 | TGTTTTGGCTGGGACTGACCTA | CGGGTGTAGGCTCGGGTAGTAG |
| IL-18 | GGAATCAGACCACTTTGGCA | TGTCAACGAAGAGAACTTGGT |
| Fth1 | AGATGGGAGCCCCTGAATCT | CTCTCATCACCGTGTCCCAG |
| Ftl | CACCTACCTCTCTCTGGGCT | CCTCCAGGGTTTTACCCCAC |
| GPX4 | TGAACCTGGACGCCAAAGTC | GGGTTGAAAGGCTCGGGAAT |
| GAPDH | TTCCTACCCCCAATGTATCCG | CATGAGGTCCACCACCCTGTT |

*Abbreviation: RT-qPCR, real-time quantitative polymerase chain reaction.*
