# Supplementary material for: Ferroptosis-associated myeloid cell heterogeneity and inflammatory amplification following spinal cord injury
Source: Front Immunol. 2026 Apr 22;17:1831161. doi: 10.3389/fimmu.2026.1831161 (PMC13143767; doi:10.3389/fimmu.2026.1831161)
Supplement: Supplementary file 1 [file DataSheet1.zip › Supplementary Table S10.docx]

| Supplementary Table S10. Correlation coefficients and P values of stage-specific hub genes after SCI | | | | | | | | | | |
| --- | --- | --- | --- | --- | --- | --- | --- | --- | --- | --- |
| **SCI_1d corr** | **Mapk8** | **Mapk9** | **Atf3** | **Jun** | **Hmox1** | **Hspb1** | **Stat3** | **Ptgs2** | **Tp53** | **Tlr4** |
| **Mapk8** | 1.000 | 0.990 | -0.950 | -0.929 | -0.933 | -0.844 | -0.857 | -0.764 | -0.979 | -0.890 |
| **Mapk9** | 0.990 | 1.000 | -0.952 | -0.948 | -0.934 | -0.854 | -0.866 | -0.780 | -0.965 | -0.912 |
| **Atf3** | -0.950 | -0.952 | 1.000 | 0.960 | 0.997 | 0.948 | 0.963 | 0.777 | 0.948 | 0.973 |
| **Jun** | -0.929 | -0.948 | 0.960 | 1.000 | 0.936 | 0.937 | 0.955 | 0.753 | 0.905 | 0.946 |
| **Hmox1** | -0.933 | -0.934 | 0.997 | 0.936 | 1.000 | 0.941 | 0.955 | 0.771 | 0.938 | 0.972 |
| **Hspb1** | -0.844 | -0.854 | 0.948 | 0.937 | 0.941 | 1.000 | 0.984 | 0.619 | 0.849 | 0.911 |
| **Stat3** | -0.857 | -0.866 | 0.963 | 0.955 | 0.955 | 0.984 | 1.000 | 0.720 | 0.861 | 0.950 |
| **Ptgs2** | -0.764 | -0.780 | 0.777 | 0.753 | 0.771 | 0.619 | 0.720 | 1.000 | 0.707 | 0.819 |
| **Tp53** | -0.979 | -0.965 | 0.948 | 0.905 | 0.938 | 0.849 | 0.861 | 0.707 | 1.000 | 0.900 |
| **Tlr4** | -0.890 | -0.912 | 0.973 | 0.946 | 0.972 | 0.911 | 0.950 | 0.819 | 0.900 | 1.000 |
| **SCI_1d p value** | **Mapk8** | **Mapk9** | **Atf3** | **Jun** | **Hmox1** | **Hspb1** | **Stat3** | **Ptgs2** | **Tp53** | **Tlr4** |
| **Mapk8** |  | 0.000 | 0.000 | 0.001 | 0.001 | 0.008 | 0.006 | 0.027 | 0.000 | 0.003 |
| **Mapk9** | 0.000 |  | 0.000 | 0.000 | 0.001 | 0.007 | 0.005 | 0.022 | 0.000 | 0.002 |
| **Atf3** | 0.000 | 0.000 |  | 0.000 | 0.000 | 0.000 | 0.000 | 0.023 | 0.000 | 0.000 |
| **Jun** | 0.001 | 0.000 | 0.000 |  | 0.001 | 0.001 | 0.000 | 0.031 | 0.002 | 0.000 |
| **Hmox1** | 0.001 | 0.001 | 0.000 | 0.001 |  | 0.000 | 0.000 | 0.025 | 0.001 | 0.000 |
| **Hspb1** | 0.008 | 0.007 | 0.000 | 0.001 | 0.000 |  | 0.000 | 0.101 | 0.008 | 0.002 |
| **Stat3** | 0.006 | 0.005 | 0.000 | 0.000 | 0.000 | 0.000 |  | 0.044 | 0.006 | 0.000 |
| **Ptgs2** | 0.027 | 0.022 | 0.023 | 0.031 | 0.025 | 0.101 | 0.044 |  | 0.050 | 0.013 |
| **Tp53** | 0.000 | 0.000 | 0.000 | 0.002 | 0.001 | 0.008 | 0.006 | 0.050 |  | 0.002 |
| **Tlr4** | 0.003 | 0.002 | 0.000 | 0.000 | 0.000 | 0.002 | 0.000 | 0.013 | 0.002 |  |
| **SCI_3d corr** | **Atf3** | **Hmox1** | **Jun** | **Hif1a** | **Stat3** | **Hspb1** | **Ddit3** | **Tlr4** | **Ptgs2** | **Il6** |
| **Atf3** | 1.000 | 0.994 | 0.972 | 0.983 | 0.991 | 0.948 | 0.896 | 0.928 | 0.770 | 0.850 |
| **Hmox1** | 0.994 | 1.000 | 0.945 | 0.964 | 0.981 | 0.949 | 0.872 | 0.921 | 0.778 | 0.826 |
| **Jun** | 0.972 | 0.945 | 1.000 | 0.982 | 0.980 | 0.925 | 0.896 | 0.910 | 0.758 | 0.875 |
| **Hif1a** | 0.983 | 0.964 | 0.982 | 1.000 | 0.973 | 0.892 | 0.931 | 0.926 | 0.739 | 0.917 |
| **Stat3** | 0.991 | 0.981 | 0.980 | 0.973 | 1.000 | 0.967 | 0.860 | 0.918 | 0.767 | 0.843 |
| **Hspb1** | 0.948 | 0.949 | 0.925 | 0.892 | 0.967 | 1.000 | 0.732 | 0.844 | 0.806 | 0.712 |
| **Ddit3** | 0.896 | 0.872 | 0.896 | 0.931 | 0.860 | 0.732 | 1.000 | 0.847 | 0.640 | 0.879 |
| **Tlr4** | 0.928 | 0.921 | 0.910 | 0.926 | 0.918 | 0.844 | 0.847 | 1.000 | 0.545 | 0.759 |
| **Ptgs2** | 0.770 | 0.778 | 0.758 | 0.739 | 0.767 | 0.806 | 0.640 | 0.545 | 1.000 | 0.727 |
| **Il6** | 0.850 | 0.826 | 0.875 | 0.917 | 0.843 | 0.712 | 0.879 | 0.759 | 0.727 | 1.000 |
| **SCI_3d p value** | **Atf3** | **Hmox1** | **Jun** | **Hif1a** | **Stat3** | **Hspb1** | **Ddit3** | **Tlr4** | **Ptgs2** | **Il6** |
| **Atf3** |  | 0.000 | 0.000 | 0.000 | 0.000 | 0.000 | 0.003 | 0.001 | 0.025 | 0.007 |
| **Hmox1** | 0.000 |  | 0.000 | 0.000 | 0.000 | 0.000 | 0.005 | 0.001 | 0.023 | 0.012 |
| **Jun** | 0.000 | 0.000 |  | 0.000 | 0.000 | 0.001 | 0.003 | 0.002 | 0.029 | 0.004 |
| **Hif1a** | 0.000 | 0.000 | 0.000 |  | 0.000 | 0.003 | 0.001 | 0.001 | 0.036 | 0.001 |
| **Stat3** | 0.000 | 0.000 | 0.000 | 0.000 |  | 0.000 | 0.006 | 0.001 | 0.026 | 0.009 |
| **Hspb1** | 0.000 | 0.000 | 0.001 | 0.003 | 0.000 |  | 0.039 | 0.008 | 0.016 | 0.047 |
| **Ddit3** | 0.003 | 0.005 | 0.003 | 0.001 | 0.006 | 0.039 |  | 0.008 | 0.087 | 0.004 |
| **Tlr4** | 0.001 | 0.001 | 0.002 | 0.001 | 0.001 | 0.008 | 0.008 |  | 0.162 | 0.029 |
| **Ptgs2** | 0.025 | 0.023 | 0.029 | 0.036 | 0.026 | 0.016 | 0.087 | 0.162 |  | 0.041 |
| **Il6** | 0.007 | 0.012 | 0.004 | 0.001 | 0.009 | 0.047 | 0.004 | 0.029 | 0.041 |  |
| **SCI_7d corr** | **Atf3** | **Jun** | **Cd44** | **Hmox1** | **Vegfa** | **Stat3** | **Tlr4** | **Nfe2l2** | **Cdkn1a** | **Rela** |
| **Atf3** | 1.000 | 0.967 | 0.985 | 0.992 | -0.891 | 0.977 | 0.955 | 0.950 | 0.849 | 0.920 |
| **Jun** | 0.967 | 1.000 | 0.987 | 0.931 | -0.919 | 0.964 | 0.932 | 0.944 | 0.832 | 0.896 |
| **Cd44** | 0.985 | 0.987 | 1.000 | 0.962 | -0.926 | 0.986 | 0.940 | 0.954 | 0.857 | 0.926 |
| **Hmox1** | 0.992 | 0.931 | 0.962 | 1.000 | -0.839 | 0.965 | 0.959 | 0.942 | 0.829 | 0.920 |
| **Vegfa** | -0.891 | -0.919 | -0.926 | -0.839 | 1.000 | -0.875 | -0.765 | -0.825 | -0.888 | -0.797 |
| **Stat3** | 0.977 | 0.964 | 0.986 | 0.965 | -0.875 | 1.000 | 0.935 | 0.944 | 0.820 | 0.960 |
| **Tlr4** | 0.955 | 0.932 | 0.940 | 0.959 | -0.765 | 0.935 | 1.000 | 0.978 | 0.783 | 0.906 |
| **Nfe2l2** | 0.950 | 0.944 | 0.954 | 0.942 | -0.825 | 0.944 | 0.978 | 1.000 | 0.872 | 0.944 |
| **Cdkn1a** | 0.849 | 0.832 | 0.857 | 0.829 | -0.888 | 0.820 | 0.783 | 0.872 | 1.000 | 0.863 |
| **Rela** | 0.920 | 0.896 | 0.926 | 0.920 | -0.797 | 0.960 | 0.906 | 0.944 | 0.863 | 1.000 |
| **SCI_7d p value** | **Atf3** | **Jun** | **Cd44** | **Hmox1** | **Vegfa** | **Stat3** | **Tlr4** | **Nfe2l2** | **Cdkn1a** | **Rela** |
| **Atf3** |  | 0.000 | 0.000 | 0.000 | 0.003 | 0.000 | 0.000 | 0.000 | 0.008 | 0.001 |
| **Jun** | 0.000 |  | 0.000 | 0.001 | 0.001 | 0.000 | 0.001 | 0.000 | 0.010 | 0.003 |
| **Cd44** | 0.000 | 0.000 |  | 0.000 | 0.001 | 0.000 | 0.001 | 0.000 | 0.007 | 0.001 |
| **Hmox1** | 0.000 | 0.001 | 0.000 |  | 0.009 | 0.000 | 0.000 | 0.000 | 0.011 | 0.001 |
| **Vegfa** | 0.003 | 0.001 | 0.001 | 0.009 |  | 0.004 | 0.027 | 0.012 | 0.003 | 0.018 |
| **Stat3** | 0.000 | 0.000 | 0.000 | 0.000 | 0.004 |  | 0.001 | 0.000 | 0.013 | 0.000 |
| **Tlr4** | 0.000 | 0.001 | 0.001 | 0.000 | 0.027 | 0.001 |  | 0.000 | 0.021 | 0.002 |
| **Nfe2l2** | 0.000 | 0.000 | 0.000 | 0.000 | 0.012 | 0.000 | 0.000 |  | 0.005 | 0.000 |
| **Cdkn1a** | 0.008 | 0.010 | 0.007 | 0.011 | 0.003 | 0.013 | 0.021 | 0.005 |  | 0.006 |
| **Rela** | 0.001 | 0.003 | 0.001 | 0.001 | 0.018 | 0.000 | 0.002 | 0.000 | 0.006 |  |
