# Supplementary material for: Ferroptosis-associated myeloid cell heterogeneity and inflammatory amplification following spinal cord injury
Source: Front Immunol. 2026 Apr 22;17:1831161. doi: 10.3389/fimmu.2026.1831161 (PMC13143767; doi:10.3389/fimmu.2026.1831161)
Supplement: Supplementary file 1 [file DataSheet1.zip › Supplementary Table S11.docx]

| Supplementary Table S11. Detailed interactions in the ceRNA network of key hub gene. | | | | | | |
| --- | --- | --- | --- | --- | --- | --- |
| **Interaction** | **RNA1_type** | **RNA1** | **RNA2_type** | **RNA2** | **RNA1_degree** | **RNA2_degree** |
| lncRNA–miRNA | lncRNA | FGD5-AS1 | miRNA | miR-16-5p | 3 | 14 |
| lncRNA–miRNA | lncRNA | AC079781.5 | miRNA | miR-16-5p | 2 | 14 |
| lncRNA–miRNA | lncRNA | STAG3L5P-PVRIG2P-PILRB | miRNA | miR-16-5p | 2 | 14 |
| lncRNA–miRNA | lncRNA | KCNQ1OT1 | miRNA | miR-16-5p | 3 | 14 |
| lncRNA–miRNA | lncRNA | NEAT1 | miRNA | miR-16-5p | 6 | 14 |
| lncRNA–miRNA | lncRNA | AC020978.7 | miRNA | miR-16-5p | 2 | 14 |
| lncRNA–miRNA | lncRNA | MIR497HG | miRNA | miR-16-5p | 2 | 14 |
| lncRNA–miRNA | lncRNA | AC018628.1 | miRNA | miR-16-5p | 3 | 14 |
| lncRNA–miRNA | lncRNA | SNHG16 | miRNA | miR-16-5p | 3 | 14 |
| lncRNA–miRNA | lncRNA | AC005261.1 | miRNA | miR-16-5p | 4 | 14 |
| lncRNA–miRNA | lncRNA | XIST | miRNA | miR-16-5p | 7 | 14 |
| lncRNA–miRNA | lncRNA | FGD5-AS1 | miRNA | miR-107-3p | 3 | 8 |
| lncRNA–miRNA | lncRNA | H19 | miRNA | miR-107-3p | 2 | 8 |
| lncRNA–miRNA | lncRNA | KCNQ1OT1 | miRNA | miR-107-3p | 3 | 8 |
| lncRNA–miRNA | lncRNA | AC018628.1 | miRNA | miR-107-3p | 3 | 8 |
| lncRNA–miRNA | lncRNA | XIST | miRNA | miR-107-3p | 7 | 8 |
| lncRNA–miRNA | lncRNA | GAS5 | miRNA | miR-26a-5p | 1 | 6 |
| lncRNA–miRNA | lncRNA | HCG11 | miRNA | miR-26a-5p | 1 | 6 |
| lncRNA–miRNA | lncRNA | MALAT1 | miRNA | miR-26a-5p | 3 | 6 |
| lncRNA–miRNA | lncRNA | AL356488.2 | miRNA | miR-423-5p | 1 | 8 |
| lncRNA–miRNA | lncRNA | NEAT1 | miRNA | miR-423-5p | 6 | 8 |
| lncRNA–miRNA | lncRNA | AC009133.5 | miRNA | miR-423-5p | 1 | 8 |
| lncRNA–miRNA | lncRNA | MIR663AHG | miRNA | miR-423-5p | 1 | 8 |
| lncRNA–miRNA | lncRNA | MIRLET7BHG | miRNA | miR-423-5p | 1 | 8 |
| lncRNA–miRNA | lncRNA | XIST | miRNA | miR-423-5p | 7 | 8 |
| lncRNA–miRNA | lncRNA | NEAT1 | miRNA | miR-150-5p | 6 | 5 |
| lncRNA–miRNA | lncRNA | MALAT1 | miRNA | miR-150-5p | 3 | 5 |
| lncRNA–miRNA | lncRNA | AC005261.1 | miRNA | miR-150-5p | 4 | 5 |
| lncRNA–miRNA | lncRNA | XIST | miRNA | miR-150-5p | 7 | 5 |
| lncRNA–miRNA | lncRNA | AC084082.1 | miRNA | miR-125b-5p | 1 | 4 |
| lncRNA–miRNA | lncRNA | AC108134.2 | miRNA | miR-125b-5p | 1 | 4 |
| lncRNA–miRNA | lncRNA | XIST | miRNA | miR-125b-5p | 7 | 4 |
| lncRNA–miRNA | lncRNA | AC007036.3 | miRNA | miR-29a-3p | 1 | 4 |
| lncRNA–miRNA | lncRNA | NEAT1 | miRNA | miR-29a-3p | 6 | 4 |
| lncRNA–miRNA | lncRNA | OIP5-AS1 | miRNA | miR-29a-3p | 1 | 4 |
| lncRNA–miRNA | lncRNA | LINC01618 | miRNA | miR-17-5p | 1 | 9 |
| lncRNA–miRNA | lncRNA | AC021078.1 | miRNA | miR-17-5p | 1 | 9 |
| lncRNA–miRNA | lncRNA | H19 | miRNA | miR-17-5p | 2 | 9 |
| lncRNA–miRNA | lncRNA | NEAT1 | miRNA | miR-17-5p | 6 | 9 |
| lncRNA–miRNA | lncRNA | MALAT1 | miRNA | miR-17-5p | 3 | 9 |
| lncRNA–miRNA | lncRNA | SNHG16 | miRNA | miR-17-5p | 3 | 9 |
| lncRNA–miRNA | lncRNA | AC005261.1 | miRNA | miR-17-5p | 4 | 9 |
| lncRNA–miRNA | lncRNA | XIST | miRNA | miR-17-5p | 7 | 9 |
| lncRNA–miRNA | lncRNA | FGD5-AS1 | miRNA | miR-15b-5p | 3 | 14 |
| lncRNA–miRNA | lncRNA | AC079781.5 | miRNA | miR-15b-5p | 2 | 14 |
| lncRNA–miRNA | lncRNA | STAG3L5P-PVRIG2P-PILRB | miRNA | miR-15b-5p | 2 | 14 |
| lncRNA–miRNA | lncRNA | KCNQ1OT1 | miRNA | miR-15b-5p | 3 | 14 |
| lncRNA–miRNA | lncRNA | NEAT1 | miRNA | miR-15b-5p | 6 | 14 |
| lncRNA–miRNA | lncRNA | AC020978.7 | miRNA | miR-15b-5p | 2 | 14 |
| lncRNA–miRNA | lncRNA | MIR497HG | miRNA | miR-15b-5p | 2 | 14 |
| lncRNA–miRNA | lncRNA | AC018628.1 | miRNA | miR-15b-5p | 3 | 14 |
| lncRNA–miRNA | lncRNA | SNHG16 | miRNA | miR-15b-5p | 3 | 14 |
| lncRNA–miRNA | lncRNA | AC005261.1 | miRNA | miR-15b-5p | 4 | 14 |
| lncRNA–miRNA | lncRNA | XIST | miRNA | miR-15b-5p | 7 | 14 |
| miRNA–mRNA | miRNA | miR-16-5p | mRNA | Tlr4 | 14 | 4 |
| miRNA–mRNA | miRNA | miR-16-5p | mRNA | Mapk8 | 14 | 4 |
| miRNA–mRNA | miRNA | miR-16-5p | mRNA | Vegfa | 14 | 6 |
| miRNA–mRNA | miRNA | miR-15b-5p | mRNA | Tlr4 | 14 | 4 |
| miRNA–mRNA | miRNA | miR-15b-5p | mRNA | Mapk8 | 14 | 4 |
| miRNA–mRNA | miRNA | miR-15b-5p | mRNA | Vegfa | 14 | 6 |
| miRNA–mRNA | miRNA | miR-107-3p | mRNA | Ptgs2 | 8 | 2 |
| miRNA–mRNA | miRNA | miR-107-3p | mRNA | Tlr4 | 8 | 4 |
| miRNA–mRNA | miRNA | miR-107-3p | mRNA | Mapk8 | 8 | 4 |
| miRNA–mRNA | miRNA | miR-26a-5p | mRNA | Il6 | 6 | 1 |
| miRNA–mRNA | miRNA | miR-26a-5p | mRNA | Vegfa | 6 | 6 |
| miRNA–mRNA | miRNA | miR-26a-5p | mRNA | Ptgs2 | 6 | 2 |
| miRNA–mRNA | miRNA | miR-423-5p | mRNA | Vegfa | 8 | 6 |
| miRNA–mRNA | miRNA | miR-423-5p | mRNA | Hmox1 | 8 | 1 |
| miRNA–mRNA | miRNA | miR-150-5p | mRNA | Vegfa | 5 | 6 |
| miRNA–mRNA | miRNA | miR-125b-5p | mRNA | Tlr4 | 4 | 4 |
| miRNA–mRNA | miRNA | miR-29a-3p | mRNA | Vegfa | 4 | 6 |
| miRNA–mRNA | miRNA | miR-17-5p | mRNA | Mapk8 | 9 | 4 |
