# Supplementary material for: Ferroptosis-associated myeloid cell heterogeneity and inflammatory amplification following spinal cord injury
Source: Front Immunol. 2026 Apr 22;17:1831161. doi: 10.3389/fimmu.2026.1831161 (PMC13143767; doi:10.3389/fimmu.2026.1831161)
Supplement: Supplementary file 1 [file DataSheet1.zip › Supplementary Table S12.docx]

| Supplementary Table S12. TF–mRNA regulatory interactions of key hub genes | | | |
| --- | --- | --- | --- |
| **TF** | **TargetGene** | **TF_degree** | **Target_degree** |
| Nfkb1 | Ptgs2 | 5 | 30 |
| Ets1 | Ptgs2 | 4 | 30 |
| Jun | Ptgs2 | 4 | 30 |
| Ep300 | Ptgs2 | 3 | 30 |
| Hif1a | Ptgs2 | 3 | 30 |
| Sp1 | Ptgs2 | 3 | 30 |
| Sp3 | Ptgs2 | 3 | 30 |
| Ahr | Ptgs2 | 2 | 30 |
| Apex1 | Ptgs2 | 2 | 30 |
| Cebpb | Ptgs2 | 2 | 30 |
| Ctnnb1 | Ptgs2 | 2 | 30 |
| Ppara | Ptgs2 | 2 | 30 |
| Pparg | Ptgs2 | 2 | 30 |
| Rela | Ptgs2 | 2 | 30 |
| Trp53 | Ptgs2 | 2 | 30 |
| Cebpa | Ptgs2 | 1 | 30 |
| Creb1 | Ptgs2 | 1 | 30 |
| Etv4 | Ptgs2 | 1 | 30 |
| Etv5 | Ptgs2 | 1 | 30 |
| Foxm1 | Ptgs2 | 1 | 30 |
| Ikbkb | Ptgs2 | 1 | 30 |
| Klf4 | Ptgs2 | 1 | 30 |
| Lef1 | Ptgs2 | 1 | 30 |
| Myb | Ptgs2 | 1 | 30 |
| Nfatc4 | Ptgs2 | 1 | 30 |
| Nfil3 | Ptgs2 | 1 | 30 |
| Nfkbia | Ptgs2 | 1 | 30 |
| Nr3c1 | Ptgs2 | 1 | 30 |
| Snai1 | Ptgs2 | 1 | 30 |
| Tcf4 | Ptgs2 | 1 | 30 |
| Nfkb1 | Vegfa | 5 | 24 |
| Ets1 | Vegfa | 4 | 24 |
| Hif1a | Vegfa | 3 | 24 |
| Sp1 | Vegfa | 3 | 24 |
| Sp3 | Vegfa | 3 | 24 |
| Ctnnb1 | Vegfa | 2 | 24 |
| Esr2 | Vegfa | 2 | 24 |
| Stat3 | Vegfa | 2 | 24 |
| E2f1 | Vegfa | 1 | 24 |
| Esr1 | Vegfa | 1 | 24 |
| Hdgf | Vegfa | 1 | 24 |
| Hexim1 | Vegfa | 1 | 24 |
| Klf5 | Vegfa | 1 | 24 |
| Nr2f2 | Vegfa | 1 | 24 |
| Smad3 | Vegfa | 1 | 24 |
| Smad4 | Vegfa | 1 | 24 |
| Smad7 | Vegfa | 1 | 24 |
| Sox9 | Vegfa | 1 | 24 |
| Sp4 | Vegfa | 1 | 24 |
| Sp7 | Vegfa | 1 | 24 |
| Tfap2a | Vegfa | 1 | 24 |
| Trp63 | Vegfa | 1 | 24 |
| Wt1 | Vegfa | 1 | 24 |
| Xbp1 | Vegfa | 1 | 24 |
| Nfkb1 | Il6 | 5 | 20 |
| Jun | Il6 | 4 | 20 |
| Ep300 | Il6 | 3 | 20 |
| Sp1 | Il6 | 3 | 20 |
| Sp3 | Il6 | 3 | 20 |
| Ahr | Il6 | 2 | 20 |
| Cebpb | Il6 | 2 | 20 |
| Crebbp | Il6 | 2 | 20 |
| Elk1 | Il6 | 2 | 20 |
| Fos | Il6 | 2 | 20 |
| Ppara | Il6 | 2 | 20 |
| Rela | Il6 | 2 | 20 |
| Stat3 | Il6 | 2 | 20 |
| Atf3 | Il6 | 1 | 20 |
| Atf4 | Il6 | 1 | 20 |
| Cebpg | Il6 | 1 | 20 |
| Ctr9 | Il6 | 1 | 20 |
| Hdac1 | Il6 | 1 | 20 |
| Sirt1 | Il6 | 1 | 20 |
| Stat1 | Il6 | 1 | 20 |
| Nfkb1 | Hmox1 | 5 | 19 |
| Ets1 | Hmox1 | 4 | 19 |
| Jun | Hmox1 | 4 | 19 |
| Ep300 | Hmox1 | 3 | 19 |
| Hif1a | Hmox1 | 3 | 19 |
| Crebbp | Hmox1 | 2 | 19 |
| Elk1 | Hmox1 | 2 | 19 |
| Pparg | Hmox1 | 2 | 19 |
| Trp53 | Hmox1 | 2 | 19 |
| Bach1 | Hmox1 | 1 | 19 |
| Ets2 | Hmox1 | 1 | 19 |
| Mafk | Hmox1 | 1 | 19 |
| Nfe2l2 | Hmox1 | 1 | 19 |
| Nrf1 | Hmox1 | 1 | 19 |
| Nupr1 | Hmox1 | 1 | 19 |
| Smarca4 | Hmox1 | 1 | 19 |
| Srebf1 | Hmox1 | 1 | 19 |
| Srebf2 | Hmox1 | 1 | 19 |
| Usf2 | Hmox1 | 1 | 19 |
| Nfkb1 | Tlr4 | 5 | 5 |
| Jun | Mapk8 | 4 | 5 |
| Ets1 | Tlr4 | 4 | 5 |
| Esr2 | Mapk8 | 2 | 5 |
| Fos | Mapk8 | 2 | 5 |
| Apex1 | Tlr4 | 2 | 5 |
| Egr1 | Mapk8 | 1 | 5 |
| Zbtb17 | Mapk8 | 1 | 5 |
| Pax6 | Tlr4 | 1 | 5 |
| Spi1 | Tlr4 | 1 | 5 |
| Gtf2b | Hspb1 | 1 | 3 |
| Hsf4 | Hspb1 | 1 | 3 |
| Nfya | Hspb1 | 1 | 3 |
