# Supplementary material for: Ferroptosis-associated myeloid cell heterogeneity and inflammatory amplification following spinal cord injury
Source: Front Immunol. 2026 Apr 22;17:1831161. doi: 10.3389/fimmu.2026.1831161 (PMC13143767; doi:10.3389/fimmu.2026.1831161)
Supplement: Supplementary file 1 [file DataSheet1.zip › Supplementary Table S13.docx]

| Supplementary Table S13. miRNA–mRNA interactions of key hub genes | | | |
| --- | --- | --- | --- |
| **miRNA** | **TargetGene** | **miRNA_degree** | **Target_degree** |
| miR-322-5p | Mapk8 | 3 | 43 |
| miR-16-5p | Mapk8 | 3 | 43 |
| miR-15b-5p | Mapk8 | 3 | 43 |
| miR-103-3p | Mapk8 | 3 | 43 |
| miR-15a-5p | Mapk8 | 3 | 43 |
| miR-107-3p | Mapk8 | 3 | 43 |
| miR-378a-5p | Mapk8 | 2 | 43 |
| miR-130b-3p | Mapk8 | 2 | 43 |
| miR-98-5p | Mapk8 | 2 | 43 |
| miR-101a-3p | Mapk8 | 2 | 43 |
| miR-25-3p | Mapk8 | 1 | 43 |
| miR-31-5p | Mapk8 | 1 | 43 |
| miR-20a-5p | Mapk8 | 1 | 43 |
| miR-27b-3p | Mapk8 | 1 | 43 |
| miR-92b-3p | Mapk8 | 1 | 43 |
| miR-210-3p | Mapk8 | 1 | 43 |
| miR-222-3p | Mapk8 | 1 | 43 |
| miR-762 | Mapk8 | 1 | 43 |
| miR-20b-5p | Mapk8 | 1 | 43 |
| miR-30d-5p | Mapk8 | 1 | 43 |
| miR-133a-5p | Mapk8 | 1 | 43 |
| miR-301a-3p | Mapk8 | 1 | 43 |
| miR-203-3p | Mapk8 | 1 | 43 |
| miR-30a-5p | Mapk8 | 1 | 43 |
| miR-18a-5p | Mapk8 | 1 | 43 |
| miR-19a-3p | Mapk8 | 1 | 43 |
| miR-202-3p | Mapk8 | 1 | 43 |
| miR-17-5p | Mapk8 | 1 | 43 |
| miR-30e-5p | Mapk8 | 1 | 43 |
| miR-19b-3p | Mapk8 | 1 | 43 |
| miR-92a-3p | Mapk8 | 1 | 43 |
| miR-130a-3p | Mapk8 | 1 | 43 |
| miR-34a-5p | Mapk8 | 1 | 43 |
| miR-214-3p | Mapk8 | 1 | 43 |
| miR-761 | Mapk8 | 1 | 43 |
| miR-30b-5p | Mapk8 | 1 | 43 |
| miR-106b-5p | Mapk8 | 1 | 43 |
| miR-497a-5p | Mapk8 | 1 | 43 |
| miR-500-3p | Mapk8 | 1 | 43 |
| miR-27a-3p | Mapk8 | 1 | 43 |
| miR-301b-3p | Mapk8 | 1 | 43 |
| miR-30c-5p | Mapk8 | 1 | 43 |
| miR-467a-5p | Mapk8 | 1 | 43 |
| miR-322-5p | Vegfa | 3 | 19 |
| miR-15a-5p | Vegfa | 3 | 19 |
| miR-16-5p | Vegfa | 3 | 19 |
| miR-26a-5p | Vegfa | 3 | 19 |
| miR-15b-5p | Vegfa | 3 | 19 |
| miR-423-5p | Vegfa | 2 | 19 |
| miR-378a-5p | Vegfa | 2 | 19 |
| miR-130b-3p | Vegfa | 2 | 19 |
| miR-150-5p | Vegfa | 1 | 19 |
| miR-30c-1-3p | Vegfa | 1 | 19 |
| miR-29c-3p | Vegfa | 1 | 19 |
| miR-29b-3p | Vegfa | 1 | 19 |
| miR-23a-3p | Vegfa | 1 | 19 |
| miR-185-5p | Vegfa | 1 | 19 |
| miR-1a-3p | Vegfa | 1 | 19 |
| miR-29a-3p | Vegfa | 1 | 19 |
| miR-206-3p | Vegfa | 1 | 19 |
| miR-669b-5p | Vegfa | 1 | 19 |
| miR-205-5p | Vegfa | 1 | 19 |
| miR-16-5p | Tlr4 | 3 | 13 |
| miR-15b-5p | Tlr4 | 3 | 13 |
| miR-103-3p | Tlr4 | 3 | 13 |
| miR-107-3p | Tlr4 | 3 | 13 |
| miR-322-5p | Tlr4 | 3 | 13 |
| miR-15a-5p | Tlr4 | 3 | 13 |
| miR-677-5p | Tlr4 | 1 | 13 |
| miR-125b-5p | Tlr4 | 1 | 13 |
| miR-466c-3p | Tlr4 | 1 | 13 |
| miR-326-3p | Tlr4 | 1 | 13 |
| miR-466b-3p | Tlr4 | 1 | 13 |
| miR-362-5p | Tlr4 | 1 | 13 |
| miR-207 | Tlr4 | 1 | 13 |
| miR-103-3p | Ptgs2 | 3 | 6 |
| miR-107-3p | Ptgs2 | 3 | 6 |
| miR-26a-5p | Ptgs2 | 3 | 6 |
| miR-26b-5p | Ptgs2 | 2 | 6 |
| miR-101a-3p | Ptgs2 | 2 | 6 |
| miR-7a-5p | Ptgs2 | 1 | 6 |
| miR-26a-5p | Il6 | 3 | 5 |
| miR-98-5p | Il6 | 2 | 5 |
| miR-26b-5p | Il6 | 2 | 5 |
| miR-181d-5p | Il6 | 1 | 5 |
| miR-223-3p | Il6 | 1 | 5 |
| miR-423-5p | Hmox1 | 2 | 2 |
| miR-183-5p | Hmox1 | 1 | 2 |
