# Supplementary material for: Ferroptosis-associated myeloid cell heterogeneity and inflammatory amplification following spinal cord injury
Source: Front Immunol. 2026 Apr 22;17:1831161. doi: 10.3389/fimmu.2026.1831161 (PMC13143767; doi:10.3389/fimmu.2026.1831161)
Supplement: Supplementary file 1 [file DataSheet1.zip › Supplementary Table S2.docx]

| Supplementary Table S2. Ferroptosis-related differentially expressed genes identified at 1, 3, and 7 days after SCI | | | |
| --- | --- | --- | --- |
| Group | **LogFC** | **Adj.Pvalue** | **Gene title** |
| SCI-1d(57) |  |  |  |
| Acsl3 | -1.18 | 0.00130 | Acyl-CoA synthetase long chain family member 3 |
| Ano6 | 2.45 | 0.00004 | Anoctamin 6 |
| Atf3 | 5.96 | 0.00015 | Activating transcription factor 3 |
| Atp6v1g2 | -4.23 | 0.00401 | ATPase H+ transporting V1 subunit G2 |
| Aurka | 2.88 | 0.00032 | Aurora kinase A |
| Bid | 1.49 | 0.00027 | BH3 interacting domain death agonist |
| Bnip3 | -1.15 | 0.00026 | BCL2 interacting protein 3 |
| Capg | 5.47 | 0.00018 | Capping actin protein, gelsolin like |
| Cav1 | 1.14 | 0.00056 | Caveolin 1 |
| Cbs | -1.61 | 0.00121 | Cystathionine beta-synthase |
| Cd44 | 2.59 | 0.00016 | CD44 molecule |
| Chac1 | 1.16 | 0.03020 | ChaC glutathione specific gamma-glutamylcyclotransferase 1 |
| Chmp6 | 1.86 | 0.00110 | Charged multivesicular body protein 6 |
| Cybb | 5.98 | 0.00006 | Cytochrome b-245 beta chain |
| Ddit4 | -1.35 | 0.01499 | DNA damage inducible transcript 4 |
| Eif2s1 | 1.11 | 0.00010 | Eukaryotic translation initiation factor 2 subunit 1 |
| Elavl1 | 1.00 | 0.03823 | ELAV like RNA binding protein 1 |
| Enpp2 | -1.52 | 0.00664 | Ectonucleotide pyrophosphatase/phosphodiesterase 2 |
| Fancd2 | 2.20 | 0.00333 | Fanconi anemia complementation group D2 |
| Fbxw7 | -2.68 | 0.00015 | F-box and WD repeat domain containing 7 |
| G6pd | 1.08 | 0.00010 | Glucose-6-phosphate dehydrogenase |
| Gabarapl1 | -1.94 | 0.00032 | GABA type A receptor associated protein like 1 |
| Gabarapl2 | -1.19 | 0.00121 | GABA type A receptor associated protein like 2 |
| Gch1 | 1.49 | 0.00285 | GTP cyclohydrolase 1 |
| Gls2 | -3.42 | 0.00011 | Glutaminase 2 |
| Got1 | -2.15 | 0.00016 | Glutamic-oxaloacetic transaminase 1 |
| Hells | 1.95 | 0.00406 | Helicase lymphoid specific |
| Hic1 | 1.02 | 0.02286 | Hypermethylated in cancer 1 |
| Hmox1 | 8.69 | 0.00025 | Heme oxygenase 1 |
| Hspb1 | 1.43 | 0.00499 | Heat shock protein family B (small) member 1 |
| Il33 | 3.72 | 0.00000 | Interleukin 33 |
| Jun | 1.12 | 0.00105 | Jun proto-oncogene |
| Lpcat3 | 1.22 | 0.00130 | Lysophosphatidylcholine acyltransferase 3 |
| Lpin1 | -1.56 | 0.00018 | Lipin 1 |
| Lurap1l | -1.64 | 0.00184 | Leucine rich adaptor protein 1 like |
| Mapk8 | -1.15 | 0.00049 | Mitogen-activated protein kinase 8 |
| Mapk9 | -3.10 | 0.00016 | Mitogen-activated protein kinase 9 |
| Mt3 | -1.81 | 0.00994 | Metallothionein 3 |
| Plin2 | 4.21 | 0.00035 | Perilipin 2 |
| Pml | 1.35 | 0.03512 | Promyelocytic leukemia |
| Psat1 | -1.39 | 0.01316 | Phosphoserine aminotransferase 1 |
| Ptgs2 | 1.86 | 0.03106 | Prostaglandin-endoperoxide synthase 2 |
| Rgs4 | -2.14 | 0.00000 | Regulator of G protein signaling 4 |
| Ripk1 | 1.79 | 0.00526 | Receptor interacting serine/threonine kinase 1 |
| Sat1 | 2.33 | 0.00007 | Spermidine/spermine N1-acetyltransferase 1 |
| Scd | -1.89 | 0.00027 | Stearoyl-CoA desaturase |
| Slc2a3 | -1.18 | 0.00023 | Solute carrier family 2 member 3 |
| Slc38a1 | -1.62 | 0.00564 | Solute carrier family 38 member 1 |
| Srxn1 | 1.89 | 0.00002 | Sulfiredoxin 1 |
| Stat3 | 1.00 | 0.00389 | Signal transducer and activator of transcription 3 |
| Tgfbr1 | 2.27 | 0.00018 | Transforming growth factor beta receptor 1 |
| Tlr4 | 2.99 | 0.00114 | Toll like receptor 4 |
| Tp53 | 1.18 | 0.00403 | Tumor protein p53 |
| Tsc22d3 | -1.38 | 0.00036 | TSC22 domain family member 3 |
| Ulk1 | -1.12 | 0.00061 | Unc-51 like autophagy activating kinase 1 |
| Vldlr | -1.17 | 0.01072 | Very low density lipoprotein receptor |
| Zfp36 | 1.40 | 0.00925 | ZFP36 ring finger protein |
| SCI-3d(49) |  |  |  |
| Acsf2 | -1.44 | 0.00079 | Acyl-CoA synthetase family member 2 |
| Acsl4 | 1.38 | 0.00077 | Acyl-CoA synthetase long chain family member 4 |
| Ano6 | 2.31 | 0.00005 | Anoctamin 6 |
| Asns | 1.62 | 0.00016 | Asparagine synthetase |
| Atf3 | 6.25 | 0.00010 | Activating transcription factor 3 |
| Atf4 | 1.89 | 0.00010 | Activating transcription factor 4 |
| Capg | 4.07 | 0.00033 | Capping actin protein, gelsolin like |
| Cbs | -1.38 | 0.00231 | Cystathionine beta-synthase |
| Cd44 | 3.04 | 0.00012 | CD44 molecule |
| Cdkn1a | 1.78 | 0.00231 | Cyclin dependent kinase inhibitor 1A |
| Cdo1 | 1.75 | 0.00001 | Cysteine dioxygenase type 1 |
| Chac1 | 3.47 | 0.00016 | ChaC glutathione specific gamma-glutamylcyclotransferase 1 |
| Chmp6 | 1.11 | 0.00536 | Charged multivesicular body protein 6 |
| Cxcl2 | 4.55 | 0.00069 | C-X-C motif chemokine ligand 2 |
| Cybb | 2.58 | 0.02649 | Cytochrome b-245 beta chain |
| Ddit3 | 1.09 | 0.00304 | DNA damage inducible transcript 3 |
| Ddit4 | -1.15 | 0.00765 | DNA damage inducible transcript 4 |
| Eif2s1 | 1.63 | 0.00010 | Eukaryotic translation initiation factor 2 subunit 1 |
| Enpp2 | -1.38 | 0.00011 | Ectonucleotide pyrophosphatase/phosphodiesterase 2 |
| Gch1 | 1.85 | 0.00064 | GTP cyclohydrolase 1 |
| Gls2 | -1.61 | 0.01848 | Glutaminase 2 |
| Gpx2 | 9.23 | 0.00000 | Glutathione peroxidase 2 |
| Hif1a | 1.14 | 0.00037 | Hypoxia inducible factor 1 alpha |
| Hmox1 | 7.92 | 0.00021 | Heme oxygenase 1 |
| Hspb1 | 1.86 | 0.00135 | Heat shock protein family B (small) member 1 |
| Il33 | 3.26 | 0.00001 | Interleukin 33 |
| Il6 | 3.19 | 0.01283 | Interleukin 6 |
| Jun | 1.61 | 0.00035 | Jun proto-oncogene |
| Klhl24 | -1.44 | 0.00042 | Kelch like family member 24 |
| Lurap1l | 1.48 | 0.00724 | Leucine rich adaptor protein 1 like |
| Mapk9 | -1.12 | 0.00770 | Mitogen-activated protein kinase 9 |
| Nras | 1.04 | 0.00067 | Neuroblastoma RAS viral oncogene homolog |
| Plin2 | 3.83 | 0.00045 | Perilipin 2 |
| Psat1 | -1.07 | 0.01487 | Phosphoserine aminotransferase 1 |
| Ptgs2 | 1.89 | 0.01000 | Prostaglandin-endoperoxide synthase 2 |
| Ripk1 | 1.00 | 0.00362 | Receptor interacting serine/threonine kinase 1 |
| Sat1 | 1.80 | 0.00025 | Spermidine/spermine N1-acetyltransferase 1 |
| Sesn2 | 1.88 | 0.00151 | Sestrin 2 |
| Slc1a5 | 1.25 | 0.00228 | Solute carrier family 1 member 5 |
| Slc3a2 | 1.24 | 0.00057 | Solute carrier family 3 member 2 |
| Slc7a11 | 1.06 | 0.04255 | Solute carrier family 7 member 11 |
| Srxn1 | 3.26 | 0.00005 | Sulfiredoxin 1 |
| Stat3 | 1.79 | 0.00037 | Signal transducer and activator of transcription 3 |
| Tgfbr1 | -1.40 | 0.02387 | Transforming growth factor beta receptor 1 |
| Tlr4 | 1.95 | 0.00879 | Toll like receptor 4 |
| Trib3 | 1.10 | 0.04020 | Tribbles pseudokinase 3 |
| Tsc22d3 | -1.18 | 0.00082 | TSC22 domain family member 3 |
| Txnrd1 | 2.16 | 0.00019 | Thioredoxin reductase 1 |
| Zfp36 | 2.40 | 0.00062 | ZFP36 ring finger protein |
| SCI-7d(36) |  |  |  |
| Ano6 | 2.23 | 0.00385 | Anoctamin 6 |
| Atf3 | 5.31 | 0.00206 | Activating transcription factor 3 |
| Atp6v1g2 | -2.40 | 0.01235 | ATPase H+ transporting V1 subunit G2 |
| Aurka | 1.34 | 0.01999 | Aurora kinase A |
| Bid | 1.18 | 0.01136 | BH3 interacting domain death agonist |
| Capg | 4.57 | 0.00388 | Capping actin protein, gelsolin like |
| Cd44 | 2.48 | 0.00267 | CD44 molecule |
| Cdkn1a | 1.39 | 0.01854 | Cyclin dependent kinase inhibitor 1A |
| Chac1 | -1.03 | 0.04900 | ChaC glutathione specific gamma-glutamylcyclotransferase 1 |
| Chmp6 | 1.64 | 0.01395 | Charged multivesicular body protein 6 |
| Cybb | 5.55 | 0.00269 | Cytochrome b-245 beta chain |
| Fbxw7 | -1.88 | 0.00847 | F-box and WD repeat domain containing 7 |
| Gabarapl1 | -1.31 | 0.00827 | GABA type A receptor associated protein like 1 |
| Gch1 | 1.09 | 0.01746 | GTP cyclohydrolase 1 |
| Gls2 | -3.04 | 0.00205 | Glutaminase 2 |
| Got1 | -1.53 | 0.01029 | Glutamic-oxaloacetic transaminase 1 |
| Hba1 | -1.13 | 0.00791 | Hemoglobin subunit alpha 1 |
| Hmox1 | 6.52 | 0.00354 | Heme oxygenase 1 |
| Il33 | 1.09 | 0.01868 | Interleukin 33 |
| Jun | 1.44 | 0.00242 | Jun proto-oncogene |
| Lpcat3 | 1.18 | 0.00548 | Lysophosphatidylcholine acyltransferase 3 |
| Lurap1l | -2.90 | 0.00197 | Leucine rich adaptor protein 1 like |
| Mapk9 | -2.24 | 0.00206 | Mitogen-activated protein kinase 9 |
| Nfe2l2 | 1.08 | 0.00807 | Nuclear factor, erythroid 2 like 2 |
| Plin2 | 3.54 | 0.00364 | Perilipin 2 |
| Pml | 1.66 | 0.01336 | Promyelocytic leukemia |
| Rela | 1.16 | 0.02509 | RELA proto-oncogene, NF-kB subunit |
| Rgs4 | -1.74 | 0.00166 | Regulator of G protein signaling 4 |
| Ripk1 | 1.63 | 0.01891 | Receptor interacting serine/threonine kinase 1 |
| Sat1 | 2.31 | 0.00382 | Spermidine/spermine N1-acetyltransferase 1 |
| Slc2a3 | -1.05 | 0.00298 | Solute carrier family 2 member 3 |
| Stat3 | 1.23 | 0.00597 | Signal transducer and activator of transcription 3 |
| Tgfbr1 | 2.18 | 0.00405 | Transforming growth factor beta receptor 1 |
| Tlr4 | 2.82 | 0.00761 | Toll like receptor 4 |
| Tsc22d3 | -1.05 | 0.02489 | TSC22 domain family member 3 |
| Vegfa | -1.01 | 0.00472 | Vascular endothelial growth factor A |
