# Supplementary material for: Ferroptosis-associated myeloid cell heterogeneity and inflammatory amplification following spinal cord injury
Source: Front Immunol. 2026 Apr 22;17:1831161. doi: 10.3389/fimmu.2026.1831161 (PMC13143767; doi:10.3389/fimmu.2026.1831161)
Supplement: Supplementary file 1 [file DataSheet1.zip › Supplementary Table S3.docx]

| **Supplementary Table S3. Gene lists of the four temporal expression clusters of FDEGs after SCI** | | | |
| --- | --- | --- | --- |
| Cluster_0 | Cluster_1 | Cluster_2 | Cluster_3 |
| Aurka | Acsf2 | Asns | Acsl4 |
| Bid | Acsl3 | Atf4 | Ano6 |
| Capg | Atp6v1g2 | Cdo1 | Atf3 |
| Cav1 | Bnip3 | Chac1 | Cd44 |
| Chmp6 | Cbs | Cxcl2 | Cdkn1a |
| Cybb | Ddit4 | Ddit3 | Eif2s1 |
| Elavl1 | Enpp2 | Gpx2 | Gch1 |
| Fancd2 | Fbxw7 | Hba1 | Hif1a |
| G6pd | Gabarapl1 | Il6 | Hmox1 |
| Hells | Gabarapl2 | Lurap1l | Hspb1 |
| Hic1 | Gls2 | Sesn2 | Il33 |
| Lpcat3 | Got1 | Slc2a3 | Jun |
| Nfe2l2 | Klhl24 | Slc7a11 | Nras |
| Pml | Lpin1 | Trib3 | Plin2 |
| Rela | Mapk8 | Txnrd1 | Ptgs2 |
| Ripk1 | Mapk9 | Vegfa | Slc1a5 |
| Sat1 | Mt3 |  | Slc3a2 |
| Tgfbr1 | Psat1 |  | Srxn1 |
| Tlr4 | Rgs4 |  | Stat3 |
|  | Scd |  | Tp53 |
|  | Slc38a1 |  | Zfp36 |
|  | Ulk1 |  |  |
|  | Vldlr |  |  |
