# Supplementary material for: Ferroptosis-associated myeloid cell heterogeneity and inflammatory amplification following spinal cord injury
Source: Front Immunol. 2026 Apr 22;17:1831161. doi: 10.3389/fimmu.2026.1831161 (PMC13143767; doi:10.3389/fimmu.2026.1831161)
Supplement: Supplementary file 1 [file DataSheet1.zip › Supplementary Table S4.docx]

| Supplementary Table S4. GO enrichment results of FDEGs at different time points after SCI | | | | | |
| --- | --- | --- | --- | --- | --- |
| Time | ID | Description | Count | p.adjust | geneID |
| SCI_1d | GO:2001233 | regulation of apoptotic signaling pathway | 16 | 6.891365979695e-10 | Fbxw7/Atf3/Cd44/Mapk9/Tgfbr1/Hmox1/Bid/Mapk8/Cav1/Tlr4/Tp53/Hells/Hspb1/Ripk1/Ptgs2/Pml |
| SCI_1d | GO:0072593 | reactive oxygen species metabolic process | 13 | 8.41821070106268e-10 | Cybb/G6pd/Gls2/Bnip3/Tlr4/Cbs/Gch1/Stat3/Tp53/Ripk1/Mt3/Ddit4/Elavl1 |
| SCI_1d | GO:0097193 | intrinsic apoptotic signaling pathway | 14 | 8.41821070106268e-10 | Fbxw7/Cd44/Hmox1/Bnip3/Bid/Cav1/Tp53/Hells/Hspb1/Ddit4/Hic1/Chac1/Ptgs2/Pml |
| SCI_1d | GO:0062197 | cellular response to chemical stress | 14 | 5.02890619998087e-09 | G6pd/Eif2s1/Mapk9/Bnip3/Mapk8/Cav1/Gch1/Fancd2/Tp53/Hspb1/Ripk1/Mt3/Ptgs2/Pml |
| SCI_1d | GO:2001235 | positive regulation of apoptotic signaling pathway | 11 | 5.02890619998087e-09 | Fbxw7/Atf3/Mapk9/Tgfbr1/Bid/Mapk8/Cav1/Tlr4/Tp53/Ripk1/Pml |
| SCI_1d | GO:0042594 | response to starvation | 12 | 1.6842231169835e-08 | Eif2s1/Atf3/Gabarapl1/Plin2/Mapk8/Cav1/Ulk1/Jun/Gabarapl2/Tp53/Zfp36/Vldlr |
| SCI_1d | GO:0009267 | cellular response to starvation | 11 | 1.6842231169835e-08 | Eif2s1/Atf3/Gabarapl1/Plin2/Mapk8/Cav1/Ulk1/Jun/Gabarapl2/Tp53/Vldlr |
| SCI_1d | GO:0034599 | cellular response to oxidative stress | 12 | 3.67757679146283e-08 | G6pd/Eif2s1/Mapk9/Bnip3/Mapk8/Gch1/Fancd2/Tp53/Hspb1/Ripk1/Mt3/Pml |
| SCI_1d | GO:0031669 | cellular response to nutrient levels | 12 | 4.2315469996793e-08 | Eif2s1/Atf3/Hmox1/Gabarapl1/Plin2/Mapk8/Cav1/Ulk1/Jun/Gabarapl2/Tp53/Vldlr |
| SCI_1d | GO:0036294 | cellular response to decreased oxygen levels | 11 | 7.15677086098378e-08 | Cybb/Bnip3/Mapk8/Cav1/Jun/Cbs/Stat3/Tp53/Mt3/Vldlr/Ptgs2 |
| SCI_1d | GO:0071453 | cellular response to oxygen levels | 11 | 1.35928944622216e-07 | Cybb/Bnip3/Mapk8/Cav1/Jun/Cbs/Stat3/Tp53/Mt3/Vldlr/Ptgs2 |
| SCI_1d | GO:0090594 | inflammatory response to wounding | 6 | 1.35928944622216e-07 | Il33/Cd44/Mapk9/Hmox1/Tlr4/Stat3 |
| SCI_1d | GO:0071456 | cellular response to hypoxia | 10 | 4.29792301838933e-07 | Cybb/Bnip3/Cav1/Jun/Cbs/Stat3/Tp53/Mt3/Vldlr/Ptgs2 |
| SCI_1d | GO:2000377 | regulation of reactive oxygen species metabolic process | 9 | 4.87034952141831e-07 | G6pd/Bnip3/Tlr4/Gch1/Stat3/Tp53/Ripk1/Mt3/Elavl1 |
| SCI_1d | GO:0046686 | response to cadmium ion | 7 | 5.76430142077285e-07 | Cybb/Mapk9/Got1/Hmox1/Mapk8/Enpp2/Mt3 |
| SCI_1d | GO:0000423 | mitophagy | 7 | 1.76077470104021e-06 | Eif2s1/Fbxw7/Bnip3/Gabarapl1/Ulk1/Gabarapl2/Tp53 |
| SCI_1d | GO:0000302 | response to reactive oxygen species | 10 | 2.50414768250378e-06 | Mapk9/Hmox1/Bnip3/Mapk8/Jun/Gch1/Tp53/Hspb1/Ripk1/Mt3 |
| SCI_1d | GO:0000422 | autophagy of mitochondrion | 7 | 4.85866451028973e-06 | Eif2s1/Fbxw7/Bnip3/Gabarapl1/Ulk1/Gabarapl2/Tp53 |
| SCI_1d | GO:0008630 | intrinsic apoptotic signaling pathway in response to DNA damage | 7 | 6.2100811456109e-06 | Cd44/Hmox1/Bid/Tp53/Ddit4/Hic1/Pml |
| SCI_1d | GO:0060964 | regulation of miRNA-mediated gene silencing | 5 | 6.2100811456109e-06 | Stat3/Tp53/Ripk1/Zfp36/Elavl1 |
| SCI_3d | GO:0009636 | response to toxic substance | 15 | 9.55104408080443e-12 | Gpx2/Srxn1/Atf4/Asns/Txnrd1/Hif1a/Gch1/Sesn2/Cdkn1a/Ddit3/Mapk9/Tlr4/Ptgs2/Tgfbr1/Slc7a11 |
| SCI_3d | GO:0090594 | inflammatory response to wounding | 8 | 1.81525519648729e-11 | Il33/Cd44/Hmox1/Hif1a/Stat3/Mapk9/Tlr4/Il6 |
| SCI_3d | GO:0072593 | reactive oxygen species metabolic process | 12 | 1.95537251522301e-09 | Txnrd1/Hif1a/Stat3/Gch1/Sesn2/Cdkn1a/Cbs/Ripk1/Ddit4/Tlr4/Gls2/Cybb |
| SCI_3d | GO:0062197 | cellular response to chemical stress | 13 | 9.09696075165326e-09 | Eif2s1/Atf4/Txnrd1/Hif1a/Gch1/Hspb1/Sesn2/Ddit3/Ripk1/Mapk9/Ptgs2/Il6/Slc7a11 |
| SCI_3d | GO:0042594 | response to starvation | 11 | 6.02229536472149e-08 | Eif2s1/Atf4/Atf3/Asns/Jun/Plin2/Slc3a2/Zfp36/Sesn2/Cdkn1a/Ddit3 |
| SCI_3d | GO:0031669 | cellular response to nutrient levels | 11 | 1.62109183018404e-07 | Eif2s1/Atf4/Atf3/Asns/Hmox1/Jun/Plin2/Slc3a2/Sesn2/Cdkn1a/Il6 |
| SCI_3d | GO:0097193 | intrinsic apoptotic signaling pathway | 11 | 2.36941421108534e-07 | Atf4/Cd44/Chac1/Hmox1/Hif1a/Hspb1/Cdkn1a/Ddit3/Ddit4/Ptgs2/Trib3 |
| SCI_3d | GO:0071248 | cellular response to metal ion | 10 | 2.36941421108534e-07 | Eif2s1/Atf4/Enpp2/Txnrd1/Hmox1/Jun/Hif1a/Ddit3/Ptgs2/Cybb |
| SCI_3d | GO:0031960 | response to corticosteroid | 11 | 3.56088171671443e-07 | Cdo1/Asns/Hif1a/Zfp36/Cxcl2/Cdkn1a/Ddit4/Tlr4/Ptgs2/Il6/Cybb |
| SCI_3d | GO:2001233 | regulation of apoptotic signaling pathway | 12 | 3.56088171671443e-07 | Atf4/Atf3/Cd44/Hmox1/Hif1a/Hspb1/Ddit3/Ripk1/Mapk9/Tlr4/Ptgs2/Tgfbr1 |
| SCI_3d | GO:0000302 | response to reactive oxygen species | 10 | 7.38600909682936e-07 | Hmox1/Jun/Hif1a/Gch1/Hspb1/Sesn2/Ddit3/Ripk1/Mapk9/Il6 |
| SCI_3d | GO:0009267 | cellular response to starvation | 9 | 8.6213853479707e-07 | Eif2s1/Atf4/Atf3/Asns/Jun/Plin2/Slc3a2/Sesn2/Cdkn1a |
| SCI_3d | GO:0034599 | cellular response to oxidative stress | 10 | 1.04022756846446e-06 | Eif2s1/Atf4/Hif1a/Gch1/Hspb1/Sesn2/Ripk1/Mapk9/Il6/Slc7a11 |
| SCI_3d | GO:0033002 | muscle cell proliferation | 10 | 1.12607467849702e-06 | Hmox1/Jun/Hif1a/Stat3/Cdkn1a/Ddit3/Tlr4/Ptgs2/Il6/Tgfbr1 |
| SCI_3d | GO:0170039 | proteinogenic amino acid metabolic process | 8 | 1.86927671338057e-06 | Cdo1/Atf4/Asns/Txnrd1/Cbs/Psat1/Gls2/Slc7a11 |
| SCI_3d | GO:0010575 | positive regulation of vascular endothelial growth factor production | 5 | 1.87858363118791e-06 | Atf4/Hif1a/Stat3/Ptgs2/Il6 |
| SCI_3d | GO:0002246 | wound healing involved in inflammatory response | 4 | 1.87858363118791e-06 | Cd44/Hmox1/Hif1a/Tlr4 |
| SCI_3d | GO:1900017 | positive regulation of cytokine production involved in inflammatory response | 5 | 2.09476763577492e-06 | Hif1a/Stat3/Mapk9/Tlr4/Il6 |
| SCI_3d | GO:0032760 | positive regulation of tumor necrosis factor production | 7 | 3.09285179321187e-06 | Il33/Stat3/Hspb1/Ripk1/Tlr4/Il6/Cybb |
| SCI_3d | GO:0170033 | L-amino acid metabolic process | 8 | 3.09285179321187e-06 | Cdo1/Atf4/Asns/Txnrd1/Cbs/Psat1/Gls2/Slc7a11 |
| SCI_7d | GO:2001233 | regulation of apoptotic signaling pathway | 13 | 1.75061876885359e-09 | Atf3/Mapk9/Cd44/Hmox1/Tgfbr1/Vegfa/Tlr4/Nfe2l2/Fbxw7/Bid/Pml/Ripk1/Rela |
| SCI_7d | GO:0097191 | extrinsic apoptotic signaling pathway | 10 | 1.58090977845309e-08 | Atf3/Hmox1/Tgfbr1/Vegfa/Tlr4/Bid/Pml/Il33/Ripk1/Rela |
| SCI_7d | GO:2001236 | regulation of extrinsic apoptotic signaling pathway | 9 | 1.58090977845309e-08 | Atf3/Hmox1/Tgfbr1/Vegfa/Tlr4/Bid/Pml/Ripk1/Rela |
| SCI_7d | GO:0090594 | inflammatory response to wounding | 6 | 1.58090977845309e-08 | Mapk9/Cd44/Hmox1/Stat3/Tlr4/Il33 |
| SCI_7d | GO:2001235 | positive regulation of apoptotic signaling pathway | 8 | 7.41680385748632e-07 | Atf3/Mapk9/Tgfbr1/Tlr4/Fbxw7/Bid/Pml/Ripk1 |
| SCI_7d | GO:2001234 | negative regulation of apoptotic signaling pathway | 8 | 7.07069444857619e-06 | Cd44/Hmox1/Tgfbr1/Vegfa/Nfe2l2/Bid/Ripk1/Rela |
| SCI_7d | GO:0072593 | reactive oxygen species metabolic process | 8 | 7.07069444857619e-06 | Gls2/Cybb/Stat3/Tlr4/Nfe2l2/Gch1/Cdkn1a/Ripk1 |
| SCI_7d | GO:0097193 | intrinsic apoptotic signaling pathway | 8 | 3.68592852057195e-05 | Cd44/Hmox1/Nfe2l2/Fbxw7/Bid/Pml/Cdkn1a/Chac1 |
| SCI_7d | GO:2001238 | positive regulation of extrinsic apoptotic signaling pathway | 5 | 3.68592852057195e-05 | Atf3/Tlr4/Bid/Pml/Ripk1 |
| SCI_7d | GO:1901342 | regulation of vasculature development | 8 | 8.36513868075553e-05 | Cybb/Hmox1/Tgfbr1/Vegfa/Stat3/Nfe2l2/Pml/Rela |
