# Supplementary material for: Ferroptosis-associated myeloid cell heterogeneity and inflammatory amplification following spinal cord injury
Source: Front Immunol. 2026 Apr 22;17:1831161. doi: 10.3389/fimmu.2026.1831161 (PMC13143767; doi:10.3389/fimmu.2026.1831161)
Supplement: Supplementary file 1 [file DataSheet1.zip › Supplementary Table S5.docx]

| Supplementary Table S5. KEGG enrichment results of FDEGs at different time points after SCI | | | | | |
| --- | --- | --- | --- | --- | --- |
| Time | ID | Description | Count | p.adjust | geneID |
| SCI_1d | rno04137 | Mitophagy - animal | 9 | 1.65E-07 | Eif2s1/Mapk9/Bnip3/Gabarapl1/Mapk8/Ulk1/Jun/Gabarapl2/Tp53 |
| SCI_1d | rno05417 | Lipid and atherosclerosis | 10 | 5.31E-06 | Cybb/Eif2s1/Mapk9/Bid/Mapk8/Jun/Tlr4/Stat3/Tp53/Vldlr |
| SCI_1d | rno04216 | Ferroptosis | 6 | 5.31E-06 | Cybb/Sat1/Hmox1/Lpcat3/Acsl3/Tp53 |
| SCI_1d | rno04217 | Necroptosis | 9 | 6.40E-06 | Il33/Cybb/Mapk9/Bid/Mapk8/Chmp6/Tlr4/Stat3/Ripk1 |
| SCI_1d | rno05162 | Measles | 8 | 2.07E-05 | Eif2s1/Mapk9/Bid/Mapk8/Jun/Tlr4/Stat3/Tp53 |
| SCI_1d | rno05161 | Hepatitis B | 8 | 3.32E-05 | Mapk9/Tgfbr1/Bid/Mapk8/Jun/Tlr4/Stat3/Tp53 |
| SCI_1d | rno04140 | Autophagy - animal | 8 | 4.58E-05 | Eif2s1/Mapk9/Bnip3/Gabarapl1/Mapk8/Ulk1/Gabarapl2/Ddit4 |
| SCI_1d | rno04621 | NOD-like receptor signaling pathway | 8 | 5.13E-05 | Cybb/Mapk9/Gabarapl1/Mapk8/Jun/Tlr4/Gabarapl2/Ripk1 |
| SCI_1d | rno04068 | FoxO signaling pathway | 7 | 6.40E-05 | Mapk9/Tgfbr1/Bnip3/Gabarapl1/Mapk8/Gabarapl2/Stat3 |
| SCI_1d | rno04210 | Apoptosis | 7 | 7.68E-05 | Eif2s1/Mapk9/Bid/Mapk8/Jun/Tp53/Ripk1 |
| SCI_1d | rno04933 | AGE-RAGE signaling pathway in diabetic complications | 6 | 0.000146586 | Cybb/Mapk9/Tgfbr1/Mapk8/Jun/Stat3 |
| SCI_1d | rno05167 | Kaposi sarcoma-associated herpesvirus infection | 8 | 0.000149733 | Mapk9/Bid/Mapk8/Jun/Stat3/Tp53/Zfp36/Ptgs2 |
| SCI_1d | rno05169 | Epstein-Barr virus infection | 8 | 0.000183608 | Cd44/Mapk9/Bid/Mapk8/Jun/Stat3/Tp53/Ripk1 |
| SCI_1d | rno05212 | Pancreatic cancer | 5 | 0.000439927 | Mapk9/Tgfbr1/Mapk8/Stat3/Tp53 |
| SCI_1d | rno04936 | Alcoholic liver disease | 6 | 0.000584697 | Mapk9/Lpin1/Scd/Mapk8/Tlr4/Ripk1 |
| SCI_3d | rno05417 | Lipid and atherosclerosis | 11 | 2.68084616357721e-07 | Eif2s1/Atf4/Jun/Stat3/Nras/Cxcl2/Ddit3/Mapk9/Tlr4/Il6/Cybb |
| SCI_3d | rno05167 | Kaposi sarcoma-associated herpesvirus infection | 10 | 1.78381650109392e-06 | Jun/Hif1a/Stat3/Zfp36/Nras/Cxcl2/Cdkn1a/Mapk9/Ptgs2/Il6 |
| SCI_3d | rno05161 | Hepatitis B | 9 | 1.78381650109392e-06 | Atf4/Jun/Stat3/Nras/Cdkn1a/Mapk9/Tlr4/Il6/Tgfbr1 |
| SCI_3d | rno04216 | Ferroptosis | 6 | 2.11949474834156e-06 | Hmox1/Sat1/Slc3a2/Acsl4/Cybb/Slc7a11 |
| SCI_3d | rno04933 | AGE-RAGE signaling pathway in diabetic complications | 7 | 9.00392039508544e-06 | Jun/Stat3/Nras/Mapk9/Il6/Tgfbr1/Cybb |
| SCI_3d | rno04668 | TNF signaling pathway | 7 | 1.88837641455169e-05 | Atf4/Jun/Cxcl2/Ripk1/Mapk9/Ptgs2/Il6 |
| SCI_3d | rno04210 | Apoptosis | 7 | 5.34670525514185e-05 | Eif2s1/Atf4/Jun/Nras/Ddit3/Ripk1/Mapk9 |
| SCI_3d | rno04137 | Mitophagy - animal | 6 | 9.01319947233931e-05 | Eif2s1/Atf4/Jun/Hif1a/Nras/Mapk9 |
| SCI_3d | rno04659 | Th17 cell differentiation | 6 | 9.01319947233931e-05 | Jun/Hif1a/Stat3/Mapk9/Il6/Tgfbr1 |
| SCI_3d | rno04066 | HIF-1 signaling pathway | 7 | 9.01319947233931e-05 | Hmox1/Hif1a/Stat3/Cdkn1a/Tlr4/Il6/Cybb |
| SCI_3d | rno04217 | Necroptosis | 7 | 0.000161138935662961 | Il33/Stat3/Ripk1/Chmp6/Mapk9/Tlr4/Cybb |
| SCI_3d | rno05166 | Human T-cell leukemia virus 1 infection | 8 | 0.000161138935662961 | Atf4/Jun/Zfp36/Nras/Cdkn1a/Mapk9/Il6/Tgfbr1 |
| SCI_3d | rno04621 | NOD-like receptor signaling pathway | 7 | 0.000172139579154147 | Jun/Cxcl2/Ripk1/Mapk9/Tlr4/Il6/Cybb |
| SCI_3d | rno04068 | FoxO signaling pathway | 6 | 0.000294519523684601 | Stat3/Nras/Cdkn1a/Mapk9/Il6/Tgfbr1 |
| SCI_3d | rno05206 | MicroRNAs in cancer | 8 | 0.000378389470543713 | Cd44/Hmox1/Stat3/Nras/Cdkn1a/Ddit4/Ptgs2/Gls2 |
| SCI_7d | rno04933 | AGE-RAGE signaling pathway in diabetic complications | 7 | 1.6131394256765e-06 | Mapk9/Jun/Cybb/Tgfbr1/Vegfa/Stat3/Rela |
| SCI_7d | rno05161 | Hepatitis B | 8 | 1.6131394256765e-06 | Mapk9/Jun/Tgfbr1/Stat3/Tlr4/Bid/Cdkn1a/Rela |
| SCI_7d | rno04217 | Necroptosis | 8 | 1.84026926525806e-06 | Mapk9/Cybb/Stat3/Tlr4/Bid/Chmp6/Il33/Ripk1 |
| SCI_7d | rno05212 | Pancreatic cancer | 6 | 3.65370655023483e-06 | Mapk9/Tgfbr1/Vegfa/Stat3/Cdkn1a/Rela |
| SCI_7d | rno05417 | Lipid and atherosclerosis | 8 | 5.57378581584532e-06 | Mapk9/Jun/Cybb/Stat3/Tlr4/Nfe2l2/Bid/Rela |
| SCI_7d | rno05169 | Epstein-Barr virus infection | 8 | 7.17932891174902e-06 | Mapk9/Jun/Cd44/Stat3/Bid/Cdkn1a/Ripk1/Rela |
| SCI_7d | rno04066 | HIF-1 signaling pathway | 7 | 7.59479560225351e-06 | Cybb/Hmox1/Vegfa/Stat3/Tlr4/Cdkn1a/Rela |
| SCI_7d | rno04621 | NOD-like receptor signaling pathway | 7 | 1.6962113280321e-05 | Mapk9/Jun/Cybb/Tlr4/Gabarapl1/Ripk1/Rela |
| SCI_7d | rno05167 | Kaposi sarcoma-associated herpesvirus infection | 7 | 5.72682571728317e-05 | Mapk9/Jun/Vegfa/Stat3/Bid/Cdkn1a/Rela |
| SCI_7d | rno05418 | Fluid shear stress and atherosclerosis | 6 | 7.16846715515051e-05 | Mapk9/Jun/Hmox1/Vegfa/Nfe2l2/Rela |
| SCI_7d | rno05162 | Measles | 6 | 7.16846715515051e-05 | Mapk9/Jun/Stat3/Tlr4/Bid/Rela |
| SCI_7d | rno04216 | Ferroptosis | 4 | 0.000134926030369389 | Cybb/Hmox1/Sat1/Lpcat3 |
| SCI_7d | rno04620 | Toll-like receptor signaling pathway | 5 | 0.000134926030369389 | Mapk9/Jun/Tlr4/Ripk1/Rela |
| SCI_7d | rno04659 | Th17 cell differentiation | 5 | 0.000134926030369389 | Mapk9/Jun/Tgfbr1/Stat3/Rela |
| SCI_7d | rno05142 | Chagas disease | 5 | 0.000138211960820048 | Mapk9/Jun/Tgfbr1/Tlr4/Rela |
