# Supplementary material for: Ferroptosis-associated myeloid cell heterogeneity and inflammatory amplification following spinal cord injury
Source: Front Immunol. 2026 Apr 22;17:1831161. doi: 10.3389/fimmu.2026.1831161 (PMC13143767; doi:10.3389/fimmu.2026.1831161)
Supplement: Supplementary file 1 [file DataSheet1.zip › Supplementary Table S6.docx]

| Supplementary Table S6. Soft-threshold selection results for WGCNA | | | | | | |
| --- | --- | --- | --- | --- | --- | --- |
| **Power** | **ScaleFreeR2** | **Slope** | **TruncatedR2** | **MeanConnectivity** | **MedianConnectivity** | **MaxConnectivity** |
| 1 | 0.746805538 | 2.645727057 | 0.877460789 | 5708.94329 | 5972.218478 | 7841.547018 |
| 2 | 0.701677862 | 1.009934119 | 0.776648426 | 3202.281715 | 3448.624649 | 5019.321101 |
| 3 | 0.521260181 | 0.371327504 | 0.469957366 | 2145.327893 | 2231.032044 | 3747.672804 |
| 4 | 0.048461769 | 0.043655194 | -0.189136725 | 1587.303536 | 1537.221868 | 3062.681042 |
| 5 | 0.70713843 | -0.247501327 | 0.625014255 | 1249.557274 | 1106.92184 | 2820.061303 |
| 6 | 0.834173058 | -0.448352818 | 0.78688032 | 1025.53218 | 826.7023568 | 2652.710455 |
| 7 | 0.845688391 | -0.582599154 | 0.802414572 | 866.9405845 | 633.0383698 | 2521.860832 |
| 8 | 0.846846213 | -0.67823592 | 0.80706573 | 749.1155755 | 493.6742071 | 2413.307999 |
| 9 | 0.836359315 | -0.744346987 | 0.798085779 | 658.2767353 | 390.2431778 | 2319.715192 |
| 10 | 0.846262563 | -0.789607213 | 0.815480515 | 586.1721226 | 314.2413508 | 2236.921172 |
| 11 | 0.840305846 | -0.820317368 | 0.817438749 | 527.5793322 | 255.1745993 | 2162.371366 |
| 12 | 0.84140756 | -0.844294792 | 0.82826454 | 479.0411038 | 209.6359153 | 2094.389147 |
| 13 | 0.840276473 | -0.862455612 | 0.836665321 | 438.1818139 | 173.3228712 | 2031.810732 |
| 14 | 0.837232533 | -0.874297189 | 0.840563569 | 403.3171589 | 144.920287 | 1973.789835 |
| 15 | 0.839909483 | -0.883182495 | 0.853133216 | 373.220736 | 122.1672778 | 1919.686954 |
| 16 | 0.845282861 | -0.886586257 | 0.867302735 | 346.9788515 | 103.2345445 | 1869.003337 |
| 17 | 0.839084054 | -0.892217595 | 0.870615664 | 323.8970933 | 87.73543315 | 1821.339778 |
| 18 | 0.839775991 | -0.897733324 | 0.880456247 | 303.4384116 | 74.8689354 | 1776.369833 |
| 19 | 0.844549795 | -0.899198756 | 0.890908063 | 285.1810056 | 64.36682737 | 1733.821786 |
| 20 | 0.84225836 | -0.900390983 | 0.893344499 | 268.789017 | 55.80143185 | 1693.466107 |
| 21 | 0.845024536 | -0.901954955 | 0.902113293 | 253.9917175 | 48.31366338 | 1655.106471 |
| 22 | 0.851134763 | -0.898831602 | 0.911722347 | 240.5684544 | 41.99750968 | 1618.573174 |
| 23 | 0.849390098 | -0.899064259 | 0.917896858 | 228.3375826 | 36.61386479 | 1583.718192 |
| 24 | 0.849792926 | -0.899642339 | 0.924539851 | 217.1482046 | 31.98915411 | 1550.411389 |
| 25 | 0.85142492 | -0.90119352 | 0.929674728 | 206.8739226 | 28.10330851 | 1518.53758 |
| 26 | 0.853559894 | -0.899460162 | 0.932653947 | 197.4080536 | 24.78065224 | 1487.994192 |
| 27 | 0.857793851 | -0.89836571 | 0.939578318 | 188.6599238 | 21.89902483 | 1458.689396 |
| 28 | 0.858123577 | -0.899300461 | 0.944129029 | 180.5519684 | 19.51259306 | 1430.54059 |
| 29 | 0.860628454 | -0.899771756 | 0.949117837 | 173.0174399 | 17.49689334 | 1403.473151 |
| 30 | 0.859179714 | -0.898480628 | 0.951698702 | 165.9985813 | 15.61573084 | 1377.419404 |
