# Supplementary material for: Ferroptosis-associated myeloid cell heterogeneity and inflammatory amplification following spinal cord injury
Source: Front Immunol. 2026 Apr 22;17:1831161. doi: 10.3389/fimmu.2026.1831161 (PMC13143767; doi:10.3389/fimmu.2026.1831161)
Supplement: Supplementary file 1 [file DataSheet1.zip › Supplementary Table S7.docx]

| Supplementary Table S7. Module–trait correlations and gene counts of WGCNA modules | | | | | | | | |
| --- | --- | --- | --- | --- | --- | --- | --- | --- |
| **Module** | **Sham** | **SCI_1d** | **SCI_3d** | **SCI_7d** | **P_Sham** | **P_SCI_1d** | **P_SCI_3d** | **P_SCI_7d** |
| MEtan | -0.111197773 | -0.124228492 | 0.541074809 | -0.305648544 | 0.681816104 | 0.646673605 | 0.03043751 | 0.249634791 |
| MEbrown | -0.592009572 | 0.100357064 | 0.870586266 | -0.378933758 | 0.015691288 | 0.711530729 | 1.15001E-05 | 0.147779498 |
| MEmagenta | -0.083978807 | -0.071560693 | 0.49204333 | -0.336503831 | 0.757159524 | 0.792272812 | 0.052867023 | 0.202524827 |
| MEpurple | -0.045230995 | 0.007975433 | -0.33861217 | 0.375867731 | 0.867895888 | 0.976614082 | 0.199532844 | 0.151355901 |
| MElightcyan | -0.146969258 | 0.683365604 | -0.289053 | -0.247343347 | 0.587023782 | 0.003519339 | 0.277561568 | 0.355703476 |
| MEred | -0.1689931 | 0.602879295 | -0.193817533 | -0.240068661 | 0.531534655 | 0.013436193 | 0.471988945 | 0.370478065 |
| MEgreenyellow | -0.169711551 | 0.5795486 | -0.162229486 | -0.247607563 | 0.529765255 | 0.018628074 | 0.548321144 | 0.355173166 |
| MEturquoise | -0.858978947 | 0.63872784 | 0.000487029 | 0.219764077 | 2.0316E-05 | 0.007738746 | 0.99857173 | 0.413460255 |
| MEpink | -0.120021299 | 0.038174299 | -0.383771495 | 0.465618494 | 0.657947868 | 0.888374969 | 0.142253788 | 0.069123965 |
| MEyellow | -0.325115074 | 0.446219143 | -0.743589328 | 0.622485259 | 0.21918705 | 0.083184934 | 0.00096059 | 0.010016323 |
| MEgrey60 | -0.037377781 | -0.125308479 | -0.316472577 | 0.479158837 | 0.890690921 | 0.643790967 | 0.232397488 | 0.060394242 |
| MElightgreen | -0.014969776 | -0.054656401 | 0.430143011 | -0.360516833 | 0.956119046 | 0.840667073 | 0.096306695 | 0.170141073 |
| MEblack | 0.803935829 | -0.201242823 | -0.214311654 | -0.388381352 | 0.000174729 | 0.454839405 | 0.425430709 | 0.13712097 |
| MEblue | 0.417805625 | 0.42877037 | -0.340508624 | -0.506067371 | 0.107326922 | 0.097491292 | 0.196866167 | 0.045489599 |
| MEsalmon | 0.557564064 | -0.087097433 | -0.202863273 | -0.267603358 | 0.024829666 | 0.748408058 | 0.451138768 | 0.316336471 |
| MEgreen | 0.612082201 | -0.786568996 | 0.24201513 | -0.067528335 | 0.011732614 | 0.000301179 | 0.366492151 | 0.803759051 |
| MEcyan | 0.695235308 | -0.140138257 | -0.233206652 | -0.321890399 | 0.002790806 | 0.604703353 | 0.384719097 | 0.224058824 |
| MEmidnightblue | 0.742599739 | -0.07022398 | -0.285642481 | -0.386733278 | 0.000983993 | 0.796076144 | 0.283525282 | 0.138941219 |
| MEgrey | -0.156634265 | 0.001707371 | -0.127201743 | 0.282128637 | 0.562380853 | 0.994992964 | 0.638749065 | 0.289749574 |

| **ModuleColor** | **GeneCount** |
| --- | --- |
| black | 198 |
| blue | 4287 |
| brown | 2262 |
| cyan | 76 |
| green | 764 |
| greenyellow | 102 |
| grey | 33 |
| grey60 | 37 |
| lightcyan | 57 |
| lightgreen | 35 |
| magenta | 156 |
| midnightblue | 68 |
| pink | 173 |
| purple | 103 |
| red | 208 |
| salmon | 80 |
| tan | 89 |
| turquoise | 5381 |
| yellow | 1009 |
