# Supplementary material for: Ferroptosis-associated myeloid cell heterogeneity and inflammatory amplification following spinal cord injury
Source: Front Immunol. 2026 Apr 22;17:1831161. doi: 10.3389/fimmu.2026.1831161 (PMC13143767; doi:10.3389/fimmu.2026.1831161)
Supplement: Supplementary file 1 [file DataSheet1.zip › Supplementary Table S8.docx]

| Supplementary Table S8. Stage-specific ferroptosis hub genes identified by WGCNA–FerrDb intersection | | | |
| --- | --- | --- | --- |
| **TimePoint** | **Gene** | **ModuleMembership** | **GeneSignificance** |
| SCI_1d | AURKA | -0.949141211 | 0.84614509 |
| SCI_1d | BRD4 | -0.839565201 | 0.591546001 |
| SCI_1d | HIC1 | -0.888631925 | 0.596406267 |
| SCI_1d | MAPK8 | 0.950777353 | -0.752910256 |
| SCI_1d | PIK3CA | 0.903349373 | -0.683046313 |
| SCI_1d | SCD | 0.963930487 | -0.770944435 |
| SCI_1d | SQSTM1 | -0.894235319 | 0.710930308 |
| SCI_3d | ACSF2 | -0.862516762 | -0.910609594 |
| SCI_3d | ASNS | 0.842327506 | 0.936030396 |
| SCI_3d | ATF4 | 0.922694159 | 0.961490938 |
| SCI_3d | CHAC1 | 0.902455397 | 0.824619963 |
| SCI_3d | CXCL2 | 0.90610352 | 0.928490836 |
| SCI_3d | EIF2S1 | 0.879695376 | 0.617621445 |
| SCI_3d | GPX2 | 0.840040149 | 0.972868528 |
| SCI_3d | HIF1A | 0.862829294 | 0.549215013 |
| SCI_3d | IL6 | 0.87414809 | 0.81021359 |
| SCI_3d | KEAP1 | 0.840188068 | 0.634408404 |
| SCI_3d | KLHL24 | -0.898094634 | -0.85451 |
| SCI_3d | MTOR | 0.817037666 | 0.686554443 |
| SCI_3d | PANX1 | 0.889549038 | 0.81273388 |
| SCI_3d | PCK2 | 0.833226458 | 0.635234382 |
| SCI_3d | SESN2 | 0.934992152 | 0.829328052 |
| SCI_3d | SLC1A5 | 0.863666919 | 0.637020421 |
| SCI_3d | SLC2A1 | 0.872158896 | 0.815458234 |
| SCI_3d | SLC3A2 | 0.834510124 | 0.723758735 |
| SCI_3d | SNX4 | 0.882006281 | 0.700786676 |
| SCI_3d | SRXN1 | 0.971462465 | 0.78730005 |
| SCI_3d | TRIB3 | 0.85593564 | 0.796307862 |
| SCI_3d | TXNRD1 | 0.990958944 | 0.865669133 |
| SCI_3d | VDAC2 | 0.80972548 | 0.804272975 |
| SCI_3d | ZFP36 | 0.891525832 | 0.700514555 |
| SCI_7d | HRAS | -0.843614494 | -0.692709606 |
| SCI_7d | HSD17B11 | 0.924426232 | 0.572761431 |
| SCI_7d | LAMP2 | 0.910845112 | 0.768783716 |
| SCI_7d | LURAP1L | -0.954308293 | -0.760737319 |
| SCI_7d | MAP1LC3A | -0.837581003 | -0.585160015 |
| SCI_7d | NFE2L2 | 0.935106007 | 0.597399466 |
| SCI_7d | SLC2A3 | -0.926782821 | -0.513477931 |
| SCI_7d | VEGFA | -0.841060932 | -0.597293172 |
