# Supplementary material for: Ferroptosis-associated myeloid cell heterogeneity and inflammatory amplification following spinal cord injury
Source: Front Immunol. 2026 Apr 22;17:1831161. doi: 10.3389/fimmu.2026.1831161 (PMC13143767; doi:10.3389/fimmu.2026.1831161)
Supplement: Supplementary file 1 [file DataSheet1.zip › Supplementary Table S9.docx]

| Supplementary Table S9. Top 10 hub genes identified from PPI networks at 1, 3, and 7 days after SCI | | | | | | | | | | | | |
| --- | --- | --- | --- | --- | --- | --- | --- | --- | --- | --- | --- | --- |
| **Time Point** | **Gene** | **MCC** | **Degree** | **Betweenness** | **Closeness** | **BottleNeck** | **EPC** | **DMNC** | **MNC** | **EcCentricity** | **Radiality** | **Stress** |
| SCI_1d | Cybb | 0.76834 | 30.227 | 0 | 5.74 | 0.2 | 1 | 7 | 7 | 24.76667 | 0 | 1 |
| SCI_1d | Cd44 | 0.73986 | 31.178 | 50 | 5.78 | 0.2 | 1 | 9 | 9 | 25.76667 | 6.70532 | 0.86111 |
| SCI_1d | Cav1 | 0.73825 | 31.206 | 56 | 5.8 | 0.2 | 1 | 10 | 10 | 26.26667 | 7.79104 | 0.82222 |
| SCI_1d | Mapk8 | 0.73825 | 31.248 | 42 | 5.82 | 0.2 | 2 | 10 | 10 | 26.43333 | 8.43271 | 0.82222 |
| SCI_1d | Atf3 | 0.73825 | 31.391 | 296 | 5.88 | 0.2 | 1 | 10 | 11 | 27.18333 | 76.16681 | 0.67273 |
| SCI_1d | Hspb1 | 0.71599 | 31.129 | 50 | 5.84 | 0.2 | 1 | 9 | 9 | 26.18333 | 9.30868 | 0.83333 |
| SCI_1d | Mapk9 | 0.71599 | 31.089 | 38 | 5.8 | 0.2 | 1 | 9 | 9 | 25.93333 | 7.18271 | 0.83333 |
| SCI_1d | Tgfbr1 | 0.71324 | 30.051 | 0 | 5.7 | 0.2 | 1 | 6 | 6 | 24.1 | 0 | 1 |
| SCI_1d | Il33 | 0.71324 | 29.753 | 0 | 5.42 | 0.16667 | 1 | 6 | 6 | 22.65 | 0 | 1 |
| SCI_1d | Ptgs2 | 0.63094 | 31.634 | 240 | 5.92 | 0.2 | 1 | 15 | 15 | 28.93333 | 42.43135 | 0.6 |
| SCI_3d | Hspb1 | 0.83533 | 17.018 | 2 | 4.14286 | 0.25 | 1 | 9 | 9 | 24.08333 | 0.22222 | 0.97222 |
| SCI_3d | Cybb | 0.81146 | 17.182 | 6 | 4.09524 | 0.25 | 1 | 9 | 9 | 23.75 | 0.59821 | 0.94444 |
| SCI_3d | Cxcl2 | 0.78759 | 16.294 | 10 | 4.09524 | 0.25 | 1 | 9 | 9 | 23.75 | 1.07097 | 0.91667 |
| SCI_3d | Il33 | 0.76834 | 13.911 | 0 | 4.02381 | 0.25 | 1 | 7 | 7 | 22.58333 | 0 | 1 |
| SCI_3d | Mapk9 | 0.73986 | 16.537 | 36 | 4.14286 | 0.25 | 1 | 9 | 9 | 24.08333 | 3.3903 | 0.86111 |
| SCI_3d | Tgfbr1 | 0.71324 | 13.77 | 0 | 3.95238 | 0.25 | 1 | 6 | 6 | 21.83333 | 0 | 1 |
| SCI_3d | Tlr4 | 0.62439 | 20.67 | 380 | 4.45238 | 0.33333 | 3 | 18 | 18 | 29.16667 | 61.46773 | 0.55556 |
| SCI_3d | Cd44 | 0.61936 | 19.192 | 342 | 4.38095 | 0.33333 | 4 | 14 | 14 | 27.33333 | 42.93448 | 0.6044 |
| SCI_3d | Nras | 0.61814 | 12.826 | 4 | 3.90476 | 0.25 | 1 | 6 | 6 | 21.58333 | 0.50794 | 0.86667 |
| SCI_3d | Cdkn1a | 0.61467 | 17.93 | 194 | 4.2619 | 0.25 | 1 | 12 | 12 | 25.91667 | 28.81844 | 0.63636 |
| SCI_7d | Cybb | 0.76834 | 7.095 | 0 | 4.33333 | 0.25 | 1 | 7 | 7 | 14.75 | 0 | 1 |
| SCI_7d | Mapk9 | 0.76834 | 6.897 | 98 | 4.45833 | 0.25 | 2 | 7 | 8 | 15.58333 | 25.47273 | 0.75 |
| SCI_7d | Hmox1 | 0.76356 | 8.241 | 36 | 4.58333 | 0.25 | 1 | 11 | 11 | 17.08333 | 5.00736 | 0.81818 |
| SCI_7d | Atf3 | 0.75809 | 7.303 | 112 | 4.5 | 0.25 | 2 | 8 | 9 | 16.08333 | 47.03333 | 0.72222 |
| SCI_7d | Nfe2l2 | 0.71711 | 8.781 | 432 | 4.79167 | 0.33333 | 5 | 12 | 14 | 18.83333 | 168.98615 | 0.54945 |
| SCI_7d | Rela | 0.71711 | 8.712 | 60 | 4.625 | 0.25 | 1 | 12 | 12 | 17.58333 | 8.98615 | 0.74242 |
| SCI_7d | Cd44 | 0.69834 | 8.03 | 46 | 4.45833 | 0.25 | 1 | 10 | 10 | 16.25 | 7.5 | 0.77778 |
| SCI_7d | Vegfa | 0.69569 | 7.973 | 54 | 4.5 | 0.25 | 1 | 11 | 11 | 16.75 | 9.11905 | 0.74545 |
| SCI_7d | Tgfbr1 | 0.64826 | 5.846 | 0 | 4.08333 | 0.2 | 1 | 5 | 5 | 13.28333 | 0 | 1 |
| SCI_7d | Ripk1 | 0.64826 | 6.059 | 82 | 4.33333 | 0.25 | 1 | 5 | 6 | 14.41667 | 18.52727 | 0.66667 |
