# Supplementary material for: Ferroptosis-associated myeloid cell heterogeneity and inflammatory amplification following spinal cord injury
Source: Front Immunol. 2026 Apr 22;17:1831161. doi: 10.3389/fimmu.2026.1831161 (PMC13143767; doi:10.3389/fimmu.2026.1831161)
Supplement: Supplementary file 1 [file DataSheet1.zip › Supplementary_Table_S15.docx]

# Supplementary Table S15 . Immune cell correlation matrix (all samples)

|  | **Activated B cell** | **Activated CD4 T cell** | **Activated CD8 T cell** | **Activated dendritic cell** | **CD56bright natural killer cell** | **CD56dim natural killer cell** | **Central memory CD4 T cell** | **Central memory CD8 T cell** | **Effector memeory CD4 T cell** | **Effector memeory CD8 T cell** | **Eosinophil** | **Gamma delta T cell** | **Immature B cell** | **Immature dendritic cell** | **Macrophage** | **Mast cell** | **MDSC** | **Memory B cell** | **Monocyte** | **Natural killer cell** | **Natural killer T cell** | **Neutrophil** | **Plasmacytoid dendritic cell** | **Regulatory T cell** | **T follicular helper cell** | **Type 1 T helper cell** | **Type 17 T helper cell** | **Type 2 T helper cell** |
| --- | --- | --- | --- | --- | --- | --- | --- | --- | --- | --- | --- | --- | --- | --- | --- | --- | --- | --- | --- | --- | --- | --- | --- | --- | --- | --- | --- | --- |
| Activated B cell | 1 | 0.344118 | 0.6 | 0.255882 | 0.126471 | -0.017647 | 0.152941 | 0.282353 | -0.047059 | 0.447059 | 0.126471 | 0.529412 | 0.5 | 0.258824 | 0.447059 | 0.935294 | 0.261765 | 0.258824 | 0.394118 | 0.320588 | 0.570588 | 0.144118 | 0.305882 | 0.305882 | 0.385294 | 0.641176 | 0.470588 | -0.585294 |
| Activated CD4 T cell | 0.344118 | 1 | 0.729412 | 0.844118 | 0.011765 | -0.261765 | 0.747059 | 0.735294 | 0.208824 | 0.402941 | -0.223529 | 0.808824 | 0.644118 | 0.294118 | 0.461765 | 0.25 | 0.847059 | 0.767647 | 0.367647 | 0.688235 | 0.691176 | -0.085294 | 0.529412 | 0.664706 | 0.441176 | 0.705882 | -0.170588 | 0.020588 |
| Activated CD8 T cell | 0.6 | 0.729412 | 1 | 0.608824 | 0.132353 | -0.041176 | 0.297059 | 0.408824 | -0.176471 | 0.444118 | 0.164706 | 0.602941 | 0.6 | -0.008824 | 0.429412 | 0.588235 | 0.582353 | 0.532353 | 0.211765 | 0.491176 | 0.820588 | 0.167647 | 0.202941 | 0.447059 | 0.294118 | 0.655882 | 0.305882 | -0.420588 |
| Activated dendritic cell | 0.255882 | 0.844118 | 0.608824 | 1 | 0.023529 | -0.255882 | 0.861765 | 0.823529 | 0.4 | 0.588235 | -0.385294 | 0.894118 | 0.767647 | 0.485294 | 0.732353 | 0.164706 | 0.926471 | 0.788235 | 0.597059 | 0.855882 | 0.729412 | -0.176471 | 0.670588 | 0.858824 | 0.679412 | 0.741176 | -0.264706 | -0.135294 |
| CD56bright natural killer cell | 0.126471 | 0.011765 | 0.132353 | 0.023529 | 1 | 0.379412 | 0.032353 | 0.182353 | 0.258824 | -0.208824 | 0.479412 | 0.020588 | 0.376471 | 0.126471 | -0.1 | 0.232353 | -0.117647 | -0.064706 | 0.238235 | -0.041176 | 0.264706 | 0.438235 | 0.117647 | 0.176471 | 0.267647 | 0.105882 | -0.108824 | -0.447059 |
| CD56dim natural killer cell | -0.017647 | -0.261765 | -0.041176 | -0.255882 | 0.379412 | 1 | -0.091176 | -0.238235 | 0.347059 | -0.273529 | 0.329412 | -0.147059 | 0.085294 | 0.014706 | -0.014706 | 0.067647 | -0.188235 | -0.085294 | 0.05 | 0.005882 | 0.138235 | 0.032353 | 0.005882 | 0.058824 | 0.135294 | 0.058824 | -0.026471 | -0.073529 |
| Central memory CD4 T cell | 0.152941 | 0.747059 | 0.297059 | 0.861765 | 0.032353 | -0.091176 | 1 | 0.852941 | 0.726471 | 0.370588 | -0.455882 | 0.847059 | 0.717647 | 0.717647 | 0.697059 | 0.044118 | 0.861765 | 0.720588 | 0.688235 | 0.814706 | 0.529412 | -0.317647 | 0.832353 | 0.882353 | 0.752941 | 0.691176 | -0.561765 | 0.111765 |
| Central memory CD8 T cell | 0.282353 | 0.735294 | 0.408824 | 0.823529 | 0.182353 | -0.238235 | 0.852941 | 1 | 0.55 | 0.370588 | -0.208824 | 0.758824 | 0.785294 | 0.714706 | 0.682353 | 0.214706 | 0.802941 | 0.514706 | 0.782353 | 0.744118 | 0.579412 | -0.323529 | 0.826471 | 0.847059 | 0.770588 | 0.623529 | -0.391176 | -0.185294 |
| Effector memeory CD4 T cell | -0.047059 | 0.208824 | -0.176471 | 0.4 | 0.258824 | 0.347059 | 0.726471 | 0.55 | 1 | 0.008824 | -0.214706 | 0.45 | 0.479412 | 0.820588 | 0.479412 | -0.094118 | 0.470588 | 0.355882 | 0.720588 | 0.552941 | 0.191176 | -0.488235 | 0.805882 | 0.679412 | 0.685294 | 0.367647 | -0.647059 | 0.082353 |
| Effector memeory CD8 T cell | 0.447059 | 0.402941 | 0.444118 | 0.588235 | -0.208824 | -0.273529 | 0.370588 | 0.370588 | 0.008824 | 1 | -0.197059 | 0.555882 | 0.491176 | 0.364706 | 0.538235 | 0.391176 | 0.55 | 0.473529 | 0.417647 | 0.585294 | 0.629412 | -0.011765 | 0.402941 | 0.476471 | 0.364706 | 0.623529 | 0.264706 | -0.444118 |
| Eosinophil | 0.126471 | -0.223529 | 0.164706 | -0.385294 | 0.479412 | 0.329412 | -0.455882 | -0.208824 | -0.214706 | -0.197059 | 1 | -0.382353 | -0.052941 | -0.176471 | -0.297059 | 0.226471 | -0.282353 | -0.255882 | -0.120588 | -0.314706 | 0.067647 | 0.232353 | -0.126471 | -0.2 | -0.270588 | -0.244118 | 0.220588 | -0.417647 |
| Gamma delta T cell | 0.529412 | 0.808824 | 0.602941 | 0.894118 | 0.020588 | -0.147059 | 0.847059 | 0.758824 | 0.45 | 0.555882 | -0.382353 | 1 | 0.826471 | 0.570588 | 0.767647 | 0.444118 | 0.879412 | 0.805882 | 0.661765 | 0.894118 | 0.764706 | -0.158824 | 0.723529 | 0.867647 | 0.785294 | 0.841176 | -0.229412 | -0.147059 |
| Immature B cell | 0.5 | 0.644118 | 0.6 | 0.767647 | 0.376471 | 0.085294 | 0.717647 | 0.785294 | 0.479412 | 0.491176 | -0.052941 | 0.826471 | 1 | 0.629412 | 0.726471 | 0.567647 | 0.761765 | 0.576471 | 0.767647 | 0.841176 | 0.820588 | 0.002941 | 0.726471 | 0.888235 | 0.867647 | 0.835294 | -0.164706 | -0.388235 |
| Immature dendritic cell | 0.258824 | 0.294118 | -0.008824 | 0.485294 | 0.126471 | 0.014706 | 0.717647 | 0.714706 | 0.820588 | 0.364706 | -0.176471 | 0.570588 | 0.629412 | 1 | 0.626471 | 0.220588 | 0.573529 | 0.367647 | 0.914706 | 0.667647 | 0.376471 | -0.514706 | 0.923529 | 0.717647 | 0.776471 | 0.538235 | -0.455882 | -0.158824 |
| Macrophage | 0.447059 | 0.461765 | 0.429412 | 0.732353 | -0.1 | -0.014706 | 0.697059 | 0.682353 | 0.479412 | 0.538235 | -0.297059 | 0.767647 | 0.726471 | 0.626471 | 1 | 0.352941 | 0.747059 | 0.467647 | 0.791176 | 0.779412 | 0.597059 | -0.338235 | 0.673529 | 0.826471 | 0.826471 | 0.661765 | -0.202941 | -0.244118 |
| Mast cell | 0.935294 | 0.25 | 0.588235 | 0.164706 | 0.232353 | 0.067647 | 0.044118 | 0.214706 | -0.094118 | 0.391176 | 0.226471 | 0.444118 | 0.567647 | 0.220588 | 0.352941 | 1 | 0.197059 | 0.158824 | 0.358824 | 0.311765 | 0.567647 | 0.235294 | 0.244118 | 0.279412 | 0.391176 | 0.614706 | 0.514706 | -0.638235 |
| MDSC | 0.261765 | 0.847059 | 0.582353 | 0.926471 | -0.117647 | -0.188235 | 0.861765 | 0.802941 | 0.470588 | 0.55 | -0.282353 | 0.879412 | 0.761765 | 0.573529 | 0.747059 | 0.197059 | 1 | 0.808824 | 0.632353 | 0.917647 | 0.691176 | -0.367647 | 0.773529 | 0.885294 | 0.664706 | 0.738235 | -0.297059 | -0.032353 |
| Memory B cell | 0.258824 | 0.767647 | 0.532353 | 0.788235 | -0.064706 | -0.085294 | 0.720588 | 0.514706 | 0.355882 | 0.473529 | -0.255882 | 0.805882 | 0.576471 | 0.367647 | 0.467647 | 0.158824 | 0.808824 | 1 | 0.364706 | 0.785294 | 0.694118 | -0.1 | 0.585294 | 0.641176 | 0.420588 | 0.738235 | -0.211765 | 0.117647 |
| Monocyte | 0.394118 | 0.367647 | 0.211765 | 0.597059 | 0.238235 | 0.05 | 0.688235 | 0.782353 | 0.720588 | 0.417647 | -0.120588 | 0.661765 | 0.767647 | 0.914706 | 0.791176 | 0.358824 | 0.632353 | 0.364706 | 1 | 0.744118 | 0.561765 | -0.447059 | 0.867647 | 0.802941 | 0.905882 | 0.605882 | -0.352941 | -0.332353 |
| Natural killer cell | 0.320588 | 0.688235 | 0.491176 | 0.855882 | -0.041176 | 0.005882 | 0.814706 | 0.744118 | 0.552941 | 0.585294 | -0.314706 | 0.894118 | 0.841176 | 0.667647 | 0.779412 | 0.311765 | 0.917647 | 0.785294 | 0.744118 | 1 | 0.767647 | -0.364706 | 0.802941 | 0.905882 | 0.814706 | 0.797059 | -0.273529 | -0.114706 |
| Natural killer T cell | 0.570588 | 0.691176 | 0.820588 | 0.729412 | 0.264706 | 0.138235 | 0.529412 | 0.579412 | 0.191176 | 0.629412 | 0.067647 | 0.764706 | 0.820588 | 0.376471 | 0.597059 | 0.567647 | 0.691176 | 0.694118 | 0.561765 | 0.767647 | 1 | 0.064706 | 0.511765 | 0.688235 | 0.626471 | 0.826471 | 0.052941 | -0.429412 |
| Neutrophil | 0.144118 | -0.085294 | 0.167647 | -0.176471 | 0.438235 | 0.032353 | -0.317647 | -0.323529 | -0.488235 | -0.011765 | 0.232353 | -0.158824 | 0.002941 | -0.514706 | -0.338235 | 0.235294 | -0.367647 | -0.1 | -0.447059 | -0.364706 | 0.064706 | 1 | -0.517647 | -0.297059 | -0.276471 | 0.023529 | 0.329412 | -0.155882 |
| Plasmacytoid dendritic cell | 0.305882 | 0.529412 | 0.202941 | 0.670588 | 0.117647 | 0.005882 | 0.832353 | 0.826471 | 0.805882 | 0.402941 | -0.126471 | 0.723529 | 0.726471 | 0.923529 | 0.673529 | 0.244118 | 0.773529 | 0.585294 | 0.867647 | 0.802941 | 0.511765 | -0.517647 | 1 | 0.858824 | 0.773529 | 0.641176 | -0.411765 | -0.176471 |
| Regulatory T cell | 0.305882 | 0.664706 | 0.447059 | 0.858824 | 0.176471 | 0.058824 | 0.882353 | 0.847059 | 0.679412 | 0.476471 | -0.2 | 0.867647 | 0.888235 | 0.717647 | 0.826471 | 0.279412 | 0.885294 | 0.641176 | 0.802941 | 0.905882 | 0.688235 | -0.297059 | 0.858824 | 1 | 0.885294 | 0.714706 | -0.4 | -0.229412 |
| T follicular helper cell | 0.385294 | 0.441176 | 0.294118 | 0.679412 | 0.267647 | 0.135294 | 0.752941 | 0.770588 | 0.685294 | 0.364706 | -0.270588 | 0.785294 | 0.867647 | 0.776471 | 0.826471 | 0.391176 | 0.664706 | 0.420588 | 0.905882 | 0.814706 | 0.626471 | -0.276471 | 0.773529 | 0.885294 | 1 | 0.661765 | -0.4 | -0.267647 |
| Type 1 T helper cell | 0.641176 | 0.705882 | 0.655882 | 0.741176 | 0.105882 | 0.058824 | 0.691176 | 0.623529 | 0.367647 | 0.623529 | -0.244118 | 0.841176 | 0.835294 | 0.538235 | 0.661765 | 0.614706 | 0.738235 | 0.738235 | 0.605882 | 0.797059 | 0.826471 | 0.023529 | 0.641176 | 0.714706 | 0.661765 | 1 | 0.091176 | -0.258824 |
| Type 17 T helper cell | 0.470588 | -0.170588 | 0.305882 | -0.264706 | -0.108824 | -0.026471 | -0.561765 | -0.391176 | -0.647059 | 0.264706 | 0.220588 | -0.229412 | -0.164706 | -0.455882 | -0.202941 | 0.514706 | -0.297059 | -0.211765 | -0.352941 | -0.273529 | 0.052941 | 0.329412 | -0.411765 | -0.4 | -0.4 | 0.091176 | 1 | -0.511765 |
| Type 2 T helper cell | -0.585294 | 0.020588 | -0.420588 | -0.135294 | -0.447059 | -0.073529 | 0.111765 | -0.185294 | 0.082353 | -0.444118 | -0.417647 | -0.147059 | -0.388235 | -0.158824 | -0.244118 | -0.638235 | -0.032353 | 0.117647 | -0.332353 | -0.114706 | -0.429412 | -0.155882 | -0.176471 | -0.229412 | -0.267647 | -0.258824 | -0.511765 | 1 |

# Supplementary Table S15. P-value matrix for immune cell correlations (all samples)

|  | **Activated B cell** | **Activated CD4 T cell** | **Activated CD8 T cell** | **Activated dendritic cell** | **CD56bright natural killer cell** | **CD56dim natural killer cell** | **Central memory CD4 T cell** | **Central memory CD8 T cell** | **Effector memeory CD4 T cell** | **Effector memeory CD8 T cell** | **Eosinophil** | **Gamma delta T cell** | **Immature B cell** | **Immature dendritic cell** | **Macrophage** | **Mast cell** | **MDSC** | **Memory B cell** | **Monocyte** | **Natural killer cell** | **Natural killer T cell** | **Neutrophil** | **Plasmacytoid dendritic cell** | **Regulatory T cell** | **T follicular helper cell** | **Type 1 T helper cell** | **Type 17 T helper cell** | **Type 2 T helper cell** |
| --- | --- | --- | --- | --- | --- | --- | --- | --- | --- | --- | --- | --- | --- | --- | --- | --- | --- | --- | --- | --- | --- | --- | --- | --- | --- | --- | --- | --- |
| Activated B cell | 0 | 0.191942 | 0.01597 | 0.337574 | 0.640554 | 0.951949 | 0.57102 | 0.288413 | 0.865106 | 0.084373 | 0.640554 | 0.037277 | 0.050852 | 0.33188 | 0.084373 | 0 | 0.326245 | 0.33188 | 0.131937 | 0.225701 | 0.023176 | 0.593829 | 0.248661 | 0.248661 | 0.141409 | 0.008975 | 0.067902 | 0.01931 |
| Activated CD4 T cell | 0.191942 | 0 | 0.001927 | 1.900E-05 | 0.969412 | 0.326245 | 0.001314 | 0.001703 | 0.436322 | 0.122925 | 0.403941 | 2.200E-04 | 0.00859 | 0.268072 | 0.073768 | 0.349132 | 1.000E-05 | 7.980E-04 | 0.161766 | 0.00421 | 0.003999 | 0.75456 | 0.037277 | 0.006241 | 0.088915 | 0.003062 | 0.526599 | 0.943224 |
| Activated CD8 T cell | 0.01597 | 0.001927 | 0 | 0.014196 | 0.624821 | 0.882386 | 0.263132 | 0.117166 | 0.512164 | 0.086622 | 0.541224 | 0.01536 | 0.01597 | 0.978149 | 0.098537 | 0.018602 | 0.02004 | 0.036091 | 0.429738 | 0.05558 | 1.310E-04 | 0.533888 | 0.449649 | 0.084373 | 0.268072 | 0.007175 | 0.248661 | 0.106235 |
| Activated dendritic cell | 0.337574 | 1.900E-05 | 0.014196 | 0 | 0.934507 | 0.337574 | 0 | 1.130E-04 | 0.125879 | 0.018602 | 0.141409 | 0 | 7.980E-04 | 0.058912 | 0.001812 | 0.541224 | 0 | 4.470E-04 | 0.016599 | 0 | 0.001927 | 0.512164 | 0.005673 | 0 | 0.004899 | 0.001499 | 0.320666 | 0.617013 |
| CD56bright natural killer cell | 0.640554 | 0.969412 | 0.624821 | 0.934507 | 0 | 0.147983 | 0.908401 | 0.497921 | 0.33188 | 0.436322 | 0.062393 | 0.943224 | 0.151349 | 0.640554 | 0.71313 | 0.385168 | 0.664435 | 0.813651 | 0.372931 | 0.882386 | 0.320666 | 0.091253 | 0.664435 | 0.512164 | 0.315146 | 0.696767 | 0.688634 | 0.084373 |
| CD56dim natural killer cell | 0.951949 | 0.326245 | 0.882386 | 0.337574 | 0.147983 | 0 | 0.737903 | 0.372931 | 0.187976 | 0.304279 | 0.212614 | 0.586183 | 0.75456 | 0.960678 | 0.960678 | 0.805142 | 0.483876 | 0.75456 | 0.856487 | 0.986888 | 0.609244 | 0.908401 | 0.986888 | 0.830731 | 0.617013 | 0.830731 | 0.925796 | 0.788187 |
| Central memory CD4 T cell | 0.57102 | 0.001314 | 0.263132 | 0 | 0.908401 | 0.737903 | 0 | 0 | 0.002048 | 0.15824 | 0.077883 | 1.000E-05 | 0.002444 | 0.002444 | 0.0036 | 0.873739 | 0 | 0.002306 | 0.00421 | 1.720E-04 | 0.037277 | 0.230177 | 6.600E-05 | 0 | 0.001147 | 0.003999 | 0.025771 | 0.680533 |
| Central memory CD8 T cell | 0.288413 | 0.001703 | 0.117166 | 1.130E-04 | 0.497921 | 0.372931 | 0 | 0 | 0.029578 | 0.15824 | 0.436322 | 9.960E-04 | 4.890E-04 | 0.002588 | 0.00466 | 0.423208 | 2.740E-04 | 0.043661 | 5.330E-04 | 0.001404 | 0.020791 | 0.221281 | 9.600E-05 | 1.000E-05 | 7.390E-04 | 0.011589 | 0.135043 | 0.490874 |
| Effector memeory CD4 T cell | 0.865106 | 0.436322 | 0.512164 | 0.125879 | 0.33188 | 0.187976 | 0.002048 | 0.029578 | 0 | 0.978149 | 0.423208 | 0.082167 | 0.062393 | 1.310E-04 | 0.062393 | 0.729616 | 0.067902 | 0.176413 | 0.002306 | 0.028588 | 0.476928 | 0.057228 | 2.460E-04 | 0.004899 | 0.004431 | 0.161766 | 0.008217 | 0.762929 |
| Effector memeory CD8 T cell | 0.084373 | 0.122925 | 0.086622 | 0.018602 | 0.436322 | 0.304279 | 0.15824 | 0.15824 | 0.978149 | 0 | 0.463185 | 0.027623 | 0.05558 | 0.165346 | 0.033808 | 0.135043 | 0.029578 | 0.066027 | 0.108896 | 0.01931 | 0.010658 | 0.969412 | 0.122925 | 0.064191 | 0.165346 | 0.011589 | 0.320666 | 0.086622 |
| Eosinophil | 0.640554 | 0.403941 | 0.541224 | 0.141409 | 0.062393 | 0.212614 | 0.077883 | 0.436322 | 0.423208 | 0.463185 | 0 | 0.14467 | 0.847885 | 0.512164 | 0.263132 | 0.397628 | 0.288413 | 0.337574 | 0.656438 | 0.234712 | 0.805142 | 0.385168 | 0.640554 | 0.456391 | 0.309683 | 0.360918 | 0.410309 | 0.108896 |
| Gamma delta T cell | 0.037277 | 2.200E-04 | 0.01536 | 0 | 0.943224 | 0.586183 | 1.000E-05 | 9.960E-04 | 0.082167 | 0.027623 | 0.14467 | 0 | 9.600E-05 | 0.023176 | 7.980E-04 | 0.086622 | 0 | 2.460E-04 | 0.006541 | 0 | 8.600E-04 | 0.556032 | 0.002174 | 0 | 4.890E-04 | 2.900E-05 | 0.39137 | 0.586183 |
| Immature B cell | 0.050852 | 0.00859 | 0.01597 | 7.980E-04 | 0.151349 | 0.75456 | 0.002444 | 4.890E-04 | 0.062393 | 0.05558 | 0.847885 | 9.600E-05 | 0 | 0.010658 | 0.002048 | 0.024018 | 9.260E-04 | 0.021563 | 7.980E-04 | 2.900E-05 | 1.310E-04 | 0.995629 | 0.002048 | 0 | 0 | 5.200E-05 | 0.541224 | 0.1382 |
| Immature dendritic cell | 0.33188 | 0.268072 | 0.978149 | 0.058912 | 0.640554 | 0.960678 | 0.002444 | 0.002588 | 1.310E-04 | 0.165346 | 0.512164 | 0.023176 | 0.010658 | 0 | 0.011116 | 0.410309 | 0.022358 | 0.161766 | 0 | 0.005951 | 0.151349 | 0.043661 | 0 | 0.002444 | 6.300E-04 | 0.033808 | 0.077883 | 0.556032 |
| Macrophage | 0.084373 | 0.073768 | 0.098537 | 0.001812 | 0.71313 | 0.960678 | 0.0036 | 0.00466 | 0.062393 | 0.033808 | 0.263132 | 7.980E-04 | 0.002048 | 0.011116 | 0 | 0.180212 | 0.001314 | 0.069817 | 4.080E-04 | 5.800E-04 | 0.016599 | 0.200041 | 0.005405 | 9.600E-05 | 9.600E-05 | 0.006541 | 0.449649 | 0.360918 |
| Mast cell | 0 | 0.349132 | 0.018602 | 0.541224 | 0.385168 | 0.805142 | 0.873739 | 0.423208 | 0.729616 | 0.135043 | 0.397628 | 0.086622 | 0.024018 | 0.410309 | 0.180212 | 0 | 0.463185 | 0.556032 | 0.17267 | 0.239304 | 0.024018 | 0.379021 | 0.360918 | 0.293643 | 0.135043 | 0.013103 | 0.043661 | 0.009375 |
| MDSC | 0.326245 | 1.000E-05 | 0.02004 | 0 | 0.664435 | 0.483876 | 0 | 2.740E-04 | 0.067902 | 0.029578 | 0.288413 | 0 | 9.260E-04 | 0.022358 | 0.001314 | 0.463185 | 0 | 2.200E-04 | 0.010216 | 0 | 0.003999 | 0.161766 | 6.830E-04 | 0 | 0.006241 | 0.001598 | 0.263132 | 0.908401 |
| Memory B cell | 0.33188 | 7.980E-04 | 0.036091 | 4.470E-04 | 0.813651 | 0.75456 | 0.002306 | 0.043661 | 0.176413 | 0.066027 | 0.337574 | 2.460E-04 | 0.021563 | 0.161766 | 0.069817 | 0.556032 | 2.200E-04 | 0 | 0.165346 | 4.890E-04 | 0.003796 | 0.71313 | 0.01931 | 0.008975 | 0.106235 | 0.001598 | 0.429738 | 0.664435 |
| Monocyte | 0.131937 | 0.161766 | 0.429738 | 0.016599 | 0.372931 | 0.856487 | 0.00421 | 5.330E-04 | 0.002306 | 0.108896 | 0.656438 | 0.006541 | 7.980E-04 | 0 | 4.080E-04 | 0.17267 | 0.010216 | 0.165346 | 0 | 0.001404 | 0.025771 | 0.084373 | 0 | 2.740E-04 | 0 | 0.014769 | 0.180212 | 0.208367 |
| Natural killer cell | 0.225701 | 0.00421 | 0.05558 | 0 | 0.882386 | 0.986888 | 1.720E-04 | 0.001404 | 0.028588 | 0.01931 | 0.234712 | 0 | 2.900E-05 | 0.005951 | 5.800E-04 | 0.239304 | 0 | 4.890E-04 | 0.001404 | 0 | 7.980E-04 | 0.165346 | 2.740E-04 | 0 | 1.720E-04 | 3.370E-04 | 0.304279 | 0.672467 |
| Natural killer T cell | 0.023176 | 0.003999 | 1.310E-04 | 0.001927 | 0.320666 | 0.609244 | 0.037277 | 0.020791 | 0.476928 | 0.010658 | 0.805142 | 8.600E-04 | 1.310E-04 | 0.151349 | 0.016599 | 0.024018 | 0.003999 | 0.003796 | 0.025771 | 7.980E-04 | 0 | 0.813651 | 0.045033 | 0.00421 | 0.011116 | 9.600E-05 | 0.847885 | 0.098537 |
| Neutrophil | 0.593829 | 0.75456 | 0.533888 | 0.512164 | 0.091253 | 0.908401 | 0.230177 | 0.221281 | 0.057228 | 0.969412 | 0.385168 | 0.556032 | 0.995629 | 0.043661 | 0.200041 | 0.379021 | 0.161766 | 0.71313 | 0.084373 | 0.165346 | 0.813651 | 0 | 0.042322 | 0.263132 | 0.298932 | 0.934507 | 0.212614 | 0.563504 |
| Plasmacytoid dendritic cell | 0.248661 | 0.037277 | 0.449649 | 0.005673 | 0.664435 | 0.986888 | 6.600E-05 | 9.600E-05 | 2.460E-04 | 0.122925 | 0.640554 | 0.002174 | 0.002048 | 0 | 0.005405 | 0.360918 | 6.830E-04 | 0.01931 | 0 | 2.740E-04 | 0.045033 | 0.042322 | 0 | 0 | 6.830E-04 | 0.008975 | 0.114361 | 0.512164 |
| Regulatory T cell | 0.248661 | 0.006241 | 0.084373 | 0 | 0.512164 | 0.830731 | 0 | 1.000E-05 | 0.004899 | 0.064191 | 0.456391 | 0 | 0 | 0.002444 | 9.600E-05 | 0.293643 | 0 | 0.008975 | 2.740E-04 | 0 | 0.00421 | 0.263132 | 0 | 0 | 0 | 0.002588 | 0.125879 | 0.39137 |
| T follicular helper cell | 0.141409 | 0.088915 | 0.268072 | 0.004899 | 0.315146 | 0.617013 | 0.001147 | 7.390E-04 | 0.004431 | 0.165346 | 0.309683 | 4.890E-04 | 0 | 6.300E-04 | 9.600E-05 | 0.135043 | 0.006241 | 0.106235 | 0 | 1.720E-04 | 0.011116 | 0.298932 | 6.830E-04 | 0 | 0 | 0.006541 | 0.125879 | 0.315146 |
| Type 1 T helper cell | 0.008975 | 0.003062 | 0.007175 | 0.001499 | 0.696767 | 0.830731 | 0.003999 | 0.011589 | 0.161766 | 0.011589 | 0.360918 | 2.900E-05 | 5.200E-05 | 0.033808 | 0.006541 | 0.013103 | 0.001598 | 0.001598 | 0.014769 | 3.370E-04 | 9.600E-05 | 0.934507 | 0.008975 | 0.002588 | 0.006541 | 0 | 0.737903 | 0.33188 |
| Type 17 T helper cell | 0.067902 | 0.526599 | 0.248661 | 0.320666 | 0.688634 | 0.925796 | 0.025771 | 0.135043 | 0.008217 | 0.320666 | 0.410309 | 0.39137 | 0.541224 | 0.077883 | 0.449649 | 0.043661 | 0.263132 | 0.429738 | 0.180212 | 0.304279 | 0.847885 | 0.212614 | 0.114361 | 0.125879 | 0.125879 | 0.737903 | 0 | 0.045033 |
| Type 2 T helper cell | 0.01931 | 0.943224 | 0.106235 | 0.617013 | 0.084373 | 0.788187 | 0.680533 | 0.490874 | 0.762929 | 0.086622 | 0.108896 | 0.586183 | 0.1382 | 0.556032 | 0.360918 | 0.009375 | 0.908401 | 0.664435 | 0.208367 | 0.672467 | 0.098537 | 0.563504 | 0.512164 | 0.39137 | 0.315146 | 0.33188 | 0.045033 | 0 |

# Supplementary Table S15. Immune cell correlation matrix (Sham)

|  | **Activated B cell** | **Activated CD4 T cell** | **Activated CD8 T cell** | **Activated dendritic cell** | **CD56bright natural killer cell** | **CD56dim natural killer cell** | **Central memory CD4 T cell** | **Central memory CD8 T cell** | **Effector memeory CD4 T cell** | **Effector memeory CD8 T cell** | **Eosinophil** | **Gamma delta T cell** | **Immature B cell** | **Immature dendritic cell** | **Macrophage** | **Mast cell** | **MDSC** | **Memory B cell** | **Monocyte** | **Natural killer cell** | **Natural killer T cell** | **Neutrophil** | **Plasmacytoid dendritic cell** | **Regulatory T cell** | **T follicular helper cell** | **Type 1 T helper cell** | **Type 17 T helper cell** | **Type 2 T helper cell** |
| --- | --- | --- | --- | --- | --- | --- | --- | --- | --- | --- | --- | --- | --- | --- | --- | --- | --- | --- | --- | --- | --- | --- | --- | --- | --- | --- | --- | --- |
| Activated B cell | 1 | 1 | 0.8 | 0.4 | -0.2 | 0.4 | -0.4 | 0.4 | -0.8 | 0.4 | 1 | -0.8 | 0 | -0.2 | 0.4 | 0.2 | 0.2 | 1 | -0.4 | -0.8 | 1 | 0.4 | -0.2 | -0.4 | -0.8 | 0.2 | 0.8 | -0.6 |
| Activated CD4 T cell | 1 | 1 | 0.8 | 0.4 | -0.2 | 0.4 | -0.4 | 0.4 | -0.8 | 0.4 | 1 | -0.8 | 0 | -0.2 | 0.4 | 0.2 | 0.2 | 1 | -0.4 | -0.8 | 1 | 0.4 | -0.2 | -0.4 | -0.8 | 0.2 | 0.8 | -0.6 |
| Activated CD8 T cell | 0.8 | 0.8 | 1 | 0 | 0.4 | 0.8 | -0.8 | 0 | -0.4 | 0 | 0.8 | -1 | -0.4 | -0.4 | -0.2 | 0.4 | -0.4 | 0.8 | -0.8 | -1 | 0.8 | 0.2 | -0.4 | -0.8 | -1 | 0.4 | 1 | -0.8 |
| Activated dendritic cell | 0.4 | 0.4 | 0 | 1 | -0.8 | -0.6 | 0.6 | 1 | -0.2 | 1 | 0.4 | 0 | -0.2 | 0.8 | 0.4 | -0.8 | 0.8 | 0.4 | 0.6 | 0 | 0.4 | -0.4 | 0.8 | 0.6 | 0 | -0.8 | 0 | -0.4 |
| CD56bright natural killer cell | -0.2 | -0.2 | 0.4 | -0.8 | 1 | 0.8 | -0.8 | -0.8 | 0.4 | -0.8 | -0.2 | -0.4 | -0.4 | -0.6 | -0.8 | 0.6 | -1 | -0.2 | -0.8 | -0.4 | -0.2 | 0 | -0.6 | -0.8 | -0.4 | 0.6 | 0.4 | -0.2 |
| CD56dim natural killer cell | 0.4 | 0.4 | 0.8 | -0.6 | 0.8 | 1 | -1 | -0.6 | -0.2 | -0.6 | 0.4 | -0.8 | -0.2 | -0.8 | -0.4 | 0.8 | -0.8 | 0.4 | -1 | -0.8 | 0.4 | 0.4 | -0.8 | -1 | -0.8 | 0.8 | 0.8 | -0.4 |
| Central memory CD4 T cell | -0.4 | -0.4 | -0.8 | 0.6 | -0.8 | -1 | 1 | 0.6 | 0.2 | 0.6 | -0.4 | 0.8 | 0.2 | 0.8 | 0.4 | -0.8 | 0.8 | -0.4 | 1 | 0.8 | -0.4 | -0.4 | 0.8 | 1 | 0.8 | -0.8 | -0.8 | 0.4 |
| Central memory CD8 T cell | 0.4 | 0.4 | 0 | 1 | -0.8 | -0.6 | 0.6 | 1 | -0.2 | 1 | 0.4 | 0 | -0.2 | 0.8 | 0.4 | -0.8 | 0.8 | 0.4 | 0.6 | 0 | 0.4 | -0.4 | 0.8 | 0.6 | 0 | -0.8 | 0 | -0.4 |
| Effector memeory CD4 T cell | -0.8 | -0.8 | -0.4 | -0.2 | 0.4 | -0.2 | 0.2 | -0.2 | 1 | -0.2 | -0.8 | 0.4 | -0.6 | 0.4 | -0.8 | -0.4 | -0.4 | -0.8 | 0.2 | 0.4 | -0.8 | -0.8 | 0.4 | 0.2 | 0.4 | -0.4 | -0.4 | 0 |
| Effector memeory CD8 T cell | 0.4 | 0.4 | 0 | 1 | -0.8 | -0.6 | 0.6 | 1 | -0.2 | 1 | 0.4 | 0 | -0.2 | 0.8 | 0.4 | -0.8 | 0.8 | 0.4 | 0.6 | 0 | 0.4 | -0.4 | 0.8 | 0.6 | 0 | -0.8 | 0 | -0.4 |
| Eosinophil | 1 | 1 | 0.8 | 0.4 | -0.2 | 0.4 | -0.4 | 0.4 | -0.8 | 0.4 | 1 | -0.8 | 0 | -0.2 | 0.4 | 0.2 | 0.2 | 1 | -0.4 | -0.8 | 1 | 0.4 | -0.2 | -0.4 | -0.8 | 0.2 | 0.8 | -0.6 |
| Gamma delta T cell | -0.8 | -0.8 | -1 | 0 | -0.4 | -0.8 | 0.8 | 0 | 0.4 | 0 | -0.8 | 1 | 0.4 | 0.4 | 0.2 | -0.4 | 0.4 | -0.8 | 0.8 | 1 | -0.8 | -0.2 | 0.4 | 0.8 | 1 | -0.4 | -1 | 0.8 |
| Immature B cell | 0 | 0 | -0.4 | -0.2 | -0.4 | -0.2 | 0.2 | -0.2 | -0.6 | -0.2 | 0 | 0.4 | 1 | -0.4 | 0.8 | 0.4 | 0.4 | 0 | 0.2 | 0.4 | 0 | 0.8 | -0.4 | 0.2 | 0.4 | 0.4 | -0.4 | 0.8 |
| Immature dendritic cell | -0.2 | -0.2 | -0.4 | 0.8 | -0.6 | -0.8 | 0.8 | 0.8 | 0.4 | 0.8 | -0.2 | 0.4 | -0.4 | 1 | 0 | -1 | 0.6 | -0.2 | 0.8 | 0.4 | -0.2 | -0.8 | 1 | 0.8 | 0.4 | -1 | -0.4 | -0.2 |
| Macrophage | 0.4 | 0.4 | -0.2 | 0.4 | -0.8 | -0.4 | 0.4 | 0.4 | -0.8 | 0.4 | 0.4 | 0.2 | 0.8 | 0 | 1 | 0 | 0.8 | 0.4 | 0.4 | 0.2 | 0.4 | 0.6 | 0 | 0.4 | 0.2 | 0 | -0.2 | 0.4 |
| Mast cell | 0.2 | 0.2 | 0.4 | -0.8 | 0.6 | 0.8 | -0.8 | -0.8 | -0.4 | -0.8 | 0.2 | -0.4 | 0.4 | -1 | 0 | 1 | -0.6 | 0.2 | -0.8 | -0.4 | 0.2 | 0.8 | -1 | -0.8 | -0.4 | 1 | 0.4 | 0.2 |
| MDSC | 0.2 | 0.2 | -0.4 | 0.8 | -1 | -0.8 | 0.8 | 0.8 | -0.4 | 0.8 | 0.2 | 0.4 | 0.4 | 0.6 | 0.8 | -0.6 | 1 | 0.2 | 0.8 | 0.4 | 0.2 | 0 | 0.6 | 0.8 | 0.4 | -0.6 | -0.4 | 0.2 |
| Memory B cell | 1 | 1 | 0.8 | 0.4 | -0.2 | 0.4 | -0.4 | 0.4 | -0.8 | 0.4 | 1 | -0.8 | 0 | -0.2 | 0.4 | 0.2 | 0.2 | 1 | -0.4 | -0.8 | 1 | 0.4 | -0.2 | -0.4 | -0.8 | 0.2 | 0.8 | -0.6 |
| Monocyte | -0.4 | -0.4 | -0.8 | 0.6 | -0.8 | -1 | 1 | 0.6 | 0.2 | 0.6 | -0.4 | 0.8 | 0.2 | 0.8 | 0.4 | -0.8 | 0.8 | -0.4 | 1 | 0.8 | -0.4 | -0.4 | 0.8 | 1 | 0.8 | -0.8 | -0.8 | 0.4 |
| Natural killer cell | -0.8 | -0.8 | -1 | 0 | -0.4 | -0.8 | 0.8 | 0 | 0.4 | 0 | -0.8 | 1 | 0.4 | 0.4 | 0.2 | -0.4 | 0.4 | -0.8 | 0.8 | 1 | -0.8 | -0.2 | 0.4 | 0.8 | 1 | -0.4 | -1 | 0.8 |
| Natural killer T cell | 1 | 1 | 0.8 | 0.4 | -0.2 | 0.4 | -0.4 | 0.4 | -0.8 | 0.4 | 1 | -0.8 | 0 | -0.2 | 0.4 | 0.2 | 0.2 | 1 | -0.4 | -0.8 | 1 | 0.4 | -0.2 | -0.4 | -0.8 | 0.2 | 0.8 | -0.6 |
| Neutrophil | 0.4 | 0.4 | 0.2 | -0.4 | 0 | 0.4 | -0.4 | -0.4 | -0.8 | -0.4 | 0.4 | -0.2 | 0.8 | -0.8 | 0.6 | 0.8 | 0 | 0.4 | -0.4 | -0.2 | 0.4 | 1 | -0.8 | -0.4 | -0.2 | 0.8 | 0.2 | 0.4 |
| Plasmacytoid dendritic cell | -0.2 | -0.2 | -0.4 | 0.8 | -0.6 | -0.8 | 0.8 | 0.8 | 0.4 | 0.8 | -0.2 | 0.4 | -0.4 | 1 | 0 | -1 | 0.6 | -0.2 | 0.8 | 0.4 | -0.2 | -0.8 | 1 | 0.8 | 0.4 | -1 | -0.4 | -0.2 |
| Regulatory T cell | -0.4 | -0.4 | -0.8 | 0.6 | -0.8 | -1 | 1 | 0.6 | 0.2 | 0.6 | -0.4 | 0.8 | 0.2 | 0.8 | 0.4 | -0.8 | 0.8 | -0.4 | 1 | 0.8 | -0.4 | -0.4 | 0.8 | 1 | 0.8 | -0.8 | -0.8 | 0.4 |
| T follicular helper cell | -0.8 | -0.8 | -1 | 0 | -0.4 | -0.8 | 0.8 | 0 | 0.4 | 0 | -0.8 | 1 | 0.4 | 0.4 | 0.2 | -0.4 | 0.4 | -0.8 | 0.8 | 1 | -0.8 | -0.2 | 0.4 | 0.8 | 1 | -0.4 | -1 | 0.8 |
| Type 1 T helper cell | 0.2 | 0.2 | 0.4 | -0.8 | 0.6 | 0.8 | -0.8 | -0.8 | -0.4 | -0.8 | 0.2 | -0.4 | 0.4 | -1 | 0 | 1 | -0.6 | 0.2 | -0.8 | -0.4 | 0.2 | 0.8 | -1 | -0.8 | -0.4 | 1 | 0.4 | 0.2 |
| Type 17 T helper cell | 0.8 | 0.8 | 1 | 0 | 0.4 | 0.8 | -0.8 | 0 | -0.4 | 0 | 0.8 | -1 | -0.4 | -0.4 | -0.2 | 0.4 | -0.4 | 0.8 | -0.8 | -1 | 0.8 | 0.2 | -0.4 | -0.8 | -1 | 0.4 | 1 | -0.8 |
| Type 2 T helper cell | -0.6 | -0.6 | -0.8 | -0.4 | -0.2 | -0.4 | 0.4 | -0.4 | 0 | -0.4 | -0.6 | 0.8 | 0.8 | -0.2 | 0.4 | 0.2 | 0.2 | -0.6 | 0.4 | 0.8 | -0.6 | 0.4 | -0.2 | 0.4 | 0.8 | 0.2 | -0.8 | 1 |

# Supplementary Table S16 (Sheet 4). Immune cell correlation matrix (SCI_1d)

|  | **Activated B cell** | **Activated CD4 T cell** | **Activated CD8 T cell** | **Activated dendritic cell** | **CD56bright natural killer cell** | **CD56dim natural killer cell** | **Central memory CD4 T cell** | **Central memory CD8 T cell** | **Effector memeory CD4 T cell** | **Effector memeory CD8 T cell** | **Eosinophil** | **Gamma delta T cell** | **Immature B cell** | **Immature dendritic cell** | **Macrophage** | **Mast cell** | **MDSC** | **Memory B cell** | **Monocyte** | **Natural killer cell** | **Natural killer T cell** | **Neutrophil** | **Plasmacytoid dendritic cell** | **Regulatory T cell** | **T follicular helper cell** | **Type 1 T helper cell** | **Type 17 T helper cell** | **Type 2 T helper cell** |
| --- | --- | --- | --- | --- | --- | --- | --- | --- | --- | --- | --- | --- | --- | --- | --- | --- | --- | --- | --- | --- | --- | --- | --- | --- | --- | --- | --- | --- |
| Activated B cell | 1 | 0.8 | 0.4 | -1 | 0.4 | 0.8 | -0.4 | 0.4 | 0.2 | -0.8 | 0.8 | -0.2 | 0.6 | 0.4 | -0.8 | 0.8 | -0.2 | -0.8 | 0.2 | -0.4 | 0.8 | 0.4 | -0.2 | -0.2 | 0.4 | 0.4 | 0.4 | -0.4 |
| Activated CD4 T cell | 0.8 | 1 | 0 | -0.8 | -0.2 | 0.4 | 0 | 0.8 | 0.4 | -0.4 | 0.6 | 0.4 | 0.8 | 0.8 | -0.6 | 0.6 | 0.4 | -0.6 | 0.4 | 0.2 | 0.6 | 0 | 0.4 | 0.4 | 0.8 | 0.8 | 0 | 0 |
| Activated CD8 T cell | 0.4 | 0 | 1 | -0.4 | 0.4 | 0.2 | -1 | -0.6 | -0.8 | -0.2 | 0.8 | -0.8 | 0.4 | -0.6 | -0.8 | 0.8 | -0.8 | -0.8 | -0.8 | -0.4 | 0.8 | 1 | -0.8 | -0.8 | -0.6 | -0.6 | 1 | -1 |
| Activated dendritic cell | -1 | -0.8 | -0.4 | 1 | -0.4 | -0.8 | 0.4 | -0.4 | -0.2 | 0.8 | -0.8 | 0.2 | -0.6 | -0.4 | 0.8 | -0.8 | 0.2 | 0.8 | -0.2 | 0.4 | -0.8 | -0.4 | 0.2 | 0.2 | -0.4 | -0.4 | -0.4 | 0.4 |
| CD56bright natural killer cell | 0.4 | -0.2 | 0.4 | -0.4 | 1 | 0.8 | -0.4 | -0.4 | 0 | -0.8 | 0.2 | -0.8 | -0.4 | -0.4 | -0.2 | 0.2 | -0.8 | -0.2 | 0 | -1 | 0.2 | 0.4 | -0.8 | -0.8 | -0.4 | -0.4 | 0.4 | -0.4 |
| CD56dim natural killer cell | 0.8 | 0.4 | 0.2 | -0.8 | 0.8 | 1 | -0.2 | 0.2 | 0.4 | -1 | 0.4 | -0.4 | 0 | 0.2 | -0.4 | 0.4 | -0.4 | -0.4 | 0.4 | -0.8 | 0.4 | 0.2 | -0.4 | -0.4 | 0.2 | 0.2 | 0.2 | -0.2 |
| Central memory CD4 T cell | -0.4 | 0 | -1 | 0.4 | -0.4 | -0.2 | 1 | 0.6 | 0.8 | 0.2 | -0.8 | 0.8 | -0.4 | 0.6 | 0.8 | -0.8 | 0.8 | 0.8 | 0.8 | 0.4 | -0.8 | -1 | 0.8 | 0.8 | 0.6 | 0.6 | -1 | 1 |
| Central memory CD8 T cell | 0.4 | 0.8 | -0.6 | -0.4 | -0.4 | 0.2 | 0.6 | 1 | 0.8 | -0.2 | 0 | 0.8 | 0.4 | 1 | 0 | 0 | 0.8 | 0 | 0.8 | 0.4 | 0 | -0.6 | 0.8 | 0.8 | 1 | 1 | -0.6 | 0.6 |
| Effector memeory CD4 T cell | 0.2 | 0.4 | -0.8 | -0.2 | 0 | 0.4 | 0.8 | 0.8 | 1 | -0.4 | -0.4 | 0.6 | -0.2 | 0.8 | 0.4 | -0.4 | 0.6 | 0.4 | 1 | 0 | -0.4 | -0.8 | 0.6 | 0.6 | 0.8 | 0.8 | -0.8 | 0.8 |
| Effector memeory CD8 T cell | -0.8 | -0.4 | -0.2 | 0.8 | -0.8 | -1 | 0.2 | -0.2 | -0.4 | 1 | -0.4 | 0.4 | 0 | -0.2 | 0.4 | -0.4 | 0.4 | 0.4 | -0.4 | 0.8 | -0.4 | -0.2 | 0.4 | 0.4 | -0.2 | -0.2 | -0.2 | 0.2 |
| Eosinophil | 0.8 | 0.6 | 0.8 | -0.8 | 0.2 | 0.4 | -0.8 | 0 | -0.4 | -0.4 | 1 | -0.4 | 0.8 | 0 | -1 | 1 | -0.4 | -1 | -0.4 | -0.2 | 1 | 0.8 | -0.4 | -0.4 | 0 | 0 | 0.8 | -0.8 |
| Gamma delta T cell | -0.2 | 0.4 | -0.8 | 0.2 | -0.8 | -0.4 | 0.8 | 0.8 | 0.6 | 0.4 | -0.4 | 1 | 0.2 | 0.8 | 0.4 | -0.4 | 1 | 0.4 | 0.6 | 0.8 | -0.4 | -0.8 | 1 | 1 | 0.8 | 0.8 | -0.8 | 0.8 |
| Immature B cell | 0.6 | 0.8 | 0.4 | -0.6 | -0.4 | 0 | -0.4 | 0.4 | -0.2 | 0 | 0.8 | 0.2 | 1 | 0.4 | -0.8 | 0.8 | 0.2 | -0.8 | -0.2 | 0.4 | 0.8 | 0.4 | 0.2 | 0.2 | 0.4 | 0.4 | 0.4 | -0.4 |
| Immature dendritic cell | 0.4 | 0.8 | -0.6 | -0.4 | -0.4 | 0.2 | 0.6 | 1 | 0.8 | -0.2 | 0 | 0.8 | 0.4 | 1 | 0 | 0 | 0.8 | 0 | 0.8 | 0.4 | 0 | -0.6 | 0.8 | 0.8 | 1 | 1 | -0.6 | 0.6 |
| Macrophage | -0.8 | -0.6 | -0.8 | 0.8 | -0.2 | -0.4 | 0.8 | 0 | 0.4 | 0.4 | -1 | 0.4 | -0.8 | 0 | 1 | -1 | 0.4 | 1 | 0.4 | 0.2 | -1 | -0.8 | 0.4 | 0.4 | 0 | 0 | -0.8 | 0.8 |
| Mast cell | 0.8 | 0.6 | 0.8 | -0.8 | 0.2 | 0.4 | -0.8 | 0 | -0.4 | -0.4 | 1 | -0.4 | 0.8 | 0 | -1 | 1 | -0.4 | -1 | -0.4 | -0.2 | 1 | 0.8 | -0.4 | -0.4 | 0 | 0 | 0.8 | -0.8 |
| MDSC | -0.2 | 0.4 | -0.8 | 0.2 | -0.8 | -0.4 | 0.8 | 0.8 | 0.6 | 0.4 | -0.4 | 1 | 0.2 | 0.8 | 0.4 | -0.4 | 1 | 0.4 | 0.6 | 0.8 | -0.4 | -0.8 | 1 | 1 | 0.8 | 0.8 | -0.8 | 0.8 |
| Memory B cell | -0.8 | -0.6 | -0.8 | 0.8 | -0.2 | -0.4 | 0.8 | 0 | 0.4 | 0.4 | -1 | 0.4 | -0.8 | 0 | 1 | -1 | 0.4 | 1 | 0.4 | 0.2 | -1 | -0.8 | 0.4 | 0.4 | 0 | 0 | -0.8 | 0.8 |
| Monocyte | 0.2 | 0.4 | -0.8 | -0.2 | 0 | 0.4 | 0.8 | 0.8 | 1 | -0.4 | -0.4 | 0.6 | -0.2 | 0.8 | 0.4 | -0.4 | 0.6 | 0.4 | 1 | 0 | -0.4 | -0.8 | 0.6 | 0.6 | 0.8 | 0.8 | -0.8 | 0.8 |
| Natural killer cell | -0.4 | 0.2 | -0.4 | 0.4 | -1 | -0.8 | 0.4 | 0.4 | 0 | 0.8 | -0.2 | 0.8 | 0.4 | 0.4 | 0.2 | -0.2 | 0.8 | 0.2 | 0 | 1 | -0.2 | -0.4 | 0.8 | 0.8 | 0.4 | 0.4 | -0.4 | 0.4 |
| Natural killer T cell | 0.8 | 0.6 | 0.8 | -0.8 | 0.2 | 0.4 | -0.8 | 0 | -0.4 | -0.4 | 1 | -0.4 | 0.8 | 0 | -1 | 1 | -0.4 | -1 | -0.4 | -0.2 | 1 | 0.8 | -0.4 | -0.4 | 0 | 0 | 0.8 | -0.8 |
| Neutrophil | 0.4 | 0 | 1 | -0.4 | 0.4 | 0.2 | -1 | -0.6 | -0.8 | -0.2 | 0.8 | -0.8 | 0.4 | -0.6 | -0.8 | 0.8 | -0.8 | -0.8 | -0.8 | -0.4 | 0.8 | 1 | -0.8 | -0.8 | -0.6 | -0.6 | 1 | -1 |
| Plasmacytoid dendritic cell | -0.2 | 0.4 | -0.8 | 0.2 | -0.8 | -0.4 | 0.8 | 0.8 | 0.6 | 0.4 | -0.4 | 1 | 0.2 | 0.8 | 0.4 | -0.4 | 1 | 0.4 | 0.6 | 0.8 | -0.4 | -0.8 | 1 | 1 | 0.8 | 0.8 | -0.8 | 0.8 |
| Regulatory T cell | -0.2 | 0.4 | -0.8 | 0.2 | -0.8 | -0.4 | 0.8 | 0.8 | 0.6 | 0.4 | -0.4 | 1 | 0.2 | 0.8 | 0.4 | -0.4 | 1 | 0.4 | 0.6 | 0.8 | -0.4 | -0.8 | 1 | 1 | 0.8 | 0.8 | -0.8 | 0.8 |
| T follicular helper cell | 0.4 | 0.8 | -0.6 | -0.4 | -0.4 | 0.2 | 0.6 | 1 | 0.8 | -0.2 | 0 | 0.8 | 0.4 | 1 | 0 | 0 | 0.8 | 0 | 0.8 | 0.4 | 0 | -0.6 | 0.8 | 0.8 | 1 | 1 | -0.6 | 0.6 |
| Type 1 T helper cell | 0.4 | 0.8 | -0.6 | -0.4 | -0.4 | 0.2 | 0.6 | 1 | 0.8 | -0.2 | 0 | 0.8 | 0.4 | 1 | 0 | 0 | 0.8 | 0 | 0.8 | 0.4 | 0 | -0.6 | 0.8 | 0.8 | 1 | 1 | -0.6 | 0.6 |
| Type 17 T helper cell | 0.4 | 0 | 1 | -0.4 | 0.4 | 0.2 | -1 | -0.6 | -0.8 | -0.2 | 0.8 | -0.8 | 0.4 | -0.6 | -0.8 | 0.8 | -0.8 | -0.8 | -0.8 | -0.4 | 0.8 | 1 | -0.8 | -0.8 | -0.6 | -0.6 | 1 | -1 |
| Type 2 T helper cell | -0.4 | 0 | -1 | 0.4 | -0.4 | -0.2 | 1 | 0.6 | 0.8 | 0.2 | -0.8 | 0.8 | -0.4 | 0.6 | 0.8 | -0.8 | 0.8 | 0.8 | 0.8 | 0.4 | -0.8 | -1 | 0.8 | 0.8 | 0.6 | 0.6 | -1 | 1 |

# Supplementary Table S16 (Sheet 5). Immune cell correlation matrix (SCI_3d)

|  | **Activated B cell** | **Activated CD4 T cell** | **Activated CD8 T cell** | **Activated dendritic cell** | **CD56bright natural killer cell** | **CD56dim natural killer cell** | **Central memory CD4 T cell** | **Central memory CD8 T cell** | **Effector memeory CD4 T cell** | **Effector memeory CD8 T cell** | **Eosinophil** | **Gamma delta T cell** | **Immature B cell** | **Immature dendritic cell** | **Macrophage** | **Mast cell** | **MDSC** | **Memory B cell** | **Monocyte** | **Natural killer cell** | **Natural killer T cell** | **Neutrophil** | **Plasmacytoid dendritic cell** | **Regulatory T cell** | **T follicular helper cell** | **Type 1 T helper cell** | **Type 17 T helper cell** | **Type 2 T helper cell** |
| --- | --- | --- | --- | --- | --- | --- | --- | --- | --- | --- | --- | --- | --- | --- | --- | --- | --- | --- | --- | --- | --- | --- | --- | --- | --- | --- | --- | --- |
| Activated B cell | 1 | -0.8 | 0.2 | -0.6 | -0.8 | 0.6 | -0.8 | -0.6 | -1 | 0.8 | -0.4 | -0.6 | -0.8 | -0.8 | 0.4 | 0.8 | -0.8 | -0.8 | -0.6 | -0.8 | 0.2 | -0.4 | -1 | -0.8 | 0 | -0.4 | 0.6 | -0.2 |
| Activated CD4 T cell | -0.8 | 1 | 0.4 | 0.8 | 1 | -0.8 | 0.6 | 0.8 | 0.8 | -1 | 0.8 | 0.8 | 1 | 0.6 | -0.2 | -0.6 | 1 | 0.4 | 0.8 | 0.6 | 0.4 | 0.2 | 0.8 | 1 | 0.4 | -0.2 | -0.8 | -0.4 |
| Activated CD8 T cell | 0.2 | 0.4 | 1 | 0.2 | 0.4 | -0.2 | -0.4 | 0.2 | -0.2 | -0.4 | 0.8 | 0.2 | 0.4 | -0.4 | 0 | 0.4 | 0.4 | -0.4 | 0.2 | -0.4 | 1 | 0 | -0.2 | 0.4 | 0.4 | -0.8 | -0.2 | -1 |
| Activated dendritic cell | -0.6 | 0.8 | 0.2 | 1 | 0.8 | -1 | 0.8 | 1 | 0.6 | -0.8 | 0.4 | 1 | 0.8 | 0.8 | 0.4 | -0.8 | 0.8 | 0 | 1 | 0.8 | 0.2 | -0.4 | 0.6 | 0.8 | 0.8 | -0.4 | -1 | -0.2 |
| CD56bright natural killer cell | -0.8 | 1 | 0.4 | 0.8 | 1 | -0.8 | 0.6 | 0.8 | 0.8 | -1 | 0.8 | 0.8 | 1 | 0.6 | -0.2 | -0.6 | 1 | 0.4 | 0.8 | 0.6 | 0.4 | 0.2 | 0.8 | 1 | 0.4 | -0.2 | -0.8 | -0.4 |
| CD56dim natural killer cell | 0.6 | -0.8 | -0.2 | -1 | -0.8 | 1 | -0.8 | -1 | -0.6 | 0.8 | -0.4 | -1 | -0.8 | -0.8 | -0.4 | 0.8 | -0.8 | 0 | -1 | -0.8 | -0.2 | 0.4 | -0.6 | -0.8 | -0.8 | 0.4 | 1 | 0.2 |
| Central memory CD4 T cell | -0.8 | 0.6 | -0.4 | 0.8 | 0.6 | -0.8 | 1 | 0.8 | 0.8 | -0.6 | 0 | 0.8 | 0.6 | 1 | 0.2 | -1 | 0.6 | 0.4 | 0.8 | 1 | -0.4 | -0.2 | 0.8 | 0.6 | 0.4 | 0.2 | -0.8 | 0.4 |
| Central memory CD8 T cell | -0.6 | 0.8 | 0.2 | 1 | 0.8 | -1 | 0.8 | 1 | 0.6 | -0.8 | 0.4 | 1 | 0.8 | 0.8 | 0.4 | -0.8 | 0.8 | 0 | 1 | 0.8 | 0.2 | -0.4 | 0.6 | 0.8 | 0.8 | -0.4 | -1 | -0.2 |
| Effector memeory CD4 T cell | -1 | 0.8 | -0.2 | 0.6 | 0.8 | -0.6 | 0.8 | 0.6 | 1 | -0.8 | 0.4 | 0.6 | 0.8 | 0.8 | -0.4 | -0.8 | 0.8 | 0.8 | 0.6 | 0.8 | -0.2 | 0.4 | 1 | 0.8 | 0 | 0.4 | -0.6 | 0.2 |
| Effector memeory CD8 T cell | 0.8 | -1 | -0.4 | -0.8 | -1 | 0.8 | -0.6 | -0.8 | -0.8 | 1 | -0.8 | -0.8 | -1 | -0.6 | 0.2 | 0.6 | -1 | -0.4 | -0.8 | -0.6 | -0.4 | -0.2 | -0.8 | -1 | -0.4 | 0.2 | 0.8 | 0.4 |
| Eosinophil | -0.4 | 0.8 | 0.8 | 0.4 | 0.8 | -0.4 | 0 | 0.4 | 0.4 | -0.8 | 1 | 0.4 | 0.8 | 0 | -0.4 | 0 | 0.8 | 0.2 | 0.4 | 0 | 0.8 | 0.4 | 0.4 | 0.8 | 0.2 | -0.4 | -0.4 | -0.8 |
| Gamma delta T cell | -0.6 | 0.8 | 0.2 | 1 | 0.8 | -1 | 0.8 | 1 | 0.6 | -0.8 | 0.4 | 1 | 0.8 | 0.8 | 0.4 | -0.8 | 0.8 | 0 | 1 | 0.8 | 0.2 | -0.4 | 0.6 | 0.8 | 0.8 | -0.4 | -1 | -0.2 |
| Immature B cell | -0.8 | 1 | 0.4 | 0.8 | 1 | -0.8 | 0.6 | 0.8 | 0.8 | -1 | 0.8 | 0.8 | 1 | 0.6 | -0.2 | -0.6 | 1 | 0.4 | 0.8 | 0.6 | 0.4 | 0.2 | 0.8 | 1 | 0.4 | -0.2 | -0.8 | -0.4 |
| Immature dendritic cell | -0.8 | 0.6 | -0.4 | 0.8 | 0.6 | -0.8 | 1 | 0.8 | 0.8 | -0.6 | 0 | 0.8 | 0.6 | 1 | 0.2 | -1 | 0.6 | 0.4 | 0.8 | 1 | -0.4 | -0.2 | 0.8 | 0.6 | 0.4 | 0.2 | -0.8 | 0.4 |
| Macrophage | 0.4 | -0.2 | 0 | 0.4 | -0.2 | -0.4 | 0.2 | 0.4 | -0.4 | 0.2 | -0.4 | 0.4 | -0.2 | 0.2 | 1 | -0.2 | -0.2 | -0.8 | 0.4 | 0.2 | 0 | -1 | -0.4 | -0.2 | 0.8 | -0.6 | -0.4 | 0 |
| Mast cell | 0.8 | -0.6 | 0.4 | -0.8 | -0.6 | 0.8 | -1 | -0.8 | -0.8 | 0.6 | 0 | -0.8 | -0.6 | -1 | -0.2 | 1 | -0.6 | -0.4 | -0.8 | -1 | 0.4 | 0.2 | -0.8 | -0.6 | -0.4 | -0.2 | 0.8 | -0.4 |
| MDSC | -0.8 | 1 | 0.4 | 0.8 | 1 | -0.8 | 0.6 | 0.8 | 0.8 | -1 | 0.8 | 0.8 | 1 | 0.6 | -0.2 | -0.6 | 1 | 0.4 | 0.8 | 0.6 | 0.4 | 0.2 | 0.8 | 1 | 0.4 | -0.2 | -0.8 | -0.4 |
| Memory B cell | -0.8 | 0.4 | -0.4 | 0 | 0.4 | 0 | 0.4 | 0 | 0.8 | -0.4 | 0.2 | 0 | 0.4 | 0.4 | -0.8 | -0.4 | 0.4 | 1 | 0 | 0.4 | -0.4 | 0.8 | 0.8 | 0.4 | -0.6 | 0.8 | 0 | 0.4 |
| Monocyte | -0.6 | 0.8 | 0.2 | 1 | 0.8 | -1 | 0.8 | 1 | 0.6 | -0.8 | 0.4 | 1 | 0.8 | 0.8 | 0.4 | -0.8 | 0.8 | 0 | 1 | 0.8 | 0.2 | -0.4 | 0.6 | 0.8 | 0.8 | -0.4 | -1 | -0.2 |
| Natural killer cell | -0.8 | 0.6 | -0.4 | 0.8 | 0.6 | -0.8 | 1 | 0.8 | 0.8 | -0.6 | 0 | 0.8 | 0.6 | 1 | 0.2 | -1 | 0.6 | 0.4 | 0.8 | 1 | -0.4 | -0.2 | 0.8 | 0.6 | 0.4 | 0.2 | -0.8 | 0.4 |
| Natural killer T cell | 0.2 | 0.4 | 1 | 0.2 | 0.4 | -0.2 | -0.4 | 0.2 | -0.2 | -0.4 | 0.8 | 0.2 | 0.4 | -0.4 | 0 | 0.4 | 0.4 | -0.4 | 0.2 | -0.4 | 1 | 0 | -0.2 | 0.4 | 0.4 | -0.8 | -0.2 | -1 |
| Neutrophil | -0.4 | 0.2 | 0 | -0.4 | 0.2 | 0.4 | -0.2 | -0.4 | 0.4 | -0.2 | 0.4 | -0.4 | 0.2 | -0.2 | -1 | 0.2 | 0.2 | 0.8 | -0.4 | -0.2 | 0 | 1 | 0.4 | 0.2 | -0.8 | 0.6 | 0.4 | 0 |
| Plasmacytoid dendritic cell | -1 | 0.8 | -0.2 | 0.6 | 0.8 | -0.6 | 0.8 | 0.6 | 1 | -0.8 | 0.4 | 0.6 | 0.8 | 0.8 | -0.4 | -0.8 | 0.8 | 0.8 | 0.6 | 0.8 | -0.2 | 0.4 | 1 | 0.8 | 0 | 0.4 | -0.6 | 0.2 |
| Regulatory T cell | -0.8 | 1 | 0.4 | 0.8 | 1 | -0.8 | 0.6 | 0.8 | 0.8 | -1 | 0.8 | 0.8 | 1 | 0.6 | -0.2 | -0.6 | 1 | 0.4 | 0.8 | 0.6 | 0.4 | 0.2 | 0.8 | 1 | 0.4 | -0.2 | -0.8 | -0.4 |
| T follicular helper cell | 0 | 0.4 | 0.4 | 0.8 | 0.4 | -0.8 | 0.4 | 0.8 | 0 | -0.4 | 0.2 | 0.8 | 0.4 | 0.4 | 0.8 | -0.4 | 0.4 | -0.6 | 0.8 | 0.4 | 0.4 | -0.8 | 0 | 0.4 | 1 | -0.8 | -0.8 | -0.4 |
| Type 1 T helper cell | -0.4 | -0.2 | -0.8 | -0.4 | -0.2 | 0.4 | 0.2 | -0.4 | 0.4 | 0.2 | -0.4 | -0.4 | -0.2 | 0.2 | -0.6 | -0.2 | -0.2 | 0.8 | -0.4 | 0.2 | -0.8 | 0.6 | 0.4 | -0.2 | -0.8 | 1 | 0.4 | 0.8 |
| Type 17 T helper cell | 0.6 | -0.8 | -0.2 | -1 | -0.8 | 1 | -0.8 | -1 | -0.6 | 0.8 | -0.4 | -1 | -0.8 | -0.8 | -0.4 | 0.8 | -0.8 | 0 | -1 | -0.8 | -0.2 | 0.4 | -0.6 | -0.8 | -0.8 | 0.4 | 1 | 0.2 |
| Type 2 T helper cell | -0.2 | -0.4 | -1 | -0.2 | -0.4 | 0.2 | 0.4 | -0.2 | 0.2 | 0.4 | -0.8 | -0.2 | -0.4 | 0.4 | 0 | -0.4 | -0.4 | 0.4 | -0.2 | 0.4 | -1 | 0 | 0.2 | -0.4 | -0.4 | 0.8 | 0.2 | 1 |

# Supplementary Table S16 (Sheet 6). Immune cell correlation matrix (SCI_7d)

|  | **Activated B cell** | **Activated CD4 T cell** | **Activated CD8 T cell** | **Activated dendritic cell** | **CD56bright natural killer cell** | **CD56dim natural killer cell** | **Central memory CD4 T cell** | **Central memory CD8 T cell** | **Effector memeory CD4 T cell** | **Effector memeory CD8 T cell** | **Eosinophil** | **Gamma delta T cell** | **Immature B cell** | **Immature dendritic cell** | **Macrophage** | **Mast cell** | **MDSC** | **Memory B cell** | **Monocyte** | **Natural killer cell** | **Natural killer T cell** | **Neutrophil** | **Plasmacytoid dendritic cell** | **Regulatory T cell** | **T follicular helper cell** | **Type 1 T helper cell** | **Type 17 T helper cell** | **Type 2 T helper cell** |
| --- | --- | --- | --- | --- | --- | --- | --- | --- | --- | --- | --- | --- | --- | --- | --- | --- | --- | --- | --- | --- | --- | --- | --- | --- | --- | --- | --- | --- |
| Activated B cell | 1 | 0.2 | 0.4 | 0.2 | -0.2 | -1 | 0.4 | 0.4 | -1 | -0.2 | -0.4 | 0.8 | 0.2 | -1 | 0.4 | 0.8 | 0.2 | 0 | -0.8 | -0.8 | -0.2 | 0.8 | -0.6 | 0.2 | -0.4 | 0.4 | 0.4 | -0.4 |
| Activated CD4 T cell | 0.2 | 1 | 0.8 | 1 | 0.6 | -0.2 | 0.8 | 0.8 | -0.2 | 0.6 | -0.8 | 0.4 | 1 | -0.2 | 0.8 | 0.4 | 1 | -0.4 | 0.4 | 0.4 | 0.6 | 0.4 | 0.2 | 1 | 0.8 | 0.8 | 0 | -0.8 |
| Activated CD8 T cell | 0.4 | 0.8 | 1 | 0.8 | 0 | -0.4 | 0.4 | 1 | -0.4 | 0 | -0.4 | 0.2 | 0.8 | -0.4 | 1 | 0.8 | 0.8 | -0.8 | 0.2 | 0.2 | 0 | 0.2 | 0.4 | 0.8 | 0.4 | 0.4 | 0.6 | -1 |
| Activated dendritic cell | 0.2 | 1 | 0.8 | 1 | 0.6 | -0.2 | 0.8 | 0.8 | -0.2 | 0.6 | -0.8 | 0.4 | 1 | -0.2 | 0.8 | 0.4 | 1 | -0.4 | 0.4 | 0.4 | 0.6 | 0.4 | 0.2 | 1 | 0.8 | 0.8 | 0 | -0.8 |
| CD56bright natural killer cell | -0.2 | 0.6 | 0 | 0.6 | 1 | 0.2 | 0.8 | 0 | 0.2 | 1 | -0.8 | 0.4 | 0.6 | 0.2 | 0 | -0.4 | 0.6 | 0.4 | 0.4 | 0.4 | 1 | 0.4 | -0.2 | 0.6 | 0.8 | 0.8 | -0.8 | 0 |
| CD56dim natural killer cell | -1 | -0.2 | -0.4 | -0.2 | 0.2 | 1 | -0.4 | -0.4 | 1 | 0.2 | 0.4 | -0.8 | -0.2 | 1 | -0.4 | -0.8 | -0.2 | 0 | 0.8 | 0.8 | 0.2 | -0.8 | 0.6 | -0.2 | 0.4 | -0.4 | -0.4 | 0.4 |
| Central memory CD4 T cell | 0.4 | 0.8 | 0.4 | 0.8 | 0.8 | -0.4 | 1 | 0.4 | -0.4 | 0.8 | -1 | 0.8 | 0.8 | -0.4 | 0.4 | 0.2 | 0.8 | 0.2 | 0 | 0 | 0.8 | 0.8 | -0.4 | 0.8 | 0.6 | 1 | -0.4 | -0.4 |
| Central memory CD8 T cell | 0.4 | 0.8 | 1 | 0.8 | 0 | -0.4 | 0.4 | 1 | -0.4 | 0 | -0.4 | 0.2 | 0.8 | -0.4 | 1 | 0.8 | 0.8 | -0.8 | 0.2 | 0.2 | 0 | 0.2 | 0.4 | 0.8 | 0.4 | 0.4 | 0.6 | -1 |
| Effector memeory CD4 T cell | -1 | -0.2 | -0.4 | -0.2 | 0.2 | 1 | -0.4 | -0.4 | 1 | 0.2 | 0.4 | -0.8 | -0.2 | 1 | -0.4 | -0.8 | -0.2 | 0 | 0.8 | 0.8 | 0.2 | -0.8 | 0.6 | -0.2 | 0.4 | -0.4 | -0.4 | 0.4 |
| Effector memeory CD8 T cell | -0.2 | 0.6 | 0 | 0.6 | 1 | 0.2 | 0.8 | 0 | 0.2 | 1 | -0.8 | 0.4 | 0.6 | 0.2 | 0 | -0.4 | 0.6 | 0.4 | 0.4 | 0.4 | 1 | 0.4 | -0.2 | 0.6 | 0.8 | 0.8 | -0.8 | 0 |
| Eosinophil | -0.4 | -0.8 | -0.4 | -0.8 | -0.8 | 0.4 | -1 | -0.4 | 0.4 | -0.8 | 1 | -0.8 | -0.8 | 0.4 | -0.4 | -0.2 | -0.8 | -0.2 | 0 | 0 | -0.8 | -0.8 | 0.4 | -0.8 | -0.6 | -1 | 0.4 | 0.4 |
| Gamma delta T cell | 0.8 | 0.4 | 0.2 | 0.4 | 0.4 | -0.8 | 0.8 | 0.2 | -0.8 | 0.4 | -0.8 | 1 | 0.4 | -0.8 | 0.2 | 0.4 | 0.4 | 0.4 | -0.6 | -0.6 | 0.4 | 1 | -0.8 | 0.4 | 0 | 0.8 | -0.2 | -0.2 |
| Immature B cell | 0.2 | 1 | 0.8 | 1 | 0.6 | -0.2 | 0.8 | 0.8 | -0.2 | 0.6 | -0.8 | 0.4 | 1 | -0.2 | 0.8 | 0.4 | 1 | -0.4 | 0.4 | 0.4 | 0.6 | 0.4 | 0.2 | 1 | 0.8 | 0.8 | 0 | -0.8 |
| Immature dendritic cell | -1 | -0.2 | -0.4 | -0.2 | 0.2 | 1 | -0.4 | -0.4 | 1 | 0.2 | 0.4 | -0.8 | -0.2 | 1 | -0.4 | -0.8 | -0.2 | 0 | 0.8 | 0.8 | 0.2 | -0.8 | 0.6 | -0.2 | 0.4 | -0.4 | -0.4 | 0.4 |
| Macrophage | 0.4 | 0.8 | 1 | 0.8 | 0 | -0.4 | 0.4 | 1 | -0.4 | 0 | -0.4 | 0.2 | 0.8 | -0.4 | 1 | 0.8 | 0.8 | -0.8 | 0.2 | 0.2 | 0 | 0.2 | 0.4 | 0.8 | 0.4 | 0.4 | 0.6 | -1 |
| Mast cell | 0.8 | 0.4 | 0.8 | 0.4 | -0.4 | -0.8 | 0.2 | 0.8 | -0.8 | -0.4 | -0.2 | 0.4 | 0.4 | -0.8 | 0.8 | 1 | 0.4 | -0.6 | -0.4 | -0.4 | -0.4 | 0.4 | 0 | 0.4 | -0.2 | 0.2 | 0.8 | -0.8 |
| MDSC | 0.2 | 1 | 0.8 | 1 | 0.6 | -0.2 | 0.8 | 0.8 | -0.2 | 0.6 | -0.8 | 0.4 | 1 | -0.2 | 0.8 | 0.4 | 1 | -0.4 | 0.4 | 0.4 | 0.6 | 0.4 | 0.2 | 1 | 0.8 | 0.8 | 0 | -0.8 |
| Memory B cell | 0 | -0.4 | -0.8 | -0.4 | 0.4 | 0 | 0.2 | -0.8 | 0 | 0.4 | -0.2 | 0.4 | -0.4 | 0 | -0.8 | -0.6 | -0.4 | 1 | -0.4 | -0.4 | 0.4 | 0.4 | -0.8 | -0.4 | -0.2 | 0.2 | -0.8 | 0.8 |
| Monocyte | -0.8 | 0.4 | 0.2 | 0.4 | 0.4 | 0.8 | 0 | 0.2 | 0.8 | 0.4 | 0 | -0.6 | 0.4 | 0.8 | 0.2 | -0.4 | 0.4 | -0.4 | 1 | 1 | 0.4 | -0.6 | 0.8 | 0.4 | 0.8 | 0 | -0.2 | -0.2 |
| Natural killer cell | -0.8 | 0.4 | 0.2 | 0.4 | 0.4 | 0.8 | 0 | 0.2 | 0.8 | 0.4 | 0 | -0.6 | 0.4 | 0.8 | 0.2 | -0.4 | 0.4 | -0.4 | 1 | 1 | 0.4 | -0.6 | 0.8 | 0.4 | 0.8 | 0 | -0.2 | -0.2 |
| Natural killer T cell | -0.2 | 0.6 | 0 | 0.6 | 1 | 0.2 | 0.8 | 0 | 0.2 | 1 | -0.8 | 0.4 | 0.6 | 0.2 | 0 | -0.4 | 0.6 | 0.4 | 0.4 | 0.4 | 1 | 0.4 | -0.2 | 0.6 | 0.8 | 0.8 | -0.8 | 0 |
| Neutrophil | 0.8 | 0.4 | 0.2 | 0.4 | 0.4 | -0.8 | 0.8 | 0.2 | -0.8 | 0.4 | -0.8 | 1 | 0.4 | -0.8 | 0.2 | 0.4 | 0.4 | 0.4 | -0.6 | -0.6 | 0.4 | 1 | -0.8 | 0.4 | 0 | 0.8 | -0.2 | -0.2 |
| Plasmacytoid dendritic cell | -0.6 | 0.2 | 0.4 | 0.2 | -0.2 | 0.6 | -0.4 | 0.4 | 0.6 | -0.2 | 0.4 | -0.8 | 0.2 | 0.6 | 0.4 | 0 | 0.2 | -0.8 | 0.8 | 0.8 | -0.2 | -0.8 | 1 | 0.2 | 0.4 | -0.4 | 0.4 | -0.4 |
| Regulatory T cell | 0.2 | 1 | 0.8 | 1 | 0.6 | -0.2 | 0.8 | 0.8 | -0.2 | 0.6 | -0.8 | 0.4 | 1 | -0.2 | 0.8 | 0.4 | 1 | -0.4 | 0.4 | 0.4 | 0.6 | 0.4 | 0.2 | 1 | 0.8 | 0.8 | 0 | -0.8 |
| T follicular helper cell | -0.4 | 0.8 | 0.4 | 0.8 | 0.8 | 0.4 | 0.6 | 0.4 | 0.4 | 0.8 | -0.6 | 0 | 0.8 | 0.4 | 0.4 | -0.2 | 0.8 | -0.2 | 0.8 | 0.8 | 0.8 | 0 | 0.4 | 0.8 | 1 | 0.6 | -0.4 | -0.4 |
| Type 1 T helper cell | 0.4 | 0.8 | 0.4 | 0.8 | 0.8 | -0.4 | 1 | 0.4 | -0.4 | 0.8 | -1 | 0.8 | 0.8 | -0.4 | 0.4 | 0.2 | 0.8 | 0.2 | 0 | 0 | 0.8 | 0.8 | -0.4 | 0.8 | 0.6 | 1 | -0.4 | -0.4 |
| Type 17 T helper cell | 0.4 | 0 | 0.6 | 0 | -0.8 | -0.4 | -0.4 | 0.6 | -0.4 | -0.8 | 0.4 | -0.2 | 0 | -0.4 | 0.6 | 0.8 | 0 | -0.8 | -0.2 | -0.2 | -0.8 | -0.2 | 0.4 | 0 | -0.4 | -0.4 | 1 | -0.6 |
| Type 2 T helper cell | -0.4 | -0.8 | -1 | -0.8 | 0 | 0.4 | -0.4 | -1 | 0.4 | 0 | 0.4 | -0.2 | -0.8 | 0.4 | -1 | -0.8 | -0.8 | 0.8 | -0.2 | -0.2 | 0 | -0.2 | -0.4 | -0.8 | -0.4 | -0.4 | -0.6 | 1 |

# Supplementary Table S16 (Sheet 7). P-value matrix (Sham)

|  | **Activated B cell** | **Activated CD4 T cell** | **Activated CD8 T cell** | **Activated dendritic cell** | **CD56bright natural killer cell** | **CD56dim natural killer cell** | **Central memory CD4 T cell** | **Central memory CD8 T cell** | **Effector memeory CD4 T cell** | **Effector memeory CD8 T cell** | **Eosinophil** | **Gamma delta T cell** | **Immature B cell** | **Immature dendritic cell** | **Macrophage** | **Mast cell** | **MDSC** | **Memory B cell** | **Monocyte** | **Natural killer cell** | **Natural killer T cell** | **Neutrophil** | **Plasmacytoid dendritic cell** | **Regulatory T cell** | **T follicular helper cell** | **Type 1 T helper cell** | **Type 17 T helper cell** | **Type 2 T helper cell** |
| --- | --- | --- | --- | --- | --- | --- | --- | --- | --- | --- | --- | --- | --- | --- | --- | --- | --- | --- | --- | --- | --- | --- | --- | --- | --- | --- | --- | --- |
| Activated B cell | 0 | 0.083333 | 0.333333 | 0.75 | 0.916667 | 0.75 | 0.75 | 0.75 | 0.333333 | 0.75 | 0.083333 | 0.333333 | 1 | 0.916667 | 0.75 | 0.916667 | 0.916667 | 0.083333 | 0.75 | 0.333333 | 0.083333 | 0.75 | 0.916667 | 0.75 | 0.333333 | 0.916667 | 0.333333 | 0.416667 |
| Activated CD4 T cell | 0.083333 | 0 | 0.333333 | 0.75 | 0.916667 | 0.75 | 0.75 | 0.75 | 0.333333 | 0.75 | 0.083333 | 0.333333 | 1 | 0.916667 | 0.75 | 0.916667 | 0.916667 | 0.083333 | 0.75 | 0.333333 | 0.083333 | 0.75 | 0.916667 | 0.75 | 0.333333 | 0.916667 | 0.333333 | 0.416667 |
| Activated CD8 T cell | 0.333333 | 0.333333 | 0 | 1 | 0.75 | 0.333333 | 0.333333 | 1 | 0.75 | 1 | 0.333333 | 0.083333 | 0.75 | 0.75 | 0.916667 | 0.75 | 0.75 | 0.333333 | 0.333333 | 0.083333 | 0.333333 | 0.916667 | 0.75 | 0.333333 | 0.083333 | 0.75 | 0.083333 | 0.333333 |
| Activated dendritic cell | 0.75 | 0.75 | 1 | 0 | 0.333333 | 0.416667 | 0.416667 | 0.083333 | 0.916667 | 0.083333 | 0.75 | 1 | 0.916667 | 0.333333 | 0.75 | 0.333333 | 0.333333 | 0.75 | 0.416667 | 1 | 0.75 | 0.75 | 0.333333 | 0.416667 | 1 | 0.333333 | 1 | 0.75 |
| CD56bright natural killer cell | 0.916667 | 0.916667 | 0.75 | 0.333333 | 0 | 0.333333 | 0.333333 | 0.333333 | 0.75 | 0.333333 | 0.916667 | 0.75 | 0.75 | 0.416667 | 0.333333 | 0.416667 | 0.083333 | 0.916667 | 0.333333 | 0.75 | 0.916667 | 1 | 0.416667 | 0.333333 | 0.75 | 0.416667 | 0.75 | 0.916667 |
| CD56dim natural killer cell | 0.75 | 0.75 | 0.333333 | 0.416667 | 0.333333 | 0 | 0.083333 | 0.416667 | 0.916667 | 0.416667 | 0.75 | 0.333333 | 0.916667 | 0.333333 | 0.75 | 0.333333 | 0.333333 | 0.75 | 0.083333 | 0.333333 | 0.75 | 0.75 | 0.333333 | 0.083333 | 0.333333 | 0.333333 | 0.333333 | 0.75 |
| Central memory CD4 T cell | 0.75 | 0.75 | 0.333333 | 0.416667 | 0.333333 | 0.083333 | 0 | 0.416667 | 0.916667 | 0.416667 | 0.75 | 0.333333 | 0.916667 | 0.333333 | 0.75 | 0.333333 | 0.333333 | 0.75 | 0.083333 | 0.333333 | 0.75 | 0.75 | 0.333333 | 0.083333 | 0.333333 | 0.333333 | 0.333333 | 0.75 |
| Central memory CD8 T cell | 0.75 | 0.75 | 1 | 0.083333 | 0.333333 | 0.416667 | 0.416667 | 0 | 0.916667 | 0.083333 | 0.75 | 1 | 0.916667 | 0.333333 | 0.75 | 0.333333 | 0.333333 | 0.75 | 0.416667 | 1 | 0.75 | 0.75 | 0.333333 | 0.416667 | 1 | 0.333333 | 1 | 0.75 |
| Effector memeory CD4 T cell | 0.333333 | 0.333333 | 0.75 | 0.916667 | 0.75 | 0.916667 | 0.916667 | 0.916667 | 0 | 0.916667 | 0.333333 | 0.75 | 0.416667 | 0.75 | 0.333333 | 0.75 | 0.75 | 0.333333 | 0.916667 | 0.75 | 0.333333 | 0.333333 | 0.75 | 0.916667 | 0.75 | 0.75 | 0.75 | 1 |
| Effector memeory CD8 T cell | 0.75 | 0.75 | 1 | 0.083333 | 0.333333 | 0.416667 | 0.416667 | 0.083333 | 0.916667 | 0 | 0.75 | 1 | 0.916667 | 0.333333 | 0.75 | 0.333333 | 0.333333 | 0.75 | 0.416667 | 1 | 0.75 | 0.75 | 0.333333 | 0.416667 | 1 | 0.333333 | 1 | 0.75 |
| Eosinophil | 0.083333 | 0.083333 | 0.333333 | 0.75 | 0.916667 | 0.75 | 0.75 | 0.75 | 0.333333 | 0.75 | 0 | 0.333333 | 1 | 0.916667 | 0.75 | 0.916667 | 0.916667 | 0.083333 | 0.75 | 0.333333 | 0.083333 | 0.75 | 0.916667 | 0.75 | 0.333333 | 0.916667 | 0.333333 | 0.416667 |
| Gamma delta T cell | 0.333333 | 0.333333 | 0.083333 | 1 | 0.75 | 0.333333 | 0.333333 | 1 | 0.75 | 1 | 0.333333 | 0 | 0.75 | 0.75 | 0.916667 | 0.75 | 0.75 | 0.333333 | 0.333333 | 0.083333 | 0.333333 | 0.916667 | 0.75 | 0.333333 | 0.083333 | 0.75 | 0.083333 | 0.333333 |
| Immature B cell | 1 | 1 | 0.75 | 0.916667 | 0.75 | 0.916667 | 0.916667 | 0.916667 | 0.416667 | 0.916667 | 1 | 0.75 | 0 | 0.75 | 0.333333 | 0.75 | 0.75 | 1 | 0.916667 | 0.75 | 1 | 0.333333 | 0.75 | 0.916667 | 0.75 | 0.75 | 0.75 | 0.333333 |
| Immature dendritic cell | 0.916667 | 0.916667 | 0.75 | 0.333333 | 0.416667 | 0.333333 | 0.333333 | 0.333333 | 0.75 | 0.333333 | 0.916667 | 0.75 | 0.75 | 0 | 1 | 0.083333 | 0.416667 | 0.916667 | 0.333333 | 0.75 | 0.916667 | 0.333333 | 0.083333 | 0.333333 | 0.75 | 0.083333 | 0.75 | 0.916667 |
| Macrophage | 0.75 | 0.75 | 0.916667 | 0.75 | 0.333333 | 0.75 | 0.75 | 0.75 | 0.333333 | 0.75 | 0.75 | 0.916667 | 0.333333 | 1 | 0 | 1 | 0.333333 | 0.75 | 0.75 | 0.916667 | 0.75 | 0.416667 | 1 | 0.75 | 0.916667 | 1 | 0.916667 | 0.75 |
| Mast cell | 0.916667 | 0.916667 | 0.75 | 0.333333 | 0.416667 | 0.333333 | 0.333333 | 0.333333 | 0.75 | 0.333333 | 0.916667 | 0.75 | 0.75 | 0.083333 | 1 | 0 | 0.416667 | 0.916667 | 0.333333 | 0.75 | 0.916667 | 0.333333 | 0.083333 | 0.333333 | 0.75 | 0.083333 | 0.75 | 0.916667 |
| MDSC | 0.916667 | 0.916667 | 0.75 | 0.333333 | 0.083333 | 0.333333 | 0.333333 | 0.333333 | 0.75 | 0.333333 | 0.916667 | 0.75 | 0.75 | 0.416667 | 0.333333 | 0.416667 | 0 | 0.916667 | 0.333333 | 0.75 | 0.916667 | 1 | 0.416667 | 0.333333 | 0.75 | 0.416667 | 0.75 | 0.916667 |
| Memory B cell | 0.083333 | 0.083333 | 0.333333 | 0.75 | 0.916667 | 0.75 | 0.75 | 0.75 | 0.333333 | 0.75 | 0.083333 | 0.333333 | 1 | 0.916667 | 0.75 | 0.916667 | 0.916667 | 0 | 0.75 | 0.333333 | 0.083333 | 0.75 | 0.916667 | 0.75 | 0.333333 | 0.916667 | 0.333333 | 0.416667 |
| Monocyte | 0.75 | 0.75 | 0.333333 | 0.416667 | 0.333333 | 0.083333 | 0.083333 | 0.416667 | 0.916667 | 0.416667 | 0.75 | 0.333333 | 0.916667 | 0.333333 | 0.75 | 0.333333 | 0.333333 | 0.75 | 0 | 0.333333 | 0.75 | 0.75 | 0.333333 | 0.083333 | 0.333333 | 0.333333 | 0.333333 | 0.75 |
| Natural killer cell | 0.333333 | 0.333333 | 0.083333 | 1 | 0.75 | 0.333333 | 0.333333 | 1 | 0.75 | 1 | 0.333333 | 0.083333 | 0.75 | 0.75 | 0.916667 | 0.75 | 0.75 | 0.333333 | 0.333333 | 0 | 0.333333 | 0.916667 | 0.75 | 0.333333 | 0.083333 | 0.75 | 0.083333 | 0.333333 |
| Natural killer T cell | 0.083333 | 0.083333 | 0.333333 | 0.75 | 0.916667 | 0.75 | 0.75 | 0.75 | 0.333333 | 0.75 | 0.083333 | 0.333333 | 1 | 0.916667 | 0.75 | 0.916667 | 0.916667 | 0.083333 | 0.75 | 0.333333 | 0 | 0.75 | 0.916667 | 0.75 | 0.333333 | 0.916667 | 0.333333 | 0.416667 |
| Neutrophil | 0.75 | 0.75 | 0.916667 | 0.75 | 1 | 0.75 | 0.75 | 0.75 | 0.333333 | 0.75 | 0.75 | 0.916667 | 0.333333 | 0.333333 | 0.416667 | 0.333333 | 1 | 0.75 | 0.75 | 0.916667 | 0.75 | 0 | 0.333333 | 0.75 | 0.916667 | 0.333333 | 0.916667 | 0.75 |
| Plasmacytoid dendritic cell | 0.916667 | 0.916667 | 0.75 | 0.333333 | 0.416667 | 0.333333 | 0.333333 | 0.333333 | 0.75 | 0.333333 | 0.916667 | 0.75 | 0.75 | 0.083333 | 1 | 0.083333 | 0.416667 | 0.916667 | 0.333333 | 0.75 | 0.916667 | 0.333333 | 0 | 0.333333 | 0.75 | 0.083333 | 0.75 | 0.916667 |
| Regulatory T cell | 0.75 | 0.75 | 0.333333 | 0.416667 | 0.333333 | 0.083333 | 0.083333 | 0.416667 | 0.916667 | 0.416667 | 0.75 | 0.333333 | 0.916667 | 0.333333 | 0.75 | 0.333333 | 0.333333 | 0.75 | 0.083333 | 0.333333 | 0.75 | 0.75 | 0.333333 | 0 | 0.333333 | 0.333333 | 0.333333 | 0.75 |
| T follicular helper cell | 0.333333 | 0.333333 | 0.083333 | 1 | 0.75 | 0.333333 | 0.333333 | 1 | 0.75 | 1 | 0.333333 | 0.083333 | 0.75 | 0.75 | 0.916667 | 0.75 | 0.75 | 0.333333 | 0.333333 | 0.083333 | 0.333333 | 0.916667 | 0.75 | 0.333333 | 0 | 0.75 | 0.083333 | 0.333333 |
| Type 1 T helper cell | 0.916667 | 0.916667 | 0.75 | 0.333333 | 0.416667 | 0.333333 | 0.333333 | 0.333333 | 0.75 | 0.333333 | 0.916667 | 0.75 | 0.75 | 0.083333 | 1 | 0.083333 | 0.416667 | 0.916667 | 0.333333 | 0.75 | 0.916667 | 0.333333 | 0.083333 | 0.333333 | 0.75 | 0 | 0.75 | 0.916667 |
| Type 17 T helper cell | 0.333333 | 0.333333 | 0.083333 | 1 | 0.75 | 0.333333 | 0.333333 | 1 | 0.75 | 1 | 0.333333 | 0.083333 | 0.75 | 0.75 | 0.916667 | 0.75 | 0.75 | 0.333333 | 0.333333 | 0.083333 | 0.333333 | 0.916667 | 0.75 | 0.333333 | 0.083333 | 0.75 | 0 | 0.333333 |
| Type 2 T helper cell | 0.416667 | 0.416667 | 0.333333 | 0.75 | 0.916667 | 0.75 | 0.75 | 0.75 | 1 | 0.75 | 0.416667 | 0.333333 | 0.333333 | 0.916667 | 0.75 | 0.916667 | 0.916667 | 0.416667 | 0.75 | 0.333333 | 0.416667 | 0.75 | 0.916667 | 0.75 | 0.333333 | 0.916667 | 0.333333 | 0 |

# Supplementary Table S16 (Sheet 8). P-value matrix (SCI_1d)

|  | **Activated B cell** | **Activated CD4 T cell** | **Activated CD8 T cell** | **Activated dendritic cell** | **CD56bright natural killer cell** | **CD56dim natural killer cell** | **Central memory CD4 T cell** | **Central memory CD8 T cell** | **Effector memeory CD4 T cell** | **Effector memeory CD8 T cell** | **Eosinophil** | **Gamma delta T cell** | **Immature B cell** | **Immature dendritic cell** | **Macrophage** | **Mast cell** | **MDSC** | **Memory B cell** | **Monocyte** | **Natural killer cell** | **Natural killer T cell** | **Neutrophil** | **Plasmacytoid dendritic cell** | **Regulatory T cell** | **T follicular helper cell** | **Type 1 T helper cell** | **Type 17 T helper cell** | **Type 2 T helper cell** |
| --- | --- | --- | --- | --- | --- | --- | --- | --- | --- | --- | --- | --- | --- | --- | --- | --- | --- | --- | --- | --- | --- | --- | --- | --- | --- | --- | --- | --- |
| Activated B cell | 0 | 0.333333 | 0.75 | 0.083333 | 0.75 | 0.333333 | 0.75 | 0.75 | 0.916667 | 0.333333 | 0.333333 | 0.916667 | 0.416667 | 0.75 | 0.333333 | 0.333333 | 0.916667 | 0.333333 | 0.916667 | 0.75 | 0.333333 | 0.75 | 0.916667 | 0.916667 | 0.75 | 0.75 | 0.75 | 0.75 |
| Activated CD4 T cell | 0.333333 | 0 | 1 | 0.333333 | 0.916667 | 0.75 | 1 | 0.333333 | 0.75 | 0.75 | 0.416667 | 0.75 | 0.333333 | 0.333333 | 0.416667 | 0.416667 | 0.75 | 0.416667 | 0.75 | 0.916667 | 0.416667 | 1 | 0.75 | 0.75 | 0.333333 | 0.333333 | 1 | 1 |
| Activated CD8 T cell | 0.75 | 1 | 0 | 0.75 | 0.75 | 0.916667 | 0.083333 | 0.416667 | 0.333333 | 0.916667 | 0.333333 | 0.333333 | 0.75 | 0.416667 | 0.333333 | 0.333333 | 0.333333 | 0.333333 | 0.333333 | 0.75 | 0.333333 | 0.083333 | 0.333333 | 0.333333 | 0.416667 | 0.416667 | 0.083333 | 0.083333 |
| Activated dendritic cell | 0.083333 | 0.333333 | 0.75 | 0 | 0.75 | 0.333333 | 0.75 | 0.75 | 0.916667 | 0.333333 | 0.333333 | 0.916667 | 0.416667 | 0.75 | 0.333333 | 0.333333 | 0.916667 | 0.333333 | 0.916667 | 0.75 | 0.333333 | 0.75 | 0.916667 | 0.916667 | 0.75 | 0.75 | 0.75 | 0.75 |
| CD56bright natural killer cell | 0.75 | 0.916667 | 0.75 | 0.75 | 0 | 0.333333 | 0.75 | 0.75 | 1 | 0.333333 | 0.916667 | 0.333333 | 0.75 | 0.75 | 0.916667 | 0.916667 | 0.333333 | 0.916667 | 1 | 0.083333 | 0.916667 | 0.75 | 0.333333 | 0.333333 | 0.75 | 0.75 | 0.75 | 0.75 |
| CD56dim natural killer cell | 0.333333 | 0.75 | 0.916667 | 0.333333 | 0.333333 | 0 | 0.916667 | 0.916667 | 0.75 | 0.083333 | 0.75 | 0.75 | 1 | 0.916667 | 0.75 | 0.75 | 0.75 | 0.75 | 0.75 | 0.333333 | 0.75 | 0.916667 | 0.75 | 0.75 | 0.916667 | 0.916667 | 0.916667 | 0.916667 |
| Central memory CD4 T cell | 0.75 | 1 | 0.083333 | 0.75 | 0.75 | 0.916667 | 0 | 0.416667 | 0.333333 | 0.916667 | 0.333333 | 0.333333 | 0.75 | 0.416667 | 0.333333 | 0.333333 | 0.333333 | 0.333333 | 0.333333 | 0.75 | 0.333333 | 0.083333 | 0.333333 | 0.333333 | 0.416667 | 0.416667 | 0.083333 | 0.083333 |
| Central memory CD8 T cell | 0.75 | 0.333333 | 0.416667 | 0.75 | 0.75 | 0.916667 | 0.416667 | 0 | 0.333333 | 0.916667 | 1 | 0.333333 | 0.75 | 0.083333 | 1 | 1 | 0.333333 | 1 | 0.333333 | 0.75 | 1 | 0.416667 | 0.333333 | 0.333333 | 0.083333 | 0.083333 | 0.416667 | 0.416667 |
| Effector memeory CD4 T cell | 0.916667 | 0.75 | 0.333333 | 0.916667 | 1 | 0.75 | 0.333333 | 0.333333 | 0 | 0.75 | 0.75 | 0.416667 | 0.916667 | 0.333333 | 0.75 | 0.75 | 0.416667 | 0.75 | 0.083333 | 1 | 0.75 | 0.333333 | 0.416667 | 0.416667 | 0.333333 | 0.333333 | 0.333333 | 0.333333 |
| Effector memeory CD8 T cell | 0.333333 | 0.75 | 0.916667 | 0.333333 | 0.333333 | 0.083333 | 0.916667 | 0.916667 | 0.75 | 0 | 0.75 | 0.75 | 1 | 0.916667 | 0.75 | 0.75 | 0.75 | 0.75 | 0.75 | 0.333333 | 0.75 | 0.916667 | 0.75 | 0.75 | 0.916667 | 0.916667 | 0.916667 | 0.916667 |
| Eosinophil | 0.333333 | 0.416667 | 0.333333 | 0.333333 | 0.916667 | 0.75 | 0.333333 | 1 | 0.75 | 0.75 | 0 | 0.75 | 0.333333 | 1 | 0.083333 | 0.083333 | 0.75 | 0.083333 | 0.75 | 0.916667 | 0.083333 | 0.333333 | 0.75 | 0.75 | 1 | 1 | 0.333333 | 0.333333 |
| Gamma delta T cell | 0.916667 | 0.75 | 0.333333 | 0.916667 | 0.333333 | 0.75 | 0.333333 | 0.333333 | 0.416667 | 0.75 | 0.75 | 0 | 0.916667 | 0.333333 | 0.75 | 0.75 | 0.083333 | 0.75 | 0.416667 | 0.333333 | 0.75 | 0.333333 | 0.083333 | 0.083333 | 0.333333 | 0.333333 | 0.333333 | 0.333333 |
| Immature B cell | 0.416667 | 0.333333 | 0.75 | 0.416667 | 0.75 | 1 | 0.75 | 0.75 | 0.916667 | 1 | 0.333333 | 0.916667 | 0 | 0.75 | 0.333333 | 0.333333 | 0.916667 | 0.333333 | 0.916667 | 0.75 | 0.333333 | 0.75 | 0.916667 | 0.916667 | 0.75 | 0.75 | 0.75 | 0.75 |
| Immature dendritic cell | 0.75 | 0.333333 | 0.416667 | 0.75 | 0.75 | 0.916667 | 0.416667 | 0.083333 | 0.333333 | 0.916667 | 1 | 0.333333 | 0.75 | 0 | 1 | 1 | 0.333333 | 1 | 0.333333 | 0.75 | 1 | 0.416667 | 0.333333 | 0.333333 | 0.083333 | 0.083333 | 0.416667 | 0.416667 |
| Macrophage | 0.333333 | 0.416667 | 0.333333 | 0.333333 | 0.916667 | 0.75 | 0.333333 | 1 | 0.75 | 0.75 | 0.083333 | 0.75 | 0.333333 | 1 | 0 | 0.083333 | 0.75 | 0.083333 | 0.75 | 0.916667 | 0.083333 | 0.333333 | 0.75 | 0.75 | 1 | 1 | 0.333333 | 0.333333 |
| Mast cell | 0.333333 | 0.416667 | 0.333333 | 0.333333 | 0.916667 | 0.75 | 0.333333 | 1 | 0.75 | 0.75 | 0.083333 | 0.75 | 0.333333 | 1 | 0.083333 | 0 | 0.75 | 0.083333 | 0.75 | 0.916667 | 0.083333 | 0.333333 | 0.75 | 0.75 | 1 | 1 | 0.333333 | 0.333333 |
| MDSC | 0.916667 | 0.75 | 0.333333 | 0.916667 | 0.333333 | 0.75 | 0.333333 | 0.333333 | 0.416667 | 0.75 | 0.75 | 0.083333 | 0.916667 | 0.333333 | 0.75 | 0.75 | 0 | 0.75 | 0.416667 | 0.333333 | 0.75 | 0.333333 | 0.083333 | 0.083333 | 0.333333 | 0.333333 | 0.333333 | 0.333333 |
| Memory B cell | 0.333333 | 0.416667 | 0.333333 | 0.333333 | 0.916667 | 0.75 | 0.333333 | 1 | 0.75 | 0.75 | 0.083333 | 0.75 | 0.333333 | 1 | 0.083333 | 0.083333 | 0.75 | 0 | 0.75 | 0.916667 | 0.083333 | 0.333333 | 0.75 | 0.75 | 1 | 1 | 0.333333 | 0.333333 |
| Monocyte | 0.916667 | 0.75 | 0.333333 | 0.916667 | 1 | 0.75 | 0.333333 | 0.333333 | 0.083333 | 0.75 | 0.75 | 0.416667 | 0.916667 | 0.333333 | 0.75 | 0.75 | 0.416667 | 0.75 | 0 | 1 | 0.75 | 0.333333 | 0.416667 | 0.416667 | 0.333333 | 0.333333 | 0.333333 | 0.333333 |
| Natural killer cell | 0.75 | 0.916667 | 0.75 | 0.75 | 0.083333 | 0.333333 | 0.75 | 0.75 | 1 | 0.333333 | 0.916667 | 0.333333 | 0.75 | 0.75 | 0.916667 | 0.916667 | 0.333333 | 0.916667 | 1 | 0 | 0.916667 | 0.75 | 0.333333 | 0.333333 | 0.75 | 0.75 | 0.75 | 0.75 |
| Natural killer T cell | 0.333333 | 0.416667 | 0.333333 | 0.333333 | 0.916667 | 0.75 | 0.333333 | 1 | 0.75 | 0.75 | 0.083333 | 0.75 | 0.333333 | 1 | 0.083333 | 0.083333 | 0.75 | 0.083333 | 0.75 | 0.916667 | 0 | 0.333333 | 0.75 | 0.75 | 1 | 1 | 0.333333 | 0.333333 |
| Neutrophil | 0.75 | 1 | 0.083333 | 0.75 | 0.75 | 0.916667 | 0.083333 | 0.416667 | 0.333333 | 0.916667 | 0.333333 | 0.333333 | 0.75 | 0.416667 | 0.333333 | 0.333333 | 0.333333 | 0.333333 | 0.333333 | 0.75 | 0.333333 | 0 | 0.333333 | 0.333333 | 0.416667 | 0.416667 | 0.083333 | 0.083333 |
| Plasmacytoid dendritic cell | 0.916667 | 0.75 | 0.333333 | 0.916667 | 0.333333 | 0.75 | 0.333333 | 0.333333 | 0.416667 | 0.75 | 0.75 | 0.083333 | 0.916667 | 0.333333 | 0.75 | 0.75 | 0.083333 | 0.75 | 0.416667 | 0.333333 | 0.75 | 0.333333 | 0 | 0.083333 | 0.333333 | 0.333333 | 0.333333 | 0.333333 |
| Regulatory T cell | 0.916667 | 0.75 | 0.333333 | 0.916667 | 0.333333 | 0.75 | 0.333333 | 0.333333 | 0.416667 | 0.75 | 0.75 | 0.083333 | 0.916667 | 0.333333 | 0.75 | 0.75 | 0.083333 | 0.75 | 0.416667 | 0.333333 | 0.75 | 0.333333 | 0.083333 | 0 | 0.333333 | 0.333333 | 0.333333 | 0.333333 |
| T follicular helper cell | 0.75 | 0.333333 | 0.416667 | 0.75 | 0.75 | 0.916667 | 0.416667 | 0.083333 | 0.333333 | 0.916667 | 1 | 0.333333 | 0.75 | 0.083333 | 1 | 1 | 0.333333 | 1 | 0.333333 | 0.75 | 1 | 0.416667 | 0.333333 | 0.333333 | 0 | 0.083333 | 0.416667 | 0.416667 |
| Type 1 T helper cell | 0.75 | 0.333333 | 0.416667 | 0.75 | 0.75 | 0.916667 | 0.416667 | 0.083333 | 0.333333 | 0.916667 | 1 | 0.333333 | 0.75 | 0.083333 | 1 | 1 | 0.333333 | 1 | 0.333333 | 0.75 | 1 | 0.416667 | 0.333333 | 0.333333 | 0.083333 | 0 | 0.416667 | 0.416667 |
| Type 17 T helper cell | 0.75 | 1 | 0.083333 | 0.75 | 0.75 | 0.916667 | 0.083333 | 0.416667 | 0.333333 | 0.916667 | 0.333333 | 0.333333 | 0.75 | 0.416667 | 0.333333 | 0.333333 | 0.333333 | 0.333333 | 0.333333 | 0.75 | 0.333333 | 0.083333 | 0.333333 | 0.333333 | 0.416667 | 0.416667 | 0 | 0.083333 |
| Type 2 T helper cell | 0.75 | 1 | 0.083333 | 0.75 | 0.75 | 0.916667 | 0.083333 | 0.416667 | 0.333333 | 0.916667 | 0.333333 | 0.333333 | 0.75 | 0.416667 | 0.333333 | 0.333333 | 0.333333 | 0.333333 | 0.333333 | 0.75 | 0.333333 | 0.083333 | 0.333333 | 0.333333 | 0.416667 | 0.416667 | 0.083333 | 0 |

# Supplementary Table S16 (Sheet 9). P-value matrix (SCI_3d)

|  | **Activated B cell** | **Activated CD4 T cell** | **Activated CD8 T cell** | **Activated dendritic cell** | **CD56bright natural killer cell** | **CD56dim natural killer cell** | **Central memory CD4 T cell** | **Central memory CD8 T cell** | **Effector memeory CD4 T cell** | **Effector memeory CD8 T cell** | **Eosinophil** | **Gamma delta T cell** | **Immature B cell** | **Immature dendritic cell** | **Macrophage** | **Mast cell** | **MDSC** | **Memory B cell** | **Monocyte** | **Natural killer cell** | **Natural killer T cell** | **Neutrophil** | **Plasmacytoid dendritic cell** | **Regulatory T cell** | **T follicular helper cell** | **Type 1 T helper cell** | **Type 17 T helper cell** | **Type 2 T helper cell** |
| --- | --- | --- | --- | --- | --- | --- | --- | --- | --- | --- | --- | --- | --- | --- | --- | --- | --- | --- | --- | --- | --- | --- | --- | --- | --- | --- | --- | --- |
| Activated B cell | 0 | 0.333333 | 0.916667 | 0.416667 | 0.333333 | 0.416667 | 0.333333 | 0.416667 | 0.083333 | 0.333333 | 0.75 | 0.416667 | 0.333333 | 0.333333 | 0.75 | 0.333333 | 0.333333 | 0.333333 | 0.416667 | 0.333333 | 0.916667 | 0.75 | 0.083333 | 0.333333 | 1 | 0.75 | 0.416667 | 0.916667 |
| Activated CD4 T cell | 0.333333 | 0 | 0.75 | 0.333333 | 0.083333 | 0.333333 | 0.416667 | 0.333333 | 0.333333 | 0.083333 | 0.333333 | 0.333333 | 0.083333 | 0.416667 | 0.916667 | 0.416667 | 0.083333 | 0.75 | 0.333333 | 0.416667 | 0.75 | 0.916667 | 0.333333 | 0.083333 | 0.75 | 0.916667 | 0.333333 | 0.75 |
| Activated CD8 T cell | 0.916667 | 0.75 | 0 | 0.916667 | 0.75 | 0.916667 | 0.75 | 0.916667 | 0.916667 | 0.75 | 0.333333 | 0.916667 | 0.75 | 0.75 | 1 | 0.75 | 0.75 | 0.75 | 0.916667 | 0.75 | 0.083333 | 1 | 0.916667 | 0.75 | 0.75 | 0.333333 | 0.916667 | 0.083333 |
| Activated dendritic cell | 0.416667 | 0.333333 | 0.916667 | 0 | 0.333333 | 0.083333 | 0.333333 | 0.083333 | 0.416667 | 0.333333 | 0.75 | 0.083333 | 0.333333 | 0.333333 | 0.75 | 0.333333 | 0.333333 | 1 | 0.083333 | 0.333333 | 0.916667 | 0.75 | 0.416667 | 0.333333 | 0.333333 | 0.75 | 0.083333 | 0.916667 |
| CD56bright natural killer cell | 0.333333 | 0.083333 | 0.75 | 0.333333 | 0 | 0.333333 | 0.416667 | 0.333333 | 0.333333 | 0.083333 | 0.333333 | 0.333333 | 0.083333 | 0.416667 | 0.916667 | 0.416667 | 0.083333 | 0.75 | 0.333333 | 0.416667 | 0.75 | 0.916667 | 0.333333 | 0.083333 | 0.75 | 0.916667 | 0.333333 | 0.75 |
| CD56dim natural killer cell | 0.416667 | 0.333333 | 0.916667 | 0.083333 | 0.333333 | 0 | 0.333333 | 0.083333 | 0.416667 | 0.333333 | 0.75 | 0.083333 | 0.333333 | 0.333333 | 0.75 | 0.333333 | 0.333333 | 1 | 0.083333 | 0.333333 | 0.916667 | 0.75 | 0.416667 | 0.333333 | 0.333333 | 0.75 | 0.083333 | 0.916667 |
| Central memory CD4 T cell | 0.333333 | 0.416667 | 0.75 | 0.333333 | 0.416667 | 0.333333 | 0 | 0.333333 | 0.333333 | 0.416667 | 1 | 0.333333 | 0.416667 | 0.083333 | 0.916667 | 0.083333 | 0.416667 | 0.75 | 0.333333 | 0.083333 | 0.75 | 0.916667 | 0.333333 | 0.416667 | 0.75 | 0.916667 | 0.333333 | 0.75 |
| Central memory CD8 T cell | 0.416667 | 0.333333 | 0.916667 | 0.083333 | 0.333333 | 0.083333 | 0.333333 | 0 | 0.416667 | 0.333333 | 0.75 | 0.083333 | 0.333333 | 0.333333 | 0.75 | 0.333333 | 0.333333 | 1 | 0.083333 | 0.333333 | 0.916667 | 0.75 | 0.416667 | 0.333333 | 0.333333 | 0.75 | 0.083333 | 0.916667 |
| Effector memeory CD4 T cell | 0.083333 | 0.333333 | 0.916667 | 0.416667 | 0.333333 | 0.416667 | 0.333333 | 0.416667 | 0 | 0.333333 | 0.75 | 0.416667 | 0.333333 | 0.333333 | 0.75 | 0.333333 | 0.333333 | 0.333333 | 0.416667 | 0.333333 | 0.916667 | 0.75 | 0.083333 | 0.333333 | 1 | 0.75 | 0.416667 | 0.916667 |
| Effector memeory CD8 T cell | 0.333333 | 0.083333 | 0.75 | 0.333333 | 0.083333 | 0.333333 | 0.416667 | 0.333333 | 0.333333 | 0 | 0.333333 | 0.333333 | 0.083333 | 0.416667 | 0.916667 | 0.416667 | 0.083333 | 0.75 | 0.333333 | 0.416667 | 0.75 | 0.916667 | 0.333333 | 0.083333 | 0.75 | 0.916667 | 0.333333 | 0.75 |
| Eosinophil | 0.75 | 0.333333 | 0.333333 | 0.75 | 0.333333 | 0.75 | 1 | 0.75 | 0.75 | 0.333333 | 0 | 0.75 | 0.333333 | 1 | 0.75 | 1 | 0.333333 | 0.916667 | 0.75 | 1 | 0.333333 | 0.75 | 0.75 | 0.333333 | 0.916667 | 0.75 | 0.75 | 0.333333 |
| Gamma delta T cell | 0.416667 | 0.333333 | 0.916667 | 0.083333 | 0.333333 | 0.083333 | 0.333333 | 0.083333 | 0.416667 | 0.333333 | 0.75 | 0 | 0.333333 | 0.333333 | 0.75 | 0.333333 | 0.333333 | 1 | 0.083333 | 0.333333 | 0.916667 | 0.75 | 0.416667 | 0.333333 | 0.333333 | 0.75 | 0.083333 | 0.916667 |
| Immature B cell | 0.333333 | 0.083333 | 0.75 | 0.333333 | 0.083333 | 0.333333 | 0.416667 | 0.333333 | 0.333333 | 0.083333 | 0.333333 | 0.333333 | 0 | 0.416667 | 0.916667 | 0.416667 | 0.083333 | 0.75 | 0.333333 | 0.416667 | 0.75 | 0.916667 | 0.333333 | 0.083333 | 0.75 | 0.916667 | 0.333333 | 0.75 |
| Immature dendritic cell | 0.333333 | 0.416667 | 0.75 | 0.333333 | 0.416667 | 0.333333 | 0.083333 | 0.333333 | 0.333333 | 0.416667 | 1 | 0.333333 | 0.416667 | 0 | 0.916667 | 0.083333 | 0.416667 | 0.75 | 0.333333 | 0.083333 | 0.75 | 0.916667 | 0.333333 | 0.416667 | 0.75 | 0.916667 | 0.333333 | 0.75 |
| Macrophage | 0.75 | 0.916667 | 1 | 0.75 | 0.916667 | 0.75 | 0.916667 | 0.75 | 0.75 | 0.916667 | 0.75 | 0.75 | 0.916667 | 0.916667 | 0 | 0.916667 | 0.916667 | 0.333333 | 0.75 | 0.916667 | 1 | 0.083333 | 0.75 | 0.916667 | 0.333333 | 0.416667 | 0.75 | 1 |
| Mast cell | 0.333333 | 0.416667 | 0.75 | 0.333333 | 0.416667 | 0.333333 | 0.083333 | 0.333333 | 0.333333 | 0.416667 | 1 | 0.333333 | 0.416667 | 0.083333 | 0.916667 | 0 | 0.416667 | 0.75 | 0.333333 | 0.083333 | 0.75 | 0.916667 | 0.333333 | 0.416667 | 0.75 | 0.916667 | 0.333333 | 0.75 |
| MDSC | 0.333333 | 0.083333 | 0.75 | 0.333333 | 0.083333 | 0.333333 | 0.416667 | 0.333333 | 0.333333 | 0.083333 | 0.333333 | 0.333333 | 0.083333 | 0.416667 | 0.916667 | 0.416667 | 0 | 0.75 | 0.333333 | 0.416667 | 0.75 | 0.916667 | 0.333333 | 0.083333 | 0.75 | 0.916667 | 0.333333 | 0.75 |
| Memory B cell | 0.333333 | 0.75 | 0.75 | 1 | 0.75 | 1 | 0.75 | 1 | 0.333333 | 0.75 | 0.916667 | 1 | 0.75 | 0.75 | 0.333333 | 0.75 | 0.75 | 0 | 1 | 0.75 | 0.75 | 0.333333 | 0.333333 | 0.75 | 0.416667 | 0.333333 | 1 | 0.75 |
| Monocyte | 0.416667 | 0.333333 | 0.916667 | 0.083333 | 0.333333 | 0.083333 | 0.333333 | 0.083333 | 0.416667 | 0.333333 | 0.75 | 0.083333 | 0.333333 | 0.333333 | 0.75 | 0.333333 | 0.333333 | 1 | 0 | 0.333333 | 0.916667 | 0.75 | 0.416667 | 0.333333 | 0.333333 | 0.75 | 0.083333 | 0.916667 |
| Natural killer cell | 0.333333 | 0.416667 | 0.75 | 0.333333 | 0.416667 | 0.333333 | 0.083333 | 0.333333 | 0.333333 | 0.416667 | 1 | 0.333333 | 0.416667 | 0.083333 | 0.916667 | 0.083333 | 0.416667 | 0.75 | 0.333333 | 0 | 0.75 | 0.916667 | 0.333333 | 0.416667 | 0.75 | 0.916667 | 0.333333 | 0.75 |
| Natural killer T cell | 0.916667 | 0.75 | 0.083333 | 0.916667 | 0.75 | 0.916667 | 0.75 | 0.916667 | 0.916667 | 0.75 | 0.333333 | 0.916667 | 0.75 | 0.75 | 1 | 0.75 | 0.75 | 0.75 | 0.916667 | 0.75 | 0 | 1 | 0.916667 | 0.75 | 0.75 | 0.333333 | 0.916667 | 0.083333 |
| Neutrophil | 0.75 | 0.916667 | 1 | 0.75 | 0.916667 | 0.75 | 0.916667 | 0.75 | 0.75 | 0.916667 | 0.75 | 0.75 | 0.916667 | 0.916667 | 0.083333 | 0.916667 | 0.916667 | 0.333333 | 0.75 | 0.916667 | 1 | 0 | 0.75 | 0.916667 | 0.333333 | 0.416667 | 0.75 | 1 |
| Plasmacytoid dendritic cell | 0.083333 | 0.333333 | 0.916667 | 0.416667 | 0.333333 | 0.416667 | 0.333333 | 0.416667 | 0.083333 | 0.333333 | 0.75 | 0.416667 | 0.333333 | 0.333333 | 0.75 | 0.333333 | 0.333333 | 0.333333 | 0.416667 | 0.333333 | 0.916667 | 0.75 | 0 | 0.333333 | 1 | 0.75 | 0.416667 | 0.916667 |
| Regulatory T cell | 0.333333 | 0.083333 | 0.75 | 0.333333 | 0.083333 | 0.333333 | 0.416667 | 0.333333 | 0.333333 | 0.083333 | 0.333333 | 0.333333 | 0.083333 | 0.416667 | 0.916667 | 0.416667 | 0.083333 | 0.75 | 0.333333 | 0.416667 | 0.75 | 0.916667 | 0.333333 | 0 | 0.75 | 0.916667 | 0.333333 | 0.75 |
| T follicular helper cell | 1 | 0.75 | 0.75 | 0.333333 | 0.75 | 0.333333 | 0.75 | 0.333333 | 1 | 0.75 | 0.916667 | 0.333333 | 0.75 | 0.75 | 0.333333 | 0.75 | 0.75 | 0.416667 | 0.333333 | 0.75 | 0.75 | 0.333333 | 1 | 0.75 | 0 | 0.333333 | 0.333333 | 0.75 |
| Type 1 T helper cell | 0.75 | 0.916667 | 0.333333 | 0.75 | 0.916667 | 0.75 | 0.916667 | 0.75 | 0.75 | 0.916667 | 0.75 | 0.75 | 0.916667 | 0.916667 | 0.416667 | 0.916667 | 0.916667 | 0.333333 | 0.75 | 0.916667 | 0.333333 | 0.416667 | 0.75 | 0.916667 | 0.333333 | 0 | 0.75 | 0.333333 |
| Type 17 T helper cell | 0.416667 | 0.333333 | 0.916667 | 0.083333 | 0.333333 | 0.083333 | 0.333333 | 0.083333 | 0.416667 | 0.333333 | 0.75 | 0.083333 | 0.333333 | 0.333333 | 0.75 | 0.333333 | 0.333333 | 1 | 0.083333 | 0.333333 | 0.916667 | 0.75 | 0.416667 | 0.333333 | 0.333333 | 0.75 | 0 | 0.916667 |
| Type 2 T helper cell | 0.916667 | 0.75 | 0.083333 | 0.916667 | 0.75 | 0.916667 | 0.75 | 0.916667 | 0.916667 | 0.75 | 0.333333 | 0.916667 | 0.75 | 0.75 | 1 | 0.75 | 0.75 | 0.75 | 0.916667 | 0.75 | 0.083333 | 1 | 0.916667 | 0.75 | 0.75 | 0.333333 | 0.916667 | 0 |

# Supplementary Table S16 (Sheet 10). P-value matrix (SCI_7d)

|  | **Activated B cell** | **Activated CD4 T cell** | **Activated CD8 T cell** | **Activated dendritic cell** | **CD56bright natural killer cell** | **CD56dim natural killer cell** | **Central memory CD4 T cell** | **Central memory CD8 T cell** | **Effector memeory CD4 T cell** | **Effector memeory CD8 T cell** | **Eosinophil** | **Gamma delta T cell** | **Immature B cell** | **Immature dendritic cell** | **Macrophage** | **Mast cell** | **MDSC** | **Memory B cell** | **Monocyte** | **Natural killer cell** | **Natural killer T cell** | **Neutrophil** | **Plasmacytoid dendritic cell** | **Regulatory T cell** | **T follicular helper cell** | **Type 1 T helper cell** | **Type 17 T helper cell** | **Type 2 T helper cell** |
| --- | --- | --- | --- | --- | --- | --- | --- | --- | --- | --- | --- | --- | --- | --- | --- | --- | --- | --- | --- | --- | --- | --- | --- | --- | --- | --- | --- | --- |
| Activated B cell | 0 | 0.916667 | 0.75 | 0.916667 | 0.916667 | 0.083333 | 0.75 | 0.75 | 0.083333 | 0.916667 | 0.75 | 0.333333 | 0.916667 | 0.083333 | 0.75 | 0.333333 | 0.916667 | 1 | 0.333333 | 0.333333 | 0.916667 | 0.333333 | 0.416667 | 0.916667 | 0.75 | 0.75 | 0.75 | 0.75 |
| Activated CD4 T cell | 0.916667 | 0 | 0.333333 | 0.083333 | 0.416667 | 0.916667 | 0.333333 | 0.333333 | 0.916667 | 0.416667 | 0.333333 | 0.75 | 0.083333 | 0.916667 | 0.333333 | 0.75 | 0.083333 | 0.75 | 0.75 | 0.75 | 0.416667 | 0.75 | 0.916667 | 0.083333 | 0.333333 | 0.333333 | 1 | 0.333333 |
| Activated CD8 T cell | 0.75 | 0.333333 | 0 | 0.333333 | 1 | 0.75 | 0.75 | 0.083333 | 0.75 | 1 | 0.75 | 0.916667 | 0.333333 | 0.75 | 0.083333 | 0.333333 | 0.333333 | 0.333333 | 0.916667 | 0.916667 | 1 | 0.916667 | 0.75 | 0.333333 | 0.75 | 0.75 | 0.416667 | 0.083333 |
| Activated dendritic cell | 0.916667 | 0.083333 | 0.333333 | 0 | 0.416667 | 0.916667 | 0.333333 | 0.333333 | 0.916667 | 0.416667 | 0.333333 | 0.75 | 0.083333 | 0.916667 | 0.333333 | 0.75 | 0.083333 | 0.75 | 0.75 | 0.75 | 0.416667 | 0.75 | 0.916667 | 0.083333 | 0.333333 | 0.333333 | 1 | 0.333333 |
| CD56bright natural killer cell | 0.916667 | 0.416667 | 1 | 0.416667 | 0 | 0.916667 | 0.333333 | 1 | 0.916667 | 0.083333 | 0.333333 | 0.75 | 0.416667 | 0.916667 | 1 | 0.75 | 0.416667 | 0.75 | 0.75 | 0.75 | 0.083333 | 0.75 | 0.916667 | 0.416667 | 0.333333 | 0.333333 | 0.333333 | 1 |
| CD56dim natural killer cell | 0.083333 | 0.916667 | 0.75 | 0.916667 | 0.916667 | 0 | 0.75 | 0.75 | 0.083333 | 0.916667 | 0.75 | 0.333333 | 0.916667 | 0.083333 | 0.75 | 0.333333 | 0.916667 | 1 | 0.333333 | 0.333333 | 0.916667 | 0.333333 | 0.416667 | 0.916667 | 0.75 | 0.75 | 0.75 | 0.75 |
| Central memory CD4 T cell | 0.75 | 0.333333 | 0.75 | 0.333333 | 0.333333 | 0.75 | 0 | 0.75 | 0.75 | 0.333333 | 0.083333 | 0.333333 | 0.333333 | 0.75 | 0.75 | 0.916667 | 0.333333 | 0.916667 | 1 | 1 | 0.333333 | 0.333333 | 0.75 | 0.333333 | 0.416667 | 0.083333 | 0.75 | 0.75 |
| Central memory CD8 T cell | 0.75 | 0.333333 | 0.083333 | 0.333333 | 1 | 0.75 | 0.75 | 0 | 0.75 | 1 | 0.75 | 0.916667 | 0.333333 | 0.75 | 0.083333 | 0.333333 | 0.333333 | 0.333333 | 0.916667 | 0.916667 | 1 | 0.916667 | 0.75 | 0.333333 | 0.75 | 0.75 | 0.416667 | 0.083333 |
| Effector memeory CD4 T cell | 0.083333 | 0.916667 | 0.75 | 0.916667 | 0.916667 | 0.083333 | 0.75 | 0.75 | 0 | 0.916667 | 0.75 | 0.333333 | 0.916667 | 0.083333 | 0.75 | 0.333333 | 0.916667 | 1 | 0.333333 | 0.333333 | 0.916667 | 0.333333 | 0.416667 | 0.916667 | 0.75 | 0.75 | 0.75 | 0.75 |
| Effector memeory CD8 T cell | 0.916667 | 0.416667 | 1 | 0.416667 | 0.083333 | 0.916667 | 0.333333 | 1 | 0.916667 | 0 | 0.333333 | 0.75 | 0.416667 | 0.916667 | 1 | 0.75 | 0.416667 | 0.75 | 0.75 | 0.75 | 0.083333 | 0.75 | 0.916667 | 0.416667 | 0.333333 | 0.333333 | 0.333333 | 1 |
| Eosinophil | 0.75 | 0.333333 | 0.75 | 0.333333 | 0.333333 | 0.75 | 0.083333 | 0.75 | 0.75 | 0.333333 | 0 | 0.333333 | 0.333333 | 0.75 | 0.75 | 0.916667 | 0.333333 | 0.916667 | 1 | 1 | 0.333333 | 0.333333 | 0.75 | 0.333333 | 0.416667 | 0.083333 | 0.75 | 0.75 |
| Gamma delta T cell | 0.333333 | 0.75 | 0.916667 | 0.75 | 0.75 | 0.333333 | 0.333333 | 0.916667 | 0.333333 | 0.75 | 0.333333 | 0 | 0.75 | 0.333333 | 0.916667 | 0.75 | 0.75 | 0.75 | 0.416667 | 0.416667 | 0.75 | 0.083333 | 0.333333 | 0.75 | 1 | 0.333333 | 0.916667 | 0.916667 |
| Immature B cell | 0.916667 | 0.083333 | 0.333333 | 0.083333 | 0.416667 | 0.916667 | 0.333333 | 0.333333 | 0.916667 | 0.416667 | 0.333333 | 0.75 | 0 | 0.916667 | 0.333333 | 0.75 | 0.083333 | 0.75 | 0.75 | 0.75 | 0.416667 | 0.75 | 0.916667 | 0.083333 | 0.333333 | 0.333333 | 1 | 0.333333 |
| Immature dendritic cell | 0.083333 | 0.916667 | 0.75 | 0.916667 | 0.916667 | 0.083333 | 0.75 | 0.75 | 0.083333 | 0.916667 | 0.75 | 0.333333 | 0.916667 | 0 | 0.75 | 0.333333 | 0.916667 | 1 | 0.333333 | 0.333333 | 0.916667 | 0.333333 | 0.416667 | 0.916667 | 0.75 | 0.75 | 0.75 | 0.75 |
| Macrophage | 0.75 | 0.333333 | 0.083333 | 0.333333 | 1 | 0.75 | 0.75 | 0.083333 | 0.75 | 1 | 0.75 | 0.916667 | 0.333333 | 0.75 | 0 | 0.333333 | 0.333333 | 0.333333 | 0.916667 | 0.916667 | 1 | 0.916667 | 0.75 | 0.333333 | 0.75 | 0.75 | 0.416667 | 0.083333 |
| Mast cell | 0.333333 | 0.75 | 0.333333 | 0.75 | 0.75 | 0.333333 | 0.916667 | 0.333333 | 0.333333 | 0.75 | 0.916667 | 0.75 | 0.75 | 0.333333 | 0.333333 | 0 | 0.75 | 0.416667 | 0.75 | 0.75 | 0.75 | 0.75 | 1 | 0.75 | 0.916667 | 0.916667 | 0.333333 | 0.333333 |
| MDSC | 0.916667 | 0.083333 | 0.333333 | 0.083333 | 0.416667 | 0.916667 | 0.333333 | 0.333333 | 0.916667 | 0.416667 | 0.333333 | 0.75 | 0.083333 | 0.916667 | 0.333333 | 0.75 | 0 | 0.75 | 0.75 | 0.75 | 0.416667 | 0.75 | 0.916667 | 0.083333 | 0.333333 | 0.333333 | 1 | 0.333333 |
| Memory B cell | 1 | 0.75 | 0.333333 | 0.75 | 0.75 | 1 | 0.916667 | 0.333333 | 1 | 0.75 | 0.916667 | 0.75 | 0.75 | 1 | 0.333333 | 0.416667 | 0.75 | 0 | 0.75 | 0.75 | 0.75 | 0.75 | 0.333333 | 0.75 | 0.916667 | 0.916667 | 0.333333 | 0.333333 |
| Monocyte | 0.333333 | 0.75 | 0.916667 | 0.75 | 0.75 | 0.333333 | 1 | 0.916667 | 0.333333 | 0.75 | 1 | 0.416667 | 0.75 | 0.333333 | 0.916667 | 0.75 | 0.75 | 0.75 | 0 | 0.083333 | 0.75 | 0.416667 | 0.333333 | 0.75 | 0.333333 | 1 | 0.916667 | 0.916667 |
| Natural killer cell | 0.333333 | 0.75 | 0.916667 | 0.75 | 0.75 | 0.333333 | 1 | 0.916667 | 0.333333 | 0.75 | 1 | 0.416667 | 0.75 | 0.333333 | 0.916667 | 0.75 | 0.75 | 0.75 | 0.083333 | 0 | 0.75 | 0.416667 | 0.333333 | 0.75 | 0.333333 | 1 | 0.916667 | 0.916667 |
| Natural killer T cell | 0.916667 | 0.416667 | 1 | 0.416667 | 0.083333 | 0.916667 | 0.333333 | 1 | 0.916667 | 0.083333 | 0.333333 | 0.75 | 0.416667 | 0.916667 | 1 | 0.75 | 0.416667 | 0.75 | 0.75 | 0.75 | 0 | 0.75 | 0.916667 | 0.416667 | 0.333333 | 0.333333 | 0.333333 | 1 |
| Neutrophil | 0.333333 | 0.75 | 0.916667 | 0.75 | 0.75 | 0.333333 | 0.333333 | 0.916667 | 0.333333 | 0.75 | 0.333333 | 0.083333 | 0.75 | 0.333333 | 0.916667 | 0.75 | 0.75 | 0.75 | 0.416667 | 0.416667 | 0.75 | 0 | 0.333333 | 0.75 | 1 | 0.333333 | 0.916667 | 0.916667 |
| Plasmacytoid dendritic cell | 0.416667 | 0.916667 | 0.75 | 0.916667 | 0.916667 | 0.416667 | 0.75 | 0.75 | 0.416667 | 0.916667 | 0.75 | 0.333333 | 0.916667 | 0.416667 | 0.75 | 1 | 0.916667 | 0.333333 | 0.333333 | 0.333333 | 0.916667 | 0.333333 | 0 | 0.916667 | 0.75 | 0.75 | 0.75 | 0.75 |
| Regulatory T cell | 0.916667 | 0.083333 | 0.333333 | 0.083333 | 0.416667 | 0.916667 | 0.333333 | 0.333333 | 0.916667 | 0.416667 | 0.333333 | 0.75 | 0.083333 | 0.916667 | 0.333333 | 0.75 | 0.083333 | 0.75 | 0.75 | 0.75 | 0.416667 | 0.75 | 0.916667 | 0 | 0.333333 | 0.333333 | 1 | 0.333333 |
| T follicular helper cell | 0.75 | 0.333333 | 0.75 | 0.333333 | 0.333333 | 0.75 | 0.416667 | 0.75 | 0.75 | 0.333333 | 0.416667 | 1 | 0.333333 | 0.75 | 0.75 | 0.916667 | 0.333333 | 0.916667 | 0.333333 | 0.333333 | 0.333333 | 1 | 0.75 | 0.333333 | 0 | 0.416667 | 0.75 | 0.75 |
| Type 1 T helper cell | 0.75 | 0.333333 | 0.75 | 0.333333 | 0.333333 | 0.75 | 0.083333 | 0.75 | 0.75 | 0.333333 | 0.083333 | 0.333333 | 0.333333 | 0.75 | 0.75 | 0.916667 | 0.333333 | 0.916667 | 1 | 1 | 0.333333 | 0.333333 | 0.75 | 0.333333 | 0.416667 | 0 | 0.75 | 0.75 |
| Type 17 T helper cell | 0.75 | 1 | 0.416667 | 1 | 0.333333 | 0.75 | 0.75 | 0.416667 | 0.75 | 0.333333 | 0.75 | 0.916667 | 1 | 0.75 | 0.416667 | 0.333333 | 1 | 0.333333 | 0.916667 | 0.916667 | 0.333333 | 0.916667 | 0.75 | 1 | 0.75 | 0.75 | 0 | 0.416667 |
| Type 2 T helper cell | 0.75 | 0.333333 | 0.083333 | 0.333333 | 1 | 0.75 | 0.75 | 0.083333 | 0.75 | 1 | 0.75 | 0.916667 | 0.333333 | 0.75 | 0.083333 | 0.333333 | 0.333333 | 0.333333 | 0.916667 | 0.916667 | 1 | 0.916667 | 0.75 | 0.333333 | 0.75 | 0.75 | 0.416667 | 0 |

# Supplementary Table S16 (Sheet 11). Correlations between HMOX1/TLR4 and immune cell infiltration (stage-specific)

| **Gene** | **TimePoint** | **ImmuneCell** | **Correlation_r** | **P_value** |
| --- | --- | --- | --- | --- |
| HMOX1 | SCI_1d | Activated dendritic cell | 0.904762 | 0.004563 |
| HMOX1 | SCI_1d | Natural killer cell | 0.880952 | 0.007242 |
| HMOX1 | SCI_1d | Effector memeory CD8 T cell | 0.857143 | 0.010714 |
| HMOX1 | SCI_1d | Regulatory T cell | 0.857143 | 0.010714 |
| HMOX1 | SCI_1d | Central memory CD4 T cell | 0.833333 | 0.015377 |
| HMOX1 | SCI_1d | Gamma delta T cell | 0.833333 | 0.015377 |
| HMOX1 | SCI_1d | MDSC | 0.809524 | 0.021776 |
| HMOX1 | SCI_1d | Central memory CD8 T cell | 0.785714 | 0.027927 |
| HMOX1 | SCI_1d | Plasmacytoid dendritic cell | 0.785714 | 0.027927 |
| HMOX1 | SCI_1d | Memory B cell | 0.761905 | 0.036756 |
| HMOX1 | SCI_1d | T follicular helper cell | 0.761905 | 0.036756 |
| HMOX1 | SCI_1d | Activated CD4 T cell | 0.666667 | 0.083085 |
| HMOX1 | SCI_1d | Immature B cell | 0.666667 | 0.083085 |
| HMOX1 | SCI_1d | Macrophage | 0.666667 | 0.083085 |
| HMOX1 | SCI_1d | Natural killer T cell | 0.666667 | 0.083085 |
| HMOX1 | SCI_1d | Type 1 T helper cell | 0.642857 | 0.096181 |
| HMOX1 | SCI_1d | Activated CD8 T cell | 0.595238 | 0.132292 |
| HMOX1 | SCI_1d | Monocyte | 0.52381 | 0.196627 |
| HMOX1 | SCI_1d | Activated B cell | 0.452381 | 0.26746 |
| HMOX1 | SCI_1d | Immature dendritic cell | 0.333333 | 0.427877 |
| HMOX1 | SCI_1d | Effector memeory CD4 T cell | 0.309524 | 0.461806 |
| HMOX1 | SCI_1d | Mast cell | 0.166667 | 0.703323 |
| HMOX1 | SCI_1d | Type 2 T helper cell | -0.02381 | 0.976786 |
| HMOX1 | SCI_1d | Type 17 T helper cell | -0.190476 | 0.664583 |
| HMOX1 | SCI_1d | CD56dim natural killer cell | -0.5 | 0.216171 |
| HMOX1 | SCI_1d | Eosinophil | -0.52381 | 0.196627 |
| HMOX1 | SCI_1d | Neutrophil | -0.547619 | 0.170982 |
| HMOX1 | SCI_1d | CD56bright natural killer cell | -0.619048 | 0.11498 |
| HMOX1 | SCI_3d | Activated dendritic cell | 0.928571 | 0.002232 |
| HMOX1 | SCI_3d | Central memory CD4 T cell | 0.904762 | 0.004563 |
| HMOX1 | SCI_3d | Gamma delta T cell | 0.904762 | 0.004563 |
| HMOX1 | SCI_3d | Natural killer cell | 0.880952 | 0.007242 |
| HMOX1 | SCI_3d | MDSC | 0.857143 | 0.010714 |
| HMOX1 | SCI_3d | Regulatory T cell | 0.833333 | 0.015377 |
| HMOX1 | SCI_3d | Activated CD4 T cell | 0.809524 | 0.021776 |
| HMOX1 | SCI_3d | Memory B cell | 0.714286 | 0.057589 |
| HMOX1 | SCI_3d | Central memory CD8 T cell | 0.690476 | 0.069395 |
| HMOX1 | SCI_3d | Type 2 T helper cell | 0.690476 | 0.069395 |
| HMOX1 | SCI_3d | Effector memeory CD4 T cell | 0.666667 | 0.083085 |
| HMOX1 | SCI_3d | Plasmacytoid dendritic cell | 0.619048 | 0.11498 |
| HMOX1 | SCI_3d | T follicular helper cell | 0.380952 | 0.359871 |
| HMOX1 | SCI_3d | Immature dendritic cell | 0.285714 | 0.500794 |
| HMOX1 | SCI_3d | Immature B cell | 0.261905 | 0.536409 |
| HMOX1 | SCI_3d | Monocyte | 0.190476 | 0.664583 |
| HMOX1 | SCI_3d | Natural killer T cell | 0.190476 | 0.664583 |
| HMOX1 | SCI_3d | Activated CD8 T cell | 0.166667 | 0.703323 |
| HMOX1 | SCI_3d | Macrophage | 0.047619 | 0.934871 |
| HMOX1 | SCI_3d | Type 1 T helper cell | -0.119048 | 0.793006 |
| HMOX1 | SCI_3d | CD56bright natural killer cell | -0.238095 | 0.582143 |
| HMOX1 | SCI_3d | Eosinophil | -0.309524 | 0.461806 |
| HMOX1 | SCI_3d | CD56dim natural killer cell | -0.380952 | 0.359871 |
| HMOX1 | SCI_3d | Effector memeory CD8 T cell | -0.380952 | 0.359871 |
| HMOX1 | SCI_3d | Neutrophil | -0.642857 | 0.096181 |
| HMOX1 | SCI_3d | Type 17 T helper cell | -0.785714 | 0.027927 |
| HMOX1 | SCI_3d | Activated B cell | -0.880952 | 0.007242 |
| HMOX1 | SCI_3d | Mast cell | -0.952381 | 0.001141 |
| HMOX1 | SCI_7d | Activated dendritic cell | 0.928571 | 0.002232 |
| HMOX1 | SCI_7d | Regulatory T cell | 0.928571 | 0.002232 |
| HMOX1 | SCI_7d | Central memory CD4 T cell | 0.904762 | 0.004563 |
| HMOX1 | SCI_7d | Central memory CD8 T cell | 0.904762 | 0.004563 |
| HMOX1 | SCI_7d | MDSC | 0.880952 | 0.007242 |
| HMOX1 | SCI_7d | Plasmacytoid dendritic cell | 0.880952 | 0.007242 |
| HMOX1 | SCI_7d | T follicular helper cell | 0.880952 | 0.007242 |
| HMOX1 | SCI_7d | Monocyte | 0.857143 | 0.010714 |
| HMOX1 | SCI_7d | Activated CD4 T cell | 0.833333 | 0.015377 |
| HMOX1 | SCI_7d | CD56bright natural killer cell | 0.833333 | 0.015377 |
| HMOX1 | SCI_7d | Effector memeory CD4 T cell | 0.833333 | 0.015377 |
| HMOX1 | SCI_7d | Gamma delta T cell | 0.833333 | 0.015377 |
| HMOX1 | SCI_7d | Immature dendritic cell | 0.833333 | 0.015377 |
| HMOX1 | SCI_7d | Natural killer cell | 0.833333 | 0.015377 |
| HMOX1 | SCI_7d | Immature B cell | 0.785714 | 0.027927 |
| HMOX1 | SCI_7d | Macrophage | 0.785714 | 0.027927 |
| HMOX1 | SCI_7d | Natural killer T cell | 0.785714 | 0.027927 |
| HMOX1 | SCI_7d | Type 1 T helper cell | 0.761905 | 0.036756 |
| HMOX1 | SCI_7d | Memory B cell | 0.666667 | 0.083085 |
| HMOX1 | SCI_7d | Effector memeory CD8 T cell | 0.52381 | 0.196627 |
| HMOX1 | SCI_7d | Activated CD8 T cell | 0.47619 | 0.243056 |
| HMOX1 | SCI_7d | CD56dim natural killer cell | 0.404762 | 0.326835 |
| HMOX1 | SCI_7d | Activated B cell | 0.309524 | 0.461806 |
| HMOX1 | SCI_7d | Mast cell | 0.285714 | 0.500794 |
| HMOX1 | SCI_7d | Neutrophil | -0.333333 | 0.427877 |
| HMOX1 | SCI_7d | Eosinophil | -0.547619 | 0.170982 |
| HMOX1 | SCI_7d | Type 17 T helper cell | -0.547619 | 0.170982 |
| HMOX1 | SCI_7d | Type 2 T helper cell | -0.571429 | 0.151141 |
| TLR4 | SCI_1d | T follicular helper cell | 0.952381 | 0.001141 |
| TLR4 | SCI_1d | Central memory CD8 T cell | 0.928571 | 0.002232 |
| TLR4 | SCI_1d | Regulatory T cell | 0.928571 | 0.002232 |
| TLR4 | SCI_1d | Gamma delta T cell | 0.904762 | 0.004563 |
| TLR4 | SCI_1d | MDSC | 0.904762 | 0.004563 |
| TLR4 | SCI_1d | Central memory CD4 T cell | 0.880952 | 0.007242 |
| TLR4 | SCI_1d | Immature B cell | 0.880952 | 0.007242 |
| TLR4 | SCI_1d | Natural killer cell | 0.880952 | 0.007242 |
| TLR4 | SCI_1d | Monocyte | 0.857143 | 0.010714 |
| TLR4 | SCI_1d | Activated CD4 T cell | 0.833333 | 0.015377 |
| TLR4 | SCI_1d | Natural killer T cell | 0.785714 | 0.027927 |
| TLR4 | SCI_1d | Plasmacytoid dendritic cell | 0.761905 | 0.036756 |
| TLR4 | SCI_1d | Type 1 T helper cell | 0.761905 | 0.036756 |
| TLR4 | SCI_1d | Activated dendritic cell | 0.738095 | 0.045833 |
| TLR4 | SCI_1d | Activated B cell | 0.714286 | 0.057589 |
| TLR4 | SCI_1d | Macrophage | 0.666667 | 0.083085 |
| TLR4 | SCI_1d | Memory B cell | 0.642857 | 0.096181 |
| TLR4 | SCI_1d | Effector memeory CD8 T cell | 0.619048 | 0.11498 |
| TLR4 | SCI_1d | Activated CD8 T cell | 0.547619 | 0.170982 |
| TLR4 | SCI_1d | Immature dendritic cell | 0.52381 | 0.196627 |
| TLR4 | SCI_1d | Effector memeory CD4 T cell | 0.404762 | 0.326835 |
| TLR4 | SCI_1d | Mast cell | 0.380952 | 0.359871 |
| TLR4 | SCI_1d | Type 2 T helper cell | 0.190476 | 0.664583 |
| TLR4 | SCI_1d | CD56dim natural killer cell | -0.333333 | 0.427877 |
| TLR4 | SCI_1d | Eosinophil | -0.333333 | 0.427877 |
| TLR4 | SCI_1d | Type 17 T helper cell | -0.333333 | 0.427877 |
| TLR4 | SCI_1d | Neutrophil | -0.404762 | 0.326835 |
| TLR4 | SCI_1d | CD56bright natural killer cell | -0.452381 | 0.26746 |
| TLR4 | SCI_3d | Central memory CD4 T cell | 0.97619 | 3.968E-04 |
| TLR4 | SCI_3d | Gamma delta T cell | 0.97619 | 3.968E-04 |
| TLR4 | SCI_3d | Activated dendritic cell | 0.952381 | 0.001141 |
| TLR4 | SCI_3d | MDSC | 0.952381 | 0.001141 |
| TLR4 | SCI_3d | Natural killer cell | 0.952381 | 0.001141 |
| TLR4 | SCI_3d | Regulatory T cell | 0.952381 | 0.001141 |
| TLR4 | SCI_3d | Activated CD4 T cell | 0.809524 | 0.021776 |
| TLR4 | SCI_3d | Type 2 T helper cell | 0.785714 | 0.027927 |
| TLR4 | SCI_3d | Central memory CD8 T cell | 0.761905 | 0.036756 |
| TLR4 | SCI_3d | Memory B cell | 0.714286 | 0.057589 |
| TLR4 | SCI_3d | Plasmacytoid dendritic cell | 0.642857 | 0.096181 |
| TLR4 | SCI_3d | Effector memeory CD4 T cell | 0.571429 | 0.151141 |
| TLR4 | SCI_3d | T follicular helper cell | 0.52381 | 0.196627 |
| TLR4 | SCI_3d | Immature B cell | 0.428571 | 0.299206 |
| TLR4 | SCI_3d | Immature dendritic cell | 0.309524 | 0.461806 |
| TLR4 | SCI_3d | Macrophage | 0.261905 | 0.536409 |
| TLR4 | SCI_3d | Monocyte | 0.261905 | 0.536409 |
| TLR4 | SCI_3d | Natural killer T cell | 0.166667 | 0.703323 |
| TLR4 | SCI_3d | Activated CD8 T cell | 0.047619 | 0.934871 |
| TLR4 | SCI_3d | Type 1 T helper cell | -0.119048 | 0.793006 |
| TLR4 | SCI_3d | Effector memeory CD8 T cell | -0.261905 | 0.536409 |
| TLR4 | SCI_3d | CD56bright natural killer cell | -0.333333 | 0.427877 |
| TLR4 | SCI_3d | Eosinophil | -0.333333 | 0.427877 |
| TLR4 | SCI_3d | Neutrophil | -0.452381 | 0.26746 |
| TLR4 | SCI_3d | CD56dim natural killer cell | -0.47619 | 0.243056 |
| TLR4 | SCI_3d | Activated B cell | -0.880952 | 0.007242 |
| TLR4 | SCI_3d | Type 17 T helper cell | -0.880952 | 0.007242 |
| TLR4 | SCI_3d | Mast cell | -0.952381 | 0.001141 |
| TLR4 | SCI_7d | Regulatory T cell | 1 | 4.960E-05 |
| TLR4 | SCI_7d | Central memory CD4 T cell | 0.97619 | 3.968E-04 |
| TLR4 | SCI_7d | MDSC | 0.97619 | 3.968E-04 |
| TLR4 | SCI_7d | Activated dendritic cell | 0.952381 | 0.001141 |
| TLR4 | SCI_7d | T follicular helper cell | 0.952381 | 0.001141 |
| TLR4 | SCI_7d | Central memory CD8 T cell | 0.928571 | 0.002232 |
| TLR4 | SCI_7d | Monocyte | 0.928571 | 0.002232 |
| TLR4 | SCI_7d | Gamma delta T cell | 0.904762 | 0.004563 |
| TLR4 | SCI_7d | Immature B cell | 0.904762 | 0.004563 |
| TLR4 | SCI_7d | Macrophage | 0.904762 | 0.004563 |
| TLR4 | SCI_7d | Natural killer cell | 0.904762 | 0.004563 |
| TLR4 | SCI_7d | Plasmacytoid dendritic cell | 0.880952 | 0.007242 |
| TLR4 | SCI_7d | Activated CD4 T cell | 0.833333 | 0.015377 |
| TLR4 | SCI_7d | Immature dendritic cell | 0.833333 | 0.015377 |
| TLR4 | SCI_7d | Natural killer T cell | 0.785714 | 0.027927 |
| TLR4 | SCI_7d | Effector memeory CD4 T cell | 0.761905 | 0.036756 |
| TLR4 | SCI_7d | Type 1 T helper cell | 0.761905 | 0.036756 |
| TLR4 | SCI_7d | CD56bright natural killer cell | 0.738095 | 0.045833 |
| TLR4 | SCI_7d | Memory B cell | 0.666667 | 0.083085 |
| TLR4 | SCI_7d | Effector memeory CD8 T cell | 0.595238 | 0.132292 |
| TLR4 | SCI_7d | Activated CD8 T cell | 0.404762 | 0.326835 |
| TLR4 | SCI_7d | Activated B cell | 0.309524 | 0.461806 |
| TLR4 | SCI_7d | CD56dim natural killer cell | 0.309524 | 0.461806 |
| TLR4 | SCI_7d | Mast cell | 0.285714 | 0.500794 |
| TLR4 | SCI_7d | Neutrophil | -0.190476 | 0.664583 |
| TLR4 | SCI_7d | Type 2 T helper cell | -0.380952 | 0.359871 |
| TLR4 | SCI_7d | Eosinophil | -0.5 | 0.216171 |
| TLR4 | SCI_7d | Type 17 T helper cell | -0.714286 | 0.057589 |

# Supplementary Table S16. Immune cell correlations in long format (all samples)

| **Context** | **CellA** | **CellB** | **Correlation_r** | **P_value** |
| --- | --- | --- | --- | --- |
| All_timepoints | Activated B cell | Activated CD4 T cell | 0.344118 | 0.191942 |
| All_timepoints | Activated B cell | Activated CD8 T cell | 0.6 | 0.01597 |
| All_timepoints | Activated B cell | Activated dendritic cell | 0.255882 | 0.337574 |
| All_timepoints | Activated B cell | CD56bright natural killer cell | 0.126471 | 0.640554 |
| All_timepoints | Activated B cell | CD56dim natural killer cell | -0.017647 | 0.951949 |
| All_timepoints | Activated B cell | Central memory CD4 T cell | 0.152941 | 0.57102 |
| All_timepoints | Activated B cell | Central memory CD8 T cell | 0.282353 | 0.288413 |
| All_timepoints | Activated B cell | Effector memeory CD4 T cell | -0.047059 | 0.865106 |
| All_timepoints | Activated B cell | Effector memeory CD8 T cell | 0.447059 | 0.084373 |
| All_timepoints | Activated B cell | Eosinophil | 0.126471 | 0.640554 |
| All_timepoints | Activated B cell | Gamma delta T cell | 0.529412 | 0.037277 |
| All_timepoints | Activated B cell | Immature B cell | 0.5 | 0.050852 |
| All_timepoints | Activated B cell | Immature dendritic cell | 0.258824 | 0.33188 |
| All_timepoints | Activated B cell | Macrophage | 0.447059 | 0.084373 |
| All_timepoints | Activated B cell | Mast cell | 0.935294 | 0 |
| All_timepoints | Activated B cell | MDSC | 0.261765 | 0.326245 |
| All_timepoints | Activated B cell | Memory B cell | 0.258824 | 0.33188 |
| All_timepoints | Activated B cell | Monocyte | 0.394118 | 0.131937 |
| All_timepoints | Activated B cell | Natural killer cell | 0.320588 | 0.225701 |
| All_timepoints | Activated B cell | Natural killer T cell | 0.570588 | 0.023176 |
| All_timepoints | Activated B cell | Neutrophil | 0.144118 | 0.593829 |
| All_timepoints | Activated B cell | Plasmacytoid dendritic cell | 0.305882 | 0.248661 |
| All_timepoints | Activated B cell | Regulatory T cell | 0.305882 | 0.248661 |
| All_timepoints | Activated B cell | T follicular helper cell | 0.385294 | 0.141409 |
| All_timepoints | Activated B cell | Type 1 T helper cell | 0.641176 | 0.008975 |
| All_timepoints | Activated B cell | Type 17 T helper cell | 0.470588 | 0.067902 |
| All_timepoints | Activated B cell | Type 2 T helper cell | -0.585294 | 0.01931 |
| All_timepoints | Activated CD4 T cell | Activated CD8 T cell | 0.729412 | 0.001927 |
| All_timepoints | Activated CD4 T cell | Activated dendritic cell | 0.844118 | 1.932E-05 |
| All_timepoints | Activated CD4 T cell | CD56bright natural killer cell | 0.011765 | 0.969412 |
| All_timepoints | Activated CD4 T cell | CD56dim natural killer cell | -0.261765 | 0.326245 |
| All_timepoints | Activated CD4 T cell | Central memory CD4 T cell | 0.747059 | 0.001314 |
| All_timepoints | Activated CD4 T cell | Central memory CD8 T cell | 0.735294 | 0.001703 |
| All_timepoints | Activated CD4 T cell | Effector memeory CD4 T cell | 0.208824 | 0.436322 |
| All_timepoints | Activated CD4 T cell | Effector memeory CD8 T cell | 0.402941 | 0.122925 |
| All_timepoints | Activated CD4 T cell | Eosinophil | -0.223529 | 0.403941 |
| All_timepoints | Activated CD4 T cell | Gamma delta T cell | 0.808824 | 2.195E-04 |
| All_timepoints | Activated CD4 T cell | Immature B cell | 0.644118 | 0.00859 |
| All_timepoints | Activated CD4 T cell | Immature dendritic cell | 0.294118 | 0.268072 |
| All_timepoints | Activated CD4 T cell | Macrophage | 0.461765 | 0.073768 |
| All_timepoints | Activated CD4 T cell | Mast cell | 0.25 | 0.349132 |
| All_timepoints | Activated CD4 T cell | MDSC | 0.847059 | 1.023E-05 |
| All_timepoints | Activated CD4 T cell | Memory B cell | 0.767647 | 7.980E-04 |
| All_timepoints | Activated CD4 T cell | Monocyte | 0.367647 | 0.161766 |
| All_timepoints | Activated CD4 T cell | Natural killer cell | 0.688235 | 0.00421 |
| All_timepoints | Activated CD4 T cell | Natural killer T cell | 0.691176 | 0.003999 |
| All_timepoints | Activated CD4 T cell | Neutrophil | -0.085294 | 0.75456 |
| All_timepoints | Activated CD4 T cell | Plasmacytoid dendritic cell | 0.529412 | 0.037277 |
| All_timepoints | Activated CD4 T cell | Regulatory T cell | 0.664706 | 0.006241 |
| All_timepoints | Activated CD4 T cell | T follicular helper cell | 0.441176 | 0.088915 |
| All_timepoints | Activated CD4 T cell | Type 1 T helper cell | 0.705882 | 0.003062 |
| All_timepoints | Activated CD4 T cell | Type 17 T helper cell | -0.170588 | 0.526599 |
| All_timepoints | Activated CD4 T cell | Type 2 T helper cell | 0.020588 | 0.943224 |
| All_timepoints | Activated CD8 T cell | Activated dendritic cell | 0.608824 | 0.014196 |
| All_timepoints | Activated CD8 T cell | CD56bright natural killer cell | 0.132353 | 0.624821 |
| All_timepoints | Activated CD8 T cell | CD56dim natural killer cell | -0.041176 | 0.882386 |
| All_timepoints | Activated CD8 T cell | Central memory CD4 T cell | 0.297059 | 0.263132 |
| All_timepoints | Activated CD8 T cell | Central memory CD8 T cell | 0.408824 | 0.117166 |
| All_timepoints | Activated CD8 T cell | Effector memeory CD4 T cell | -0.176471 | 0.512164 |
| All_timepoints | Activated CD8 T cell | Effector memeory CD8 T cell | 0.444118 | 0.086622 |
| All_timepoints | Activated CD8 T cell | Eosinophil | 0.164706 | 0.541224 |
| All_timepoints | Activated CD8 T cell | Gamma delta T cell | 0.602941 | 0.01536 |
| All_timepoints | Activated CD8 T cell | Immature B cell | 0.6 | 0.01597 |
| All_timepoints | Activated CD8 T cell | Immature dendritic cell | -0.008824 | 0.978149 |
| All_timepoints | Activated CD8 T cell | Macrophage | 0.429412 | 0.098537 |
| All_timepoints | Activated CD8 T cell | Mast cell | 0.588235 | 0.018602 |
| All_timepoints | Activated CD8 T cell | MDSC | 0.582353 | 0.02004 |
| All_timepoints | Activated CD8 T cell | Memory B cell | 0.532353 | 0.036091 |
| All_timepoints | Activated CD8 T cell | Monocyte | 0.211765 | 0.429738 |
| All_timepoints | Activated CD8 T cell | Natural killer cell | 0.491176 | 0.05558 |
| All_timepoints | Activated CD8 T cell | Natural killer T cell | 0.820588 | 1.308E-04 |
| All_timepoints | Activated CD8 T cell | Neutrophil | 0.167647 | 0.533888 |
| All_timepoints | Activated CD8 T cell | Plasmacytoid dendritic cell | 0.202941 | 0.449649 |
| All_timepoints | Activated CD8 T cell | Regulatory T cell | 0.447059 | 0.084373 |
| All_timepoints | Activated CD8 T cell | T follicular helper cell | 0.294118 | 0.268072 |
| All_timepoints | Activated CD8 T cell | Type 1 T helper cell | 0.655882 | 0.007175 |
| All_timepoints | Activated CD8 T cell | Type 17 T helper cell | 0.305882 | 0.248661 |
| All_timepoints | Activated CD8 T cell | Type 2 T helper cell | -0.420588 | 0.106235 |
| All_timepoints | Activated dendritic cell | CD56bright natural killer cell | 0.023529 | 0.934507 |
| All_timepoints | Activated dendritic cell | CD56dim natural killer cell | -0.255882 | 0.337574 |
| All_timepoints | Activated dendritic cell | Central memory CD4 T cell | 0.861765 | 0 |
| All_timepoints | Activated dendritic cell | Central memory CD8 T cell | 0.823529 | 1.125E-04 |
| All_timepoints | Activated dendritic cell | Effector memeory CD4 T cell | 0.4 | 0.125879 |
| All_timepoints | Activated dendritic cell | Effector memeory CD8 T cell | 0.588235 | 0.018602 |
| All_timepoints | Activated dendritic cell | Eosinophil | -0.385294 | 0.141409 |
| All_timepoints | Activated dendritic cell | Gamma delta T cell | 0.894118 | 0 |
| All_timepoints | Activated dendritic cell | Immature B cell | 0.767647 | 7.980E-04 |
| All_timepoints | Activated dendritic cell | Immature dendritic cell | 0.485294 | 0.058912 |
| All_timepoints | Activated dendritic cell | Macrophage | 0.732353 | 0.001812 |
| All_timepoints | Activated dendritic cell | Mast cell | 0.164706 | 0.541224 |
| All_timepoints | Activated dendritic cell | MDSC | 0.926471 | 0 |
| All_timepoints | Activated dendritic cell | Memory B cell | 0.788235 | 4.474E-04 |
| All_timepoints | Activated dendritic cell | Monocyte | 0.597059 | 0.016599 |
| All_timepoints | Activated dendritic cell | Natural killer cell | 0.855882 | 0 |
| All_timepoints | Activated dendritic cell | Natural killer T cell | 0.729412 | 0.001927 |
| All_timepoints | Activated dendritic cell | Neutrophil | -0.176471 | 0.512164 |
| All_timepoints | Activated dendritic cell | Plasmacytoid dendritic cell | 0.670588 | 0.005673 |
| All_timepoints | Activated dendritic cell | Regulatory T cell | 0.858824 | 0 |
| All_timepoints | Activated dendritic cell | T follicular helper cell | 0.679412 | 0.004899 |
| All_timepoints | Activated dendritic cell | Type 1 T helper cell | 0.741176 | 0.001499 |
| All_timepoints | Activated dendritic cell | Type 17 T helper cell | -0.264706 | 0.320666 |
| All_timepoints | Activated dendritic cell | Type 2 T helper cell | -0.135294 | 0.617013 |
| All_timepoints | CD56bright natural killer cell | CD56dim natural killer cell | 0.379412 | 0.147983 |
| All_timepoints | CD56bright natural killer cell | Central memory CD4 T cell | 0.032353 | 0.908401 |
| All_timepoints | CD56bright natural killer cell | Central memory CD8 T cell | 0.182353 | 0.497921 |
| All_timepoints | CD56bright natural killer cell | Effector memeory CD4 T cell | 0.258824 | 0.33188 |
| All_timepoints | CD56bright natural killer cell | Effector memeory CD8 T cell | -0.208824 | 0.436322 |
| All_timepoints | CD56bright natural killer cell | Eosinophil | 0.479412 | 0.062393 |
| All_timepoints | CD56bright natural killer cell | Gamma delta T cell | 0.020588 | 0.943224 |
| All_timepoints | CD56bright natural killer cell | Immature B cell | 0.376471 | 0.151349 |
| All_timepoints | CD56bright natural killer cell | Immature dendritic cell | 0.126471 | 0.640554 |
| All_timepoints | CD56bright natural killer cell | Macrophage | -0.1 | 0.71313 |
| All_timepoints | CD56bright natural killer cell | Mast cell | 0.232353 | 0.385168 |
| All_timepoints | CD56bright natural killer cell | MDSC | -0.117647 | 0.664435 |
| All_timepoints | CD56bright natural killer cell | Memory B cell | -0.064706 | 0.813651 |
| All_timepoints | CD56bright natural killer cell | Monocyte | 0.238235 | 0.372931 |
| All_timepoints | CD56bright natural killer cell | Natural killer cell | -0.041176 | 0.882386 |
| All_timepoints | CD56bright natural killer cell | Natural killer T cell | 0.264706 | 0.320666 |
| All_timepoints | CD56bright natural killer cell | Neutrophil | 0.438235 | 0.091253 |
| All_timepoints | CD56bright natural killer cell | Plasmacytoid dendritic cell | 0.117647 | 0.664435 |
| All_timepoints | CD56bright natural killer cell | Regulatory T cell | 0.176471 | 0.512164 |
| All_timepoints | CD56bright natural killer cell | T follicular helper cell | 0.267647 | 0.315146 |
| All_timepoints | CD56bright natural killer cell | Type 1 T helper cell | 0.105882 | 0.696767 |
| All_timepoints | CD56bright natural killer cell | Type 17 T helper cell | -0.108824 | 0.688634 |
| All_timepoints | CD56bright natural killer cell | Type 2 T helper cell | -0.447059 | 0.084373 |
| All_timepoints | CD56dim natural killer cell | Central memory CD4 T cell | -0.091176 | 0.737903 |
| All_timepoints | CD56dim natural killer cell | Central memory CD8 T cell | -0.238235 | 0.372931 |
| All_timepoints | CD56dim natural killer cell | Effector memeory CD4 T cell | 0.347059 | 0.187976 |
| All_timepoints | CD56dim natural killer cell | Effector memeory CD8 T cell | -0.273529 | 0.304279 |
| All_timepoints | CD56dim natural killer cell | Eosinophil | 0.329412 | 0.212614 |
| All_timepoints | CD56dim natural killer cell | Gamma delta T cell | -0.147059 | 0.586183 |
| All_timepoints | CD56dim natural killer cell | Immature B cell | 0.085294 | 0.75456 |
| All_timepoints | CD56dim natural killer cell | Immature dendritic cell | 0.014706 | 0.960678 |
| All_timepoints | CD56dim natural killer cell | Macrophage | -0.014706 | 0.960678 |
| All_timepoints | CD56dim natural killer cell | Mast cell | 0.067647 | 0.805142 |
| All_timepoints | CD56dim natural killer cell | MDSC | -0.188235 | 0.483876 |
| All_timepoints | CD56dim natural killer cell | Memory B cell | -0.085294 | 0.75456 |
| All_timepoints | CD56dim natural killer cell | Monocyte | 0.05 | 0.856487 |
| All_timepoints | CD56dim natural killer cell | Natural killer cell | 0.005882 | 0.986888 |
| All_timepoints | CD56dim natural killer cell | Natural killer T cell | 0.138235 | 0.609244 |
| All_timepoints | CD56dim natural killer cell | Neutrophil | 0.032353 | 0.908401 |
| All_timepoints | CD56dim natural killer cell | Plasmacytoid dendritic cell | 0.005882 | 0.986888 |
| All_timepoints | CD56dim natural killer cell | Regulatory T cell | 0.058824 | 0.830731 |
| All_timepoints | CD56dim natural killer cell | T follicular helper cell | 0.135294 | 0.617013 |
| All_timepoints | CD56dim natural killer cell | Type 1 T helper cell | 0.058824 | 0.830731 |
| All_timepoints | CD56dim natural killer cell | Type 17 T helper cell | -0.026471 | 0.925796 |
| All_timepoints | CD56dim natural killer cell | Type 2 T helper cell | -0.073529 | 0.788187 |
| All_timepoints | Central memory CD4 T cell | Central memory CD8 T cell | 0.852941 | 0 |
| All_timepoints | Central memory CD4 T cell | Effector memeory CD4 T cell | 0.726471 | 0.002048 |
| All_timepoints | Central memory CD4 T cell | Effector memeory CD8 T cell | 0.370588 | 0.15824 |
| All_timepoints | Central memory CD4 T cell | Eosinophil | -0.455882 | 0.077883 |
| All_timepoints | Central memory CD4 T cell | Gamma delta T cell | 0.847059 | 1.023E-05 |
| All_timepoints | Central memory CD4 T cell | Immature B cell | 0.717647 | 0.002444 |
| All_timepoints | Central memory CD4 T cell | Immature dendritic cell | 0.717647 | 0.002444 |
| All_timepoints | Central memory CD4 T cell | Macrophage | 0.697059 | 0.0036 |
| All_timepoints | Central memory CD4 T cell | Mast cell | 0.044118 | 0.873739 |
| All_timepoints | Central memory CD4 T cell | MDSC | 0.861765 | 0 |
| All_timepoints | Central memory CD4 T cell | Memory B cell | 0.720588 | 0.002306 |
| All_timepoints | Central memory CD4 T cell | Monocyte | 0.688235 | 0.00421 |
| All_timepoints | Central memory CD4 T cell | Natural killer cell | 0.814706 | 1.719E-04 |
| All_timepoints | Central memory CD4 T cell | Natural killer T cell | 0.529412 | 0.037277 |
| All_timepoints | Central memory CD4 T cell | Neutrophil | -0.317647 | 0.230177 |
| All_timepoints | Central memory CD4 T cell | Plasmacytoid dendritic cell | 0.832353 | 6.562E-05 |
| All_timepoints | Central memory CD4 T cell | Regulatory T cell | 0.882353 | 0 |
| All_timepoints | Central memory CD4 T cell | T follicular helper cell | 0.752941 | 0.001147 |
| All_timepoints | Central memory CD4 T cell | Type 1 T helper cell | 0.691176 | 0.003999 |
| All_timepoints | Central memory CD4 T cell | Type 17 T helper cell | -0.561765 | 0.025771 |
| All_timepoints | Central memory CD4 T cell | Type 2 T helper cell | 0.111765 | 0.680533 |
| All_timepoints | Central memory CD8 T cell | Effector memeory CD4 T cell | 0.55 | 0.029578 |
| All_timepoints | Central memory CD8 T cell | Effector memeory CD8 T cell | 0.370588 | 0.15824 |
| All_timepoints | Central memory CD8 T cell | Eosinophil | -0.208824 | 0.436322 |
| All_timepoints | Central memory CD8 T cell | Gamma delta T cell | 0.758824 | 9.961E-04 |
| All_timepoints | Central memory CD8 T cell | Immature B cell | 0.785294 | 4.891E-04 |
| All_timepoints | Central memory CD8 T cell | Immature dendritic cell | 0.714706 | 0.002588 |
| All_timepoints | Central memory CD8 T cell | Macrophage | 0.682353 | 0.00466 |
| All_timepoints | Central memory CD8 T cell | Mast cell | 0.214706 | 0.423208 |
| All_timepoints | Central memory CD8 T cell | MDSC | 0.802941 | 2.743E-04 |
| All_timepoints | Central memory CD8 T cell | Memory B cell | 0.514706 | 0.043661 |
| All_timepoints | Central memory CD8 T cell | Monocyte | 0.782353 | 5.334E-04 |
| All_timepoints | Central memory CD8 T cell | Natural killer cell | 0.744118 | 0.001404 |
| All_timepoints | Central memory CD8 T cell | Natural killer T cell | 0.579412 | 0.020791 |
| All_timepoints | Central memory CD8 T cell | Neutrophil | -0.323529 | 0.221281 |
| All_timepoints | Central memory CD8 T cell | Plasmacytoid dendritic cell | 0.826471 | 9.560E-05 |
| All_timepoints | Central memory CD8 T cell | Regulatory T cell | 0.847059 | 1.023E-05 |
| All_timepoints | Central memory CD8 T cell | T follicular helper cell | 0.770588 | 7.389E-04 |
| All_timepoints | Central memory CD8 T cell | Type 1 T helper cell | 0.623529 | 0.011589 |
| All_timepoints | Central memory CD8 T cell | Type 17 T helper cell | -0.391176 | 0.135043 |
| All_timepoints | Central memory CD8 T cell | Type 2 T helper cell | -0.185294 | 0.490874 |
| All_timepoints | Effector memeory CD4 T cell | Effector memeory CD8 T cell | 0.008824 | 0.978149 |
| All_timepoints | Effector memeory CD4 T cell | Eosinophil | -0.214706 | 0.423208 |
| All_timepoints | Effector memeory CD4 T cell | Gamma delta T cell | 0.45 | 0.082167 |
| All_timepoints | Effector memeory CD4 T cell | Immature B cell | 0.479412 | 0.062393 |
| All_timepoints | Effector memeory CD4 T cell | Immature dendritic cell | 0.820588 | 1.308E-04 |
| All_timepoints | Effector memeory CD4 T cell | Macrophage | 0.479412 | 0.062393 |
| All_timepoints | Effector memeory CD4 T cell | Mast cell | -0.094118 | 0.729616 |
| All_timepoints | Effector memeory CD4 T cell | MDSC | 0.470588 | 0.067902 |
| All_timepoints | Effector memeory CD4 T cell | Memory B cell | 0.355882 | 0.176413 |
| All_timepoints | Effector memeory CD4 T cell | Monocyte | 0.720588 | 0.002306 |
| All_timepoints | Effector memeory CD4 T cell | Natural killer cell | 0.552941 | 0.028588 |
| All_timepoints | Effector memeory CD4 T cell | Natural killer T cell | 0.191176 | 0.476928 |
| All_timepoints | Effector memeory CD4 T cell | Neutrophil | -0.488235 | 0.057228 |
| All_timepoints | Effector memeory CD4 T cell | Plasmacytoid dendritic cell | 0.805882 | 2.460E-04 |
| All_timepoints | Effector memeory CD4 T cell | Regulatory T cell | 0.679412 | 0.004899 |
| All_timepoints | Effector memeory CD4 T cell | T follicular helper cell | 0.685294 | 0.004431 |
| All_timepoints | Effector memeory CD4 T cell | Type 1 T helper cell | 0.367647 | 0.161766 |
| All_timepoints | Effector memeory CD4 T cell | Type 17 T helper cell | -0.647059 | 0.008217 |
| All_timepoints | Effector memeory CD4 T cell | Type 2 T helper cell | 0.082353 | 0.762929 |
| All_timepoints | Effector memeory CD8 T cell | Eosinophil | -0.197059 | 0.463185 |
| All_timepoints | Effector memeory CD8 T cell | Gamma delta T cell | 0.555882 | 0.027623 |
| All_timepoints | Effector memeory CD8 T cell | Immature B cell | 0.491176 | 0.05558 |
| All_timepoints | Effector memeory CD8 T cell | Immature dendritic cell | 0.364706 | 0.165346 |
| All_timepoints | Effector memeory CD8 T cell | Macrophage | 0.538235 | 0.033808 |
| All_timepoints | Effector memeory CD8 T cell | Mast cell | 0.391176 | 0.135043 |
| All_timepoints | Effector memeory CD8 T cell | MDSC | 0.55 | 0.029578 |
| All_timepoints | Effector memeory CD8 T cell | Memory B cell | 0.473529 | 0.066027 |
| All_timepoints | Effector memeory CD8 T cell | Monocyte | 0.417647 | 0.108896 |
| All_timepoints | Effector memeory CD8 T cell | Natural killer cell | 0.585294 | 0.01931 |
| All_timepoints | Effector memeory CD8 T cell | Natural killer T cell | 0.629412 | 0.010658 |
| All_timepoints | Effector memeory CD8 T cell | Neutrophil | -0.011765 | 0.969412 |
| All_timepoints | Effector memeory CD8 T cell | Plasmacytoid dendritic cell | 0.402941 | 0.122925 |
| All_timepoints | Effector memeory CD8 T cell | Regulatory T cell | 0.476471 | 0.064191 |
| All_timepoints | Effector memeory CD8 T cell | T follicular helper cell | 0.364706 | 0.165346 |
| All_timepoints | Effector memeory CD8 T cell | Type 1 T helper cell | 0.623529 | 0.011589 |
| All_timepoints | Effector memeory CD8 T cell | Type 17 T helper cell | 0.264706 | 0.320666 |
| All_timepoints | Effector memeory CD8 T cell | Type 2 T helper cell | -0.444118 | 0.086622 |
| All_timepoints | Eosinophil | Gamma delta T cell | -0.382353 | 0.14467 |
| All_timepoints | Eosinophil | Immature B cell | -0.052941 | 0.847885 |
| All_timepoints | Eosinophil | Immature dendritic cell | -0.176471 | 0.512164 |
| All_timepoints | Eosinophil | Macrophage | -0.297059 | 0.263132 |
| All_timepoints | Eosinophil | Mast cell | 0.226471 | 0.397628 |
| All_timepoints | Eosinophil | MDSC | -0.282353 | 0.288413 |
| All_timepoints | Eosinophil | Memory B cell | -0.255882 | 0.337574 |
| All_timepoints | Eosinophil | Monocyte | -0.120588 | 0.656438 |
| All_timepoints | Eosinophil | Natural killer cell | -0.314706 | 0.234712 |
| All_timepoints | Eosinophil | Natural killer T cell | 0.067647 | 0.805142 |
| All_timepoints | Eosinophil | Neutrophil | 0.232353 | 0.385168 |
| All_timepoints | Eosinophil | Plasmacytoid dendritic cell | -0.126471 | 0.640554 |
| All_timepoints | Eosinophil | Regulatory T cell | -0.2 | 0.456391 |
| All_timepoints | Eosinophil | T follicular helper cell | -0.270588 | 0.309683 |
| All_timepoints | Eosinophil | Type 1 T helper cell | -0.244118 | 0.360918 |
| All_timepoints | Eosinophil | Type 17 T helper cell | 0.220588 | 0.410309 |
| All_timepoints | Eosinophil | Type 2 T helper cell | -0.417647 | 0.108896 |
| All_timepoints | Gamma delta T cell | Immature B cell | 0.826471 | 9.560E-05 |
| All_timepoints | Gamma delta T cell | Immature dendritic cell | 0.570588 | 0.023176 |
| All_timepoints | Gamma delta T cell | Macrophage | 0.767647 | 7.980E-04 |
| All_timepoints | Gamma delta T cell | Mast cell | 0.444118 | 0.086622 |
| All_timepoints | Gamma delta T cell | MDSC | 0.879412 | 0 |
| All_timepoints | Gamma delta T cell | Memory B cell | 0.805882 | 2.460E-04 |
| All_timepoints | Gamma delta T cell | Monocyte | 0.661765 | 0.006541 |
| All_timepoints | Gamma delta T cell | Natural killer cell | 0.894118 | 0 |
| All_timepoints | Gamma delta T cell | Natural killer T cell | 0.764706 | 8.605E-04 |
| All_timepoints | Gamma delta T cell | Neutrophil | -0.158824 | 0.556032 |
| All_timepoints | Gamma delta T cell | Plasmacytoid dendritic cell | 0.723529 | 0.002174 |
| All_timepoints | Gamma delta T cell | Regulatory T cell | 0.867647 | 0 |
| All_timepoints | Gamma delta T cell | T follicular helper cell | 0.785294 | 4.891E-04 |
| All_timepoints | Gamma delta T cell | Type 1 T helper cell | 0.841176 | 2.934E-05 |
| All_timepoints | Gamma delta T cell | Type 17 T helper cell | -0.229412 | 0.39137 |
| All_timepoints | Gamma delta T cell | Type 2 T helper cell | -0.147059 | 0.586183 |
| All_timepoints | Immature B cell | Immature dendritic cell | 0.629412 | 0.010658 |
| All_timepoints | Immature B cell | Macrophage | 0.726471 | 0.002048 |
| All_timepoints | Immature B cell | Mast cell | 0.567647 | 0.024018 |
| All_timepoints | Immature B cell | MDSC | 0.761765 | 9.265E-04 |
| All_timepoints | Immature B cell | Memory B cell | 0.576471 | 0.021563 |
| All_timepoints | Immature B cell | Monocyte | 0.767647 | 7.980E-04 |
| All_timepoints | Immature B cell | Natural killer cell | 0.841176 | 2.934E-05 |
| All_timepoints | Immature B cell | Natural killer T cell | 0.820588 | 1.308E-04 |
| All_timepoints | Immature B cell | Neutrophil | 0.002941 | 0.995629 |
| All_timepoints | Immature B cell | Plasmacytoid dendritic cell | 0.726471 | 0.002048 |
| All_timepoints | Immature B cell | Regulatory T cell | 0.888235 | 0 |
| All_timepoints | Immature B cell | T follicular helper cell | 0.867647 | 0 |
| All_timepoints | Immature B cell | Type 1 T helper cell | 0.835294 | 5.244E-05 |
| All_timepoints | Immature B cell | Type 17 T helper cell | -0.164706 | 0.541224 |
| All_timepoints | Immature B cell | Type 2 T helper cell | -0.388235 | 0.1382 |
| All_timepoints | Immature dendritic cell | Macrophage | 0.626471 | 0.011116 |
| All_timepoints | Immature dendritic cell | Mast cell | 0.220588 | 0.410309 |
| All_timepoints | Immature dendritic cell | MDSC | 0.573529 | 0.022358 |
| All_timepoints | Immature dendritic cell | Memory B cell | 0.367647 | 0.161766 |
| All_timepoints | Immature dendritic cell | Monocyte | 0.914706 | 0 |
| All_timepoints | Immature dendritic cell | Natural killer cell | 0.667647 | 0.005951 |
| All_timepoints | Immature dendritic cell | Natural killer T cell | 0.376471 | 0.151349 |
| All_timepoints | Immature dendritic cell | Neutrophil | -0.514706 | 0.043661 |
| All_timepoints | Immature dendritic cell | Plasmacytoid dendritic cell | 0.923529 | 0 |
| All_timepoints | Immature dendritic cell | Regulatory T cell | 0.717647 | 0.002444 |
| All_timepoints | Immature dendritic cell | T follicular helper cell | 0.776471 | 6.302E-04 |
| All_timepoints | Immature dendritic cell | Type 1 T helper cell | 0.538235 | 0.033808 |
| All_timepoints | Immature dendritic cell | Type 17 T helper cell | -0.455882 | 0.077883 |
| All_timepoints | Immature dendritic cell | Type 2 T helper cell | -0.158824 | 0.556032 |
| All_timepoints | Macrophage | Mast cell | 0.352941 | 0.180212 |
| All_timepoints | Macrophage | MDSC | 0.747059 | 0.001314 |
| All_timepoints | Macrophage | Memory B cell | 0.467647 | 0.069817 |
| All_timepoints | Macrophage | Monocyte | 0.791176 | 4.083E-04 |
| All_timepoints | Macrophage | Natural killer cell | 0.779412 | 5.804E-04 |
| All_timepoints | Macrophage | Natural killer T cell | 0.597059 | 0.016599 |
| All_timepoints | Macrophage | Neutrophil | -0.338235 | 0.200041 |
| All_timepoints | Macrophage | Plasmacytoid dendritic cell | 0.673529 | 0.005405 |
| All_timepoints | Macrophage | Regulatory T cell | 0.826471 | 9.560E-05 |
| All_timepoints | Macrophage | T follicular helper cell | 0.826471 | 9.560E-05 |
| All_timepoints | Macrophage | Type 1 T helper cell | 0.661765 | 0.006541 |
| All_timepoints | Macrophage | Type 17 T helper cell | -0.202941 | 0.449649 |
| All_timepoints | Macrophage | Type 2 T helper cell | -0.244118 | 0.360918 |
| All_timepoints | Mast cell | MDSC | 0.197059 | 0.463185 |
| All_timepoints | Mast cell | Memory B cell | 0.158824 | 0.556032 |
| All_timepoints | Mast cell | Monocyte | 0.358824 | 0.17267 |
| All_timepoints | Mast cell | Natural killer cell | 0.311765 | 0.239304 |
| All_timepoints | Mast cell | Natural killer T cell | 0.567647 | 0.024018 |
| All_timepoints | Mast cell | Neutrophil | 0.235294 | 0.379021 |
| All_timepoints | Mast cell | Plasmacytoid dendritic cell | 0.244118 | 0.360918 |
| All_timepoints | Mast cell | Regulatory T cell | 0.279412 | 0.293643 |
| All_timepoints | Mast cell | T follicular helper cell | 0.391176 | 0.135043 |
| All_timepoints | Mast cell | Type 1 T helper cell | 0.614706 | 0.013103 |
| All_timepoints | Mast cell | Type 17 T helper cell | 0.514706 | 0.043661 |
| All_timepoints | Mast cell | Type 2 T helper cell | -0.638235 | 0.009375 |
| All_timepoints | MDSC | Memory B cell | 0.808824 | 2.195E-04 |
| All_timepoints | MDSC | Monocyte | 0.632353 | 0.010216 |
| All_timepoints | MDSC | Natural killer cell | 0.917647 | 0 |
| All_timepoints | MDSC | Natural killer T cell | 0.691176 | 0.003999 |
| All_timepoints | MDSC | Neutrophil | -0.367647 | 0.161766 |
| All_timepoints | MDSC | Plasmacytoid dendritic cell | 0.773529 | 6.830E-04 |
| All_timepoints | MDSC | Regulatory T cell | 0.885294 | 0 |
| All_timepoints | MDSC | T follicular helper cell | 0.664706 | 0.006241 |
| All_timepoints | MDSC | Type 1 T helper cell | 0.738235 | 0.001598 |
| All_timepoints | MDSC | Type 17 T helper cell | -0.297059 | 0.263132 |
| All_timepoints | MDSC | Type 2 T helper cell | -0.032353 | 0.908401 |
| All_timepoints | Memory B cell | Monocyte | 0.364706 | 0.165346 |
| All_timepoints | Memory B cell | Natural killer cell | 0.785294 | 4.891E-04 |
| All_timepoints | Memory B cell | Natural killer T cell | 0.694118 | 0.003796 |
| All_timepoints | Memory B cell | Neutrophil | -0.1 | 0.71313 |
| All_timepoints | Memory B cell | Plasmacytoid dendritic cell | 0.585294 | 0.01931 |
| All_timepoints | Memory B cell | Regulatory T cell | 0.641176 | 0.008975 |
| All_timepoints | Memory B cell | T follicular helper cell | 0.420588 | 0.106235 |
| All_timepoints | Memory B cell | Type 1 T helper cell | 0.738235 | 0.001598 |
| All_timepoints | Memory B cell | Type 17 T helper cell | -0.211765 | 0.429738 |
| All_timepoints | Memory B cell | Type 2 T helper cell | 0.117647 | 0.664435 |
| All_timepoints | Monocyte | Natural killer cell | 0.744118 | 0.001404 |
| All_timepoints | Monocyte | Natural killer T cell | 0.561765 | 0.025771 |
| All_timepoints | Monocyte | Neutrophil | -0.447059 | 0.084373 |
| All_timepoints | Monocyte | Plasmacytoid dendritic cell | 0.867647 | 0 |
| All_timepoints | Monocyte | Regulatory T cell | 0.802941 | 2.743E-04 |
| All_timepoints | Monocyte | T follicular helper cell | 0.905882 | 0 |
| All_timepoints | Monocyte | Type 1 T helper cell | 0.605882 | 0.014769 |
| All_timepoints | Monocyte | Type 17 T helper cell | -0.352941 | 0.180212 |
| All_timepoints | Monocyte | Type 2 T helper cell | -0.332353 | 0.208367 |
| All_timepoints | Natural killer cell | Natural killer T cell | 0.767647 | 7.980E-04 |
| All_timepoints | Natural killer cell | Neutrophil | -0.364706 | 0.165346 |
| All_timepoints | Natural killer cell | Plasmacytoid dendritic cell | 0.802941 | 2.743E-04 |
| All_timepoints | Natural killer cell | Regulatory T cell | 0.905882 | 0 |
| All_timepoints | Natural killer cell | T follicular helper cell | 0.814706 | 1.719E-04 |
| All_timepoints | Natural killer cell | Type 1 T helper cell | 0.797059 | 3.369E-04 |
| All_timepoints | Natural killer cell | Type 17 T helper cell | -0.273529 | 0.304279 |
| All_timepoints | Natural killer cell | Type 2 T helper cell | -0.114706 | 0.672467 |
| All_timepoints | Natural killer T cell | Neutrophil | 0.064706 | 0.813651 |
| All_timepoints | Natural killer T cell | Plasmacytoid dendritic cell | 0.511765 | 0.045033 |
| All_timepoints | Natural killer T cell | Regulatory T cell | 0.688235 | 0.00421 |
| All_timepoints | Natural killer T cell | T follicular helper cell | 0.626471 | 0.011116 |
| All_timepoints | Natural killer T cell | Type 1 T helper cell | 0.826471 | 9.560E-05 |
| All_timepoints | Natural killer T cell | Type 17 T helper cell | 0.052941 | 0.847885 |
| All_timepoints | Natural killer T cell | Type 2 T helper cell | -0.429412 | 0.098537 |
| All_timepoints | Neutrophil | Plasmacytoid dendritic cell | -0.517647 | 0.042322 |
| All_timepoints | Neutrophil | Regulatory T cell | -0.297059 | 0.263132 |
| All_timepoints | Neutrophil | T follicular helper cell | -0.276471 | 0.298932 |
| All_timepoints | Neutrophil | Type 1 T helper cell | 0.023529 | 0.934507 |
| All_timepoints | Neutrophil | Type 17 T helper cell | 0.329412 | 0.212614 |
| All_timepoints | Neutrophil | Type 2 T helper cell | -0.155882 | 0.563504 |
| All_timepoints | Plasmacytoid dendritic cell | Regulatory T cell | 0.858824 | 0 |
| All_timepoints | Plasmacytoid dendritic cell | T follicular helper cell | 0.773529 | 6.830E-04 |
| All_timepoints | Plasmacytoid dendritic cell | Type 1 T helper cell | 0.641176 | 0.008975 |
| All_timepoints | Plasmacytoid dendritic cell | Type 17 T helper cell | -0.411765 | 0.114361 |
| All_timepoints | Plasmacytoid dendritic cell | Type 2 T helper cell | -0.176471 | 0.512164 |
| All_timepoints | Regulatory T cell | T follicular helper cell | 0.885294 | 0 |
| All_timepoints | Regulatory T cell | Type 1 T helper cell | 0.714706 | 0.002588 |
| All_timepoints | Regulatory T cell | Type 17 T helper cell | -0.4 | 0.125879 |
| All_timepoints | Regulatory T cell | Type 2 T helper cell | -0.229412 | 0.39137 |
| All_timepoints | T follicular helper cell | Type 1 T helper cell | 0.661765 | 0.006541 |
| All_timepoints | T follicular helper cell | Type 17 T helper cell | -0.4 | 0.125879 |
| All_timepoints | T follicular helper cell | Type 2 T helper cell | -0.267647 | 0.315146 |
| All_timepoints | Type 1 T helper cell | Type 17 T helper cell | 0.091176 | 0.737903 |
| All_timepoints | Type 1 T helper cell | Type 2 T helper cell | -0.258824 | 0.33188 |
| All_timepoints | Type 17 T helper cell | Type 2 T helper cell | -0.511765 | 0.045033 |
