# Supplementary material for: Ferroptosis-associated myeloid cell heterogeneity and inflammatory amplification following spinal cord injury
Source: Front Immunol. 2026 Apr 22;17:1831161. doi: 10.3389/fimmu.2026.1831161 (PMC13143767; doi:10.3389/fimmu.2026.1831161)
Supplement: Supplementary file 1 [file DataSheet1.zip › Supplementary_Table_S16.docx]

# Supplementary Table S16. Annotation map of myeloid subpopulations identified by single-cell RNA-sequencing

| **cluster_id** | **subpopulation** |
| --- | --- |
| 0 | M1a |
| 1 | M0 |
| 2 | M1b |
| 3 | M2 |
| 4 | M3 |
| 5 | M4 |
| 6 | M5 |

# Supplementary Table S16. Top marker genes of dominant injury-associated myeloid subpopulations

| **subpopulation** | **cluster** | **gene** | **avg_log2FC** | **pct.1** | **pct.2** | **p_val** | **p_val_adj** |
| --- | --- | --- | --- | --- | --- | --- | --- |
| M1a | 0 | Apoc1 | 4.144317 | 0.817 | 0.287 | 0 | 0 |
| M1a | 0 | AABR07030791.1 | 3.449156 | 0.415 | 0.057 | 0 | 0 |
| M1a | 0 | Ly49si1 | 3.335121 | 0.689 | 0.071 | 0 | 0 |
| M1a | 0 | Siglec1 | 3.305143 | 0.692 | 0.078 | 0 | 0 |
| M1a | 0 | AABR07027569.3 | 3.188985 | 0.3 | 0.03 | 0 | 0 |
| M1a | 0 | Htr2b | 3.116205 | 0.535 | 0.067 | 0 | 0 |
| M1a | 0 | Pon3 | 3.040059 | 0.255 | 0.026 | 0 | 0 |
| M1a | 0 | RGD1305807 | 3.03341 | 0.251 | 0.024 | 0 | 0 |
| M1a | 0 | Apoe | 3.014653 | 0.996 | 0.804 | 0 | 0 |
| M1a | 0 | Fxyd2 | 2.96628 | 0.499 | 0.066 | 0 | 0 |
| M1b | 2 | Edn3 | 4.113691 | 0.278 | 0.018 | 0 | 0 |
| M1b | 2 | P2ry12 | 3.667873 | 0.456 | 0.048 | 0 | 0 |
| M1b | 2 | Ltc4s | 3.529148 | 0.623 | 0.07 | 0 | 0 |
| M1b | 2 | Grxcr1 | 3.493738 | 0.526 | 0.049 | 0 | 0 |
| M1b | 2 | Tmem119 | 3.487054 | 0.584 | 0.078 | 0 | 0 |
| M1b | 2 | Gpr34 | 3.469932 | 0.573 | 0.068 | 0 | 0 |
| M1b | 2 | F10 | 3.436795 | 0.296 | 0.029 | 0 | 0 |
| M1b | 2 | Slco2b1 | 3.335109 | 0.682 | 0.086 | 0 | 0 |
| M1b | 2 | Spint1 | 3.295031 | 0.334 | 0.03 | 0 | 0 |
| M1b | 2 | Clec2l | 3.266915 | 0.743 | 0.112 | 0 | 0 |
