# Supplementary material for: Ferroptosis-associated myeloid cell heterogeneity and inflammatory amplification following spinal cord injury
Source: Front Immunol. 2026 Apr 22;17:1831161. doi: 10.3389/fimmu.2026.1831161 (PMC13143767; doi:10.3389/fimmu.2026.1831161)
Supplement: Supplementary file 1 [file DataSheet1.zip › Supplementary_Table_S17.docx]

# Supplementary Table S17. GO enrichment results of marker genes from M1a

| **subpopulation** | **ID** | **Description** | **GeneRatio** | **BgRatio** | **RichFactor** | **FoldEnrichment** | **pvalue** | **p.adjust** | **qvalue** | **geneID** | **Count** |
| --- | --- | --- | --- | --- | --- | --- | --- | --- | --- | --- | --- |
| M1a | GO:0002181 | cytoplasmic translation | 23/296 | 126/18913 | 0.18254 | 11.663422 | 2.762E-18 | 1.014E-14 | 8.295E-15 | 29284/64298/291434/124440/691531/245981/290641/64307/29565/81775/29283/64640/317646/81766/293427/64205/362631/140661/124323/29236/303831/292762/292964 | 23 |
| M1a | GO:0042255 | ribosome assembly | 13/296 | 64/18913 | 0.203125 | 12.978727 | 1.672E-11 | 3.069E-08 | 2.510E-08 | 29285/29284/94266/691531/81763/29752/64307/292624/362631/25538/29236/81773/122799 | 13 |
| M1a | GO:0042254 | ribosome biogenesis | 25/296 | 317/18913 | 0.078864 | 5.039059 | 3.650E-11 | 3.861E-08 | 3.158E-08 | 29285/29284/65139/140655/81774/94266/691531/81763/29752/65136/64307/81775/29286/171456/64306/292624/65043/362631/25538/296709/124323/297755/29236/81773/122799 | 25 |
| M1a | GO:0042274 | ribosomal small subunit biogenesis | 16/296 | 117/18913 | 0.136752 | 8.737815 | 4.207E-11 | 3.861E-08 | 3.158E-08 | 29285/29284/65139/140655/81774/94266/691531/29752/65136/81775/29286/25538/124323/29236/81773/122799 | 16 |
| M1a | GO:0000028 | ribosomal small subunit assembly | 9/296 | 25/18913 | 0.36 | 23.002297 | 8.185E-11 | 6.009E-08 | 4.916E-08 | 29285/29284/94266/691531/29752/25538/29236/81773/122799 | 9 |
| M1a | GO:0022613 | ribonucleoprotein complex biogenesis | 29/296 | 450/18913 | 0.064444 | 4.117695 | 1.250E-10 | 7.651E-08 | 6.259E-08 | 29285/29284/65139/140655/81774/94266/691531/81763/29752/65136/64307/81775/29286/317646/171456/64306/293427/292624/65043/362631/25538/296709/124323/297755/29236/292762/81773/122799/680309 | 29 |
| M1a | GO:0022618 | protein-RNA complex assembly | 17/296 | 190/18913 | 0.089474 | 5.716945 | 8.298E-09 | 4.352E-06 | 3.560E-06 | 29285/29284/94266/691531/81763/29752/64307/317646/293427/292624/362631/25538/29236/292762/81773/122799/680309 | 17 |
| M1a | GO:0071826 | protein-RNA complex organization | 17/296 | 199/18913 | 0.085427 | 5.45839 | 1.662E-08 | 7.628E-06 | 6.240E-06 | 29285/29284/94266/691531/81763/29752/64307/317646/293427/292624/362631/25538/29236/292762/81773/122799/680309 | 17 |
| M1a | GO:0006364 | rRNA processing | 17/296 | 205/18913 | 0.082927 | 5.298632 | 2.586E-08 | 1.055E-05 | 8.629E-06 | 29285/29284/140655/94266/691531/81763/65136/81775/29286/171456/64306/292624/65043/362631/296709/297755/122799 | 17 |
| M1a | GO:0016072 | rRNA metabolic process | 18/296 | 243/18913 | 0.074074 | 4.732983 | 5.725E-08 | 2.102E-05 | 1.719E-05 | 29285/29284/140655/94266/691531/81763/65136/81775/29286/171456/64306/292624/65043/362631/296709/297755/690966/122799 | 18 |
| M1a | GO:0007033 | vacuole organization | 17/296 | 221/18913 | 0.076923 | 4.915021 | 7.797E-08 | 2.602E-05 | 2.129E-05 | 83534/313210/362245/29411/25055/292156/300757/171293/64862/307415/114638/367562/289185/298575/246772/362645/315746 | 17 |
| M1a | GO:0002495 | APC | 7/296 | 27/18913 | 0.259259 | 16.56544 | 1.455E-07 | 4.451E-05 | 3.641E-05 | 301227/25441/24223/361689/294269/25599/309621 | 7 |
| M1a | GO:1902105 | regulation of leukocyte differentiation | 21/296 | 350/18913 | 0.06 | 3.833716 | 1.663E-07 | 4.697E-05 | 3.842E-05 | 314322/24516/307403/171411/361537/301227/297077/29344/25587/361103/315348/294269/25599/54264/309621/690899/290995/360918/78969/362966/298765 | 21 |
| M1a | GO:0002504 | APC-MHC II | 7/296 | 29/18913 | 0.241379 | 15.422996 | 2.489E-07 | 6.527E-05 | 5.340E-05 | 301227/25441/24223/361689/294269/25599/309621 | 7 |
| M1a | GO:0006414 | translational elongation | 10/296 | 76/18913 | 0.131579 | 8.407272 | 2.906E-07 | 7.113E-05 | 5.819E-05 | 83427/499782/29565/64640/363241/140661/293725/292082/289508/79049 | 10 |
| M1a | GO:0042554 | superoxide anion generation | 8/296 | 45/18913 | 0.177778 | 11.359159 | 4.270E-07 | 9.797E-05 | 8.015E-05 | 24426/361537/502902/66021/24786/81664/114553/25732 | 8 |
| M1a | GO:1903706 | regulation of hemopoiesis | 23/296 | 438/18913 | 0.052511 | 3.355231 | 4.572E-07 | 9.874E-05 | 8.077E-05 | 314322/24516/307403/171411/361537/301227/297077/29344/24223/25587/362176/361103/315348/294269/25599/54264/309621/690899/290995/360918/78969/362966/298765 | 23 |
| M1a | GO:0002763 | positive regulation of myeloid leukocyte differentiation | 10/296 | 82/18913 | 0.121951 | 7.792106 | 5.997E-07 | 1.164E-04 | 9.525E-05 | 314322/24516/307403/361537/301227/29344/25587/25599/360918/78969 | 10 |
| M1a | GO:1902107 | positive regulation of leukocyte differentiation | 15/296 | 200/18913 | 0.075 | 4.792145 | 6.344E-07 | 1.164E-04 | 9.525E-05 | 314322/24516/307403/361537/301227/29344/25587/361103/315348/294269/25599/309621/690899/360918/78969 | 15 |
| M1a | GO:1903708 | positive regulation of hemopoiesis | 15/296 | 200/18913 | 0.075 | 4.792145 | 6.344E-07 | 1.164E-04 | 9.525E-05 | 314322/24516/307403/361537/301227/29344/25587/361103/315348/294269/25599/309621/690899/360918/78969 | 15 |

# Supplementary Table S17. GO enrichment results of marker genes from M1b

| **subpopulation** | **ID** | **Description** | **GeneRatio** | **BgRatio** | **RichFactor** | **FoldEnrichment** | **pvalue** | **p.adjust** | **qvalue** | **geneID** | **Count** |
| --- | --- | --- | --- | --- | --- | --- | --- | --- | --- | --- | --- |
| M1b | GO:0002443 | leukocyte mediated immunity | 58/852 | 445/18913 | 0.130337 | 2.893269 | 3.004E-13 | 8.743E-10 | 7.179E-10 | 24494/81503/414792/24974/117254/365395/361689/292594/24468/287362/25464/25425/25423/499342/366126/501872/29146/25476/294269/292483/414783/691993/24231/60466/25542/414788/282829/294228/24747/363578/25599/361537/25301/289211/294270/64171/24223/287437/474146/502902/680611/304005/367901/317371/100049583/25441/308977/79113/29681/25513/366957/81804/27100/59086/25621/361242/114095/24451 | 58 |
| M1b | GO:0002460 | Adaptive Immune Response based on Somatic Recombination | 50/852 | 348/18913 | 0.143678 | 3.189419 | 3.367E-13 | 8.743E-10 | 7.179E-10 | 24494/414792/24974/308496/83785/365395/361689/292594/24468/287362/25464/25425/25423/499342/309452/313587/29146/683206/294269/292483/414783/24231/60466/414788/294228/24747/363578/25599/289211/294270/64171/24223/287437/316519/502902/680611/304005/367901/317371/100049583/25441/308977/29681/29355/24499/59086/25621/361242/25496/310630 | 50 |
| M1b | GO:0002181 | cytoplasmic translation | 28/852 | 126/18913 | 0.222222 | 4.932968 | 1.593E-12 | 1.828E-09 | 1.501E-09 | 29304/287417/117042/83789/81768/689284/81767/58927/140661/64205/300069/28298/29283/124323/124440/171350/29236/64307/81729/300955/293427/500538/117019/299027/360659/81775/100365062/498225 | 28 |
| M1b | GO:0002250 | adaptive immune response | 56/852 | 438/18913 | 0.127854 | 2.838146 | 1.729E-12 | 1.828E-09 | 1.501E-09 | 24494/414792/24974/308496/83785/365395/361689/292594/24468/287362/25464/25425/25423/499342/309452/313587/29146/683206/294269/292483/414783/309621/24231/60466/414788/294228/24747/363578/25599/289211/294270/64171/24223/287437/316519/502902/680611/304005/367901/317371/100049583/25441/308977/29681/29355/170637/24499/59086/291171/24931/24514/679975/25621/361242/25496/310630 | 56 |
| M1b | GO:0002697 | regulation of immune effector process | 57/852 | 451/18913 | 0.126386 | 2.805557 | 1.760E-12 | 1.828E-09 | 1.501E-09 | 24494/29287/81503/414792/24974/308496/365395/89783/292594/24468/114091/287362/25732/499342/29185/366126/313587/501872/56822/25476/294269/414783/691993/60466/414788/294228/24747/363578/25599/361537/289211/24822/296953/294270/64171/24223/287437/502902/297961/304005/367901/317371/100049583/25441/79113/29681/29355/366957/81804/27100/59086/25621/361242/114766/24451/29143/294009 | 57 |
| M1b | GO:0045088 | regulation of innate immune response | 57/852 | 461/18913 | 0.123644 | 2.744699 | 4.349E-12 | 3.764E-09 | 3.091E-09 | 29287/25493/60350/24974/291091/25338/304545/450223/361689/24468/114091/294422/287362/499342/24508/366126/291921/56822/25728/683206/25476/361532/362418/691993/24747/363578/361537/64171/292892/502902/501664/305340/304005/361794/367901/117063/289357/364674/100049583/302557/79113/29681/304291/316136/58852/171121/25513/27100/59086/83619/252832/360630/310877/114766/29411/29143/501854 | 57 |
| M1b | GO:0002757 | immune response-activating signaling pathway | 58/852 | 480/18913 | 0.120833 | 2.682301 | 7.267E-12 | 5.391E-09 | 4.427E-09 | 25493/24626/60350/308496/25338/304545/450223/114203/89783/361689/292594/24468/114091/294422/287362/499342/24508/313587/56822/683206/25476/361532/362418/691993/81736/363578/361537/289211/294270/292892/502902/29364/304005/361794/367901/117019/117063/289357/364674/100049583/25441/29681/316136/58852/171121/25513/60669/24931/25621/29192/252832/361242/360630/310877/114766/29411/298203/501854 | 58 |
| M1b | GO:0002833 | positive regulation of response to biotic stimulus | 51/852 | 408/18913 | 0.125 | 2.774795 | 4.034E-11 | 2.619E-08 | 2.150E-08 | 29287/81503/25493/60350/24974/25338/304545/450223/361689/24468/114091/294422/287362/499342/24508/366126/56822/683206/25476/361532/362418/691993/24747/363578/361537/294270/64171/292892/502902/305340/304005/361794/367901/360571/289357/364674/100049583/302557/29681/304291/316136/58852/171121/25513/252832/360630/310877/114766/29411/29143/501854 | 51 |
| M1b | GO:0002449 | lymphocyte mediated immunity | 45/852 | 335/18913 | 0.134328 | 2.981869 | 5.312E-11 | 3.065E-08 | 2.517E-08 | 24494/414792/24974/117254/365395/361689/292594/24468/287362/25464/25425/25423/499342/29146/25476/294269/292483/414783/24231/60466/414788/294228/24747/363578/25599/25301/289211/294270/64171/24223/287437/474146/680611/304005/367901/317371/100049583/25441/308977/29681/25513/27100/59086/25621/361242 | 45 |
| M1b | GO:0045089 | positive regulation of innate immune response | 47/852 | 375/18913 | 0.125333 | 2.782194 | 2.177E-10 | 1.071E-07 | 8.795E-08 | 29287/25493/60350/24974/25338/304545/450223/361689/24468/114091/294422/287362/499342/24508/366126/56822/683206/25476/361532/362418/691993/24747/363578/361537/64171/292892/502902/305340/304005/361794/367901/289357/364674/100049583/302557/29681/304291/316136/58852/171121/25513/252832/360630/310877/114766/29411/501854 | 47 |
| M1b | GO:0019882 | antigen processing and presentation | 25/852 | 124/18913 | 0.201613 | 4.475475 | 2.314E-10 | 1.071E-07 | 8.795E-08 | 414792/24974/361689/25464/294269/414783/309621/414788/294228/24747/25599/289211/25217/294270/24223/367328/316519/25532/290644/317371/360571/100049583/25441/362431/29614 | 25 |
| M1b | GO:0034341 | response to type II interferon | 28/852 | 154/18913 | 0.181818 | 4.036065 | 2.475E-10 | 1.071E-07 | 8.795E-08 | 309175/116465/29244/689377/25464/24508/56822/303653/360697/25476/292483/497942/309621/25542/293618/25599/294270/316519/29364/287876/317371/117063/81804/24514/114095/29528/114709/501854 | 28 |
| M1b | GO:0002221 | pattern recognition receptor signaling pathway | 38/852 | 267/18913 | 0.142322 | 3.159317 | 3.179E-10 | 1.182E-07 | 9.706E-08 | 25493/60350/25338/304545/450223/361689/24468/114091/294422/287362/499342/24508/56822/683206/25476/361532/362418/691993/363578/292892/502902/304005/361794/367901/289357/364674/100049583/29681/316136/58852/171121/25513/252832/360630/310877/114766/29411/501854 | 38 |
| M1b | GO:0002758 | innate immune response-activating signaling pathway | 39/852 | 279/18913 | 0.139785 | 3.102996 | 3.187E-10 | 1.182E-07 | 9.706E-08 | 25493/60350/25338/304545/450223/361689/24468/114091/294422/287362/499342/24508/56822/683206/25476/361532/362418/691993/363578/361537/292892/502902/304005/361794/367901/289357/364674/100049583/29681/316136/58852/171121/25513/252832/360630/310877/114766/29411/501854 | 39 |
| M1b | GO:0050866 | negative regulation of cell activation | 35/852 | 236/18913 | 0.148305 | 3.292129 | 5.169E-10 | 1.790E-07 | 1.470E-07 | 24253/24771/24974/308496/89783/292594/156726/499342/29185/24508/313587/501872/56822/25728/683206/25476/309621/24747/363578/25599/361537/289211/294270/367901/296257/117063/79113/310663/29355/58852/170637/59086/361242/24936/29143 | 35 |
| M1b | GO:0002819 | regulation of adaptive immune response | 35/852 | 239/18913 | 0.146444 | 3.250805 | 7.321E-10 | 2.023E-07 | 1.661E-07 | 24494/414792/24974/308496/365395/292594/24468/287362/499342/313587/683206/294269/414783/60466/414788/294228/24747/363578/289211/294270/64171/24223/287437/316519/502902/304005/367901/317371/25441/29355/170637/59086/291171/25621/361242 | 35 |
| M1b | GO:0002695 | negative regulation of leukocyte activation | 33/852 | 216/18913 | 0.152778 | 3.391416 | 7.400E-10 | 2.023E-07 | 1.661E-07 | 24253/24771/24974/308496/89783/292594/156726/499342/29185/24508/313587/501872/56822/683206/25476/309621/24747/363578/25599/361537/289211/294270/367901/296257/117063/79113/310663/29355/58852/170637/59086/361242/29143 | 33 |
| M1b | GO:0071706 | tumor necrosis factor superfamily cytokine production | 33/852 | 216/18913 | 0.152778 | 3.391416 | 7.400E-10 | 2.023E-07 | 1.661E-07 | 24493/60350/24974/116465/24426/292594/25732/499342/313587/501872/683206/25476/25542/363578/361537/289211/64171/502902/29364/297961/680611/361794/367901/361927/100049583/25441/362431/25513/290549/59086/291171/24514/29192 | 33 |
| M1b | GO:1903555 | regulation of tumor necrosis factor superfamily cytokine production | 33/852 | 216/18913 | 0.152778 | 3.391416 | 7.400E-10 | 2.023E-07 | 1.661E-07 | 24493/60350/24974/116465/24426/292594/25732/499342/313587/501872/683206/25476/25542/363578/361537/289211/64171/502902/29364/297961/680611/361794/367901/361927/100049583/25441/362431/25513/290549/59086/291171/24514/29192 | 33 |
| M1b | GO:0002218 | activation of innate immune response | 40/852 | 305/18913 | 0.131148 | 2.91126 | 1.293E-09 | 3.356E-07 | 2.756E-07 | 25493/60350/25338/304545/450223/361689/24468/114091/294422/287362/499342/24508/56822/683206/25476/361532/362418/691993/363578/361537/292892/502902/304005/361794/367901/289357/364674/100049583/302557/29681/316136/58852/171121/25513/252832/360630/310877/114766/29411/501854 | 40 |

# Supplementary Table S17. KEGG enrichment results of marker genes from M1a

| **subpopulation** | **ID** | **Description** | **GeneRatio** | **BgRatio** | **RichFactor** | **FoldEnrichment** | **pvalue** | **p.adjust** | **qvalue** | **geneID** | **Count** |
| --- | --- | --- | --- | --- | --- | --- | --- | --- | --- | --- | --- |
| M1a | rno04380 | Osteoclast differentiation | 22/362 | 132/11141 | 0.166667 | 5.129374 | 2.303E-10 | 6.841E-08 | 5.941E-08 | 314322/24516/307403/100360880/361537/301227/116465/24518/29591/155918/114553/113894/25732/499356/289211/81810/81649/24674/288264/25155/25625/25150 | 22 |
| M1a | rno04142 | Lysosome | 30/362 | 254/11141 | 0.11811 | 3.634989 | 8.125E-10 | 1.207E-07 | 1.048E-07 | 286898/83534/282838/25425/29411/25055/300757/171293/288785/287435/361378/25524/361704/79248/316033/353307/367562/306332/315746/25732/688966/683402/315222/315047/85491/316214/246280/50654/361401/309969 | 30 |
| M1a | rno05152 | Tuberculosis | 17/362 | 189/11141 | 0.089947 | 2.768234 | 1.418E-04 | 0.014035 | 0.012187 | 25441/502902/116465/171293/294269/25599/309621/289211/29197/294270/81649/24674/64171/25155/25337/25625/50654 | 17 |
| M1a | rno04979 | Cholesterol metabolism | 8/362 | 53/11141 | 0.150943 | 4.645471 | 2.836E-04 | 0.019492 | 0.016925 | 296371/25728/286898/313210/25292/25055/25073/306825 | 8 |
| M1a | rno00600 | Sphingolipid metabolism | 8/362 | 56/11141 | 0.142857 | 4.396606 | 4.176E-04 | 0.019492 | 0.016925 | 282838/300757/25524/316033/305684/315222/300129/499210 | 8 |
| M1a | rno04137 | Mitophagy - animal | 11/362 | 102/11141 | 0.107843 | 3.319007 | 4.622E-04 | 0.019492 | 0.016925 | 64156/24516/362245/64862/288584/113894/307641/303554/316214/363442/81650 | 11 |
| M1a | rno04659 | Th17 cell differentiation | 11/362 | 104/11141 | 0.105769 | 3.25518 | 5.462E-04 | 0.019492 | 0.016925 | 314322/24516/116465/294269/309621/29591/81810/294270/81649/24499/24674 | 11 |
| M1a | rno05135 | Yersinia infection | 13/362 | 139/11141 | 0.093525 | 2.878354 | 5.861E-04 | 0.019492 | 0.016925 | 314322/24516/308875/24931/155918/81771/317371/292892/29197/81649/499537/25166/360820 | 13 |
| M1a | rno05140 | Leishmaniasis | 9/362 | 74/11141 | 0.121622 | 3.743057 | 6.260E-04 | 0.019492 | 0.016925 | 314322/24516/116465/66021/294269/309621/114553/294270/81649 | 9 |
| M1a | rno04612 | Antigen processing and presentation | 10/362 | 90/11141 | 0.111111 | 3.419583 | 6.563E-04 | 0.019492 | 0.016925 | 24973/24223/294269/25599/309621/24931/294270/25337/414784/50654 | 10 |
| M1a | rno04658 | Th1 and Th2 cell differentiation | 9/362 | 88/11141 | 0.102273 | 3.14757 | 0.002175 | 0.05338 | 0.046352 | 314322/24516/116465/294269/309621/294270/81649/24674/367264 | 9 |
| M1a | rno05142 | Chagas disease | 10/362 | 106/11141 | 0.09434 | 2.903419 | 0.002313 | 0.05338 | 0.046352 | 314322/24516/89788/116465/29591/81664/81810/81649/25625/117281 | 10 |
| M1a | rno00511 | Other glycan degradation | 4/362 | 18/11141 | 0.222222 | 6.839165 | 0.002337 | 0.05338 | 0.046352 | 360955/300757/361378/316033 | 4 |
| M1a | rno05133 | Pertussis | 8/362 | 74/11141 | 0.108108 | 3.327161 | 0.002671 | 0.056666 | 0.049205 | 314322/24516/81664/292892/24231/81649/25166/292060 | 8 |
| M1a | rno05321 | Inflammatory bowel disease | 7/362 | 61/11141 | 0.114754 | 3.5317 | 0.003481 | 0.068915 | 0.059841 | 24516/116465/294269/309621/29197/294270/367264 | 7 |
| M1a | rno05166 | Human T-cell leukemia virus 1 infection | 17/362 | 256/11141 | 0.066406 | 2.043735 | 0.004188 | 0.077745 | 0.067509 | 314322/24516/24973/24330/24223/294269/309621/29591/81810/294270/296558/24674/307842/685029/414784/25625/85333 | 17 |
| M1a | rno04010 | MAPK signaling pathway | 19/362 | 302/11141 | 0.062914 | 1.936254 | 0.004579 | 0.07999 | 0.069458 | 314322/114856/24516/116663/307403/24518/291005/29591/498003/81771/25112/81810/81649/24674/24482/25625/315994/304530/292763 | 19 |
| M1a | rno04662 | B cell receptor signaling pathway | 8/362 | 82/11141 | 0.097561 | 3.00256 | 0.005051 | 0.080383 | 0.069799 | 314322/24516/293618/25621/499356/289211/24674/25155 | 8 |
| M1a | rno04064 | NF-kappa B signaling pathway | 9/362 | 100/11141 | 0.09 | 2.769862 | 0.005159 | 0.080383 | 0.069799 | 291005/25112/307641/499356/494338/25155/25591/25625/81650 | 9 |
| M1a | rno00310 | Lysine degradation | 7/362 | 66/11141 | 0.106061 | 3.264147 | 0.005413 | 0.080383 | 0.069799 | 306764/311968/64040/364975/290637/29539/310638 | 7 |

# Supplementary Table S17. KEGG enrichment results of marker genes from M1b

| **subpopulation** | **ID** | **Description** | **GeneRatio** | **BgRatio** | **RichFactor** | **FoldEnrichment** | **pvalue** | **p.adjust** | **qvalue** | **geneID** | **Count** |
| --- | --- | --- | --- | --- | --- | --- | --- | --- | --- | --- | --- |
| M1b | rno04142 | Lysosome | 35/518 | 254/11141 | 0.137795 | 2.963662 | 6.764E-09 | 2.158E-06 | 1.645E-06 | 286898/89783/25425/25423/25732/300757/54398/171575/282838/290923/24162/361704/60575/316519/83615/309243/84431/306619/308909/293485/363087/83800/315746/24375/360476/498606/306991/294012/292485/360630/266732/361401/29411/291969/25524 | 35 |
| M1b | rno04064 | NF-kappa B signaling pathway | 20/518 | 100/11141 | 0.2 | 4.301544 | 2.575E-08 | 4.107E-06 | 3.131E-06 | 24494/81503/25493/170929/114105/299626/78971/60350/29527/25464/171551/117279/309452/683206/81736/60371/367901/307641/297604/81650 | 20 |
| M1b | rno05323 | Rheumatoid arthritis | 17/518 | 87/11141 | 0.195402 | 4.202658 | 4.093E-07 | 4.352E-05 | 3.318E-05 | 24494/81503/114105/24493/83785/25464/171551/25732/56822/294269/309621/25542/294270/287437/83615/59086/291969 | 17 |
| M1b | rno04612 | Antigen processing and presentation | 17/518 | 90/11141 | 0.188889 | 4.06257 | 6.791E-07 | 5.416E-05 | 4.128E-05 | 414792/24974/24468/294269/414783/309621/414788/294228/24747/25599/25217/294270/24223/290644/306353/24931/29614 | 17 |
| M1b | rno05140 | Leishmaniasis | 15/518 | 74/11141 | 0.202703 | 4.359673 | 1.205E-06 | 7.691E-05 | 5.862E-05 | 24494/25493/24493/81525/116465/29527/360697/294269/309621/81736/500904/294270/171361/59086/24514 | 15 |
| M1b | rno05152 | Tuberculosis | 24/518 | 189/11141 | 0.126984 | 2.731139 | 7.081E-06 | 3.765E-04 | 2.870E-04 | 24494/24253/24493/60350/116465/450223/360697/294269/362418/309621/81736/25599/25301/289211/294270/64171/502902/83615/304091/25441/306353/59086/24514/291969 | 24 |
| M1b | rno04668 | TNF signaling pathway | 18/518 | 119/11141 | 0.151261 | 3.253269 | 8.952E-06 | 4.079E-04 | 3.110E-04 | 24494/81503/25493/24253/114105/24517/78971/29527/25464/171551/117279/24508/29146/683206/81736/60371/680611/25513 | 18 |
| M1b | rno04380 | Osteoclast differentiation | 19/518 | 132/11141 | 0.143939 | 3.095808 | 1.060E-05 | 4.226E-04 | 3.222E-04 | 24494/25493/24493/24517/116465/292594/25732/309452/366126/65146/360697/81736/361537/289211/500904/367901/686326/25513/59086 | 19 |
| M1b | rno04145 | Phagosome | 24/518 | 196/11141 | 0.122449 | 2.633599 | 1.323E-05 | 4.690E-04 | 3.575E-04 | 414792/300218/60350/24974/140914/500929/294269/414783/309621/60466/414788/294228/24747/289211/500904/294270/502902/83615/287876/360953/29528/291969/501854/445442 | 24 |
| M1b | rno05168 | Herpes simplex virus 1 infection | 26/518 | 226/11141 | 0.115044 | 2.47434 | 1.791E-05 | 5.640E-04 | 4.299E-04 | 24494/25493/414792/78971/24974/116465/360697/294269/414783/309621/81736/414788/294228/24747/25599/25217/60371/294270/64171/24223/292892/686326/117019/25513/24514/252832 | 26 |
| M1b | rno05332 | Graft-versus-host disease | 12/518 | 61/11141 | 0.196721 | 4.231027 | 1.945E-05 | 5.640E-04 | 4.299E-04 | 24494/414792/24493/24974/56822/294269/414783/309621/414788/294228/24747/294270 | 12 |
| M1b | rno05169 | Epstein-Barr virus infection | 26/518 | 237/11141 | 0.109705 | 2.359497 | 4.102E-05 | 0.00109 | 8.312E-04 | 25493/414792/299626/81525/24974/156726/25464/114851/309452/83571/683206/294269/414783/309621/81736/414788/294228/24747/25217/294270/24223/292892/64547/367901/686326/25513 | 26 |
| M1b | rno04621 | NOD-like receptor signaling pathway | 22/518 | 186/11141 | 0.11828 | 2.543924 | 5.151E-05 | 0.001264 | 9.635E-04 | 24494/81503/25493/114105/78971/81525/287362/171551/683206/64670/83531/81736/58974/252961/362245/60371/64171/292892/406163/315084/686326/29355 | 22 |
| M1b | rno04940 | Type I diabetes mellitus | 12/518 | 70/11141 | 0.171429 | 3.687038 | 8.112E-05 | 0.001848 | 0.001409 | 24494/414792/24493/24974/56822/294269/414783/309621/414788/294228/24747/294270 | 12 |
| M1b | rno04217 | Necroptosis | 21/518 | 181/11141 | 0.116022 | 2.495371 | 1.004E-04 | 0.002136 | 0.001628 | 24494/29292/24493/100360977/78971/25518/116465/287362/117279/683206/25319/360697/83531/60371/679886/24957/25176/365024/686326/308909/24514 | 21 |
| M1b | rno05134 | Legionellosis | 11/518 | 64/11141 | 0.171875 | 3.69664 | 1.561E-04 | 0.003112 | 0.002372 | 24494/81503/25493/114105/60350/24468/171551/309452/81736/171361/287276 | 11 |
| M1b | rno04060 | Cytokine-cytokine receptor interaction | 27/518 | 274/11141 | 0.09854 | 2.119374 | 1.842E-04 | 0.003457 | 0.002635 | 24494/81503/114105/24493/680609/116465/365395/60582/60628/29455/171551/353218/652957/360697/497942/25542/287437/246144/304091/171499/686326/308977/297604/24499/59086/289747/360548 | 27 |
| M1b | rno04137 | Mitophagy - animal | 14/518 | 102/11141 | 0.137255 | 2.95204 | 2.538E-04 | 0.004497 | 0.003428 | 64670/58974/362245/361568/290652/292886/307641/363442/140923/81650/288150/288584/114766/501854 | 14 |
| M1b | rno05132 | Salmonella infection | 26/518 | 268/11141 | 0.097015 | 2.08657 | 3.087E-04 | 0.004539 | 0.00346 | 24494/25493/300218/78971/60350/287362/500929/54227/290798/81736/60371/50685/315084/81824/287280/287876/361568/363448/308875/288064/299121/64303/29355/306991/84589/501854 | 26 |
| M1b | rno04210 | Apoptosis | 17/518 | 142/11141 | 0.119718 | 2.574868 | 3.116E-04 | 0.004539 | 0.00346 | 25493/170929/300218/299626/78971/25425/25423/117279/500929/81736/361704/60371/246144/287876/288753/64547/25513 | 17 |
