# Supplementary material for: Ferroptosis-associated myeloid cell heterogeneity and inflammatory amplification following spinal cord injury
Source: Front Immunol. 2026 Apr 22;17:1831161. doi: 10.3389/fimmu.2026.1831161 (PMC13143767; doi:10.3389/fimmu.2026.1831161)
Supplement: Supplementary file 1 [file DataSheet1.zip › Supplementary_Table_S18.docx]

# Supplementary Table S18. GSEA results for M1a vs. M0

| **comparison** | **ID** | **Description** | **setSize** | **enrichmentScore** | **NES** | **pvalue** | **p.adjust** | **qvalue** | **rank** | **leading_edge** | **core_enrichment** |
| --- | --- | --- | --- | --- | --- | --- | --- | --- | --- | --- | --- |
| M1a_vs_M0 | rno05012 | Parkinson disease | 176 | -0.452304 | -2.523913 | 1.000E-10 | 3.210E-08 | 2.368E-08 | 2218 | tags=60%, list=30%, signal=43% | 29425/362344/171374/192241/499529/54322/25643/24887/24888/79462/688386/24246/100361008/65138/29668/309159/363836/289754/303353/301011/81728/685322/65262/299954/117262/85492/29677/117550/362897/301427/362440/64476/289990/64539/116550/497902/29666/361385/361986/25282/252934/25581/307821/29675/29676/295923/301123/294964/94271/81827/282819/245965/29545/365388/289217/362588/299739/117263/29671/83806/301458/363061/161475/171041/300047/192647/306009/292766/29670/287772/287984/29669/25715/29754/293991/293130/54318/29673/314906/287670/114630/288088/300218/689938/29477/287796/297990/295455/294281/50663/300160/293453/316531/311078/25617/116484/498736/25679/64158/307351/305846/296554/25369/25750/500377 |
| M1a_vs_M0 | rno05150 | Staphylococcus aureus infection | 25 | 0.79511 | 2.740122 | 2.552E-10 | 4.096E-08 | 3.022E-08 | 542 | tags=52%, list=7%, signal=48% | 309622/309621/294269/294270/298566/362634/289211/29687/304966/54249/294273/24232/155012 |
| M1a_vs_M0 | rno04141 | Protein processing in endoplasmic reticulum | 125 | -0.464307 | -2.437917 | 5.851E-10 | 6.260E-08 | 4.619E-08 | 1966 | tags=55%, list=26%, signal=41% | 499751/361712/114590/289754/362912/83809/297994/297504/291673/89842/116643/287276/60331/361381/293721/690038/290783/311483/500972/289085/361367/58817/117030/685144/298012/290994/81784/116967/246146/313648/140639/171129/689134/304766/298943/29154/100910823/25506/310843/54318/192275/407784/29468/25596/29144/295235/63880/362226/297522/295243/64701/312903/286900/25617/100362805/361233/363160/298068/80843/116666/171562/116598/286906/192235/362862/360734/64202/362040/362859 |
| M1a_vs_M0 | rno05152 | Tuberculosis | 90 | 0.517318 | 2.348655 | 3.108E-08 | 2.350E-06 | 1.734E-06 | 714 | tags=23%, list=10%, signal=21% | 309622/85483/309621/294269/25599/294270/289211/24494/304966/171378/310659/450223/294273/29414/499271/24232/116465/108348076/171140/64171/116554 |
| M1a_vs_M0 | rno00190 | Oxidative phosphorylation | 86 | -0.499679 | -2.43229 | 3.660E-08 | 2.350E-06 | 1.734E-06 | 2697 | tags=73%, list=36%, signal=47% | 25488/688869/291660/690848/300677/100188937/641434/681024/362837/303393/362344/171374/192241/499529/54322/298103/299971/688386/100361008/301011/81728/685322/65262/299954/362897/301427/362440/89786/64539/116550/497902/361385/25282/252934/295923/301123/294964/94271/298451/245965/289217/299159/362588/299739/301458/363061/116664/300047/29754/293991/293130/297566/170667/297797/294504/114630/288088/689938/94170/297990/293453/117596/685232 |
| M1a_vs_M0 | rno05014 | Amyotrophic lateral sclerosis | 220 | -0.361718 | -2.074143 | 4.402E-08 | 2.355E-06 | 1.738E-06 | 2245 | tags=53%, list=30%, signal=38% | 155423/362504/29425/362344/171374/192241/499529/54322/60431/114558/53372/24887/24888/29748/688386/292085/100361008/65138/29668/309159/114590/289754/362912/303353/301011/81728/685322/65262/299954/117262/85492/29677/117550/287845/362897/301427/362440/304971/363720/116643/289990/64539/316333/116550/497902/29666/361385/29513/25282/252934/362335/25581/307821/29675/29676/295923/301123/64303/294964/94271/362152/81827/290527/245965/299706/365388/289217/362588/299739/64639/117263/81754/29671/83806/301458/363061/161475/171041/300047/292766/29336/29670/287772/287984/29669/317385/29754/293991/293130/54318/29673/298075/314906/246294/287670/114630/288088/300218/689938/297990/297522/315352/293453/316531/311078/308416/25617/690585/362687/498736/25679/64158/295692/307351/296554/116555/500377 |
| M1a_vs_M0 | rno04658 | Th1 and Th2 cell differentiation | 41 | 0.644823 | 2.462899 | 9.662E-08 | 4.323E-06 | 3.189E-06 | 927 | tags=44%, list=12%, signal=39% | 309622/309621/294269/294270/24932/25496/314322/171378/313050/316241/156726/294273/116465/24516/54267/116554/100361294/689844 |
| M1a_vs_M0 | rno04659 | Th17 cell differentiation | 49 | 0.597963 | 2.387519 | 1.077E-07 | 4.323E-06 | 3.189E-06 | 823 | tags=35%, list=11%, signal=31% | 309622/309621/294269/294270/25690/24932/24494/314322/171378/313050/316241/294273/116465/24516/308977/116554/100361294 |
| M1a_vs_M0 | rno05322 | Systemic lupus erythematosus | 29 | 0.697959 | 2.454814 | 1.369E-07 | 4.394E-06 | 3.242E-06 | 615 | tags=45%, list=8%, signal=41% | 309622/309621/294269/294270/298566/362634/29687/306970/304966/25408/294273/24232/291157 |
| M1a_vs_M0 | rno05164 | Influenza A | 87 | 0.504011 | 2.276018 | 1.348E-07 | 4.394E-06 | 3.242E-06 | 951 | tags=28%, list=13%, signal=24% | 309622/85483/309621/294269/294270/286918/293624/245920/25023/81635/192281/24575/317468/24494/89829/294273/29414/116465/361384/100361294/364594/294392/304277/366508 |
| M1a_vs_M0 | rno05140 | Leishmaniasis | 34 | 0.668683 | 2.441383 | 2.660E-07 | 7.761E-06 | 5.726E-06 | 614 | tags=41%, list=8%, signal=38% | 309622/309621/294269/294270/25023/24494/304966/314322/315139/81520/294273/24232/116465/24516 |
| M1a_vs_M0 | rno05010 | Alzheimer disease | 227 | -0.341962 | -1.966594 | 2.977E-07 | 7.962E-06 | 5.875E-06 | 1924 | tags=51%, list=26%, signal=39% | 29425/362344/171374/192241/499529/54322/114558/309361/25233/29748/688386/100361008/65138/29668/309159/289754/303353/301011/81728/685322/65262/299954/117262/85492/29677/117550/362897/301427/362440/289990/64539/116550/497902/29666/361385/361986/83721/25282/252934/25581/307821/29675/29676/170851/25031/29192/295923/301123/294964/94271/81827/282819/309295/245965/293621/365388/289217/362588/299739/64639/117263/29671/83806/301458/363061/161475/83765/171041/300047/84021/64026/306009/292766/24703/29670/287772/287984/29669/29154/25715/29754/293991/293130/54318/29673/314906/287670/114630/288088/300218/689938/29477/287796/299858/78965/297990/24525/295455/294281/50663/293453/316531/311078/24493/24605/498736/25679/64158/307351/25391/305846/296554/24539/24654/500377 |
| M1a_vs_M0 | rno05016 | Huntington disease | 184 | -0.363557 | -2.043026 | 3.779E-07 | 9.332E-06 | 6.885E-06 | 2227 | tags=53%, list=30%, signal=38% | 362504/29425/362344/171374/192241/499529/54322/114558/24887/688386/100361008/65138/29668/309159/303353/301011/308578/81728/685322/65262/299954/117262/85492/29677/117550/287845/362897/301427/362440/116561/65046/289990/100361574/64539/316333/116550/497902/29666/361385/298400/361365/25282/252934/25581/307821/29675/29676/25031/295923/301123/294964/94271/81827/282819/245965/83503/365388/289217/362588/299739/117263/29671/83806/303968/301458/363061/161475/171041/300047/292766/29670/287772/287984/29669/29754/293991/293130/29673/314906/287670/114630/288088/300218/689938/297990/293453/316531/311078/498736/64158/307351/56083/296554/362165/24654/500131/500377 |
| M1a_vs_M0 | rno05171 | Coronavirus disease - COVID-19 | 140 | 0.430734 | 2.102459 | 1.246E-06 | 2.857E-05 | 2.108E-05 | 2674 | tags=72%, list=36%, signal=47% | 286918/298566/362634/245920/25023/29687/81635/192281/24575/317468/24494/314322/298693/54249/24232/24516/317646/116554/100361294/364594/366508/29752/24499/684440/94266/24237/287362/296545/25493/29260/81775/117053/58927/64307/64638/362041/290641/29284/29283/81763/291434/29285/65139/245981/124440/117042/29304/295340/100360679/122772/66021/29287/102555453/294282/287417/29282/64205/81774/24231/25166/122799/100362640/363248/64298/25738/293418/300079/296709/311245/140655/499782/25538/65136/81764/29236/292892/124323/691531/81768/83789/140654/64640/64306/499801/103689992/360572/81776/29741/64156/100362400/309165/362631/81767/81766/689284/81729/25155/25625/297755/84351/300177 |
| M1a_vs_M0 | rno05145 | Toxoplasmosis | 54 | 0.558847 | 2.281637 | 1.418E-06 | 3.035E-05 | 2.239E-05 | 823 | tags=30%, list=11%, signal=27% | 309622/85483/309621/294269/294270/114495/117029/24963/497932/315139/294273/29414/116465/108348108/116554/100361294 |
| M1a_vs_M0 | rno04612 | Antigen processing and presentation | 47 | 0.557532 | 2.207405 | 3.691E-06 | 7.405E-05 | 5.463E-05 | 525 | tags=23%, list=7%, signal=22% | 309622/85483/309621/294269/25599/294270/24932/24963/310659/294273/414792 |
| M1a_vs_M0 | rno03050 | Proteasome | 41 | -0.575665 | -2.386696 | 4.358E-06 | 8.228E-05 | 6.071E-05 | 2218 | tags=73%, list=30%, signal=52% | 29425/288455/65138/29668/303353/117262/85492/29677/289990/29666/25581/307821/29675/29676/81827/29614/365388/117263/29671/83806/161475/287716/292766/29670/287772/287984/29669/29673/287670/311078 |
| M1a_vs_M0 | rno05321 | Inflammatory bowel disease | 22 | 0.705658 | 2.353914 | 4.991E-06 | 8.901E-05 | 6.567E-05 | 713 | tags=45%, list=9%, signal=41% | 309622/309621/294269/294270/24494/294273/116465/24516/308977/54267 |
| M1a_vs_M0 | rno05020 | Prion disease | 168 | -0.346542 | -1.918511 | 5.294E-06 | 8.944E-05 | 6.599E-05 | 2218 | tags=57%, list=30%, signal=41% | 29425/362344/171374/192241/499529/54322/24887/29748/688386/100361008/65138/29668/309159/303353/301011/81728/685322/65262/299954/117262/85492/29677/117550/362897/301427/362440/289990/64539/116550/497902/29666/361385/298400/29513/25282/24686/252934/25581/307821/29675/29676/295923/301123/294964/94271/81827/282819/245965/79129/365388/289217/362588/25404/299739/64639/117263/29671/83806/301458/363061/161475/171041/300047/292766/29670/287772/287984/29669/192277/29754/293991/293130/54318/29673/314906/287670/114630/288088/300218/689938/297990/293453/316531/311078/24493/25617/24586/498736/25679/64158/307351/296554/362165/500131/500377 |
| M1a_vs_M0 | rno01250 | Biosynthesis of nucleotide sugars | 29 | -0.621443 | -2.351688 | 1.055E-05 | 1.694E-04 | 1.250E-04 | 1347 | tags=55%, list=18%, signal=45% | 83472/292804/24645/298071/302915/363109/287835/291095/310935/297393/363145/498272/114860/25060/498486/360518 |
| M1a_vs_M0 | rno05332 | Graft-versus-host disease | 19 | 0.710574 | 2.270345 | 1.254E-05 | 1.917E-04 | 1.415E-04 | 525 | tags=42%, list=7%, signal=39% | 309622/309621/294269/294270/24494/25408/294273/414792 |
| M1a_vs_M0 | rno00510 | N-Glycan biosynthesis | 40 | -0.566167 | -2.341953 | 1.641E-05 | 2.394E-04 | 1.766E-04 | 1772 | tags=60%, list=24%, signal=46% | 300668/293721/290783/296394/25478/500972/296624/116967/313648/295051/311847/192275/360475/108349244/287983/25596/24390/295243/64701/362547/245960/363160/362040/315212 |
| M1a_vs_M0 | rno05330 | Allograft rejection | 18 | 0.701546 | 2.220965 | 2.392E-05 | 3.339E-04 | 2.464E-04 | 525 | tags=39%, list=7%, signal=36% | 309622/309621/294269/294270/25408/294273/414792 |
| M1a_vs_M0 | rno04672 | Intestinal immune network for IgA production | 15 | 0.729677 | 2.18746 | 3.103E-05 | 4.150E-04 | 3.062E-04 | 870 | tags=60%, list=12%, signal=53% | 309622/309621/294269/294270/25408/294273/690369/499415/360640 |
| M1a_vs_M0 | rno01230 | Biosynthesis of amino acids | 37 | -0.549497 | -2.208499 | 3.332E-05 | 4.278E-04 | 3.156E-04 | 886 | tags=41%, list=12%, signal=36% | 25630/25721/299857/59085/29592/29562/79250/81670/24849/114096/24644/24333/25741/24642/29221 |

# Supplementary Table S18. GSEA results for M1b vs. M0

| **comparison** | **ID** | **Description** | **setSize** | **enrichmentScore** | **NES** | **pvalue** | **p.adjust** | **qvalue** | **rank** | **leading_edge** | **core_enrichment** |
| --- | --- | --- | --- | --- | --- | --- | --- | --- | --- | --- | --- |
| M1b_vs_M0 | rno04060 | Cytokine-cytokine receptor interaction | 90 | 0.68212 | 3.335727 | 1.000E-10 | 3.633E-09 | 2.819E-09 | 521 | tags=38%, list=6%, signal=36% | 155140/24494/24835/25610/81503/171551/114105/24493/245920/365395/246759/60582/252963/305236/25325/353218/24498/29455/680609/25542/362076/287910/116637/360579/497942/60628/116465/117029/29454/364031/24932/25069/308977/360697 |
| M1b_vs_M0 | rno05323 | Rheumatoid arthritis | 54 | 0.712 | 3.125694 | 1.000E-10 | 3.633E-09 | 2.819E-09 | 240 | tags=33%, list=3%, signal=33% | 155140/24494/24835/81503/171551/114105/24493/24984/294269/309621/309622/294270/24498/25408/83785/25464/25542/56822 |
| M1b_vs_M0 | rno05144 | Malaria | 37 | 0.767499 | 3.08837 | 1.000E-10 | 3.633E-09 | 2.819E-09 | 343 | tags=46%, list=4%, signal=44% | 24494/24440/24835/360504/25610/287167/25361/25325/24498/25464/494203/494194/24934/338457/297666/494206/24553 |
| M1b_vs_M0 | rno05321 | Inflammatory bowel disease | 33 | 0.766194 | 3.001761 | 1.000E-10 | 3.633E-09 | 2.819E-09 | 521 | tags=48%, list=6%, signal=46% | 155140/24494/24835/24493/24984/294269/309621/309622/294270/25325/24498/81736/116465/291912/308977/360697 |
| M1b_vs_M0 | rno04668 | TNF signaling pathway | 83 | 0.620192 | 2.969792 | 1.000E-10 | 3.633E-09 | 2.819E-09 | 411 | tags=30%, list=5%, signal=29% | 24494/24835/81503/171551/114105/29527/245920/117279/78971/25361/687813/25493/303200/24498/24508/683206/25464/29146/24253/24517/81646/81736/312937/680611/291912 |
| M1b_vs_M0 | rno05152 | Tuberculosis | 114 | 0.551048 | 2.80846 | 1.000E-10 | 3.633E-09 | 2.819E-09 | 531 | tags=23%, list=6%, signal=22% | 155140/24494/24835/450223/24493/24984/294269/309621/25599/309622/294270/25325/24498/24253/60350/289211/85483/338457/305354/81646/81736/116465/291912/362418/360697/64171 |
| M1b_vs_M0 | rno04064 | NF-kappa B signaling pathway | 69 | 0.626398 | 2.800889 | 1.000E-10 | 3.633E-09 | 2.819E-09 | 429 | tags=30%, list=5%, signal=29% | 24494/24835/81503/171551/114105/29527/117279/78971/25361/687813/25493/683206/299626/25464/60350/309452/170929/116637/81736/312937/313050 |
| M1b_vs_M0 | rno05164 | Influenza A | 98 | 0.557516 | 2.753663 | 1.000E-10 | 3.633E-09 | 2.819E-09 | 442 | tags=22%, list=5%, signal=22% | 24494/24835/24493/245920/24984/294269/309621/309622/294270/287362/25493/24498/293624/25464/81525/286918/85483/81736/116465/317468/24575/192281 |
| M1b_vs_M0 | rno05310 | Asthma | 10 | 0.962587 | 2.742909 | 1.000E-10 | 3.633E-09 | 2.819E-09 | 77 | tags=70%, list=1%, signal=69% | 24835/24984/294269/309621/309622/294270/25325 |
| M1b_vs_M0 | rno05143 | African trypanosomiasis | 21 | 0.8351 | 2.844843 | 1.775E-10 | 5.805E-09 | 4.503E-09 | 237 | tags=52%, list=3%, signal=51% | 24494/24440/24835/360504/287167/25361/25325/24498/25464/25081/338457 |
| M1b_vs_M0 | rno05171 | Coronavirus disease - COVID-19 | 142 | 0.453657 | 2.350485 | 2.833E-10 | 8.421E-09 | 6.533E-09 | 1326 | tags=43%, list=16%, signal=37% | 24494/24835/25610/29287/294257/245920/100361715/29304/287362/25493/24498/81525/298693/286918/362634/140662/287417/117042/117053/298566/57809/81736/100362640/317468/29687/83789/24575/192281/54249/689284/81768/58927/25023/28298/24231/499801/29752/300079/684440/100360679/292892/81776/24499/122772/25513/64205/362041/293418/81767/29288/29283/296545/296709/81729/64307/686326/682147/103689992/25166/124323/29236 |
| M1b_vs_M0 | rno04061 | Viral protein interaction with cytokine and cytokine receptor | 36 | 0.717523 | 2.871342 | 3.514E-10 | 9.575E-09 | 7.429E-09 | 400 | tags=42%, list=5%, signal=40% | 24835/81503/171551/114105/245920/246759/305236/25325/24498/25542/287910/116637/360579/60628/117029 |
| M1b_vs_M0 | rno05140 | Leishmaniasis | 51 | 0.657763 | 2.825049 | 6.401E-10 | 1.610E-08 | 1.249E-08 | 895 | tags=41%, list=11%, signal=37% | 24494/24835/24493/29527/24984/294269/309621/309622/294270/25493/25325/81525/81736/116465/81520/360697/25023/171361/24514/304966/500904 |
| M1b_vs_M0 | rno05332 | Graft-versus-host disease | 23 | 0.81715 | 2.936268 | 7.466E-10 | 1.628E-08 | 1.263E-08 | 376 | tags=70%, list=4%, signal=67% | 24494/24835/24493/24984/294269/309621/309622/294270/24498/24988/25408/414792/24974/56822/414788/294228 |
| M1b_vs_M0 | rno05169 | Epstein-Barr virus infection | 148 | 0.438587 | 2.307098 | 7.459E-10 | 1.628E-08 | 1.263E-08 | 665 | tags=24%, list=8%, signal=23% | 24835/245920/24984/294269/309621/309622/294270/29577/25493/303200/24498/293624/24988/156726/114851/683206/299626/25464/81525/298693/414792/83571/309452/316241/24974/414788/81736/294228/192281/25217/362817/24747/100360982/114495/367901/64547 |
| M1b_vs_M0 | rno04672 | Intestinal immune network for IgA production | 21 | 0.814925 | 2.776115 | 1.989E-09 | 4.064E-08 | 3.153E-08 | 309 | tags=48%, list=4%, signal=46% | 24984/294269/309621/309622/294270/25325/24498/25408/56822/60628 |
| M1b_vs_M0 | rno05150 | Staphylococcus aureus infection | 27 | 0.77254 | 2.872492 | 2.517E-09 | 4.741E-08 | 3.678E-08 | 448 | tags=48%, list=5%, signal=46% | 294257/24984/294269/309621/309622/294270/25325/25464/289211/362634/298566/29687/54249 |
| M1b_vs_M0 | rno05168 | Herpes simplex virus 1 infection | 120 | 0.474956 | 2.451205 | 2.610E-09 | 4.741E-08 | 3.678E-08 | 536 | tags=22%, list=6%, signal=21% | 24494/24835/24984/294269/309621/78971/25599/309622/294270/25493/24498/293624/24988/414792/299314/24974/338457/414788/81736/116465/294228/192281/308417/25217/360697/64171/24747 |
| M1b_vs_M0 | rno04657 | IL-17 signaling pathway | 53 | 0.623156 | 2.736489 | 4.654E-09 | 8.009E-08 | 6.213E-08 | 156 | tags=23%, list=2%, signal=22% | 24494/24835/25610/81503/171551/114105/29527/245920/25493/24498/683206/24253 |
| M1b_vs_M0 | rno05133 | Pertussis | 48 | 0.647196 | 2.755077 | 5.173E-09 | 8.458E-08 | 6.562E-08 | 404 | tags=27%, list=5%, signal=26% | 155140/24494/24835/24493/287362/25325/24498/24508/60350/362634/298566/81736/29687 |
| M1b_vs_M0 | rno04620 | Toll-like receptor signaling pathway | 70 | 0.554982 | 2.521108 | 7.457E-09 | 1.161E-07 | 9.009E-08 | 403 | tags=26%, list=5%, signal=25% | 24494/24835/245920/246759/25493/303200/305236/24498/293624/25408/25542/60350/338457/56822/305354/116637/81736/317468 |
| M1b_vs_M0 | rno05322 | Systemic lupus erythematosus | 36 | 0.686249 | 2.746192 | 9.763E-09 | 1.451E-07 | 1.126E-07 | 404 | tags=36%, list=5%, signal=35% | 24835/24984/294269/309621/309622/294270/25325/25408/362634/56822/298566/117056/29687 |
| M1b_vs_M0 | rno04621 | NOD-like receptor signaling pathway | 119 | 0.450414 | 2.328754 | 1.356E-08 | 1.927E-07 | 1.495E-07 | 531 | tags=18%, list=6%, signal=18% | 24494/24835/81503/171551/114105/78971/362050/287362/25493/58923/24498/293624/683206/81525/298784/81736/685067/291912/192281/252961/305633/64171 |
| M1b_vs_M0 | rno05330 | Allograft rejection | 22 | 0.78003 | 2.727212 | 2.248E-08 | 3.063E-07 | 2.376E-07 | 376 | tags=64%, list=4%, signal=61% | 24835/24984/294269/309621/309622/294270/25325/24988/25408/414792/24974/56822/414788/294228 |
| M1b_vs_M0 | rno04625 | C-type lectin receptor signaling pathway | 79 | 0.520959 | 2.484712 | 2.436E-08 | 3.186E-07 | 2.472E-07 | 542 | tags=23%, list=6%, signal=22% | 155140/24494/24835/450223/29527/287362/25493/25325/24498/362432/24508/309452/25148/81736/312937/680611/64171/100360982 |
