# Supplementary material for: Ferroptosis-associated myeloid cell heterogeneity and inflammatory amplification following spinal cord injury
Source: Front Immunol. 2026 Apr 22;17:1831161. doi: 10.3389/fimmu.2026.1831161 (PMC13143767; doi:10.3389/fimmu.2026.1831161)
Supplement: Supplementary file 1 [file DataSheet1.zip › supplementary_Table_S14.docx]

**Supplementary Table S15. ssGSEA scores**

| **Immune_Cell** | **GSM1095725** | **GSM1095726** | **GSM1095727** | **GSM1095728** | **GSM1095733** | **GSM1095734** | **GSM1095735** | **GSM1095736** | **GSM1095737** | **GSM1095738** | **GSM1095739** | **GSM1095740** | **GSM1095741** | **GSM1095742** | **GSM1095743** | **GSM1095744** |
| --- | --- | --- | --- | --- | --- | --- | --- | --- | --- | --- | --- | --- | --- | --- | --- | --- |
| Activated B cell | 0.09249457 | 0.01181716 | 0.06461861 | 0.04224326 | -0.23425046 | 0.15440022 | 0.13066095 | 0.1196081 | 0.07694064 | 0.09519021 | 0.37340943 | 0.13480765 | -0.16445868 | -0.35605958 | -0.32098922 | -0.0341308 |
| Activated CD4 T cell | -0.32072465 | -0.59780489 | -0.35414291 | -0.47062929 | 0.10574701 | 0.45964642 | -0.31776905 | 0.05923799 | 0.26608109 | 0.37991041 | 0.42009054 | 0.55358584 | -0.18047164 | 0.33730688 | 0.15098721 | 0.13773057 |
| Activated CD8 T cell | 0.08732528 | -0.21706398 | -0.19661156 | -0.30270193 | -0.0847348 | 0.28714738 | -0.16962703 | 0.01531077 | 0.33374558 | 0.06725748 | 0.39028643 | 0.12337887 | -0.28040691 | -0.16154595 | -0.19472682 | 0.08099087 |
| Activated dendritic cell | -0.49349339 | -0.51330655 | -0.33191737 | -0.4154828 | 0.41309363 | 0.6004487 | -0.28537319 | -0.0806566 | 0.54148385 | 0.53337838 | 0.04174268 | 0.33547744 | -0.32887837 | -0.07448019 | 0.04527842 | -0.30773434 |
| CD56bright natural killer cell | 0.04854151 | 0.05812842 | 0.02196965 | -0.00988286 | 0.12168893 | 0.10792099 | 0.07873325 | 0.07603764 | -0.1822191 | -0.03624849 | 0.18465032 | -0.26127929 | -0.26854697 | 0.14440629 | -0.01865852 | -0.02684045 |
| CD56dim natural killer cell | 0.16880076 | 0.0489741 | -0.14385955 | -0.15895538 | 0.34926838 | -0.05149046 | 0.19570373 | 0.20318287 | -0.20563134 | -0.04359319 | -0.03983978 | -0.11634044 | 0.12424165 | -0.00037998 | -0.38336976 | 0.07216698 |
| Central memory CD4 T cell | -0.7005377 | -0.64389833 | -0.55859333 | -0.51746452 | 0.43403746 | 0.53970257 | 0.26608353 | 0.23656247 | 0.18661018 | 0.53223142 | -0.15796769 | 0.48117138 | -0.15591018 | 0.37593034 | 0.40631284 | -0.19412074 |
| Central memory CD8 T cell | -0.35897334 | -0.47218956 | -0.15635124 | -0.21789686 | 0.24363126 | 0.55539741 | -0.08869878 | 0.26743844 | -0.087619 | 0.12515529 | 0.01019868 | 0.2890261 | -0.35656116 | -0.04238421 | 0.47658127 | -0.32719955 |
| Effector memeory CD4 T cell | -0.44157234 | -0.10984431 | -0.19492169 | -0.2130366 | 0.53749809 | 0.19298926 | 0.35076535 | 0.35670425 | -0.43316572 | 0.49379577 | -0.31289534 | 0.17405507 | -0.17309093 | 0.34112536 | 0.13027483 | -0.26425302 |
| Effector memeory CD8 T cell | -0.06777522 | -0.45804962 | 0.20542876 | -0.01627399 | 0.3292295 | 0.29904459 | -0.06586485 | -0.11867751 | 0.4355279 | 0.17532935 | 0.10402853 | 0.43375472 | -0.02830571 | -0.33253356 | -0.30849521 | -0.24430152 |
| Eosinophil | 0.476021 | -0.03314486 | 0.17780561 | 0.07825223 | 0.06763202 | -0.14213506 | 0.07619214 | 0.29576783 | 0.03766267 | -0.23211454 | 0.16014565 | 0.0409136 | -0.44405002 | 0.2091609 | -0.23550913 | 0.09488171 |
| Gamma delta T cell | -0.54482125 | -0.50777146 | -0.54061755 | -0.42448252 | 0.22950563 | 0.4574953 | 0.31410333 | 0.22184879 | 0.35134318 | 0.35600823 | 0.23897154 | 0.42863333 | -0.15272132 | 0.11650031 | 0.14425582 | -0.0324285 |
| Immature B cell | -0.48431269 | -0.49627692 | -0.63238542 | -0.40654552 | 0.6635053 | 0.69009873 | 0.32805413 | 0.45288537 | 0.43474807 | 0.28721044 | 0.45191584 | 0.47976547 | -0.57639757 | -0.28249934 | -0.39875081 | -0.52194995 |
| Immature dendritic cell | -0.36864055 | -0.14976104 | 0.119528 | -0.03550199 | 0.34904609 | 0.20188639 | 0.2610219 | 0.30652186 | -0.20949927 | 0.345821 | -0.20948367 | 0.36043821 | -0.35664824 | -0.04446086 | 0.1289926 | -0.45829316 |
| Macrophage | -0.15136554 | -0.32738828 | -0.31465978 | 0.05031713 | 0.1507053 | 0.27144263 | 0.10315454 | 0.21213304 | 0.12059194 | 0.29023918 | -0.09910064 | 0.1129425 | -0.21508645 | -0.34174926 | 0.07060603 | -0.04079823 |
| Mast cell | 0.12560224 | -0.02681441 | -0.09709655 | -0.03858061 | -0.16523496 | 0.27516894 | 0.13935322 | 0.25510726 | 0.01718794 | -0.05559543 | 0.58093252 | 0.36497111 | -0.28957407 | -0.52325154 | -0.52646027 | -0.26379307 |
| MDSC | -0.62812129 | -0.68243111 | -0.61283764 | -0.47555594 | 0.36409811 | 0.39631465 | -0.03636463 | 0.31518349 | 0.42227556 | 0.44581699 | 0.19064956 | 0.60985809 | -0.26875451 | 0.27235092 | 0.19202223 | -0.01114482 |
| Memory B cell | -0.275576 | -0.57482919 | -0.31163189 | -0.46543201 | 0.17336913 | 0.1298198 | 0.19605507 | -0.0690145 | 0.39419129 | 0.44415083 | 0.19262415 | 0.27674871 | 0.00235868 | 0.22385132 | -0.00755783 | -0.10661477 |
| Monocyte | -0.50899541 | -0.24399434 | -0.07412402 | -0.07309516 | 0.54143294 | 0.39656888 | 0.38144186 | 0.49930965 | -0.14861097 | 0.44415945 | -0.00555676 | 0.41454664 | -0.51263023 | -0.31478755 | 0.12389207 | -0.40531481 |
| Natural killer cell | -0.4482684 | -0.30760344 | -0.44205014 | -0.29821241 | 0.35751017 | 0.18146649 | 0.18143003 | 0.2457088 | 0.33679593 | 0.31905204 | 0.1121178 | 0.48817485 | -0.12884764 | -0.07571005 | 0.08784996 | -0.26287066 |
| Natural killer T cell | 0.00301941 | -0.26776741 | -0.23058146 | -0.23535212 | 0.2769289 | 0.19276598 | 0.09866845 | 0.04696165 | 0.19481912 | 0.07963146 | 0.38217252 | 0.27337464 | -0.23105807 | -0.19311966 | -0.20808711 | -0.16679047 |
| Neutrophil | 0.50770976 | 0.09041567 | -0.29540641 | 0.19836183 | 0.04249538 | 0.37561287 | 0.14079143 | -0.44703659 | 0.12474537 | -0.42152675 | 0.47961026 | -0.32908032 | 0.11906087 | 0.15489881 | -0.30282068 | -0.24338099 |
| Plasmacytoid dendritic cell | -0.42450375 | -0.22623296 | -0.07736246 | -0.19660503 | 0.2855853 | 0.24992303 | 0.20003135 | 0.35463865 | -0.1155296 | 0.29090657 | -0.15342874 | 0.36583632 | -0.20567757 | 0.10202515 | 0.08977962 | -0.3867077 |
| Regulatory T cell | -0.65893957 | -0.59419351 | -0.47875709 | -0.4706173 | 0.60431555 | 0.63969803 | 0.1741055 | 0.54964506 | 0.32019244 | 0.4305893 | -0.19266419 | 0.50891225 | -0.47841684 | 0.11921283 | -0.18146967 | -0.31348328 |
| T follicular helper cell | -0.33800404 | -0.09767921 | -0.32342437 | -0.07236089 | 0.51171631 | 0.38895245 | 0.24007267 | 0.32738122 | -0.0582232 | 0.12280981 | 0.00742374 | 0.15321875 | -0.20260044 | -0.19910287 | 0.09959216 | -0.18487454 |
| Type 1 T helper cell | -0.15165886 | -0.21504645 | -0.24110856 | -0.22210304 | 0.1792233 | 0.29816119 | 0.03108838 | -0.08385734 | 0.09495916 | 0.29201139 | 0.20111901 | 0.33031788 | -0.17997185 | -0.19456243 | -0.20893984 | -0.28897733 |
| Type 17 T helper cell | 0.4455428 | 0.11126779 | 0.16795318 | -0.09962236 | -0.26099504 | 0.01989569 | -0.19156697 | 0.02310668 | 0.26530189 | -0.11983468 | 0.40605665 | -0.02405231 | 0.1433708 | -0.44242142 | -0.52390019 | -0.15931216 |
| Type 2 T helper cell | 0.00458329 | 0.06195733 | -0.26167734 | 0.09266061 | -0.0169113 | -0.18874212 | 0.02448726 | -0.14403024 | -0.06387285 | 0.17253003 | -0.0655967 | 0.0778593 | 0.244188 | 0.1794443 | 0.19183564 | 0.12793026 |

**Supplementary Table S15. ssGSEA scores (long format with group labels)**

| **Immune_Cell** | **Sample** | **ssGSEA_score** | **Group** |
| --- | --- | --- | --- |
| Activated B cell | GSM1095725 | 0.09249457 | Sham |
| Activated CD4 T cell | GSM1095725 | -0.32072465 | Sham |
| Activated CD8 T cell | GSM1095725 | 0.08732528 | Sham |
| Activated dendritic cell | GSM1095725 | -0.49349339 | Sham |
| CD56bright natural killer cell | GSM1095725 | 0.04854151 | Sham |
| CD56dim natural killer cell | GSM1095725 | 0.16880076 | Sham |
| Central memory CD4 T cell | GSM1095725 | -0.7005377 | Sham |
| Central memory CD8 T cell | GSM1095725 | -0.35897334 | Sham |
| Effector memeory CD4 T cell | GSM1095725 | -0.44157234 | Sham |
| Effector memeory CD8 T cell | GSM1095725 | -0.06777522 | Sham |
| Eosinophil | GSM1095725 | 0.476021 | Sham |
| Gamma delta T cell | GSM1095725 | -0.54482125 | Sham |
| Immature B cell | GSM1095725 | -0.48431269 | Sham |
| Immature dendritic cell | GSM1095725 | -0.36864055 | Sham |
| Macrophage | GSM1095725 | -0.15136554 | Sham |
| Mast cell | GSM1095725 | 0.12560224 | Sham |
| MDSC | GSM1095725 | -0.62812129 | Sham |
| Memory B cell | GSM1095725 | -0.275576 | Sham |
| Monocyte | GSM1095725 | -0.50899541 | Sham |
| Natural killer cell | GSM1095725 | -0.4482684 | Sham |
| Natural killer T cell | GSM1095725 | 0.00301941 | Sham |
| Neutrophil | GSM1095725 | 0.50770976 | Sham |
| Plasmacytoid dendritic cell | GSM1095725 | -0.42450375 | Sham |
| Regulatory T cell | GSM1095725 | -0.65893957 | Sham |
| T follicular helper cell | GSM1095725 | -0.33800404 | Sham |
| Type 1 T helper cell | GSM1095725 | -0.15165886 | Sham |
| Type 17 T helper cell | GSM1095725 | 0.4455428 | Sham |
| Type 2 T helper cell | GSM1095725 | 0.00458329 | Sham |
| Activated B cell | GSM1095726 | 0.01181716 | Sham |
| Activated CD4 T cell | GSM1095726 | -0.59780489 | Sham |
| Activated CD8 T cell | GSM1095726 | -0.21706398 | Sham |
| Activated dendritic cell | GSM1095726 | -0.51330655 | Sham |
| CD56bright natural killer cell | GSM1095726 | 0.05812842 | Sham |
| CD56dim natural killer cell | GSM1095726 | 0.0489741 | Sham |
| Central memory CD4 T cell | GSM1095726 | -0.64389833 | Sham |
| Central memory CD8 T cell | GSM1095726 | -0.47218956 | Sham |
| Effector memeory CD4 T cell | GSM1095726 | -0.10984431 | Sham |
| Effector memeory CD8 T cell | GSM1095726 | -0.45804962 | Sham |
| Eosinophil | GSM1095726 | -0.03314486 | Sham |
| Gamma delta T cell | GSM1095726 | -0.50777146 | Sham |
| Immature B cell | GSM1095726 | -0.49627692 | Sham |
| Immature dendritic cell | GSM1095726 | -0.14976104 | Sham |
| Macrophage | GSM1095726 | -0.32738828 | Sham |
| Mast cell | GSM1095726 | -0.02681441 | Sham |
| MDSC | GSM1095726 | -0.68243111 | Sham |
| Memory B cell | GSM1095726 | -0.57482919 | Sham |
| Monocyte | GSM1095726 | -0.24399434 | Sham |
| Natural killer cell | GSM1095726 | -0.30760344 | Sham |
| Natural killer T cell | GSM1095726 | -0.26776741 | Sham |
| Neutrophil | GSM1095726 | 0.09041567 | Sham |
| Plasmacytoid dendritic cell | GSM1095726 | -0.22623296 | Sham |
| Regulatory T cell | GSM1095726 | -0.59419351 | Sham |
| T follicular helper cell | GSM1095726 | -0.09767921 | Sham |
| Type 1 T helper cell | GSM1095726 | -0.21504645 | Sham |
| Type 17 T helper cell | GSM1095726 | 0.11126779 | Sham |
| Type 2 T helper cell | GSM1095726 | 0.06195733 | Sham |
| Activated B cell | GSM1095727 | 0.06461861 | Sham |
| Activated CD4 T cell | GSM1095727 | -0.35414291 | Sham |
| Activated CD8 T cell | GSM1095727 | -0.19661156 | Sham |
| Activated dendritic cell | GSM1095727 | -0.33191737 | Sham |
| CD56bright natural killer cell | GSM1095727 | 0.02196965 | Sham |
| CD56dim natural killer cell | GSM1095727 | -0.14385955 | Sham |
| Central memory CD4 T cell | GSM1095727 | -0.55859333 | Sham |
| Central memory CD8 T cell | GSM1095727 | -0.15635124 | Sham |
| Effector memeory CD4 T cell | GSM1095727 | -0.19492169 | Sham |
| Effector memeory CD8 T cell | GSM1095727 | 0.20542876 | Sham |
| Eosinophil | GSM1095727 | 0.17780561 | Sham |
| Gamma delta T cell | GSM1095727 | -0.54061755 | Sham |
| Immature B cell | GSM1095727 | -0.63238542 | Sham |
| Immature dendritic cell | GSM1095727 | 0.119528 | Sham |
| Macrophage | GSM1095727 | -0.31465978 | Sham |
| Mast cell | GSM1095727 | -0.09709655 | Sham |
| MDSC | GSM1095727 | -0.61283764 | Sham |
| Memory B cell | GSM1095727 | -0.31163189 | Sham |
| Monocyte | GSM1095727 | -0.07412402 | Sham |
| Natural killer cell | GSM1095727 | -0.44205014 | Sham |
| Natural killer T cell | GSM1095727 | -0.23058146 | Sham |
| Neutrophil | GSM1095727 | -0.29540641 | Sham |
| Plasmacytoid dendritic cell | GSM1095727 | -0.07736246 | Sham |
| Regulatory T cell | GSM1095727 | -0.47875709 | Sham |
| T follicular helper cell | GSM1095727 | -0.32342437 | Sham |
| Type 1 T helper cell | GSM1095727 | -0.24110856 | Sham |
| Type 17 T helper cell | GSM1095727 | 0.16795318 | Sham |
| Type 2 T helper cell | GSM1095727 | -0.26167734 | Sham |
| Activated B cell | GSM1095728 | 0.04224326 | Sham |
| Activated CD4 T cell | GSM1095728 | -0.47062929 | Sham |
| Activated CD8 T cell | GSM1095728 | -0.30270193 | Sham |
| Activated dendritic cell | GSM1095728 | -0.4154828 | Sham |
| CD56bright natural killer cell | GSM1095728 | -0.00988286 | Sham |
| CD56dim natural killer cell | GSM1095728 | -0.15895538 | Sham |
| Central memory CD4 T cell | GSM1095728 | -0.51746452 | Sham |
| Central memory CD8 T cell | GSM1095728 | -0.21789686 | Sham |
| Effector memeory CD4 T cell | GSM1095728 | -0.2130366 | Sham |
| Effector memeory CD8 T cell | GSM1095728 | -0.01627399 | Sham |
| Eosinophil | GSM1095728 | 0.07825223 | Sham |
| Gamma delta T cell | GSM1095728 | -0.42448252 | Sham |
| Immature B cell | GSM1095728 | -0.40654552 | Sham |
| Immature dendritic cell | GSM1095728 | -0.03550199 | Sham |
| Macrophage | GSM1095728 | 0.05031713 | Sham |
| Mast cell | GSM1095728 | -0.03858061 | Sham |
| MDSC | GSM1095728 | -0.47555594 | Sham |
| Memory B cell | GSM1095728 | -0.46543201 | Sham |
| Monocyte | GSM1095728 | -0.07309516 | Sham |
| Natural killer cell | GSM1095728 | -0.29821241 | Sham |
| Natural killer T cell | GSM1095728 | -0.23535212 | Sham |
| Neutrophil | GSM1095728 | 0.19836183 | Sham |
| Plasmacytoid dendritic cell | GSM1095728 | -0.19660503 | Sham |
| Regulatory T cell | GSM1095728 | -0.4706173 | Sham |
| T follicular helper cell | GSM1095728 | -0.07236089 | Sham |
| Type 1 T helper cell | GSM1095728 | -0.22210304 | Sham |
| Type 17 T helper cell | GSM1095728 | -0.09962236 | Sham |
| Type 2 T helper cell | GSM1095728 | 0.09266061 | Sham |
| Activated B cell | GSM1095733 | -0.23425046 | SCI_1d |
| Activated CD4 T cell | GSM1095733 | 0.10574701 | SCI_1d |
| Activated CD8 T cell | GSM1095733 | -0.0847348 | SCI_1d |
| Activated dendritic cell | GSM1095733 | 0.41309363 | SCI_1d |
| CD56bright natural killer cell | GSM1095733 | 0.12168893 | SCI_1d |
| CD56dim natural killer cell | GSM1095733 | 0.34926838 | SCI_1d |
| Central memory CD4 T cell | GSM1095733 | 0.43403746 | SCI_1d |
| Central memory CD8 T cell | GSM1095733 | 0.24363126 | SCI_1d |
| Effector memeory CD4 T cell | GSM1095733 | 0.53749809 | SCI_1d |
| Effector memeory CD8 T cell | GSM1095733 | 0.3292295 | SCI_1d |
| Eosinophil | GSM1095733 | 0.06763202 | SCI_1d |
| Gamma delta T cell | GSM1095733 | 0.22950563 | SCI_1d |
| Immature B cell | GSM1095733 | 0.6635053 | SCI_1d |
| Immature dendritic cell | GSM1095733 | 0.34904609 | SCI_1d |
| Macrophage | GSM1095733 | 0.1507053 | SCI_1d |
| Mast cell | GSM1095733 | -0.16523496 | SCI_1d |
| MDSC | GSM1095733 | 0.36409811 | SCI_1d |
| Memory B cell | GSM1095733 | 0.17336913 | SCI_1d |
| Monocyte | GSM1095733 | 0.54143294 | SCI_1d |
| Natural killer cell | GSM1095733 | 0.35751017 | SCI_1d |
| Natural killer T cell | GSM1095733 | 0.2769289 | SCI_1d |
| Neutrophil | GSM1095733 | 0.04249538 | SCI_1d |
| Plasmacytoid dendritic cell | GSM1095733 | 0.2855853 | SCI_1d |
| Regulatory T cell | GSM1095733 | 0.60431555 | SCI_1d |
| T follicular helper cell | GSM1095733 | 0.51171631 | SCI_1d |
| Type 1 T helper cell | GSM1095733 | 0.1792233 | SCI_1d |
| Type 17 T helper cell | GSM1095733 | -0.26099504 | SCI_1d |
| Type 2 T helper cell | GSM1095733 | -0.0169113 | SCI_1d |
| Activated B cell | GSM1095734 | 0.15440022 | SCI_1d |
| Activated CD4 T cell | GSM1095734 | 0.45964642 | SCI_1d |
| Activated CD8 T cell | GSM1095734 | 0.28714738 | SCI_1d |
| Activated dendritic cell | GSM1095734 | 0.6004487 | SCI_1d |
| CD56bright natural killer cell | GSM1095734 | 0.10792099 | SCI_1d |
| CD56dim natural killer cell | GSM1095734 | -0.05149046 | SCI_1d |
| Central memory CD4 T cell | GSM1095734 | 0.53970257 | SCI_1d |
| Central memory CD8 T cell | GSM1095734 | 0.55539741 | SCI_1d |
| Effector memeory CD4 T cell | GSM1095734 | 0.19298926 | SCI_1d |
| Effector memeory CD8 T cell | GSM1095734 | 0.29904459 | SCI_1d |
| Eosinophil | GSM1095734 | -0.14213506 | SCI_1d |
| Gamma delta T cell | GSM1095734 | 0.4574953 | SCI_1d |
| Immature B cell | GSM1095734 | 0.69009873 | SCI_1d |
| Immature dendritic cell | GSM1095734 | 0.20188639 | SCI_1d |
| Macrophage | GSM1095734 | 0.27144263 | SCI_1d |
| Mast cell | GSM1095734 | 0.27516894 | SCI_1d |
| MDSC | GSM1095734 | 0.39631465 | SCI_1d |
| Memory B cell | GSM1095734 | 0.1298198 | SCI_1d |
| Monocyte | GSM1095734 | 0.39656888 | SCI_1d |
| Natural killer cell | GSM1095734 | 0.18146649 | SCI_1d |
| Natural killer T cell | GSM1095734 | 0.19276598 | SCI_1d |
| Neutrophil | GSM1095734 | 0.37561287 | SCI_1d |
| Plasmacytoid dendritic cell | GSM1095734 | 0.24992303 | SCI_1d |
| Regulatory T cell | GSM1095734 | 0.63969803 | SCI_1d |
| T follicular helper cell | GSM1095734 | 0.38895245 | SCI_1d |
| Type 1 T helper cell | GSM1095734 | 0.29816119 | SCI_1d |
| Type 17 T helper cell | GSM1095734 | 0.01989569 | SCI_1d |
| Type 2 T helper cell | GSM1095734 | -0.18874212 | SCI_1d |
| Activated B cell | GSM1095735 | 0.13066095 | SCI_1d |
| Activated CD4 T cell | GSM1095735 | -0.31776905 | SCI_1d |
| Activated CD8 T cell | GSM1095735 | -0.16962703 | SCI_1d |
| Activated dendritic cell | GSM1095735 | -0.28537319 | SCI_1d |
| CD56bright natural killer cell | GSM1095735 | 0.07873325 | SCI_1d |
| CD56dim natural killer cell | GSM1095735 | 0.19570373 | SCI_1d |
| Central memory CD4 T cell | GSM1095735 | 0.26608353 | SCI_1d |
| Central memory CD8 T cell | GSM1095735 | -0.08869878 | SCI_1d |
| Effector memeory CD4 T cell | GSM1095735 | 0.35076535 | SCI_1d |
| Effector memeory CD8 T cell | GSM1095735 | -0.06586485 | SCI_1d |
| Eosinophil | GSM1095735 | 0.07619214 | SCI_1d |
| Gamma delta T cell | GSM1095735 | 0.31410333 | SCI_1d |
| Immature B cell | GSM1095735 | 0.32805413 | SCI_1d |
| Immature dendritic cell | GSM1095735 | 0.2610219 | SCI_1d |
| Macrophage | GSM1095735 | 0.10315454 | SCI_1d |
| Mast cell | GSM1095735 | 0.13935322 | SCI_1d |
| MDSC | GSM1095735 | -0.03636463 | SCI_1d |
| Memory B cell | GSM1095735 | 0.19605507 | SCI_1d |
| Monocyte | GSM1095735 | 0.38144186 | SCI_1d |
| Natural killer cell | GSM1095735 | 0.18143003 | SCI_1d |
| Natural killer T cell | GSM1095735 | 0.09866845 | SCI_1d |
| Neutrophil | GSM1095735 | 0.14079143 | SCI_1d |
| Plasmacytoid dendritic cell | GSM1095735 | 0.20003135 | SCI_1d |
| Regulatory T cell | GSM1095735 | 0.1741055 | SCI_1d |
| T follicular helper cell | GSM1095735 | 0.24007267 | SCI_1d |
| Type 1 T helper cell | GSM1095735 | 0.03108838 | SCI_1d |
| Type 17 T helper cell | GSM1095735 | -0.19156697 | SCI_1d |
| Type 2 T helper cell | GSM1095735 | 0.02448726 | SCI_1d |
| Activated B cell | GSM1095736 | 0.1196081 | SCI_1d |
| Activated CD4 T cell | GSM1095736 | 0.05923799 | SCI_1d |
| Activated CD8 T cell | GSM1095736 | 0.01531077 | SCI_1d |
| Activated dendritic cell | GSM1095736 | -0.0806566 | SCI_1d |
| CD56bright natural killer cell | GSM1095736 | 0.07603764 | SCI_1d |
| CD56dim natural killer cell | GSM1095736 | 0.20318287 | SCI_1d |
| Central memory CD4 T cell | GSM1095736 | 0.23656247 | SCI_1d |
| Central memory CD8 T cell | GSM1095736 | 0.26743844 | SCI_1d |
| Effector memeory CD4 T cell | GSM1095736 | 0.35670425 | SCI_1d |
| Effector memeory CD8 T cell | GSM1095736 | -0.11867751 | SCI_1d |
| Eosinophil | GSM1095736 | 0.29576783 | SCI_1d |
| Gamma delta T cell | GSM1095736 | 0.22184879 | SCI_1d |
| Immature B cell | GSM1095736 | 0.45288537 | SCI_1d |
| Immature dendritic cell | GSM1095736 | 0.30652186 | SCI_1d |
| Macrophage | GSM1095736 | 0.21213304 | SCI_1d |
| Mast cell | GSM1095736 | 0.25510726 | SCI_1d |
| MDSC | GSM1095736 | 0.31518349 | SCI_1d |
| Memory B cell | GSM1095736 | -0.0690145 | SCI_1d |
| Monocyte | GSM1095736 | 0.49930965 | SCI_1d |
| Natural killer cell | GSM1095736 | 0.2457088 | SCI_1d |
| Natural killer T cell | GSM1095736 | 0.04696165 | SCI_1d |
| Neutrophil | GSM1095736 | -0.44703659 | SCI_1d |
| Plasmacytoid dendritic cell | GSM1095736 | 0.35463865 | SCI_1d |
| Regulatory T cell | GSM1095736 | 0.54964506 | SCI_1d |
| T follicular helper cell | GSM1095736 | 0.32738122 | SCI_1d |
| Type 1 T helper cell | GSM1095736 | -0.08385734 | SCI_1d |
| Type 17 T helper cell | GSM1095736 | 0.02310668 | SCI_1d |
| Type 2 T helper cell | GSM1095736 | -0.14403024 | SCI_1d |
| Activated B cell | GSM1095737 | 0.07694064 | SCI_3d |
| Activated CD4 T cell | GSM1095737 | 0.26608109 | SCI_3d |
| Activated CD8 T cell | GSM1095737 | 0.33374558 | SCI_3d |
| Activated dendritic cell | GSM1095737 | 0.54148385 | SCI_3d |
| CD56bright natural killer cell | GSM1095737 | -0.1822191 | SCI_3d |
| CD56dim natural killer cell | GSM1095737 | -0.20563134 | SCI_3d |
| Central memory CD4 T cell | GSM1095737 | 0.18661018 | SCI_3d |
| Central memory CD8 T cell | GSM1095737 | -0.087619 | SCI_3d |
| Effector memeory CD4 T cell | GSM1095737 | -0.43316572 | SCI_3d |
| Effector memeory CD8 T cell | GSM1095737 | 0.4355279 | SCI_3d |
| Eosinophil | GSM1095737 | 0.03766267 | SCI_3d |
| Gamma delta T cell | GSM1095737 | 0.35134318 | SCI_3d |
| Immature B cell | GSM1095737 | 0.43474807 | SCI_3d |
| Immature dendritic cell | GSM1095737 | -0.20949927 | SCI_3d |
| Macrophage | GSM1095737 | 0.12059194 | SCI_3d |
| Mast cell | GSM1095737 | 0.01718794 | SCI_3d |
| MDSC | GSM1095737 | 0.42227556 | SCI_3d |
| Memory B cell | GSM1095737 | 0.39419129 | SCI_3d |
| Monocyte | GSM1095737 | -0.14861097 | SCI_3d |
| Natural killer cell | GSM1095737 | 0.33679593 | SCI_3d |
| Natural killer T cell | GSM1095737 | 0.19481912 | SCI_3d |
| Neutrophil | GSM1095737 | 0.12474537 | SCI_3d |
| Plasmacytoid dendritic cell | GSM1095737 | -0.1155296 | SCI_3d |
| Regulatory T cell | GSM1095737 | 0.32019244 | SCI_3d |
| T follicular helper cell | GSM1095737 | -0.0582232 | SCI_3d |
| Type 1 T helper cell | GSM1095737 | 0.09495916 | SCI_3d |
| Type 17 T helper cell | GSM1095737 | 0.26530189 | SCI_3d |
| Type 2 T helper cell | GSM1095737 | -0.06387285 | SCI_3d |
| Activated B cell | GSM1095738 | 0.09519021 | SCI_3d |
| Activated CD4 T cell | GSM1095738 | 0.37991041 | SCI_3d |
| Activated CD8 T cell | GSM1095738 | 0.06725748 | SCI_3d |
| Activated dendritic cell | GSM1095738 | 0.53337838 | SCI_3d |
| CD56bright natural killer cell | GSM1095738 | -0.03624849 | SCI_3d |
| CD56dim natural killer cell | GSM1095738 | -0.04359319 | SCI_3d |
| Central memory CD4 T cell | GSM1095738 | 0.53223142 | SCI_3d |
| Central memory CD8 T cell | GSM1095738 | 0.12515529 | SCI_3d |
| Effector memeory CD4 T cell | GSM1095738 | 0.49379577 | SCI_3d |
| Effector memeory CD8 T cell | GSM1095738 | 0.17532935 | SCI_3d |
| Eosinophil | GSM1095738 | -0.23211454 | SCI_3d |
| Gamma delta T cell | GSM1095738 | 0.35600823 | SCI_3d |
| Immature B cell | GSM1095738 | 0.28721044 | SCI_3d |
| Immature dendritic cell | GSM1095738 | 0.345821 | SCI_3d |
| Macrophage | GSM1095738 | 0.29023918 | SCI_3d |
| Mast cell | GSM1095738 | -0.05559543 | SCI_3d |
| MDSC | GSM1095738 | 0.44581699 | SCI_3d |
| Memory B cell | GSM1095738 | 0.44415083 | SCI_3d |
| Monocyte | GSM1095738 | 0.44415945 | SCI_3d |
| Natural killer cell | GSM1095738 | 0.31905204 | SCI_3d |
| Natural killer T cell | GSM1095738 | 0.07963146 | SCI_3d |
| Neutrophil | GSM1095738 | -0.42152675 | SCI_3d |
| Plasmacytoid dendritic cell | GSM1095738 | 0.29090657 | SCI_3d |
| Regulatory T cell | GSM1095738 | 0.4305893 | SCI_3d |
| T follicular helper cell | GSM1095738 | 0.12280981 | SCI_3d |
| Type 1 T helper cell | GSM1095738 | 0.29201139 | SCI_3d |
| Type 17 T helper cell | GSM1095738 | -0.11983468 | SCI_3d |
| Type 2 T helper cell | GSM1095738 | 0.17253003 | SCI_3d |
| Activated B cell | GSM1095739 | 0.37340943 | SCI_3d |
| Activated CD4 T cell | GSM1095739 | 0.42009054 | SCI_3d |
| Activated CD8 T cell | GSM1095739 | 0.39028643 | SCI_3d |
| Activated dendritic cell | GSM1095739 | 0.04174268 | SCI_3d |
| CD56bright natural killer cell | GSM1095739 | 0.18465032 | SCI_3d |
| CD56dim natural killer cell | GSM1095739 | -0.03983978 | SCI_3d |
| Central memory CD4 T cell | GSM1095739 | -0.15796769 | SCI_3d |
| Central memory CD8 T cell | GSM1095739 | 0.01019868 | SCI_3d |
| Effector memeory CD4 T cell | GSM1095739 | -0.31289534 | SCI_3d |
| Effector memeory CD8 T cell | GSM1095739 | 0.10402853 | SCI_3d |
| Eosinophil | GSM1095739 | 0.16014565 | SCI_3d |
| Gamma delta T cell | GSM1095739 | 0.23897154 | SCI_3d |
| Immature B cell | GSM1095739 | 0.45191584 | SCI_3d |
| Immature dendritic cell | GSM1095739 | -0.20948367 | SCI_3d |
| Macrophage | GSM1095739 | -0.09910064 | SCI_3d |
| Mast cell | GSM1095739 | 0.58093252 | SCI_3d |
| MDSC | GSM1095739 | 0.19064956 | SCI_3d |
| Memory B cell | GSM1095739 | 0.19262415 | SCI_3d |
| Monocyte | GSM1095739 | -0.00555676 | SCI_3d |
| Natural killer cell | GSM1095739 | 0.1121178 | SCI_3d |
| Natural killer T cell | GSM1095739 | 0.38217252 | SCI_3d |
| Neutrophil | GSM1095739 | 0.47961026 | SCI_3d |
| Plasmacytoid dendritic cell | GSM1095739 | -0.15342874 | SCI_3d |
| Regulatory T cell | GSM1095739 | -0.19266419 | SCI_3d |
| T follicular helper cell | GSM1095739 | 0.00742374 | SCI_3d |
| Type 1 T helper cell | GSM1095739 | 0.20111901 | SCI_3d |
| Type 17 T helper cell | GSM1095739 | 0.40605665 | SCI_3d |
| Type 2 T helper cell | GSM1095739 | -0.0655967 | SCI_3d |
| Activated B cell | GSM1095740 | 0.13480765 | SCI_3d |
| Activated CD4 T cell | GSM1095740 | 0.55358584 | SCI_3d |
| Activated CD8 T cell | GSM1095740 | 0.12337887 | SCI_3d |
| Activated dendritic cell | GSM1095740 | 0.33547744 | SCI_3d |
| CD56bright natural killer cell | GSM1095740 | -0.26127929 | SCI_3d |
| CD56dim natural killer cell | GSM1095740 | -0.11634044 | SCI_3d |
| Central memory CD4 T cell | GSM1095740 | 0.48117138 | SCI_3d |
| Central memory CD8 T cell | GSM1095740 | 0.2890261 | SCI_3d |
| Effector memeory CD4 T cell | GSM1095740 | 0.17405507 | SCI_3d |
| Effector memeory CD8 T cell | GSM1095740 | 0.43375472 | SCI_3d |
| Eosinophil | GSM1095740 | 0.0409136 | SCI_3d |
| Gamma delta T cell | GSM1095740 | 0.42863333 | SCI_3d |
| Immature B cell | GSM1095740 | 0.47976547 | SCI_3d |
| Immature dendritic cell | GSM1095740 | 0.36043821 | SCI_3d |
| Macrophage | GSM1095740 | 0.1129425 | SCI_3d |
| Mast cell | GSM1095740 | 0.36497111 | SCI_3d |
| MDSC | GSM1095740 | 0.60985809 | SCI_3d |
| Memory B cell | GSM1095740 | 0.27674871 | SCI_3d |
| Monocyte | GSM1095740 | 0.41454664 | SCI_3d |
| Natural killer cell | GSM1095740 | 0.48817485 | SCI_3d |
| Natural killer T cell | GSM1095740 | 0.27337464 | SCI_3d |
| Neutrophil | GSM1095740 | -0.32908032 | SCI_3d |
| Plasmacytoid dendritic cell | GSM1095740 | 0.36583632 | SCI_3d |
| Regulatory T cell | GSM1095740 | 0.50891225 | SCI_3d |
| T follicular helper cell | GSM1095740 | 0.15321875 | SCI_3d |
| Type 1 T helper cell | GSM1095740 | 0.33031788 | SCI_3d |
| Type 17 T helper cell | GSM1095740 | -0.02405231 | SCI_3d |
| Type 2 T helper cell | GSM1095740 | 0.0778593 | SCI_3d |
| Activated B cell | GSM1095741 | -0.16445868 | SCI_7d |
| Activated CD4 T cell | GSM1095741 | -0.18047164 | SCI_7d |
| Activated CD8 T cell | GSM1095741 | -0.28040691 | SCI_7d |
| Activated dendritic cell | GSM1095741 | -0.32887837 | SCI_7d |
| CD56bright natural killer cell | GSM1095741 | -0.26854697 | SCI_7d |
| CD56dim natural killer cell | GSM1095741 | 0.12424165 | SCI_7d |
| Central memory CD4 T cell | GSM1095741 | -0.15591018 | SCI_7d |
| Central memory CD8 T cell | GSM1095741 | -0.35656116 | SCI_7d |
| Effector memeory CD4 T cell | GSM1095741 | -0.17309093 | SCI_7d |
| Effector memeory CD8 T cell | GSM1095741 | -0.02830571 | SCI_7d |
| Eosinophil | GSM1095741 | -0.44405002 | SCI_7d |
| Gamma delta T cell | GSM1095741 | -0.15272132 | SCI_7d |
| Immature B cell | GSM1095741 | -0.57639757 | SCI_7d |
| Immature dendritic cell | GSM1095741 | -0.35664824 | SCI_7d |
| Macrophage | GSM1095741 | -0.21508645 | SCI_7d |
| Mast cell | GSM1095741 | -0.28957407 | SCI_7d |
| MDSC | GSM1095741 | -0.26875451 | SCI_7d |
| Memory B cell | GSM1095741 | 0.00235868 | SCI_7d |
| Monocyte | GSM1095741 | -0.51263023 | SCI_7d |
| Natural killer cell | GSM1095741 | -0.12884764 | SCI_7d |
| Natural killer T cell | GSM1095741 | -0.23105807 | SCI_7d |
| Neutrophil | GSM1095741 | 0.11906087 | SCI_7d |
| Plasmacytoid dendritic cell | GSM1095741 | -0.20567757 | SCI_7d |
| Regulatory T cell | GSM1095741 | -0.47841684 | SCI_7d |
| T follicular helper cell | GSM1095741 | -0.20260044 | SCI_7d |
| Type 1 T helper cell | GSM1095741 | -0.17997185 | SCI_7d |
| Type 17 T helper cell | GSM1095741 | 0.1433708 | SCI_7d |
| Type 2 T helper cell | GSM1095741 | 0.244188 | SCI_7d |
| Activated B cell | GSM1095742 | -0.35605958 | SCI_7d |
| Activated CD4 T cell | GSM1095742 | 0.33730688 | SCI_7d |
| Activated CD8 T cell | GSM1095742 | -0.16154595 | SCI_7d |
| Activated dendritic cell | GSM1095742 | -0.07448019 | SCI_7d |
| CD56bright natural killer cell | GSM1095742 | 0.14440629 | SCI_7d |
| CD56dim natural killer cell | GSM1095742 | -0.00037998 | SCI_7d |
| Central memory CD4 T cell | GSM1095742 | 0.37593034 | SCI_7d |
| Central memory CD8 T cell | GSM1095742 | -0.04238421 | SCI_7d |
| Effector memeory CD4 T cell | GSM1095742 | 0.34112536 | SCI_7d |
| Effector memeory CD8 T cell | GSM1095742 | -0.33253356 | SCI_7d |
| Eosinophil | GSM1095742 | 0.2091609 | SCI_7d |
| Gamma delta T cell | GSM1095742 | 0.11650031 | SCI_7d |
| Immature B cell | GSM1095742 | -0.28249934 | SCI_7d |
| Immature dendritic cell | GSM1095742 | -0.04446086 | SCI_7d |
| Macrophage | GSM1095742 | -0.34174926 | SCI_7d |
| Mast cell | GSM1095742 | -0.52325154 | SCI_7d |
| MDSC | GSM1095742 | 0.27235092 | SCI_7d |
| Memory B cell | GSM1095742 | 0.22385132 | SCI_7d |
| Monocyte | GSM1095742 | -0.31478755 | SCI_7d |
| Natural killer cell | GSM1095742 | -0.07571005 | SCI_7d |
| Natural killer T cell | GSM1095742 | -0.19311966 | SCI_7d |
| Neutrophil | GSM1095742 | 0.15489881 | SCI_7d |
| Plasmacytoid dendritic cell | GSM1095742 | 0.10202515 | SCI_7d |
| Regulatory T cell | GSM1095742 | 0.11921283 | SCI_7d |
| T follicular helper cell | GSM1095742 | -0.19910287 | SCI_7d |
| Type 1 T helper cell | GSM1095742 | -0.19456243 | SCI_7d |
| Type 17 T helper cell | GSM1095742 | -0.44242142 | SCI_7d |
| Type 2 T helper cell | GSM1095742 | 0.1794443 | SCI_7d |
| Activated B cell | GSM1095743 | -0.32098922 | SCI_7d |
| Activated CD4 T cell | GSM1095743 | 0.15098721 | SCI_7d |
| Activated CD8 T cell | GSM1095743 | -0.19472682 | SCI_7d |
| Activated dendritic cell | GSM1095743 | 0.04527842 | SCI_7d |
| CD56bright natural killer cell | GSM1095743 | -0.01865852 | SCI_7d |
| CD56dim natural killer cell | GSM1095743 | -0.38336976 | SCI_7d |
| Central memory CD4 T cell | GSM1095743 | 0.40631284 | SCI_7d |
| Central memory CD8 T cell | GSM1095743 | 0.47658127 | SCI_7d |
| Effector memeory CD4 T cell | GSM1095743 | 0.13027483 | SCI_7d |
| Effector memeory CD8 T cell | GSM1095743 | -0.30849521 | SCI_7d |
| Eosinophil | GSM1095743 | -0.23550913 | SCI_7d |
| Gamma delta T cell | GSM1095743 | 0.14425582 | SCI_7d |
| Immature B cell | GSM1095743 | -0.39875081 | SCI_7d |
| Immature dendritic cell | GSM1095743 | 0.1289926 | SCI_7d |
| Macrophage | GSM1095743 | 0.07060603 | SCI_7d |
| Mast cell | GSM1095743 | -0.52646027 | SCI_7d |
| MDSC | GSM1095743 | 0.19202223 | SCI_7d |
| Memory B cell | GSM1095743 | -0.00755783 | SCI_7d |
| Monocyte | GSM1095743 | 0.12389207 | SCI_7d |
| Natural killer cell | GSM1095743 | 0.08784996 | SCI_7d |
| Natural killer T cell | GSM1095743 | -0.20808711 | SCI_7d |
| Neutrophil | GSM1095743 | -0.30282068 | SCI_7d |
| Plasmacytoid dendritic cell | GSM1095743 | 0.08977962 | SCI_7d |
| Regulatory T cell | GSM1095743 | -0.18146967 | SCI_7d |
| T follicular helper cell | GSM1095743 | 0.09959216 | SCI_7d |
| Type 1 T helper cell | GSM1095743 | -0.20893984 | SCI_7d |
| Type 17 T helper cell | GSM1095743 | -0.52390019 | SCI_7d |
| Type 2 T helper cell | GSM1095743 | 0.19183564 | SCI_7d |
| Activated B cell | GSM1095744 | -0.0341308 | SCI_7d |
| Activated CD4 T cell | GSM1095744 | 0.13773057 | SCI_7d |
| Activated CD8 T cell | GSM1095744 | 0.08099087 | SCI_7d |
| Activated dendritic cell | GSM1095744 | -0.30773434 | SCI_7d |
| CD56bright natural killer cell | GSM1095744 | -0.02684045 | SCI_7d |
| CD56dim natural killer cell | GSM1095744 | 0.07216698 | SCI_7d |
| Central memory CD4 T cell | GSM1095744 | -0.19412074 | SCI_7d |
| Central memory CD8 T cell | GSM1095744 | -0.32719955 | SCI_7d |
| Effector memeory CD4 T cell | GSM1095744 | -0.26425302 | SCI_7d |
| Effector memeory CD8 T cell | GSM1095744 | -0.24430152 | SCI_7d |
| Eosinophil | GSM1095744 | 0.09488171 | SCI_7d |
| Gamma delta T cell | GSM1095744 | -0.0324285 | SCI_7d |
| Immature B cell | GSM1095744 | -0.52194995 | SCI_7d |
| Immature dendritic cell | GSM1095744 | -0.45829316 | SCI_7d |
| Macrophage | GSM1095744 | -0.04079823 | SCI_7d |
| Mast cell | GSM1095744 | -0.26379307 | SCI_7d |
| MDSC | GSM1095744 | -0.01114482 | SCI_7d |
| Memory B cell | GSM1095744 | -0.10661477 | SCI_7d |
| Monocyte | GSM1095744 | -0.40531481 | SCI_7d |
| Natural killer cell | GSM1095744 | -0.26287066 | SCI_7d |
| Natural killer T cell | GSM1095744 | -0.16679047 | SCI_7d |
| Neutrophil | GSM1095744 | -0.24338099 | SCI_7d |
| Plasmacytoid dendritic cell | GSM1095744 | -0.3867077 | SCI_7d |
| Regulatory T cell | GSM1095744 | -0.31348328 | SCI_7d |
| T follicular helper cell | GSM1095744 | -0.18487454 | SCI_7d |
| Type 1 T helper cell | GSM1095744 | -0.28897733 | SCI_7d |
| Type 17 T helper cell | GSM1095744 | -0.15931216 | SCI_7d |
| Type 2 T helper cell | GSM1095744 | 0.12793026 | SCI_7d |

**Supplementary Table S15. Group-wise mean ± SEM of ssGSEA scores**

| **Immune_Cell** | **SCI_1d** | **SCI_3d** | **SCI_7d** | **Sham** |
| --- | --- | --- | --- | --- |
| Activated B cell | 0.0426 ± 0.0926 | 0.1701 ± 0.0688 | -0.2189 ± 0.0744 | 0.0528 ± 0.0171 |
| Activated CD4 T cell | 0.0767 ± 0.1590 | 0.4049 ± 0.0593 | 0.1114 ± 0.1074 | -0.4358 ± 0.0628 |
| Activated CD8 T cell | 0.0120 ± 0.0992 | 0.2287 ± 0.0787 | -0.1389 ± 0.0775 | -0.1573 ± 0.0847 |
| Activated dendritic cell | 0.1619 ± 0.2070 | 0.3630 ± 0.1172 | -0.1665 ± 0.0911 | -0.4386 ± 0.0413 |
| CD56bright natural killer cell | 0.0961 ± 0.0112 | -0.0738 ± 0.0979 | -0.0424 ± 0.0851 | 0.0297 ± 0.0152 |
| CD56dim natural killer cell | 0.1742 ± 0.0831 | -0.1014 ± 0.0390 | -0.0468 ± 0.1151 | -0.0213 ± 0.0791 |
| Central memory CD4 T cell | 0.3691 ± 0.0716 | 0.2605 ± 0.1589 | 0.1081 ± 0.1637 | -0.6051 ± 0.0413 |
| Central memory CD8 T cell | 0.2444 ± 0.1317 | 0.0842 ± 0.0809 | -0.0624 ± 0.1931 | -0.3014 ± 0.0710 |
| Effector memeory CD4 T cell | 0.3595 ± 0.0704 | -0.0196 ± 0.2157 | 0.0085 ± 0.1393 | -0.2398 ± 0.0709 |
| Effector memeory CD8 T cell | 0.1109 ± 0.1180 | 0.2872 ± 0.0864 | -0.2284 ± 0.0693 | -0.0842 ± 0.1380 |
| Eosinophil | 0.0744 ± 0.0894 | 0.0017 ± 0.0830 | -0.0939 ± 0.1500 | 0.1747 ± 0.1093 |
| Gamma delta T cell | 0.3057 ± 0.0547 | 0.3437 ± 0.0391 | 0.0189 ± 0.0691 | -0.5044 ± 0.0279 |
| Immature B cell | 0.5336 ± 0.0867 | 0.4134 ± 0.0431 | -0.4449 ± 0.0657 | -0.5049 ± 0.0469 |
| Immature dendritic cell | 0.2796 ± 0.0315 | 0.0718 ± 0.1624 | -0.1826 ± 0.1362 | -0.1086 ± 0.1028 |
| MDSC | 0.2598 ± 0.1001 | 0.4172 ± 0.0863 | 0.0461 ± 0.1207 | -0.5997 ± 0.0440 |
| Macrophage | 0.1844 ± 0.0366 | 0.1062 ± 0.0797 | -0.1318 ± 0.0914 | -0.1858 ± 0.0883 |
| Mast cell | 0.1261 ± 0.1016 | 0.2269 ± 0.1495 | -0.4008 ± 0.0718 | -0.0092 ± 0.0475 |
| Memory B cell | 0.1076 ± 0.0604 | 0.3269 ± 0.0569 | 0.0280 ± 0.0698 | -0.4069 ± 0.0695 |
| Monocyte | 0.4547 ± 0.0390 | 0.1761 ± 0.1492 | -0.2772 ± 0.1397 | -0.2251 ± 0.1028 |
| Natural killer T cell | 0.1538 ± 0.0509 | 0.2325 ± 0.0638 | -0.1998 ± 0.0135 | -0.1827 ± 0.0624 |
| Natural killer cell | 0.2415 ± 0.0415 | 0.3140 ± 0.0773 | -0.0949 ± 0.0725 | -0.3740 ± 0.0411 |
| Neutrophil | 0.0280 ± 0.1731 | -0.0366 ± 0.2094 | -0.0681 ± 0.1192 | 0.1253 ± 0.1658 |
| Plasmacytoid dendritic cell | 0.2725 ± 0.0325 | 0.0969 ± 0.1347 | -0.1001 ± 0.1191 | -0.2312 ± 0.0720 |
| Regulatory T cell | 0.4919 ± 0.1076 | 0.2668 ± 0.1580 | -0.2135 ± 0.1265 | -0.5506 ± 0.0458 |
| T follicular helper cell | 0.3670 ± 0.0571 | 0.0563 ± 0.0494 | -0.1217 ± 0.0739 | -0.2079 ± 0.0712 |
| Type 1 T helper cell | 0.1062 ± 0.0836 | 0.2296 ± 0.0524 | -0.2181 ± 0.0244 | -0.2075 ± 0.0194 |
| Type 17 T helper cell | -0.1024 ± 0.0729 | 0.1319 ± 0.1227 | -0.2456 ± 0.1514 | 0.1563 ± 0.1123 |
| Type 2 T helper cell | -0.0813 ± 0.0507 | 0.0302 ± 0.0581 | 0.1858 ± 0.0239 | -0.0256 ± 0.0808 |

**Supplementary Table S15. Dunn's post-hoc pairwise comparisons (adjusted P values)**

| **Immune_Cell** | **group1** | **group2** | **p.adj** | **p.adj.signif** |
| --- | --- | --- | --- | --- |
| Memory B cell | Sham | SCI_1 Day | 0.00381277 | ** |
| Activated CD4 T cell | Sham | SCI_1 Day | 0.00651041 | ** |
| Regulatory T cell | Sham | SCI_7 Day | 0.00651041 | ** |
| Gamma delta T cell | Sham | SCI_1 Day | 0.00844157 | ** |
| MDSC | Sham | SCI_1 Day | 0.00844157 | ** |
| Natural killer cell | Sham | SCI_1 Day | 0.01088926 | * |
| T follicular helper cell | Sham | SCI_7 Day | 0.01088926 | * |
| Activated dendritic cell | Sham | SCI_1 Day | 0.01397458 | * |
| Natural killer cell | Sham | SCI_7 Day | 0.0143211 | * |
| Gamma delta T cell | Sham | SCI_7 Day | 0.0180067 | * |
| T follicular helper cell | SCI_3 Day | SCI_7 Day | 0.02252611 | * |
| Type 1 T helper cell | Sham | SCI_1 Day | 0.02803751 | * |
| Type 1 T helper cell | SCI_1 Day | SCI_3 Day | 0.02803751 | * |
| Mast cell | SCI_1 Day | SCI_3 Day | 0.02864221 | * |
| Activated B cell | SCI_1 Day | SCI_3 Day | 0.02864221 | * |
| Central memory CD4 T cell | Sham | SCI_7 Day | 0.02864221 | * |
| Type 2 T helper cell | SCI_3 Day | SCI_7 Day | 0.02864221 | * |
| Natural killer T cell | Sham | SCI_1 Day | 0.02864221 | * |
| Immature B cell | Sham | SCI_7 Day | 0.02864221 | * |
| Activated dendritic cell | Sham | SCI_7 Day | 0.03472171 | * |
| Mast cell | SCI_3 Day | SCI_7 Day | 0.03472171 | * |
| Immature B cell | SCI_3 Day | SCI_7 Day | 0.03472171 | * |
| Natural killer T cell | Sham | SCI_7 Day | 0.03496935 | * |
| Natural killer T cell | SCI_1 Day | SCI_3 Day | 0.03496935 | * |
| Central memory CD4 T cell | Sham | SCI_1 Day | 0.0427837 | * |
| Monocyte | SCI_3 Day | SCI_7 Day | 0.04505221 | * |
| Immature B cell | Sham | SCI_1 Day | 0.05178344 | ns |
| Monocyte | Sham | SCI_7 Day | 0.05245402 | ns |
| Regulatory T cell | Sham | SCI_1 Day | 0.05245402 | ns |
| MDSC | Sham | SCI_7 Day | 0.05245402 | ns |
| Effector memeory CD8 T cell | SCI_1 Day | SCI_3 Day | 0.05607503 | ns |
| Type 1 T helper cell | Sham | SCI_7 Day | 0.06254768 | ns |
| Macrophage | Sham | SCI_7 Day | 0.06398978 | ns |
| Activated B cell | SCI_3 Day | SCI_7 Day | 0.06398978 | ns |
| Macrophage | SCI_3 Day | SCI_7 Day | 0.06398978 | ns |
| Type 1 T helper cell | SCI_3 Day | SCI_7 Day | 0.06743675 | ns |
| Plasmacytoid dendritic cell | Sham | SCI_7 Day | 0.06944343 | ns |
| Effector memeory CD4 T cell | Sham | SCI_7 Day | 0.06944343 | ns |
| Activated CD8 T cell | Sham | SCI_1 Day | 0.06944343 | ns |
| Central memory CD8 T cell | Sham | SCI_7 Day | 0.06944343 | ns |
| Regulatory T cell | SCI_3 Day | SCI_7 Day | 0.07517776 | ns |
| Immature B cell | SCI_1 Day | SCI_3 Day | 0.08026397 | ns |
| Natural killer T cell | SCI_3 Day | SCI_7 Day | 0.08026397 | ns |
| Gamma delta T cell | SCI_1 Day | SCI_3 Day | 0.08991567 | ns |
| Macrophage | Sham | SCI_1 Day | 0.08991567 | ns |
| Type 2 T helper cell | Sham | SCI_3 Day | 0.09382151 | ns |
| Central memory CD8 T cell | Sham | SCI_1 Day | 0.09382151 | ns |
| Activated CD8 T cell | SCI_1 Day | SCI_3 Day | 0.09382151 | ns |
| Plasmacytoid dendritic cell | SCI_3 Day | SCI_7 Day | 0.10701862 | ns |
| Plasmacytoid dendritic cell | Sham | SCI_1 Day | 0.10701862 | ns |
| Natural killer cell | SCI_1 Day | SCI_3 Day | 0.10701862 | ns |
| Macrophage | SCI_1 Day | SCI_3 Day | 0.1120589 | ns |
| Type 2 T helper cell | SCI_1 Day | SCI_3 Day | 0.12675706 | ns |
| Activated CD4 T cell | Sham | SCI_7 Day | 0.12675706 | ns |
| Activated CD4 T cell | Sham | SCI_3 Day | 0.12675706 | ns |
| Central memory CD4 T cell | Sham | SCI_3 Day | 0.12675706 | ns |
| Memory B cell | Sham | SCI_7 Day | 0.13145326 | ns |
| Memory B cell | Sham | SCI_3 Day | 0.13145326 | ns |
| Memory B cell | SCI_1 Day | SCI_3 Day | 0.13145326 | ns |
| Mast cell | Sham | SCI_3 Day | 0.14941187 | ns |
| Monocyte | SCI_1 Day | SCI_3 Day | 0.14941187 | ns |
| T follicular helper cell | Sham | SCI_1 Day | 0.14941187 | ns |
| Natural killer cell | SCI_3 Day | SCI_7 Day | 0.1534691 | ns |
| Immature dendritic cell | SCI_3 Day | SCI_7 Day | 0.15535033 | ns |
| Immature dendritic cell | Sham | SCI_7 Day | 0.16052793 | ns |
| Gamma delta T cell | SCI_3 Day | SCI_7 Day | 0.17832338 | ns |
| MDSC | SCI_1 Day | SCI_3 Day | 0.17832338 | ns |
| MDSC | Sham | SCI_3 Day | 0.17832338 | ns |
| CD56dim natural killer cell | SCI_1 Day | SCI_7 Day | 0.18764303 | ns |
| Memory B cell | SCI_1 Day | SCI_7 Day | 0.1899076 | ns |
| Effector memeory CD4 T cell | SCI_1 Day | SCI_7 Day | 0.20462546 | ns |
| Effector memeory CD8 T cell | Sham | SCI_1 Day | 0.20462546 | ns |
| Effector memeory CD4 T cell | SCI_3 Day | SCI_7 Day | 0.20462546 | ns |
| Effector memeory CD8 T cell | SCI_3 Day | SCI_7 Day | 0.20462546 | ns |
| Activated dendritic cell | SCI_1 Day | SCI_3 Day | 0.20622781 | ns |
| Monocyte | Sham | SCI_1 Day | 0.20622781 | ns |
| Activated dendritic cell | Sham | SCI_3 Day | 0.20622781 | ns |
| T follicular helper cell | SCI_1 Day | SCI_3 Day | 0.21758502 | ns |
| T follicular helper cell | SCI_1 Day | SCI_7 Day | 0.21758502 | ns |
| Activated CD4 T cell | SCI_1 Day | SCI_7 Day | 0.2373845 | ns |
| Activated B cell | Sham | SCI_3 Day | 0.2373845 | ns |
| Activated B cell | Sham | SCI_1 Day | 0.2373845 | ns |
| Type 17 T helper cell | SCI_1 Day | SCI_3 Day | 0.2377645 | ns |
| Type 17 T helper cell | Sham | SCI_3 Day | 0.2377645 | ns |
| Type 17 T helper cell | Sham | SCI_7 Day | 0.2377645 | ns |
| Activated CD4 T cell | SCI_1 Day | SCI_3 Day | 0.24814942 | ns |
| CD56bright natural killer cell | SCI_1 Day | SCI_7 Day | 0.26290652 | ns |
| CD56bright natural killer cell | SCI_3 Day | SCI_7 Day | 0.26290652 | ns |
| Plasmacytoid dendritic cell | SCI_1 Day | SCI_3 Day | 0.27198127 | ns |
| Natural killer cell | Sham | SCI_3 Day | 0.2817164 | ns |
| Regulatory T cell | SCI_1 Day | SCI_3 Day | 0.2817164 | ns |
| Regulatory T cell | Sham | SCI_3 Day | 0.2817164 | ns |
| Gamma delta T cell | Sham | SCI_3 Day | 0.2817164 | ns |
| Central memory CD8 T cell | SCI_3 Day | SCI_7 Day | 0.31018677 | ns |
| Activated CD8 T cell | SCI_1 Day | SCI_7 Day | 0.31018677 | ns |
| Activated CD8 T cell | Sham | SCI_7 Day | 0.31018677 | ns |
| Type 17 T helper cell | SCI_1 Day | SCI_7 Day | 0.31018677 | ns |
| Central memory CD8 T cell | Sham | SCI_3 Day | 0.31018677 | ns |
| CD56dim natural killer cell | Sham | SCI_7 Day | 0.35664675 | ns |
| Immature dendritic cell | SCI_1 Day | SCI_3 Day | 0.3626417 | ns |
| CD56dim natural killer cell | SCI_3 Day | SCI_7 Day | 0.3626417 | ns |
| Regulatory T cell | SCI_1 Day | SCI_7 Day | 0.37285794 | ns |
| Activated dendritic cell | SCI_3 Day | SCI_7 Day | 0.40121709 | ns |
| Effector memeory CD8 T cell | SCI_1 Day | SCI_7 Day | 0.41400174 | ns |
| Effector memeory CD8 T cell | Sham | SCI_7 Day | 0.41400174 | ns |
| MDSC | SCI_1 Day | SCI_7 Day | 0.41400174 | ns |
| Effector memeory CD8 T cell | Sham | SCI_3 Day | 0.41400174 | ns |
| MDSC | SCI_3 Day | SCI_7 Day | 0.41400174 | ns |
| Activated CD8 T cell | SCI_3 Day | SCI_7 Day | 0.44742952 | ns |
| Immature dendritic cell | Sham | SCI_1 Day | 0.44742952 | ns |
| Monocyte | SCI_1 Day | SCI_7 Day | 0.44742952 | ns |
| Activated B cell | Sham | SCI_7 Day | 0.44742952 | ns |
| Central memory CD8 T cell | SCI_1 Day | SCI_3 Day | 0.44742952 | ns |
| Immature dendritic cell | SCI_1 Day | SCI_7 Day | 0.44742952 | ns |
| Mast cell | Sham | SCI_1 Day | 0.44775038 | ns |
| CD56bright natural killer cell | Sham | SCI_7 Day | 0.46952733 | ns |
| Type 2 T helper cell | SCI_1 Day | SCI_7 Day | 0.50152137 | ns |
| Central memory CD4 T cell | SCI_3 Day | SCI_7 Day | 0.50152137 | ns |
| Mast cell | Sham | SCI_7 Day | 0.54926092 | ns |
| Effector memeory CD4 T cell | Sham | SCI_1 Day | 0.54926092 | ns |
| Effector memeory CD4 T cell | Sham | SCI_3 Day | 0.54926092 | ns |
| Plasmacytoid dendritic cell | SCI_1 Day | SCI_7 Day | 0.5524529 | ns |
| Plasmacytoid dendritic cell | Sham | SCI_3 Day | 0.5524529 | ns |
| Activated B cell | SCI_1 Day | SCI_7 Day | 0.60318384 | ns |
| Activated dendritic cell | SCI_1 Day | SCI_7 Day | 0.60318384 | ns |
| Type 2 T helper cell | Sham | SCI_7 Day | 0.60469256 | ns |
| CD56dim natural killer cell | SCI_1 Day | SCI_3 Day | 0.62100261 | ns |
| Natural killer T cell | SCI_1 Day | SCI_7 Day | 0.65591049 | ns |
| T follicular helper cell | Sham | SCI_3 Day | 0.65591049 | ns |
| Natural killer T cell | Sham | SCI_3 Day | 0.65591049 | ns |
| Gamma delta T cell | SCI_1 Day | SCI_7 Day | 0.65591049 | ns |
| Eosinophil | Sham | SCI_7 Day | 0.66294349 | ns |
| Eosinophil | Sham | SCI_1 Day | 0.66294349 | ns |
| Eosinophil | Sham | SCI_3 Day | 0.66294349 | ns |
| Immature B cell | SCI_1 Day | SCI_7 Day | 0.66294349 | ns |
| Eosinophil | SCI_1 Day | SCI_7 Day | 0.66294349 | ns |
| CD56dim natural killer cell | Sham | SCI_1 Day | 0.66294349 | ns |
| Type 1 T helper cell | SCI_1 Day | SCI_7 Day | 0.66294349 | ns |
| Eosinophil | SCI_3 Day | SCI_7 Day | 0.66294349 | ns |
| Central memory CD4 T cell | SCI_1 Day | SCI_3 Day | 0.66294349 | ns |
| Central memory CD8 T cell | SCI_1 Day | SCI_7 Day | 0.71041013 | ns |
| Central memory CD4 T cell | SCI_1 Day | SCI_7 Day | 0.71041013 | ns |
| Activated CD8 T cell | Sham | SCI_3 Day | 0.71041013 | ns |
| CD56bright natural killer cell | Sham | SCI_1 Day | 0.72382061 | ns |
| CD56bright natural killer cell | Sham | SCI_3 Day | 0.72382061 | ns |
| Macrophage | SCI_1 Day | SCI_7 Day | 0.72382061 | ns |
| Monocyte | Sham | SCI_3 Day | 0.76643272 | ns |
| Memory B cell | SCI_3 Day | SCI_7 Day | 0.76643272 | ns |
| Mast cell | SCI_1 Day | SCI_7 Day | 0.76643272 | ns |
| Type 17 T helper cell | SCI_3 Day | SCI_7 Day | 0.76643272 | ns |
| Immature B cell | Sham | SCI_3 Day | 0.76643272 | ns |
| Immature dendritic cell | Sham | SCI_3 Day | 0.76643272 | ns |
| Type 17 T helper cell | Sham | SCI_1 Day | 0.76643272 | ns |
| Type 2 T helper cell | Sham | SCI_1 Day | 0.76643272 | ns |
| Natural killer cell | SCI_1 Day | SCI_7 Day | 0.76643272 | ns |
| CD56dim natural killer cell | Sham | SCI_3 Day | 0.82370425 | ns |
| Macrophage | Sham | SCI_3 Day | 0.82370425 | ns |
| Activated CD4 T cell | SCI_3 Day | SCI_7 Day | 0.88193072 | ns |
| Effector memeory CD4 T cell | SCI_1 Day | SCI_3 Day | 0.88193072 | ns |
| CD56bright natural killer cell | SCI_1 Day | SCI_3 Day | 0.88193072 | ns |
| Type 1 T helper cell | Sham | SCI_3 Day | 0.88193072 | ns |
| Neutrophil | Sham | SCI_7 Day | 0.94080266 | ns |
| Neutrophil | Sham | SCI_1 Day | 0.94080266 | ns |
| Neutrophil | Sham | SCI_3 Day | 0.94080266 | ns |
| Neutrophil | SCI_1 Day | SCI_7 Day | 0.94080266 | ns |
| Neutrophil | SCI_3 Day | SCI_7 Day | 0.94080266 | ns |
| Neutrophil | SCI_1 Day | SCI_3 Day | 0.94080266 | ns |
| Eosinophil | SCI_1 Day | SCI_3 Day | 1 | ns |
