# Supplementary material for: Ferroptosis-associated myeloid cell heterogeneity and inflammatory amplification following spinal cord injury
Source: Front Immunol. 2026 Apr 22;17:1831161. doi: 10.3389/fimmu.2026.1831161 (PMC13143767; doi:10.3389/fimmu.2026.1831161)

iNOS


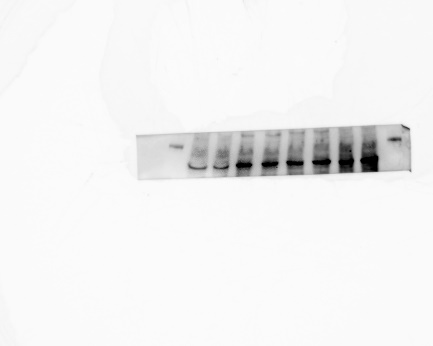

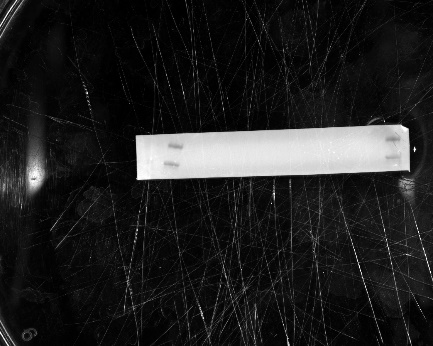

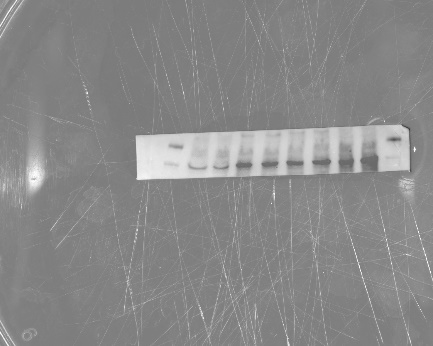


β-actin


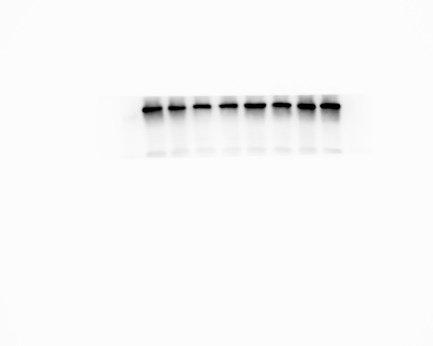

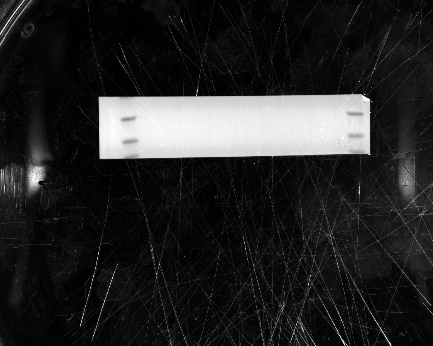

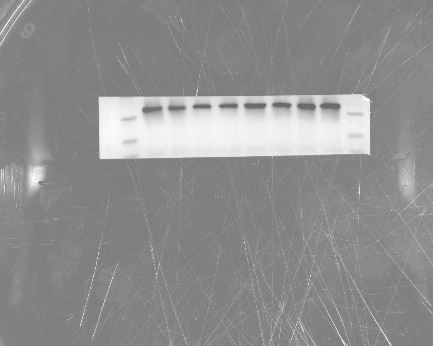


merge

130kDa

180kDa

100kDa

70kDa

55kDa

40kDa

15kDa

25kDa

35kDa


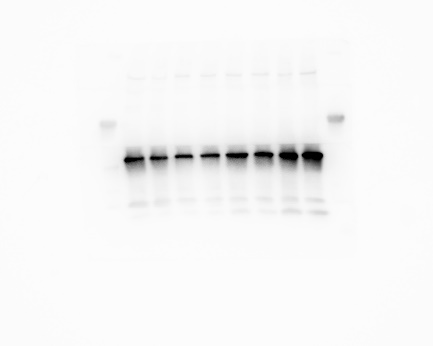

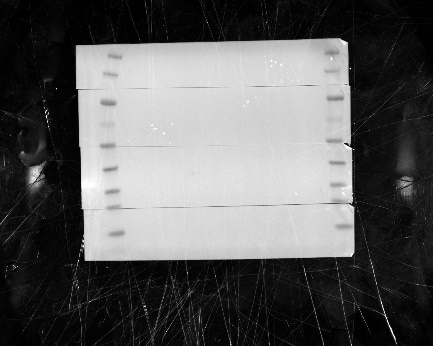

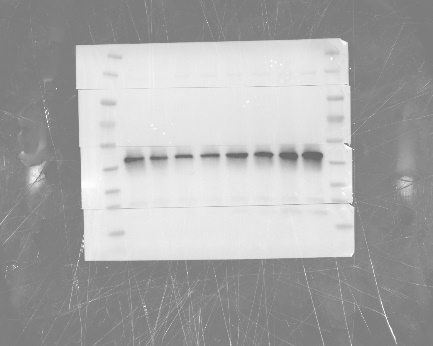


ARG-1


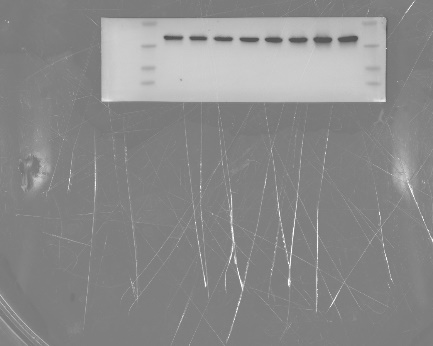

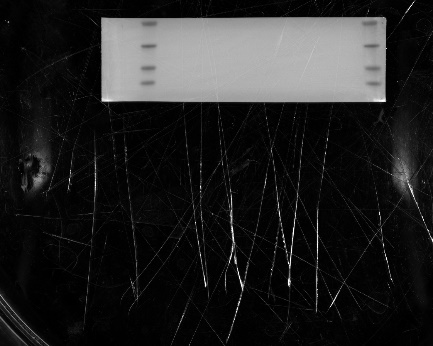

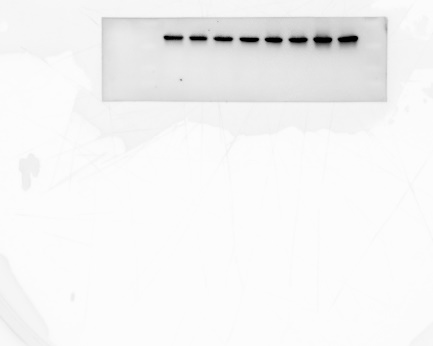


Vinculin


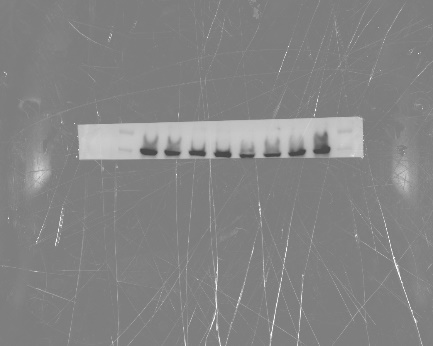

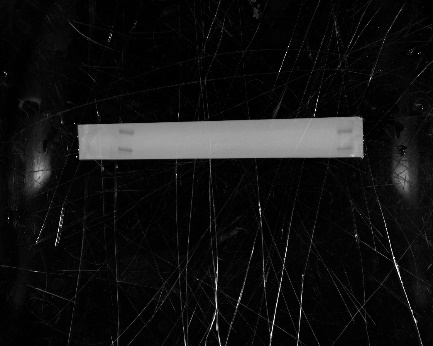

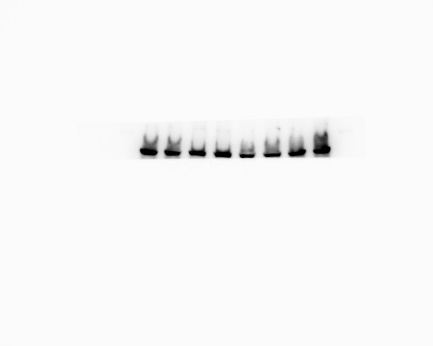


Merge

130kDa

15kDa

25kDa

35kDa

55kDa

70kDa

100kDa

180kDa


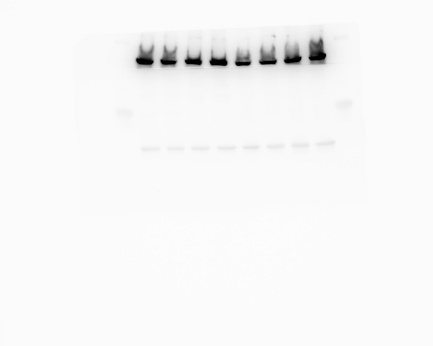

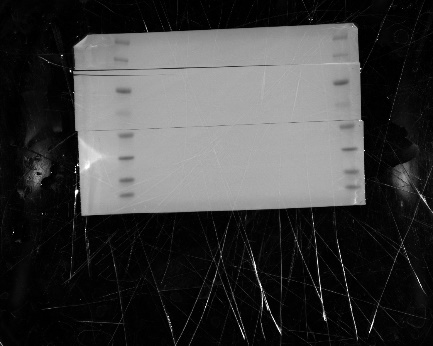

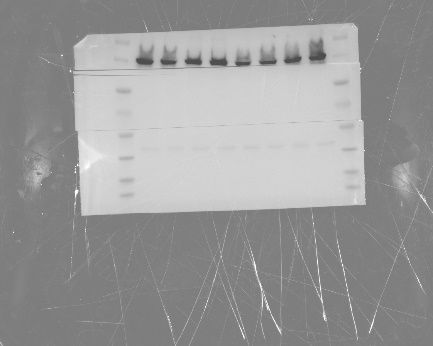


CD206


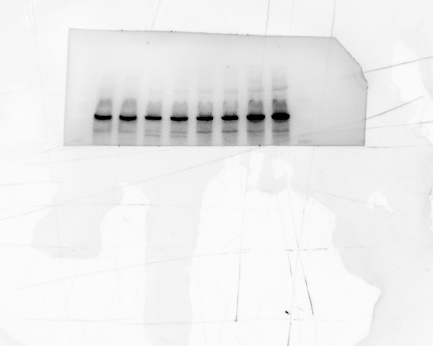

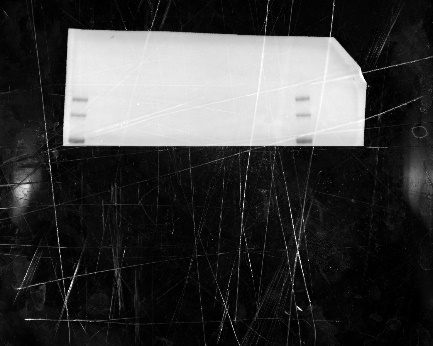

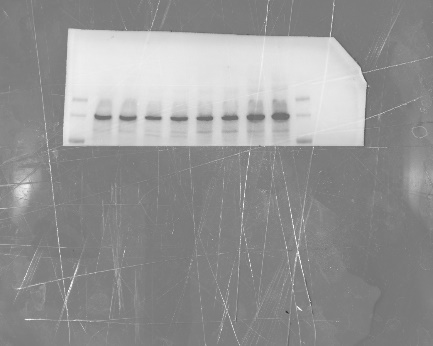


β-actin


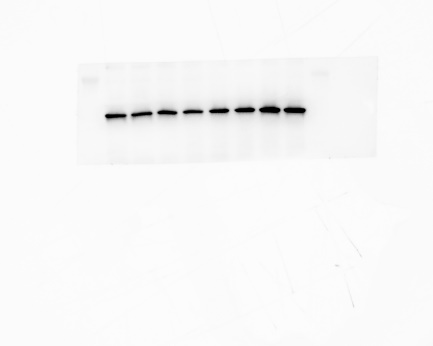

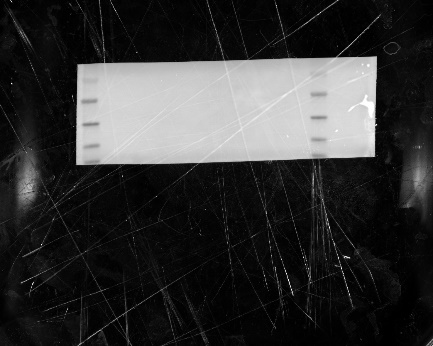

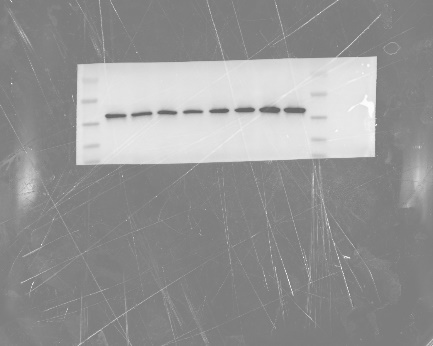


merge

25kDa

35kDa

40kDa

55kDa

70kDa

100kDa

130kDa

180kDa


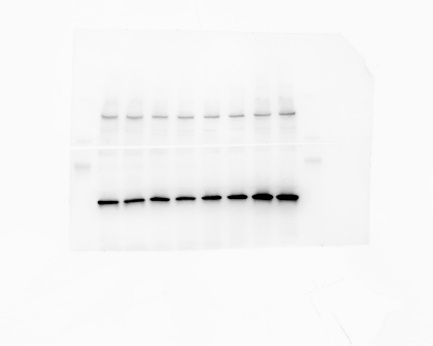

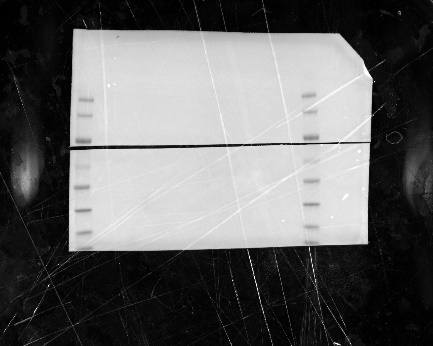

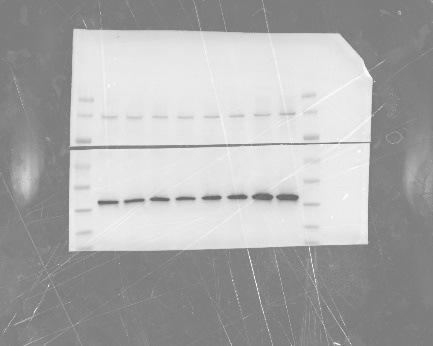


Tlr4


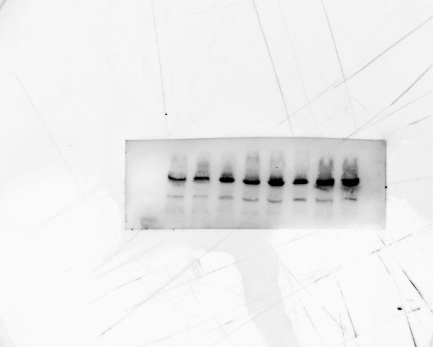

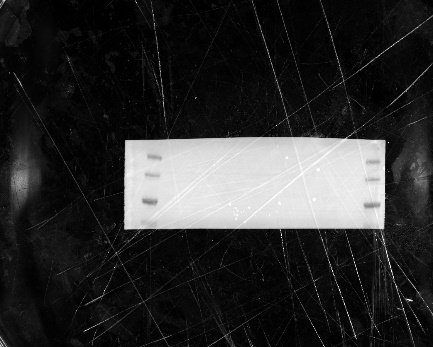

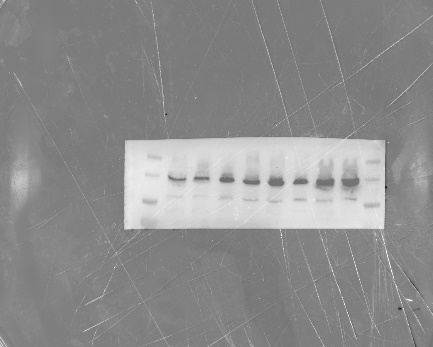


β-actin


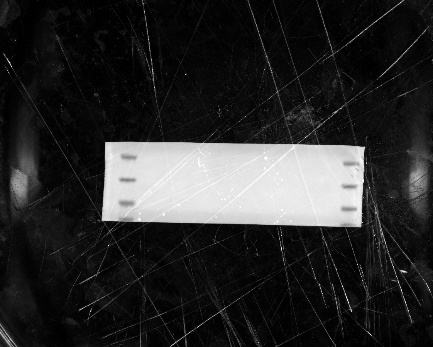

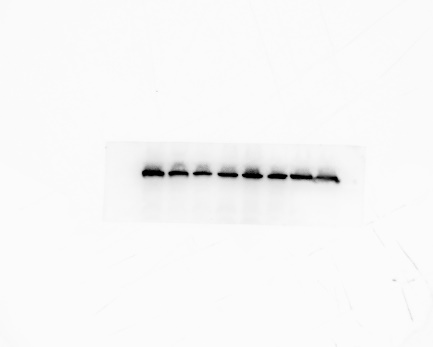

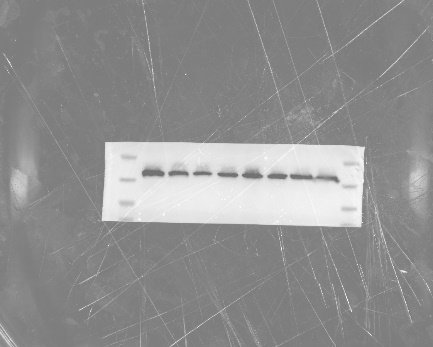


merge

180kDa

130kDa

100kDa

70kDa

55kDa

35kDa

40kDa


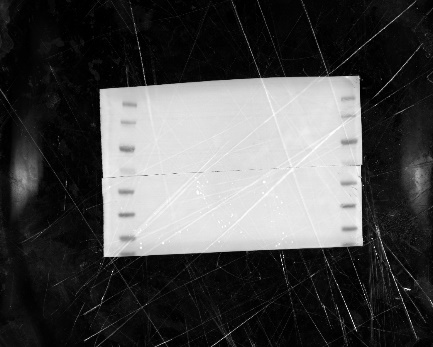

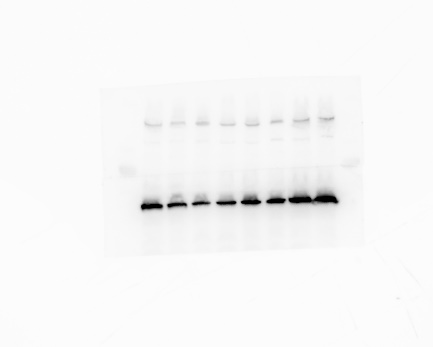

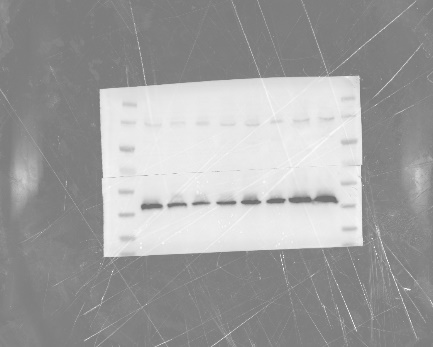


p53


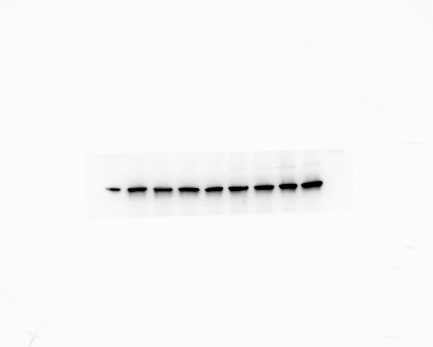

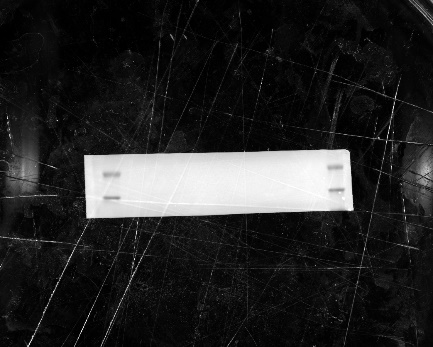

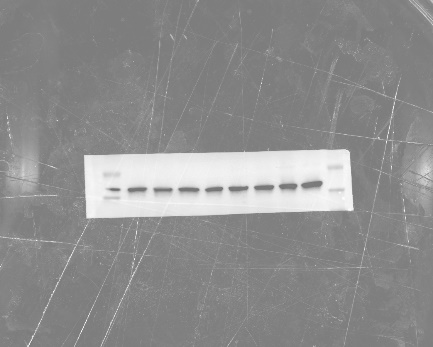


vinculin


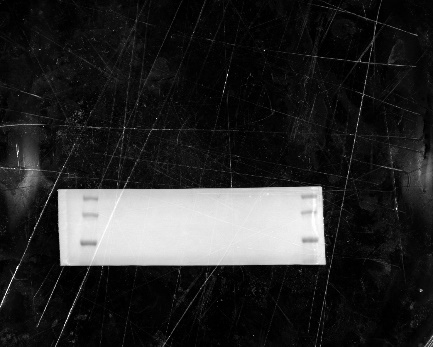

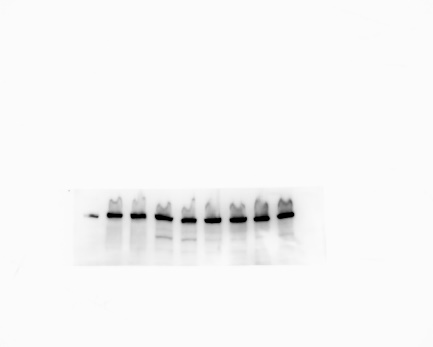

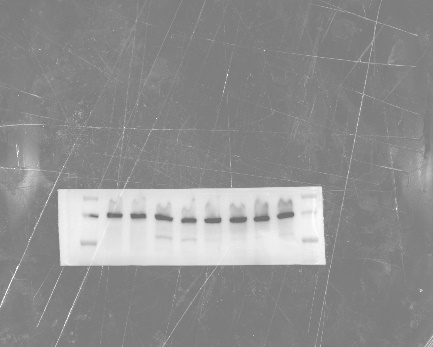


merge

15kDa

25kDa

35kDa

40kDa

55kDa

70kDa

100kDa

130kDa

180kDa


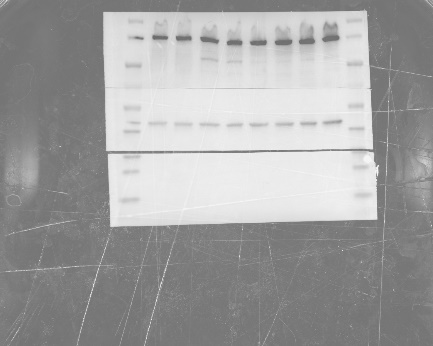

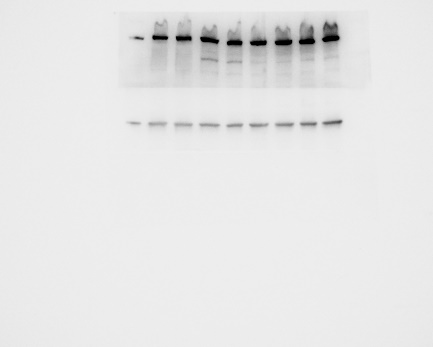

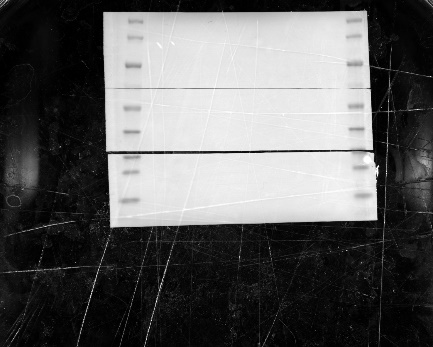


Nrf2


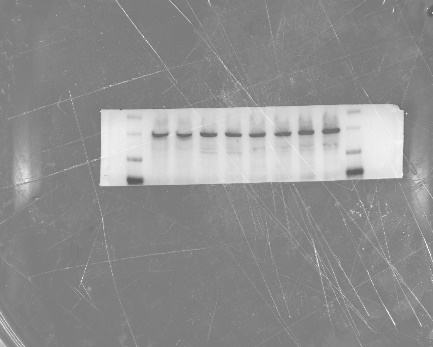


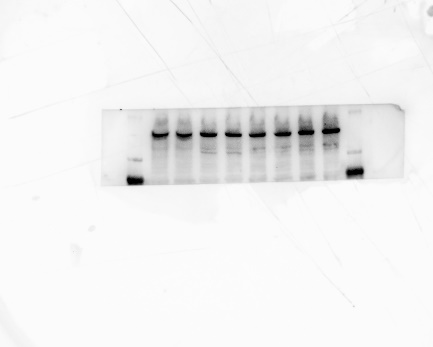

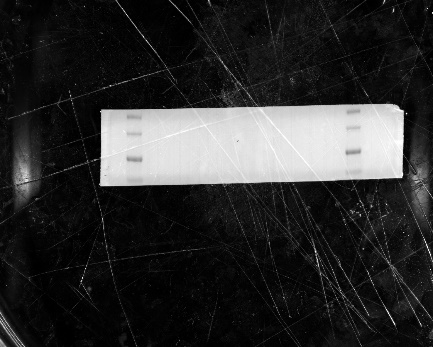


β-actin


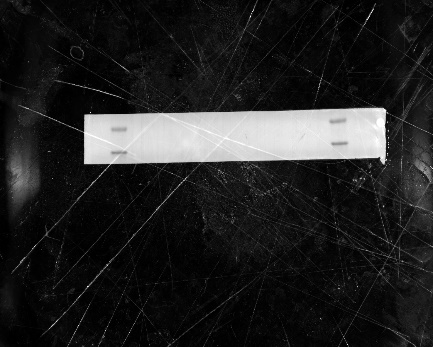

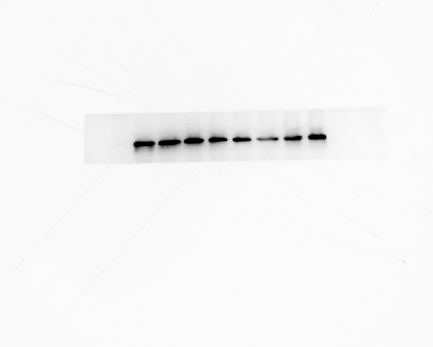

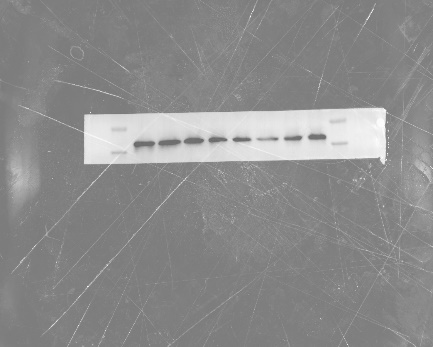


merge

25kDa

15kDa

35kDa

40kDa

55kDa

70kDa

100kDa

130kDa

180kDa


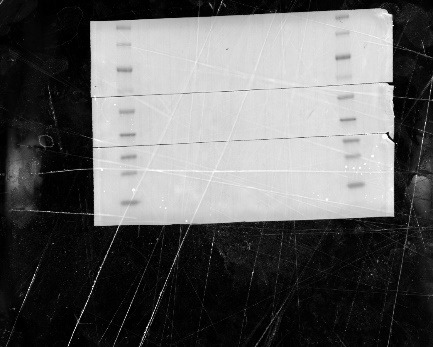

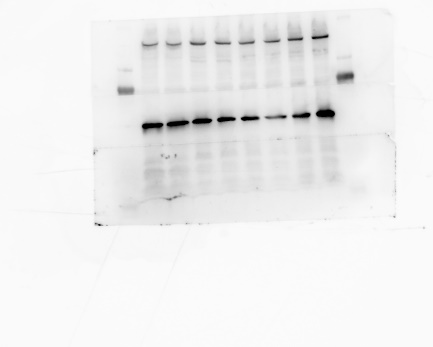

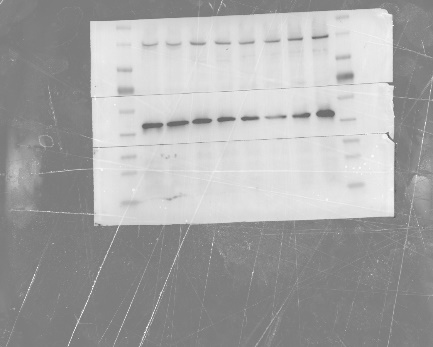


Hmox1


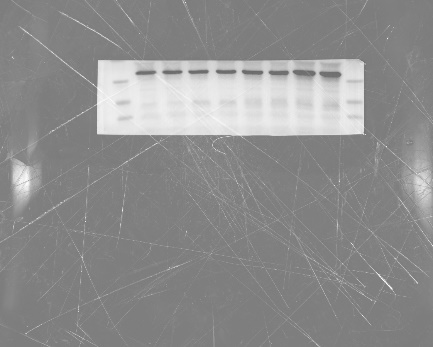

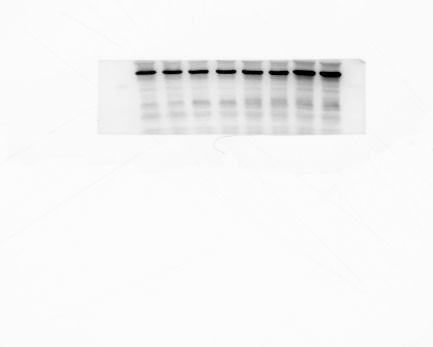

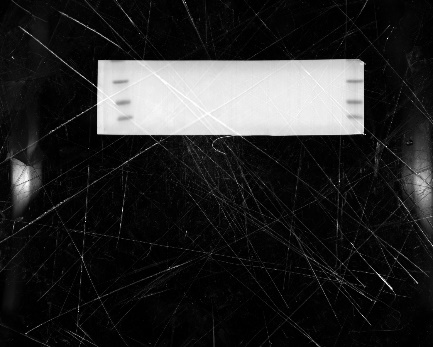


Vinculin


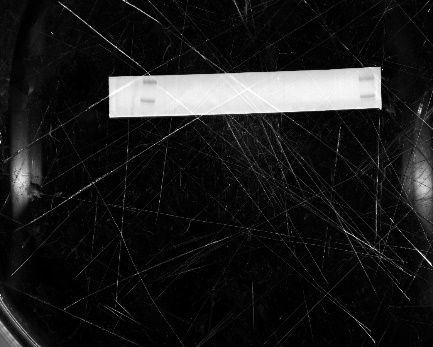

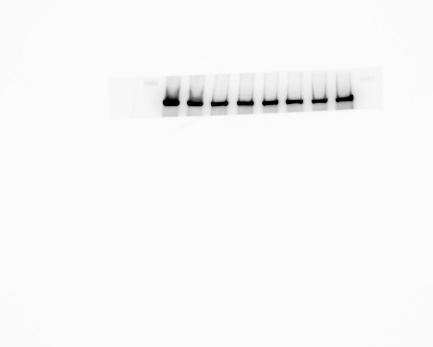

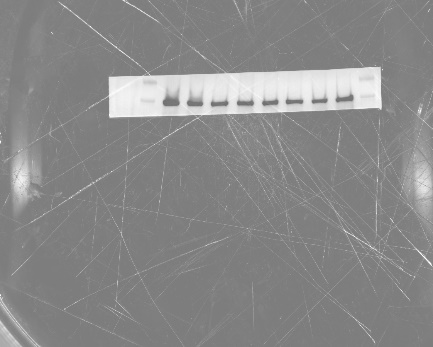


Merge

25kDa

35kDa

40kDa

55kDa

70kDa

100kDa

130kDa

180kDa


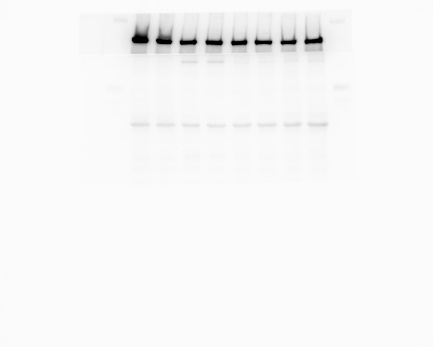

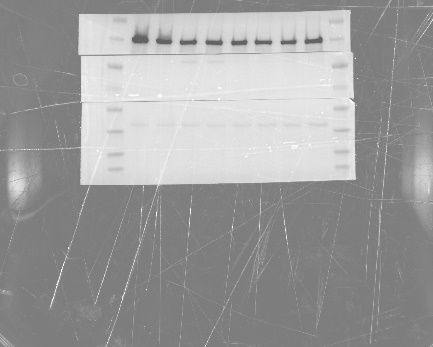

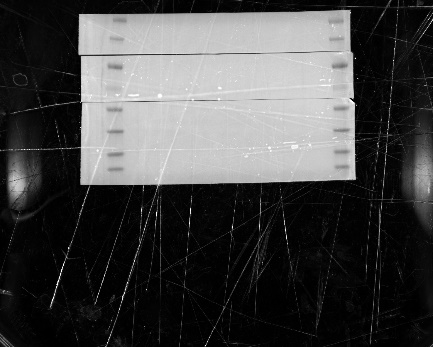


GPX4


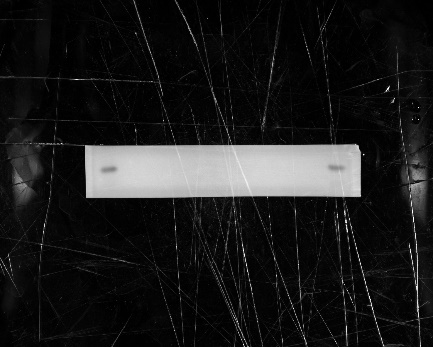

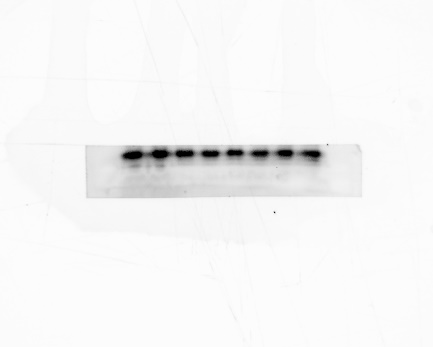

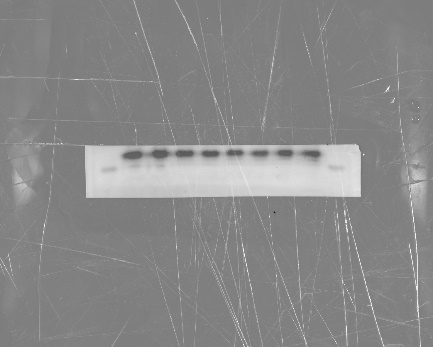


Vinculin


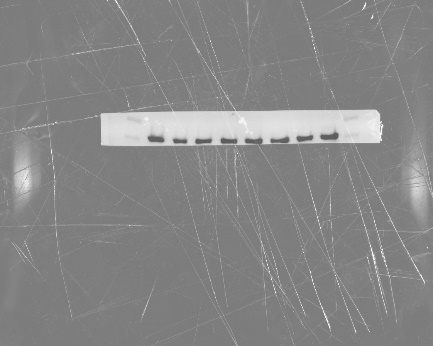

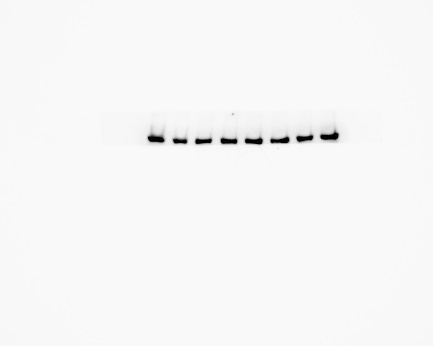

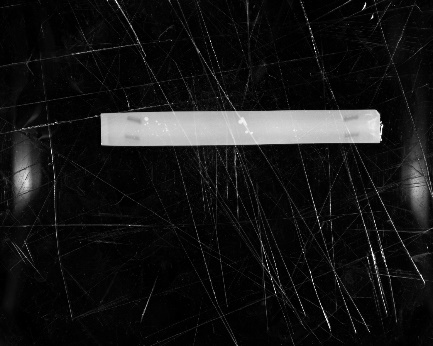


Merge

15kDa

25kDa

35kDa

40kDa

55kDa

70kDa

100kDa

130kDa

180kDa


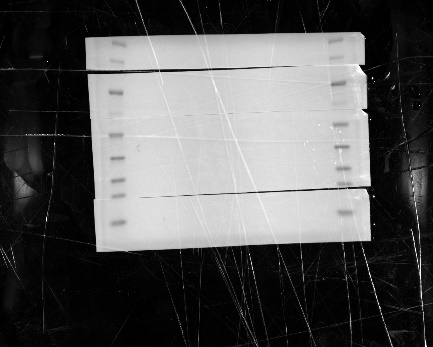

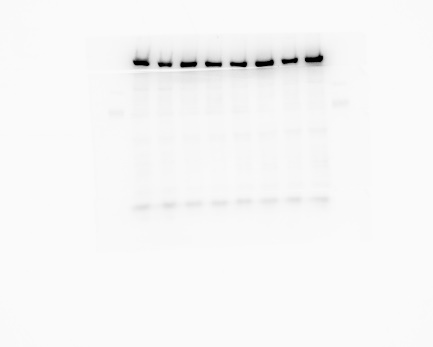

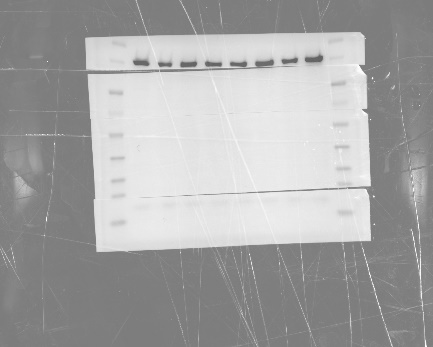


Slc7a11


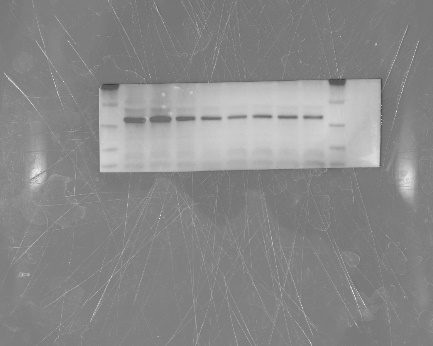

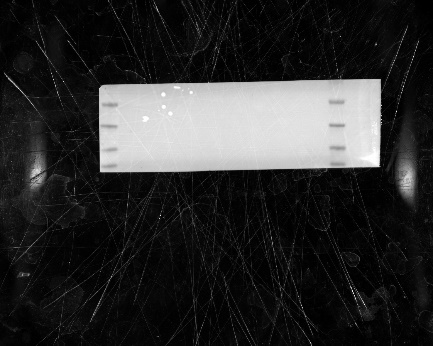

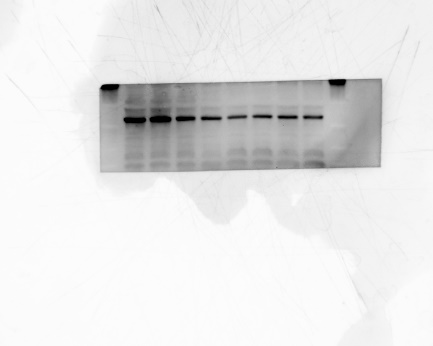


Vinculin


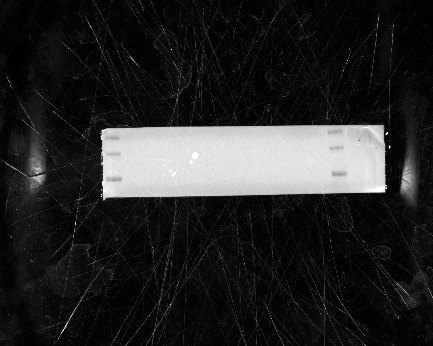

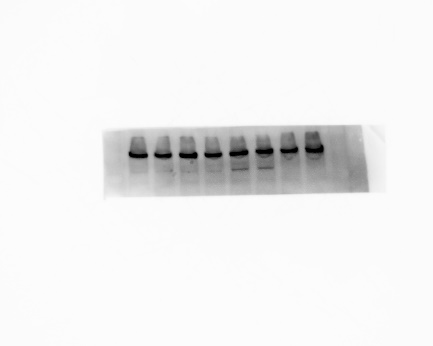

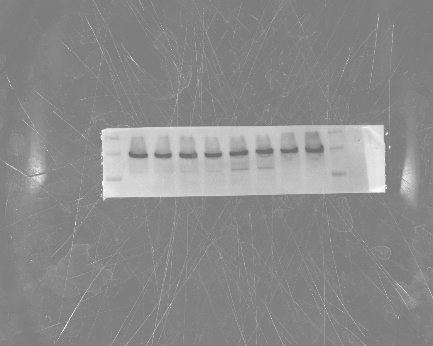


Merge


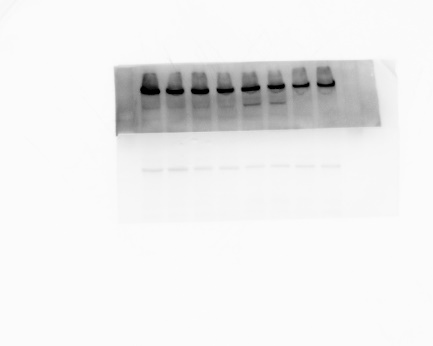

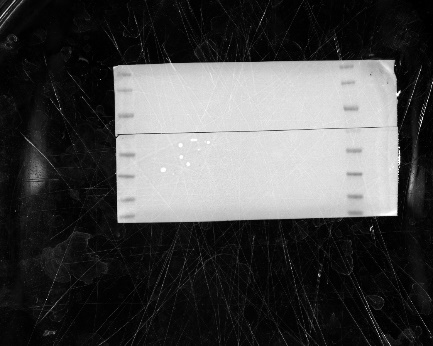

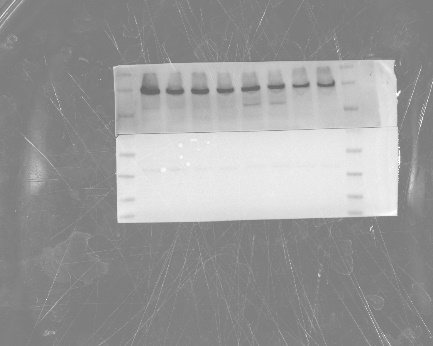


35kDa

40kDa

55kDa

70kDa

100kDa

130kDa

180kDa

Fth1


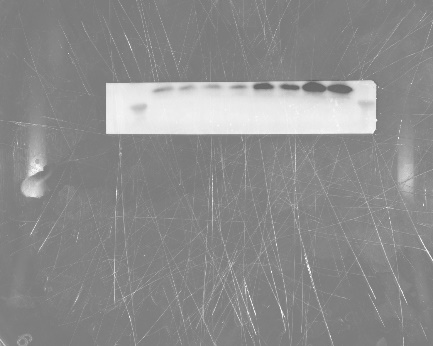

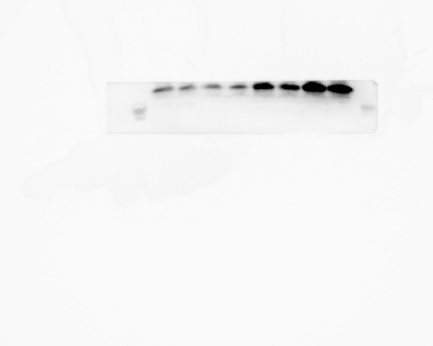

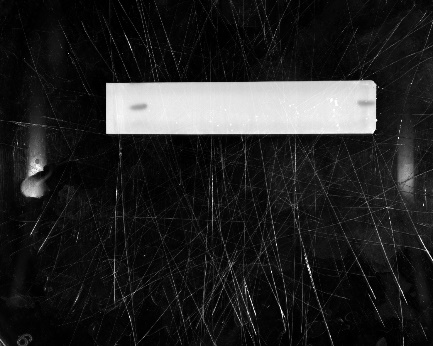


β-actin


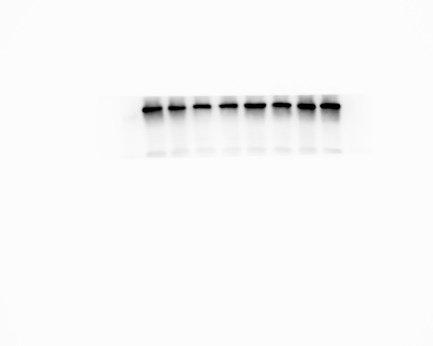

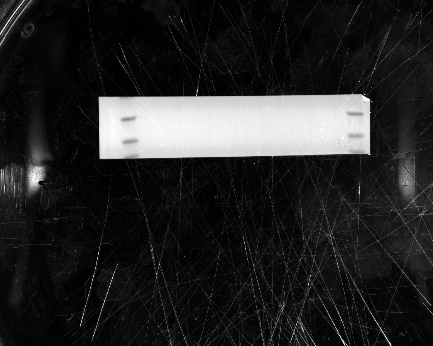

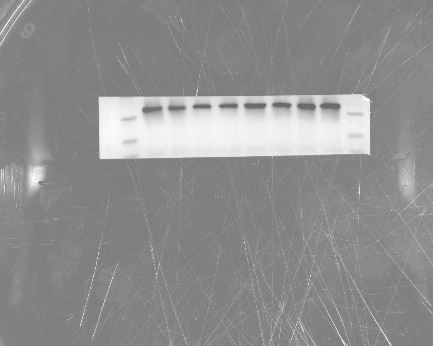


merge


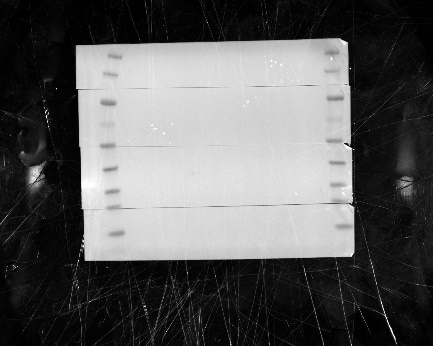

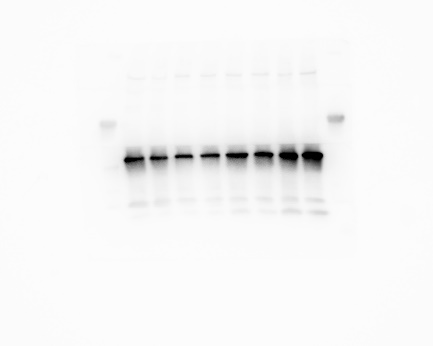

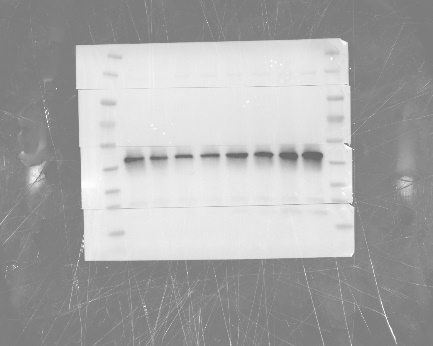


15kDa

25kDa

35kDa

55kDa

40kDa

70kDa

100kDa

130kDa

180kDa

FTL


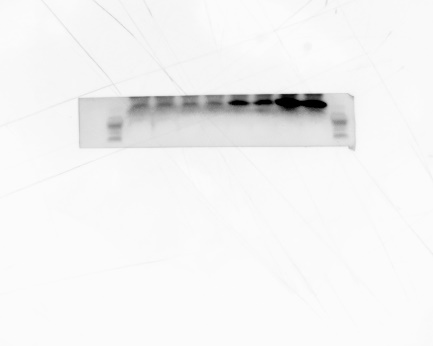

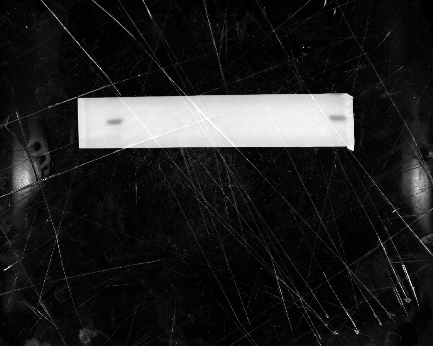

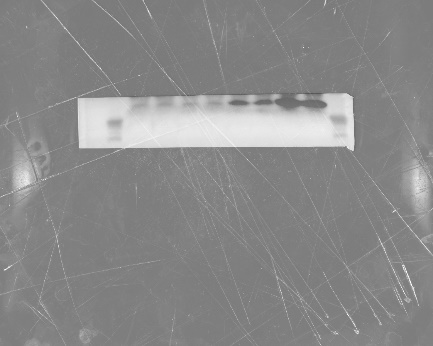


Vinculin


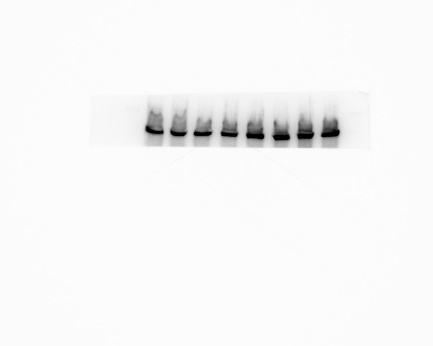

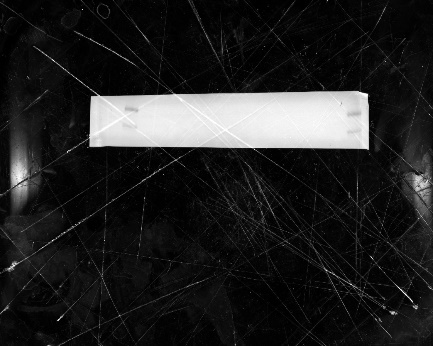

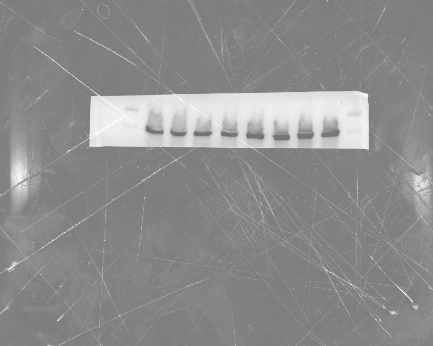


Merge

15kDa

25kDa

35kDa

40kDa

55kDa

70kDa

100kDa

130kDa

180kDa


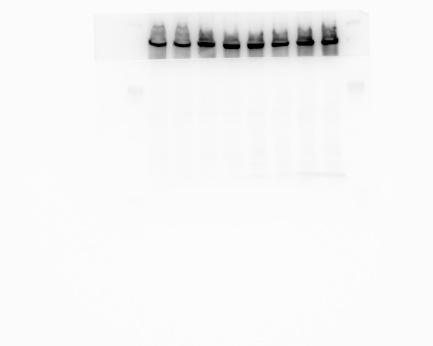

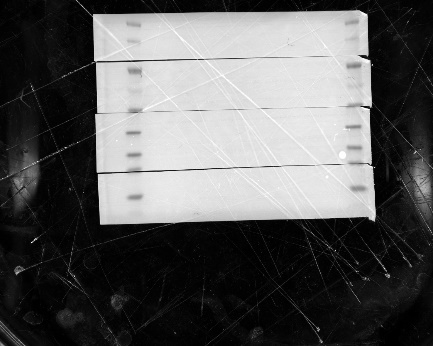

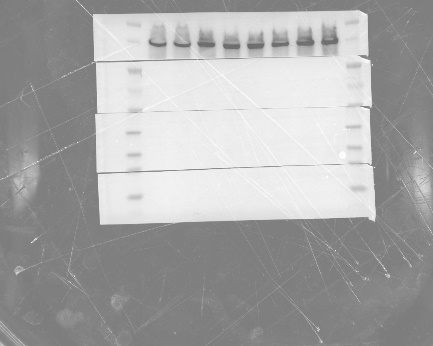

Supplement: Supplementary file 1 [file DataSheet1.zip › wb.docx]
